# *C*_4_-Symmetric Bowl-Shaped Diruthenium Tetracarboxylate Catalysts for Enantioselective C–H Functionalization Using Donor/Acceptor Carbenes

**DOI:** 10.1021/acscatal.5c01052

**Published:** 2025-03-27

**Authors:** Joshua K. Sailer, John Bacsa, Huw M. L. Davies

**Affiliations:** Department of Chemistry, Emory University, 1515 Dickey Drive, Atlanta, Georgia 30322, United States

**Keywords:** C−H functionalization, aryldiazoacetates, diruthenium tetracarboxylates, asymmetric catalysis, dirhodium, cyclopropanation

## Abstract

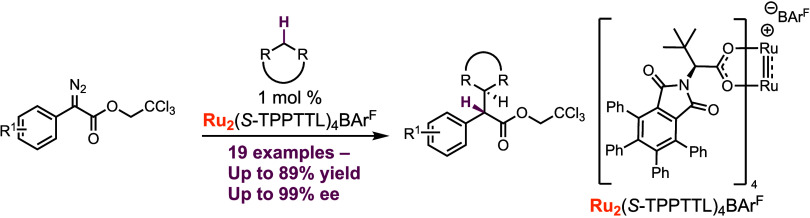

Cationic diruthenium (II,III) tetracarboxylate catalysts have been shown to catalyze selective intermolecular C–H functionalization reactions using donor/acceptor carbenes in high yield and with high levels of enantioselectivity. The diruthenium catalysts were compared to the analogous dirhodium (II,II) tetracarboxylate and showed similar levels of enantioselectivity for most reactions. A distinctive feature of the diruthenium catalysts is a greater preference for C–H functionalization over cyclopropanation compared to the corresponding dirhodium catalysts. Also, the diruthenium catalysts have a greater preference for sterically more accessible sites compared with their dirhodium counterparts. These studies show that the diruthenium catalysts are generally effective catalysts for enantioselective intermolecular C–H functionalization, but further optimization would be needed for them to match the dirhodium catalysts in terms of functional group compatibility, turnover frequency, and turnover numbers.

## Introduction

**Scheme 1:**
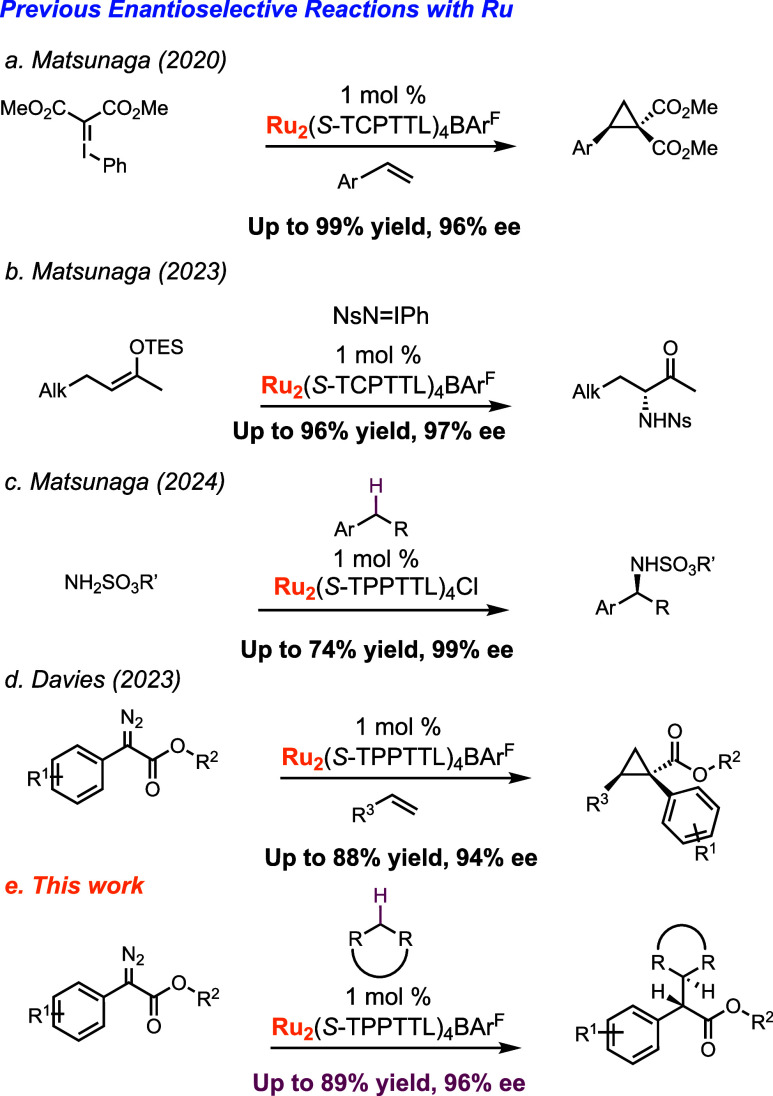
Current and Previous Works

Selective C–H functionalization has emerged as an invaluable synthetic tool for the organic chemist in the past three decades.^1−6^ Reimagining C–H bonds as functional groups allows one to draw upon the subtle differences between them, enabling highly selective reactions. A powerful method for C–H functionalization is the use of metallo-carbene intermediates, commonly generated via the decomposition of diazo compounds.^7−9^ Dirhodium tetracarboxylate complexes, in particular, have been shown to catalyze the decomposition of aryldiazoacetates to generate donor/acceptor metallo-carbenes capable of intermolecular C(sp^3^)–H functionalization in a highly regio-, enantio-, and diastereoselective manner.^10,11^ The Davies group has developed a toolbox of catalysts, each capable of selectively functionalizing a unique C–H bond from a variety of alkane substrates.^12−14^ Studies of intermolecular C–H functionalization with other catalysts derived from less expensive metals often do not extend to asymmetric catalysis,^15−21^ although there have been some exciting advances using bioengineered enzymes.^22−26^ The most promising nonenzymatic complexes have been Rh–Bi tetracarboxylate complexes which have been shown by Davies to be capable of similar levels of enantioinduction to the dirhodium catalysts but are about 100 times slower,^27^ and by Fürstner to be highly effective for enantioselective intermolecular C–H functionalization of methyl ethers.^28^ Even though our most practical C–H functionalization approach has been with dirhodium catalysts under ultralow catalyst loading conditions (<0.001 mol %),^29^ we are still interested in exploring cheaper alternatives to dirhodium. Thus, we were intrigued by a report by Matsunaga on the synthesis of chiral diruthenium paddlewheel complexes and their application toward carbene insertion reactions.^30^ Using an iodonium ylide as the carbene precursor, the diruthenium catalysts are capable of catalyzing the cyclopropanation of activated olefins in excellent yield with high levels of enantioselectivity (Scheme 1a). More recently, they have shown that these diruthenium catalysts are effective in enantioselective intermolecular C–H amination of silyl enol ethers and benzylic sites (Scheme 1b,c).^31^

Inspired by their initial work, our lab studied the synthesis, characterization, and application of five novel diruthenium paddlewheel complexes.^32^ These complexes were shown to be capable of the cyclopropanation of a variety of alkenes using aryldiazoacetates as the carbene precursors in up to 88% yield with 94% ee (Scheme 1c). We now describe the use of these catalysts in enantioselective C–H functionalization and compare their efficiency and selectivity profiles to the corresponding dirhodium catalysts (Scheme 1d).

**Figure 1:**
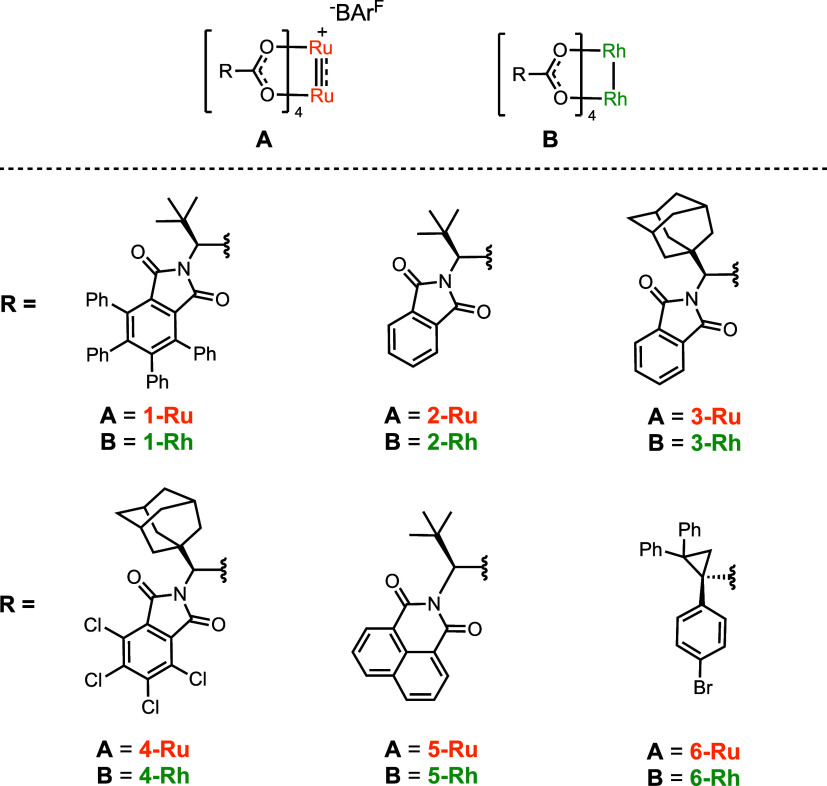
Structure of tetracarboxylate catalysts.

In our previous cyclopropanation studies, we examined the synthesis and characterization of six bowl-shaped C_4_-symmetric catalysts, Ru_2_(S-TCPTTL)_4_BAr^F^, the catalysts used by Matsunaga, and five new ones, Ru_2_(S-TPPTTL)_4_BAr^F^ (**1-Ru**), Ru_2_(S-PTTL)_4_BAr^F^ (**2-Ru**), Ru_2_(S-PTAD)_4_BAr^F^ (**3-Ru**), Ru_2_(S-TCPTAD)_4_BAr^F^ (**4-Ru**), and Ru_2_(S-NTTL)_4_BAr^F^ (**5-Ru**), related to previously studied rhodium catalysts (**1–5-Rh**) (Figure 1).^32^ The corresponding rhodium catalysts adopt a C_4_-symmetric bowl-shaped structure and are not considered to be particularly sterically demanding near the carbene, but the wall of the bowl can cause subtle control of site selectivity.^12^ The X-ray structures of **1–5-Ru** catalysts show that the ligands have self-assembled in a way similar to that of the dirhodium complexes, resulting in C_4_-symmetric bowl-shaped structures. The optimum ruthenium catalyst in the cyclopropanation reactions was **1-Ru**.

## Methods

**Scheme 2:**
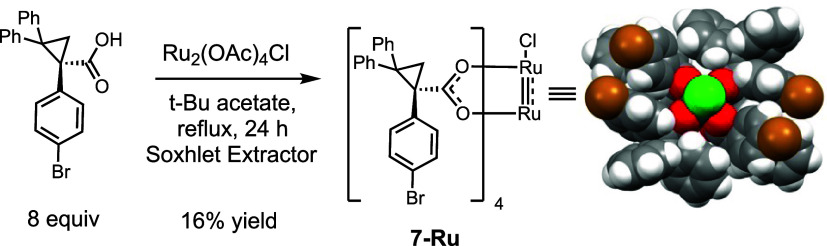
Synthesis of Ru_2_(*S*-*p*BrTPCP)_4_Cl

In order to expand the potential selectivity profile of the diruthenium catalysts, we prepared a sixth catalyst, Ru_2_(*S*-*p*BrTPCP)_4_ BAr^F^ (**6-Ru**). The corresponding rhodium catalyst (**6-Rh**) is sterically demanding and tends to cause C–H functionalization reactions to occur at sterically less crowded C–H bonds, even when they are electronically less favored.^33,34^ The synthesis of **6-Ru** was performed with a slight modification to the standard ligand exchange procedure, using *tert*-butylacetate as the solvent. The reaction was not as clean as it was for the bowl-shaped catalysts **1–5-Ru** but after extensive purification by chromatography followed by recrystallization provided the chloro diruthenium catalyst **7-Ru** in 16% yield (Scheme 2). Gratifyingly, the X-ray structure showed that the complex was C_2_-symmetric, very similar to what had been observed for the corresponding rhodium catalyst **6-Rh**.^34^ Treatment of **7-Ru** with sodium BAr^F^ resulted in replacement of the chloride to form **6-Ru** in 96% yield.

We began testing the reactivity of these complexes by comparing the diruthenium and dirhodium catalysts in reactions with substrates chosen to establish key similarities and differences between the ruthenium and rhodium catalysts. The first substrate screened was *p*-cymene (**8**) (Table 1). This substrate offers competition between a tertiary and primary C–H bond allowing us to gain an understanding of the electronic and steric influence of each catalyst tested.^33^ The bowl-shaped diruthenium catalysts **2–5-Ru** strongly preferred reaction at the primary C–H bond giving **9** in >20:1 r.r. However, the enantioselectivity was variable and in some cases the diruthenium catalysts generated preferentially the opposite enantiomer to the one formed with dirhodium catalysis. The reaction with the PTAD catalyst **3-Ru** gave the highest level of asymmetric induction (86% ee) (Table 1a). In contrast, the reaction with the TPPTTL catalyst **1-Ru** and the TPCP catalyst **6-Ru** was not very effective. The overall yield was low, and the site selectivity and enantioselectivity were considerably inferior to the reactions with the other diruthenium catalysts. In general, it was found that C–H functionalization of substrates containing an aryl group was relatively sluggish and 5 mol % diruthenium catalyst loading was required for complete conversion of the diazo compound. The corresponding dirhodium-catalyzed reaction, which can be readily conducted with 1 mol % catalyst loading, showed some interesting differences with the diruthenium system (Table 1b), most notably the preferred formation of the opposite enantiomer to that formed under diruthenium catalysis. The bowl-shaped catalysts **1–5-Rh** showed a preference for reaction at the tertiary site to form **10** with the TPPTTL catalyst (**1-Rh**) giving the best selectivity (4.7:1 r.r.). The TPCP catalyst **6-Rh** was originally designed as a sterically demanding catalyst, and it causes the site selectivity to strongly favor the primary site forming **9** with >20:1 r.r.

**Table 1:**
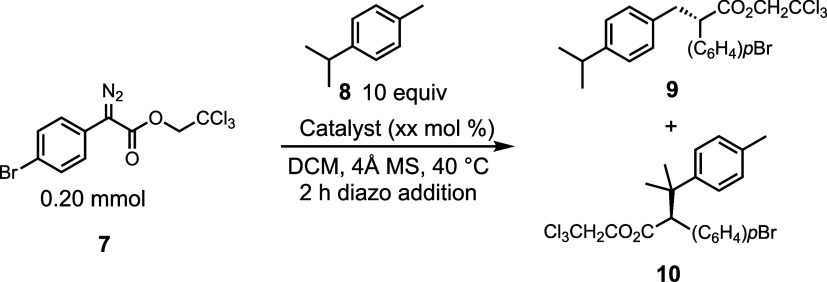

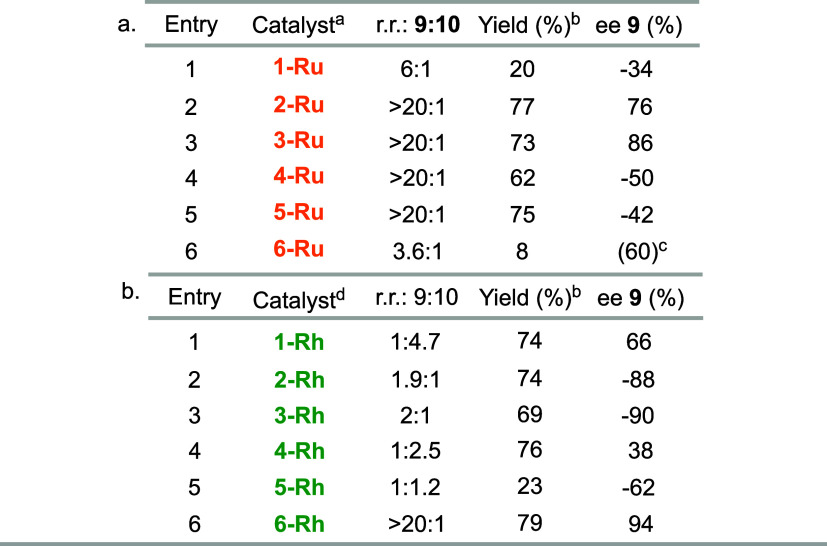
Catalyst Screen with *p*-Cymene

a Catalyst loading at 5.0 mol %.

b Combined yield.

c Estimated level of enantioselectivity due to overlapping signals in the chiral HPLC.

d Catalyst loading at 1.0 mol %. Diastereomeric ratios were determined by crude NMR analysis. Enantiomeric excess was determined by chiral HPLC analysis. Isolated yields are reported. Negative value for the ee indicates that the opposite enantiomer to the drawn structure is preferentially formed.

Next, we conducted a catalyst screen on 4-isoproylethylbenzene (**11**) to compare the site selectivity between secondary and tertiary C–H bonds (Table 2). In general four of the bowl-shaped diruthenium catalysts **1–5-Ru** gave selective reactions (>20:1 r.r.) for the secondary sites to form a diastereomeric mixture of **12a** and **12b** in a ratio of 3–5:1 (Table 2a). The enantioselectivity varied tremendously between the various catalysts, and in some cases, the diruthenium catalysts generated preferentially the opposite enantiomer to the one formed with dirhodium catalysis. The best diruthenium catalyst overall was the TPPTTL derivative **1-Ru**, which gave excellent site selectivity (>20:1 r.r.) and both the diastereomers **12a** and **12b** were generated with high enantioselectivity (94% ee). The TPCP catalyst **6-Ru** was not an effective catalyst in this reaction, giving low yield and virtually racemic products. The dirhodium catalysts had similar selectivity trends, with some subtle differences (Table 2B). In general, the site selectivity was lower with the bowl-shaped dirhodium catalysts **1–5-Rh** (Table 2B, entries 1–5) compared to the corresponding diruthenium catalysts. For most of the catalysts, the diastereoselectivity was similar to that of the diruthenium catalysts except for the TPPTTL derivative **1-Rh**, which gave a 12:1d.r favoring **12a**. This catalyst also gave the highest enantioselectivity, generating **12a** in 94% ee, but the site selectivity was only moderate (6.5:1 r. r.).

**Table 2:**
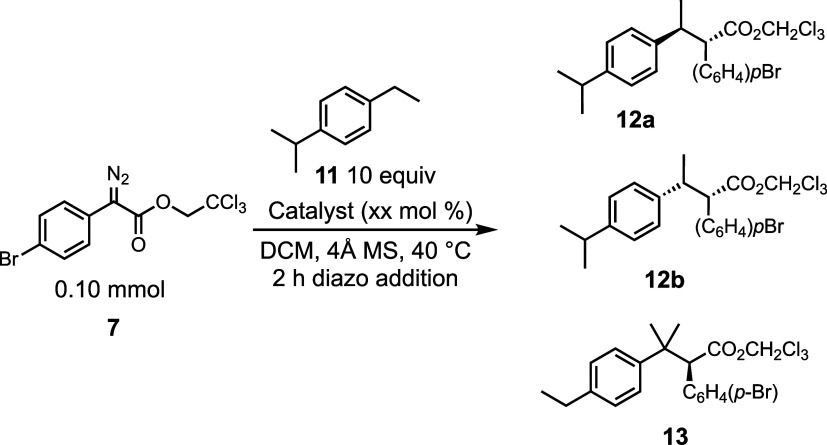

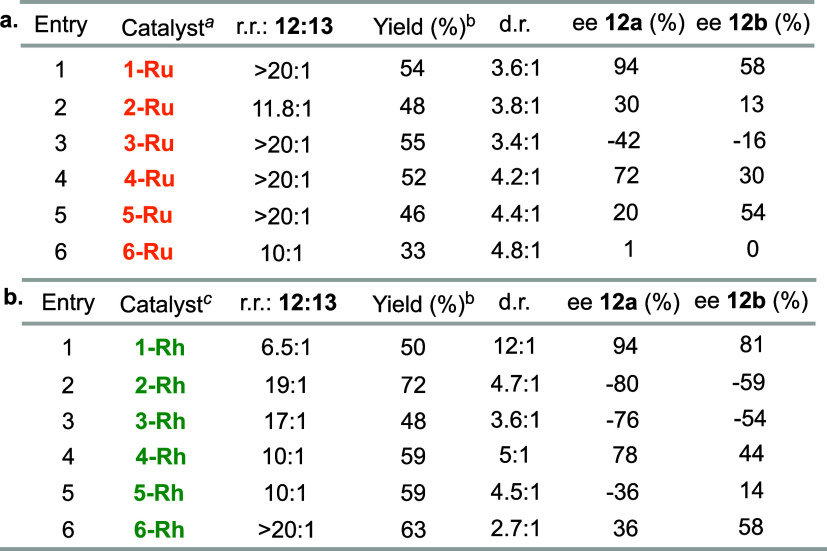
Catalyst Screen with 4-Isopropylethylbenzene

a Catalyst loading at 5.0 mol %.

b Combined yield.

c Catalyst loading at 1.0 mol %. Diastereomeric ratios were determined by crude NMR analysis. Enantiomeric excess was determined by chiral HPLC analysis. Isolated yields are reported. Negative value for the ee indicates that the opposite enantiomer to the drawn structure is preferentially formed.

One of the most impressive aspects of the rhodium-catalyzed reactions of aryldiazoacetates is their great effectiveness in intermolecular functionalization of unactivated C–H bonds.^29^ Therefore, we conducted an evaluation of the efficiency of these catalysts in the C–H functionalization of cyclohexane (Table 3). All of the bowl-shaped diruthenium catalysts **1–5-Ru** gave the C–H functionalization in good yield (70–80%) and even though the cyclohexane would be expected to be less active compared to the benzylic systems, these reactions could be readily conducted with 1 mol % catalyst loading (Table 3A, entries 1–5). The TPCP derivative **6-Ru** was once again not an efficient catalyst even with 5% catalyst loading, with only trace product being seen (Table 3A, entry 6). The optimum catalyst in terms of asymmetric induction is the TPPTTL derivative **1-Ru** (95% ee), and it compares well with the rhodium analogue **1-Rh** (94% ee, Table 3B, entry 1).^29^

**Table 3:**
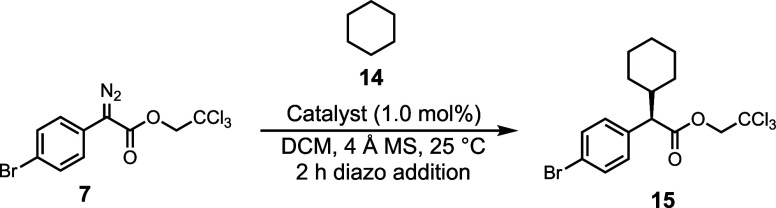

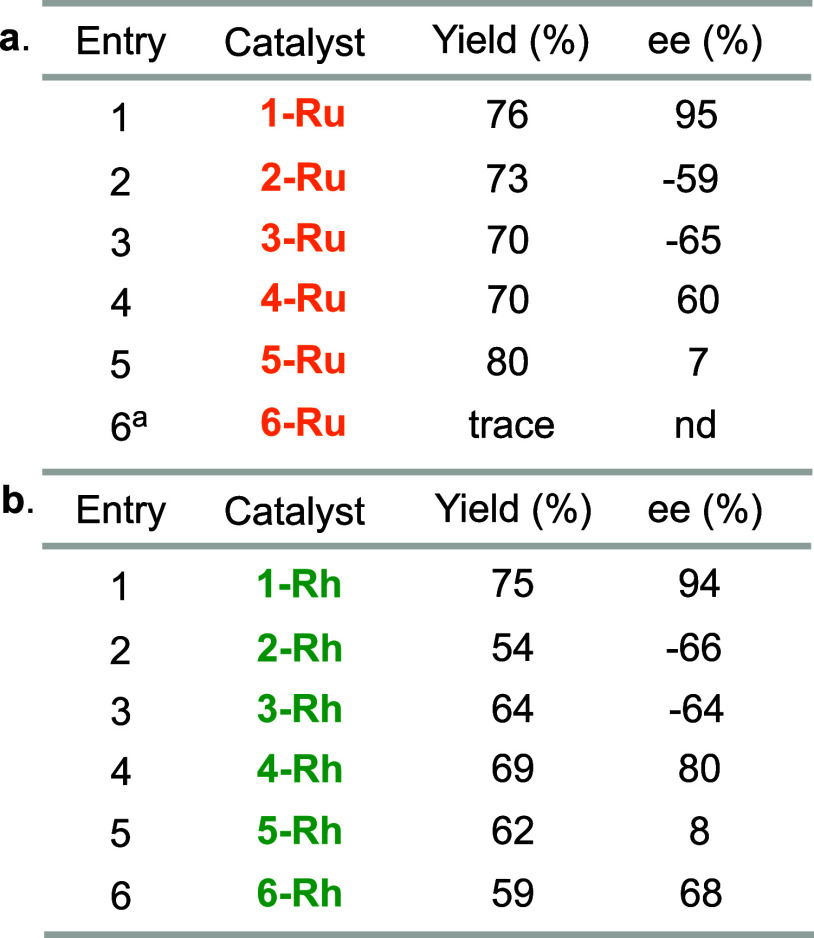
Catalyst Screen with Cyclohexane

a Catalyst loading of 5.0 mol % in refluxing cyclohexane as solvent. Enantiomeric excess was determined by chiral HPLC analysis. Isolated yields are reported. Negative value for the ee indicates that the opposite enantiomer to the drawn structure is preferentially formed.

The three sets of evaluations revealed that the diruthenium bowl-shaped catalysts gave enhanced site selectivity for less crowded sites compared to the corresponding dirhodium catalysts, but it was not possible to extend this selectivity profile by using a sterically crowded ligand because TPCP catalyst **6-Ru** was not an efficient catalyst. The reason for the poor performance of **6-Ru** is uncertain at this time, but it could be because the BAr^F^ counterion still interferes with the approach of the substrates or the catalysts are not stable under the reaction conditions. The ruthenium-catalyzed reactions of cyclohexane can be conducted at 1 mol % catalyst loading, but in the benzylic C–H functionalization, 5 mol % catalyst loading is required. For secondary C–H functionalization, the optimum diruthenium catalyst for high asymmetric induction is the TPPTTL derivative **1-Ru** and it compares well with its dirhodium counterpart **1-Rh**. Similarly, Matsunaga found that **1-Ru** was the optimum catalyst for enantioselective C–H amination,^31^ further demonstrating the effectiveness of this TPPTTL ligand in enantioselective group transfer reactions.

Having established that **1-Ru** is the optimum catalyst for secondary C–H functionalization, its utility with a range of substrates was examined (Table 4). The results of the reaction with **1-Ru** are shown in orange, and the comparison results with **1-Rh** are shown in green. In all of these reactions, both diruthenium and dirhodium catalysts preferentially generated the same enantiomer. The reaction can be applied to a range of cycloalkanes with high asymmetric induction (90–96% ee) as illustrated in the formation of **16–18**. Aryldiazoacetates react preferentially with adamantane at the tertiary site,^35^ and this is also the case in the **1-Ru**-catalyzed reaction, forming **19** in 81% yield and 92% ee.

**Table 4:**
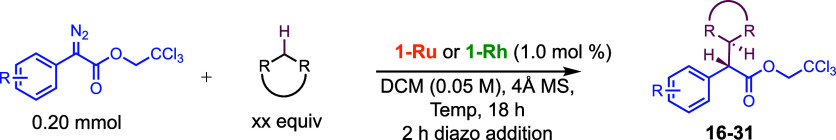

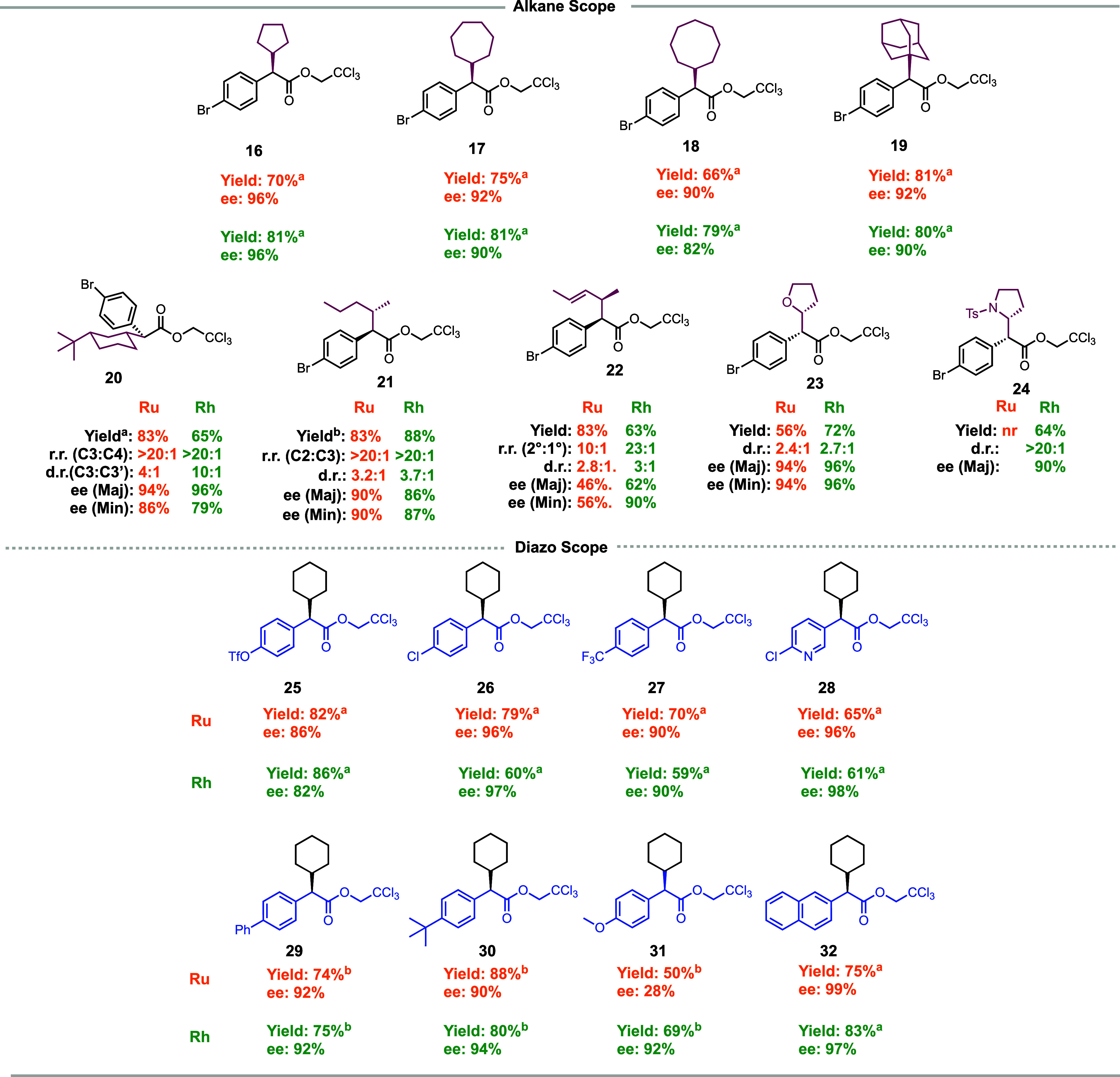
Substrate Scope of C–H Insertion Reactions

a Reactions run at 25 °C.

b Reactions run neat at refluxing temperature. Diastereomeric ratios were determined by crude NMR analysis. Enantiomeric excess was determined by chiral HPLC analysis. Isolated yields are reported.

The next series of substrates challenged the site selectivity and diastereoselectivity of the C–H functionalization. One of the signature reactions with **1-Rh** is the desymmetrization of *tert*-butylcyclohexane by C–H functionalization at C3 to form **20** with high levels of site selectivity, diastereoselectivity and enantioselectivity.^36^ The parallel reaction with **1-Ru** was also quite effective. The reaction was highly site-selective for C3 (>20:1 r. r.) and highly enantioselective (93% ee), but the diastereoselectivity had dropped from 10:1 d. r. to 4:1 d. r. The next classic substrate is pentane, which has been shown to be cleanly functionalized at C2 when bulky dirhodium catalysts were used.^37^ Both **1-Ru** and **1-Rh** gave very similar results, forming **21** with high site selectivity for C2 (>20:1 r. r.), moderate diastereocontrol (3–4:1 d. r.), and relatively high levels of asymmetric induction (86–90% ee). The next substrate, *trans*-2-hexene, has been shown to be efficiently functionalized at either the 1° or 2° allylic C–H site, depending on the steric environment present in the rhodium catalyst.^38^ Both **1-Ru** and **1-Rh** were selective for 2° functionalization, giving **22** in 10:1 r. r. and 23:1 r. r., respectively. The selective formation of **22** indicates that neither of the catalysts are behaving as an extremely crowded catalyst,^33^ but the lower ratio of secondary products with **Ru-1** shows once again that the diruthenium catalysts have a greater preference for the less crowded site compared to dirhodium.

The next two substrates examined how the presence of heteroatoms would affect the efficiency of the Ru-catalyzed C–H functionalization. One of the distinctive features of dirhodium-catalyzed C–H functionalization is the range of functional groups that can be accommodated,^12^ but this would not necessarily be the case with the diruthenium catalysts, especially as they are cationic species. The reaction with tetrahydrofuran proceeded uneventfully, with both **1-Ru** and **1-Rh** resulting in C–H functionalization at C2 to form **23** with relatively low diastereoselectivity (2–3:1) but high enantioselectivity (94–96% ee). For an effective reaction in the **1-Ru**-catalyzed process, the reaction needed to be conducted at higher temperatures in trifluorotoluene as the solvent, indicating that the oxygen functionality may be interfering with the catalytic performance. This effect was more drastic in the reaction with *N*-tosylpyrrolidine because **1-Ru** failed to form any of the C–H functionalization product **24**, but **1-Rh** generated **24** in 64% yield. The site selectivity and functional group compatibility is indicative that the ruthenium carbene is behaving as if it is more electrophilic, capable of effective reactions at secondary sites and interference from nucleophilic sites.

Having determined the selectivity profile with *p*-bromophenyldiazoacetate as the carbene source, the C–H functionalization of cyclohexane was examined with a range of aryldiazoacetates to see if the similar enantioselectivity trends of **1-Ru** and **1-Rh** are independent of aryldiazoacetate structures. The resulting products **25–32** were formed in high yields and high levels of asymmetric induction (87–96% ee), except for the *p*-methoxy derivative **31** (28% ee). The high yielding formation of **29–32** required conducting the reactions under reflux conditions, and it is known that an electron-donating group on an aryldiazoacetate causes nitrogen extrusion to occur at lower temperatures,^39^ and so, it is likely that the low enantioselectivity in the formation of **31** is due to a background thermal and non-metal-catalyzed reaction.

**Scheme 3:**
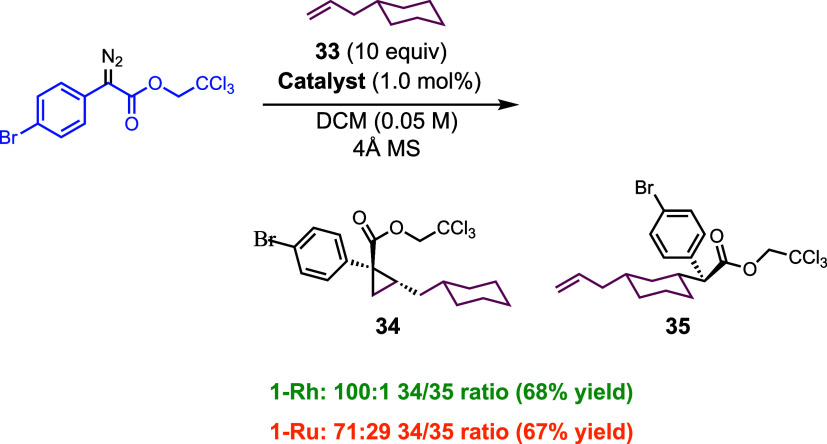
Product 35 Was Found to Be Inseparable on Chiral HPLC Analysis

During the course of examining the reaction with substituted cyclohexanes, we made a serendipitous discovery that the diruthenium catalysts have a distinctly different profile to the dirhodium catalysts in selectivity between C–H functionalization and cyclopropanation (Scheme 3). The reaction with the alkenylcyclohexane **33** would be expected to undergo clean cyclopropanation to form **34** because it is well established that a monosubstituted alkene is sterically very accessible for cyclopropanation with donor/acceptor carbenes.^40^ In the **1-Rh**-catalyzed reactions, this is indeed the case and the cyclopropane **34** is the major product with only a trace of the C–H functionalization product observed (100:1 **34**/**35** ratio). In contrast, the reaction catalyzed with **1-Ru** gave a significant amount of the C–H functionalization product **35** as well as the cyclopropane **34** (71:29 **34**/**35** ratio).

The further evaluation of the selectivity difference was examined in competition reactions between cyclohexane (10 equiv) and 1-hexene (2 equiv) with representative examples of aryldiazoacetates (Table 5). In the **1-Ru**-catalyzed reactions, the C–H functionalization of cyclohexane was strongly preferred over cyclopropanation of 1-hexene by a ratio of 9–19:1, whereas the **1-Rh**-catalyzed reactions were unselective with a slight preference for the cyclopropanation products. These results support the concept that the diruthenium catalysts have a greater selectivity preference for C–H functionalization over cyclopropanation, at least in the case of monosubstituted alkenes. The most likely reason for this effect is the different electrophilicity profiles of the two sets of catalysts.

**Table 5:**
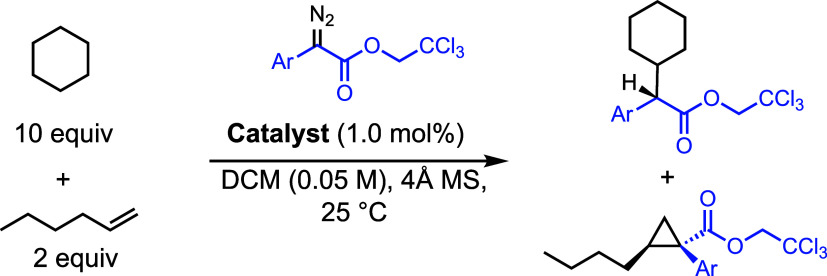

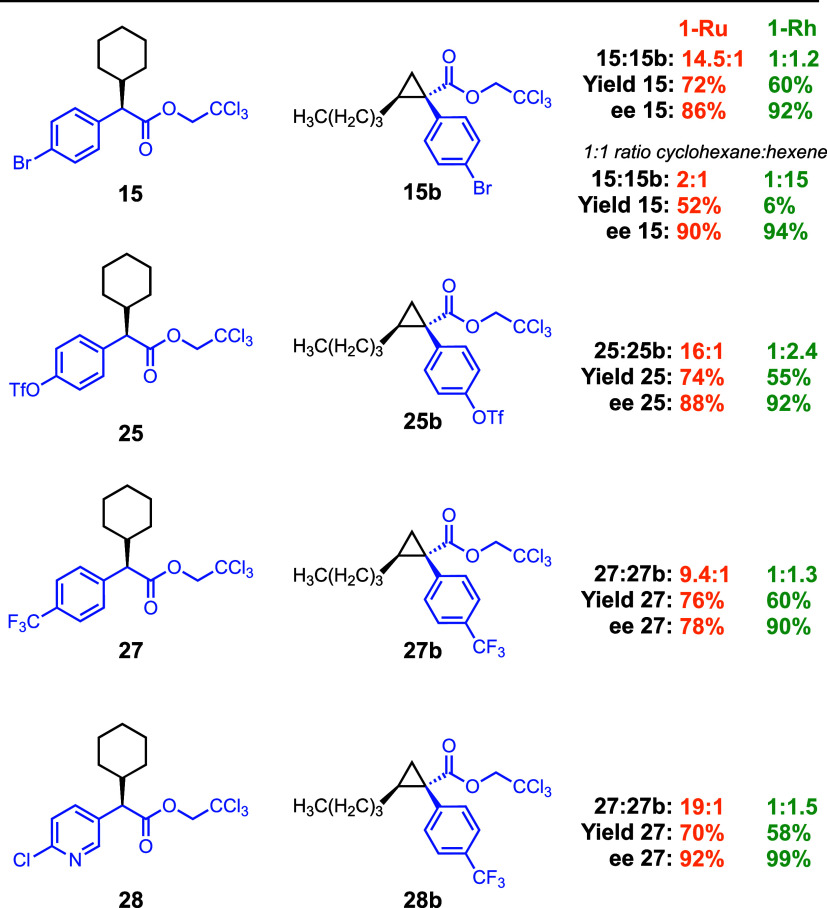
Competition between Cyclopopanation and C–H Functionalization^a^

a Diazo compound was added over a period of 2 h. Regioisomeric ratio was determined by crude NMR analysis.

## Conclusions

In summary, diruthenium tetracarboxylates are competent catalysts for intermolecular C–H functionalization with aryldiazoacetates. In the reaction with alkanes, the levels of enantioselectivity with C_4_-symmetric bowl-shaped catalysis are similar to the results obtained with the corresponding dirhodium catalysts. However, in the case of benzylic C–H functionalization, considerable variability is observed, including switching of the enantioselectivity between the two catalyst systems. The diruthenium catalysts do have distinctive reactivity profiles compared to the dirhodium catalysts. The diruthenium catalysts have a greater preference for C–H functionalization over cyclopropanation and they have a greater preference to react at the least crowded site. Of the diruthenium catalysts examined to date, the TPPTTL derivative **1-Ru** gives the highest levels of asymmetric induction for secondary C–H functionalization. The diruthenium catalysts operate well at a catalyst loading of 1 mol % with alkanes and cycloalkanes, but heteroatoms and even aromatic rings can interfere with the catalytic efficiency resulting in the need for higher catalyst loading or reactions temperatures. These studies show that even though the diruthenium catalysts are generally effective catalysts for enantioselective intermolecular C–H functionalization with donor/acceptor carbenes, further optimization would be needed for them to be truly competitive with the dirhodium catalysts in terms of functional group compatibility, turnover frequency, and turnover numbers.

## Supporting Information Available

Complete experimental procedures and compound characterization (PDF)
