# Supplementary material for: C4-Symmetric Bowl-Shaped Diruthenium Tetracarboxylate Catalysts for Enantioselective C–H Functionalization Using Donor/Acceptor Carbenes
Source: ACS Catal. 2025 Mar 27;15(8):5906–14. doi: 10.1021/acscatal.5c01052 (PMC12012802; doi:10.1021/acscatal.5c01052)
Supplement: Supplementary file 1 — cs5c01052_si_001.pdf [file cs5c01052_si_001.pdf]

Supporting Information

C_4-Symmetric Bowl-Shaped Diruthenium Tetracarboxylate Catalysts for Enantioselective C–H Functionalization Using Donor/Acceptor Carbenes

Joshua K. Sailer, John Bacsa and Huw M. L. Davies*

Department of Chemistry, Emory University, 1515 Dickey Drive, Atlanta, Georgia 30322, United States

*E-Mail: hmdavie@emory.edu

Table of Contents

General Considerations **2**

Preparation of Known Compounds **2**

Catalyst Synthesis **3**

Regioselectivity Determination **5**

C–H Insertion Reactions **47**

HPLC Traces **57**

Crystallographic Data **97**

NMR Spectrum for Novel Compounds **124**

References **131**

CAUTION: Diazo compounds are high energy compounds and need to be treated with respect. Even though we experienced no energetic decomposition in this work, care should be taken in handling large quantities of diazo compounds. Large scale reactions should be conducted behind a blast shield. For a more complete analysis of the risks associated with diazo compounds see the recent review by Bull et. al.[1]

General Considerations

All experiments were carried out in flame-dried glassware under argon atmosphere unless otherwise stated. Flash column chromatography was performed on silica gel. Unless otherwise noted, all other reagents were obtained from commercial sources (Sigma Aldrich, Fisher, TCI Chemicals, AK Scientific, Combi Blocks, Oakwood Chemicals, Ambeed) and used as received without purification. ^{1}H, ^{13}C, and ^{19}F NMR spectra were recorded at either 400 MHz (^{13}C at 100 MHz) on Bruker 400 spectrometer or 600 MHz (^{13}C at 151 MHz) on INOVA 600 or Bruker 600 spectrometer. NMR spectra were run in solutions of deuterated chloroform ($CDCl_3$) with residual chloroform taken as an internal standard (7.26 ppm for ^{1}H, and 77.16 ppm for ^{13}C), and were reported in parts per million (ppm). The abbreviations for multiplicity are as follows: s = singlet, d = doublet, t = triplet, q = quartet, p = pentet, m = multiplet, dd = doublet of doublet, etc. Coupling constants (J values) are obtained from the spectra. Thin layer chromatography was performed on aluminum-back silica gel plates with UV light and cerium aluminum molybdate (CAM) stain to visualize. Mass spectra were taken on a Thermo Finnigan LTQ-FTMS spectrometer with APCI or ESI. IR spectra were collected on a Nicolet iS10 FT-IR spectrometer from Thermo Scientific and reported in unit of cm-1. Enantiomeric excess (% ee) data were obtained on an Agilent 1100 HPLC or an Agilent 1290 Infinity UHPLC, eluting the purified products using a mixed solution of HPLC-grade 2-propanol (i-PrOH) and n-hexane.

Preparation of Known Compounds

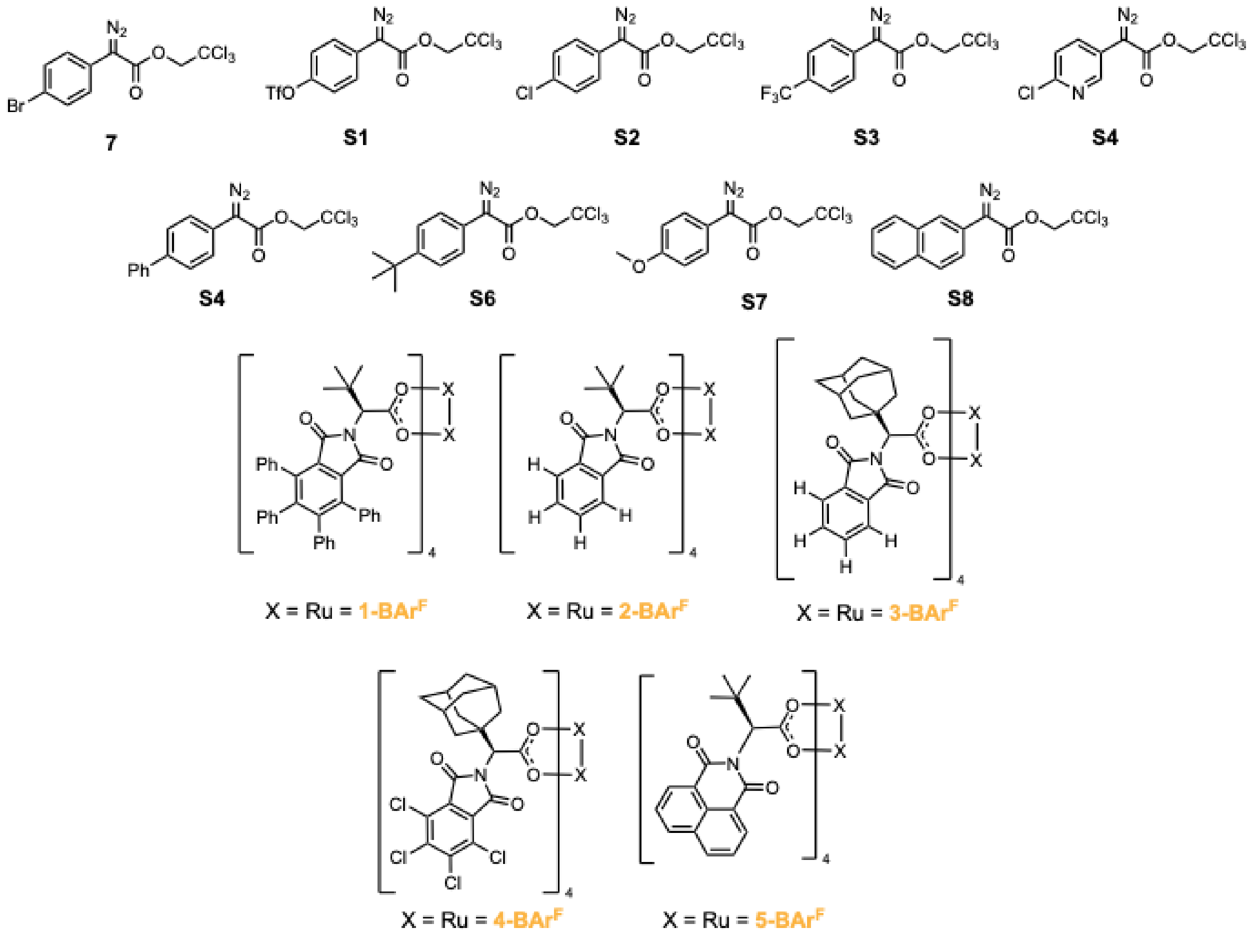

Figure S1: Known compounds synthesized.

Diazo Compounds **7** and **S1-S8** were prepared according to the established literature and matched the reported spectra.[2]
1-Ru-5-Ru were prepared according to the established literature and matched the reported spectra.[3]

Catalyst Synthesis

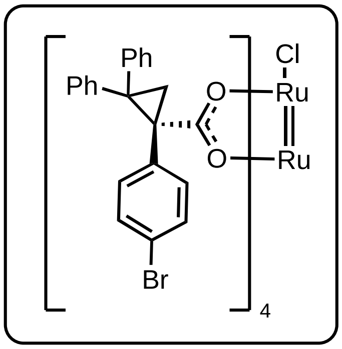

Ru_2(*S*-*p*Br-TPCP)$_4$Cl (6-Ru)
To a 25 mL RBF equipped with a stir bar was added 1-(4-bromophenyl)-2,2-diphenylcyclopropane-1-carboxylic acid (205 mg, 8 equiv, 521 µmol) and $Ru_2(OAc)_4Cl$ (50.0 mg, 1 equiv, 65.2 µmol). The solids were subsequently dissolved in *tert*-butylacetate (12.5 mL) and the RBF was fitted to a Soxlhet extractor fitted with K_2CO_3 and a small layer of sand. The reaction was heated to a vigorous reflux (~122 °C) and left for 18 h. After this time the reaction was cooled, and the crude material was concentrated and loaded onto silica. The material was purified using column chromatography (1% MeOH/DCM). The brown fractions were collected and recrystallized

from chloroform and hexanes (1:3 ratio) to afford brown needle-like crystals which were collected to afford the title compound (30.1 mg, 16%).
NMR data are unavailable for this compound due to its paramagnetic character. The key data for the structural characterization were obtained by HRMS and X-ray crystallography.
HRMS (+p ESI): Calcd for $C_{88}H_{64}O_8{}^{79}Br_2{}^{81}Br_2{}^{101}Ru^{102}Ru$ [M–Cl] 1770.9388 found 1770.9466

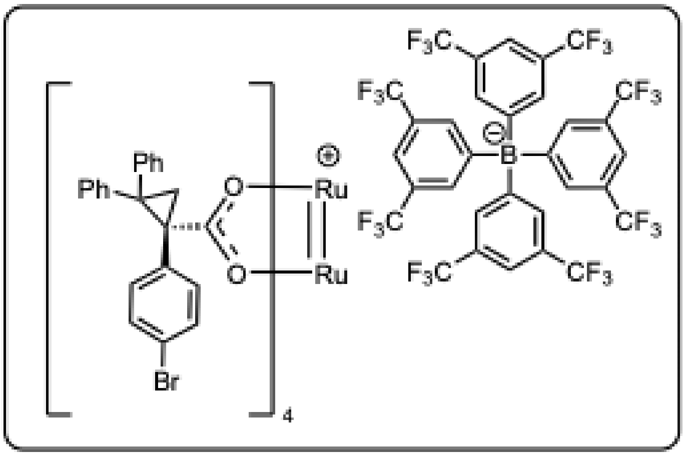

Ru2(*S*-*p*Br-TPCP)4Cl (6-Ru)
To a 4 mL vial equipped with a stir bar was added **7-Ru** (10 mg, 5.6 µmol, 1 equiv) which was subsequently dissolved in 1 mL of DCM. Then, NaBAr^F (5.3 mg, 5.9 µmol, 1.05 equiv) was added in one portion and the reaction was left to stir overnight. After 16 h, the solution was passed over a small pad of silica and concentrated down to afford an orange/brown powder (14.3 mg, 96%). NMR data are unavailable for this compound due to its paramagnetic character. The key data for the structural characterization were obtained by HRMS.
HRMS (+p ESI): Calcd for $C_{88}H_{64}O_8{}^{79}Br_2{}^{81}Br_2{}^{101}Ru^{102}Ru$ [M–Cl] 1770.9388 found 1770.9408.
HRMS (-p ESI): Calcd for $C_{32}H_{12}{}^{10}B^{-}F_{24}$ [M$^-$] 862.0691, found 862.0693.

Regioselectivity Determination

Regioselectivity determination for reactions with *p*-Cymene

Regioselectivity was determined through the integration of the TCE peaks comparing the 1° and 3° insertion. One of the 3° insertion TCE peak is located at 4.47 ppm and one of the 1° insertion TCE peaks is located at 4.71 ppm.

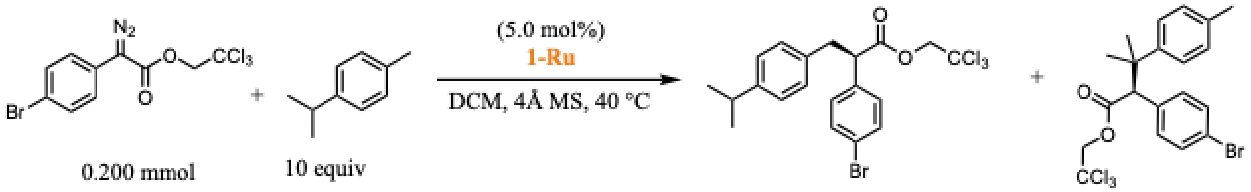

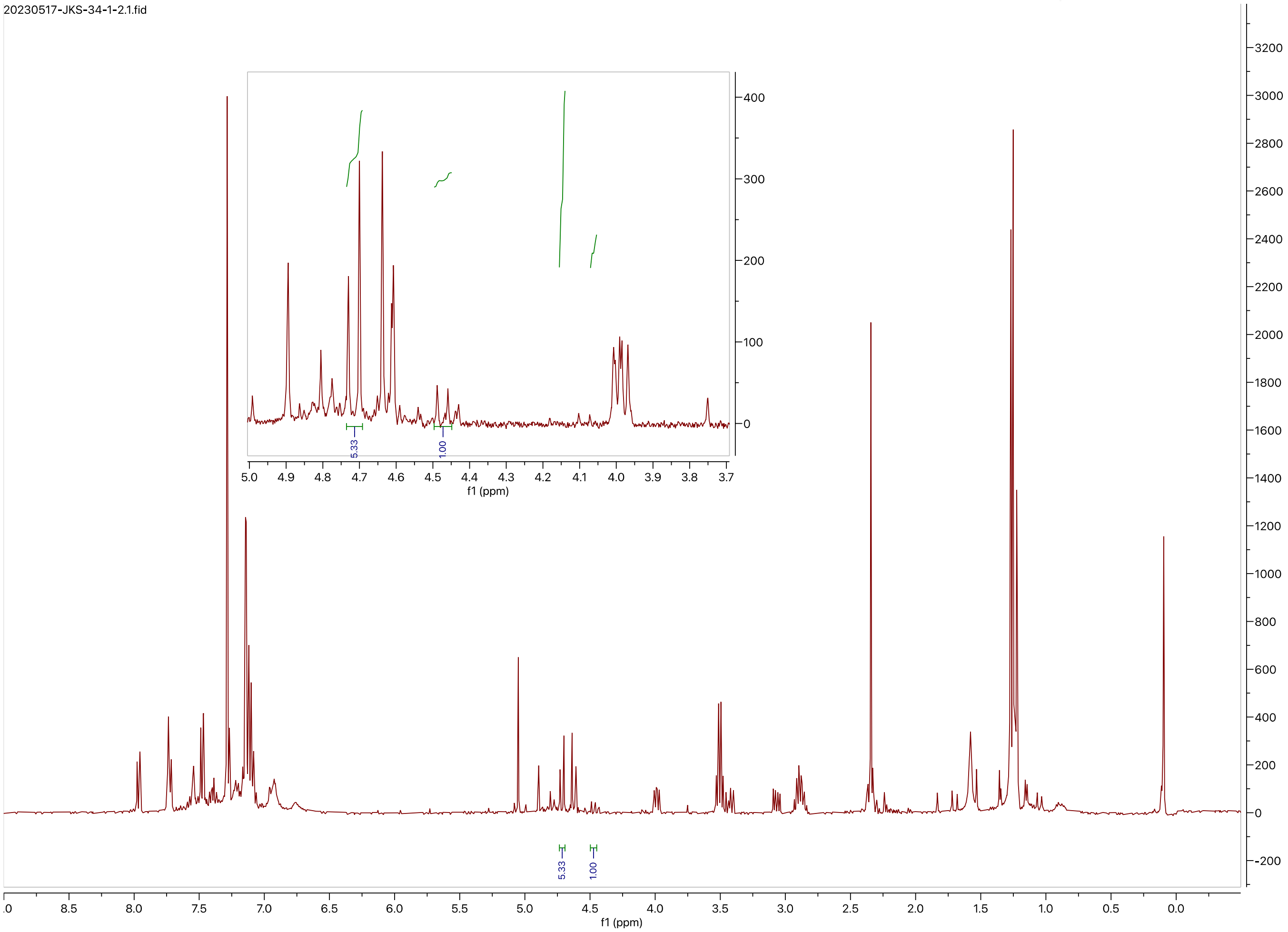

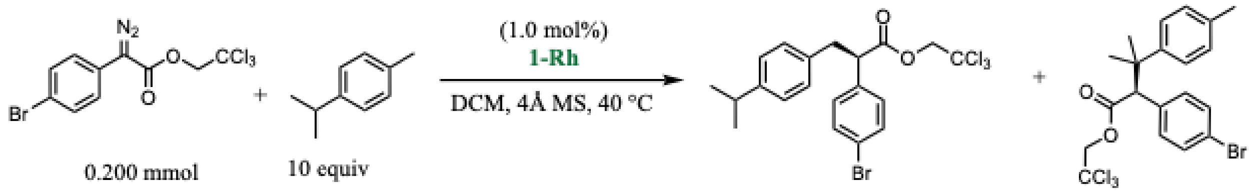
N2
Br
O
CCl3
0.200 mmol
10 equiv
(1.0 mol%)
1-Rh
DCM, 4Å MS, 40 °C
Br
CCl3

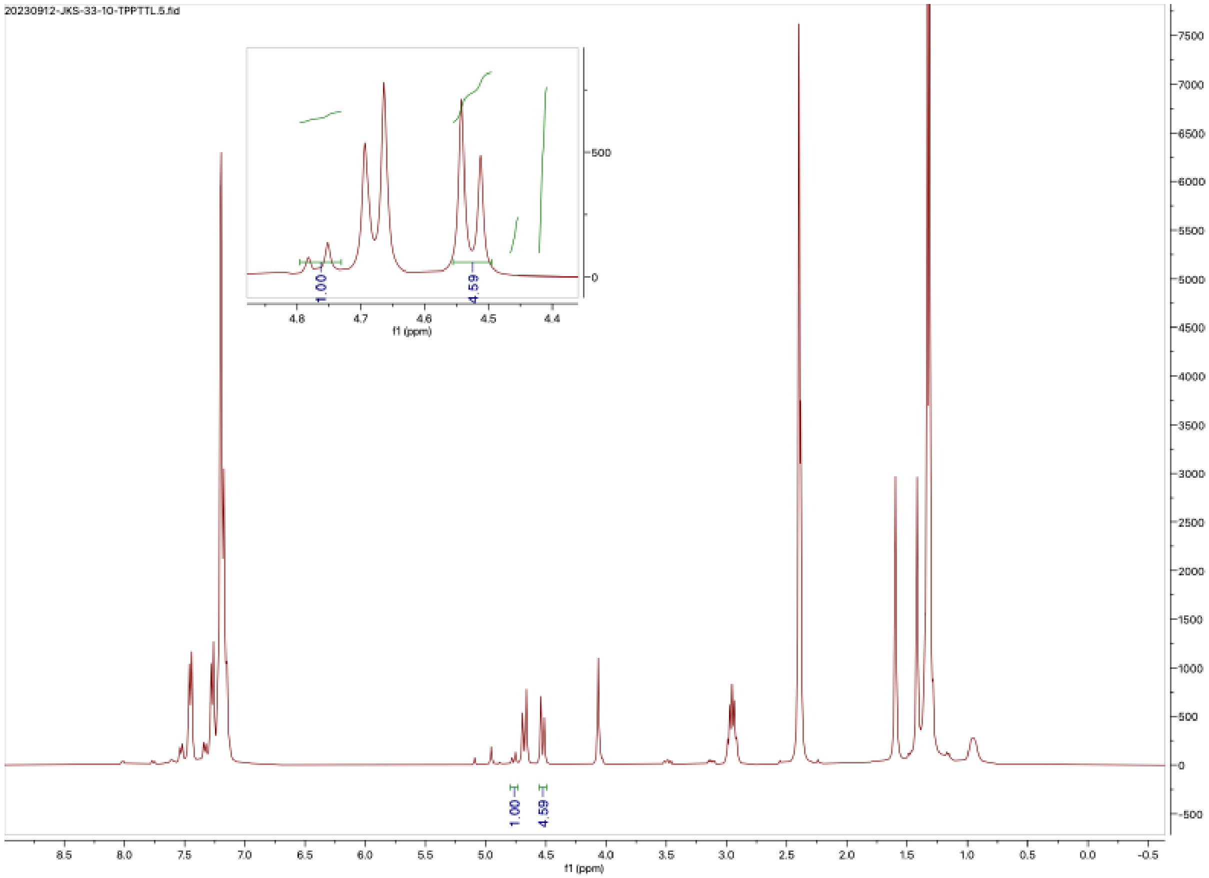
20230912-JKS-33-10-TPPTTL.5.fid
1.00
4.59
f1 (ppm)

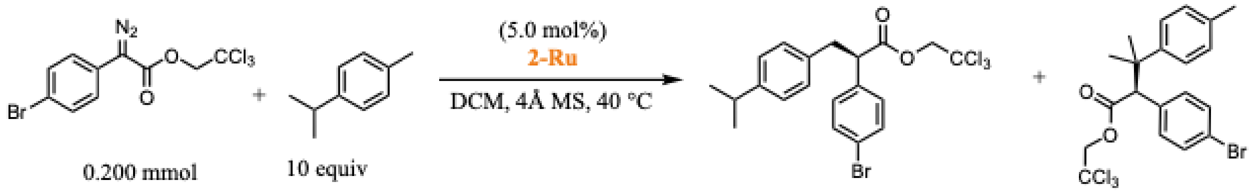
N2
CCl3
Br
O
(5.0 mol%)
2-Ru
DCM, 4Å MS, 40 °C
0.200 mmol
10 equiv

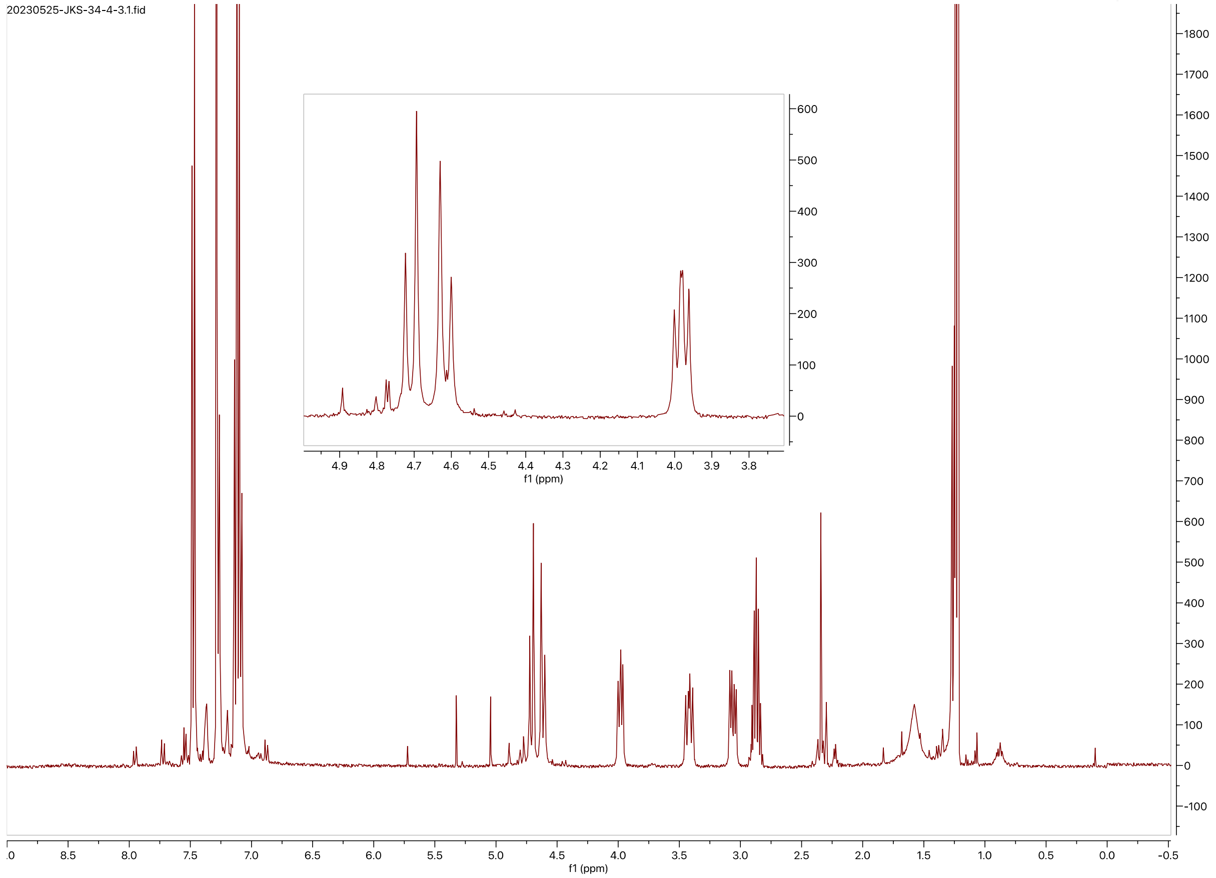
20230525-JKS-34-4-3.1.fid
f1 (ppm)

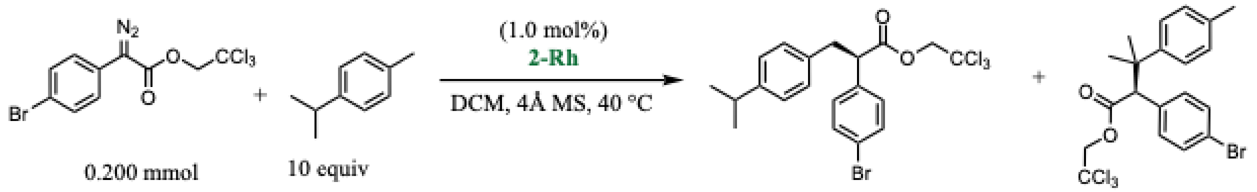
N2
O
CCl3
Br
O
+
(1.0 mol%)
2-Rh
DCM, 4Å MS, 40 °C
CCl3
Br
+
Br
CCl3
0.200 mmol
10 equiv

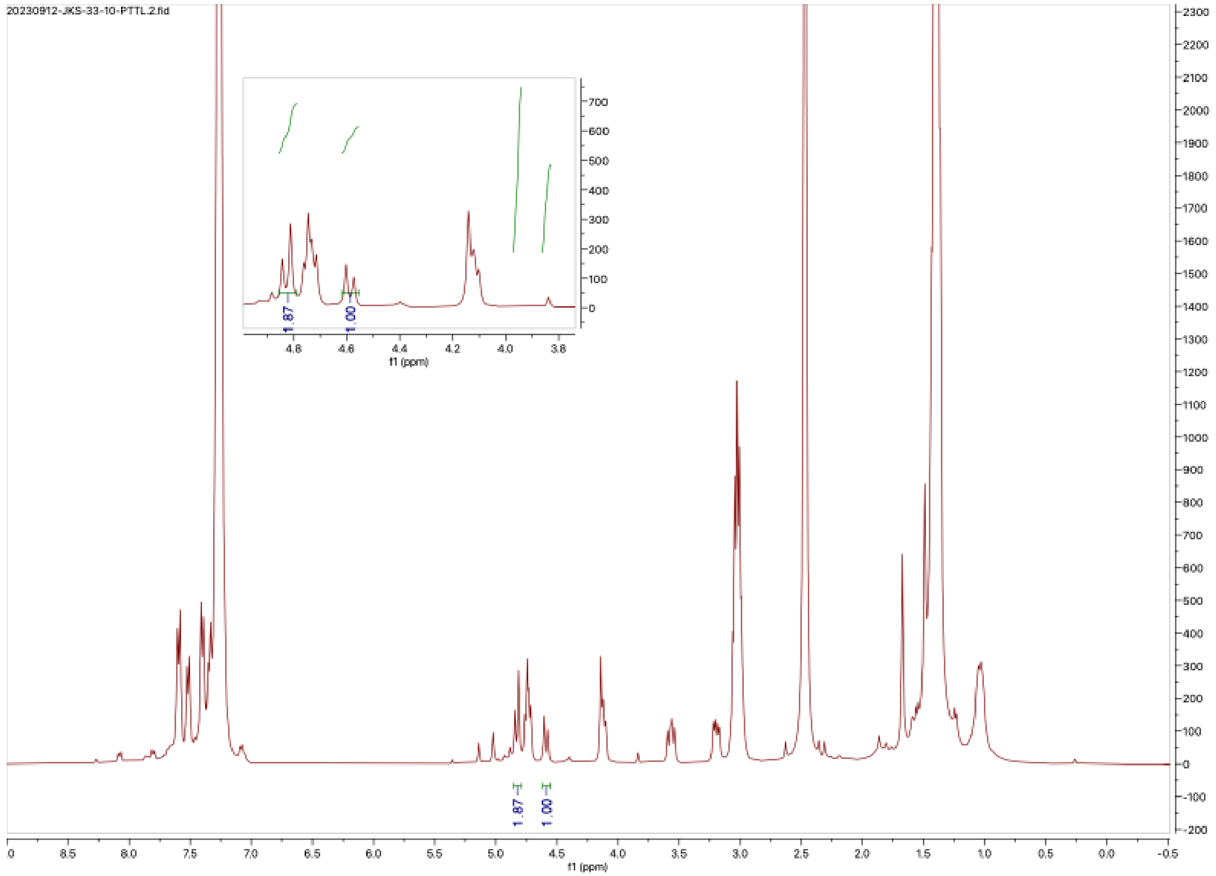
20230912-JKS-33-10-PTTL.2.fid
1.87
1.00
f1 (ppm)

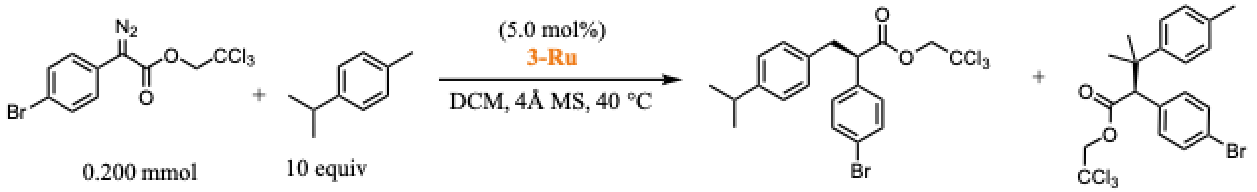
N2
CCl3
Br
0.200 mmol
10 equiv
(5.0 mol%)
3-Ru
DCM, 4Å MS, 40 °C
CCl3
Br
Br
CCl3

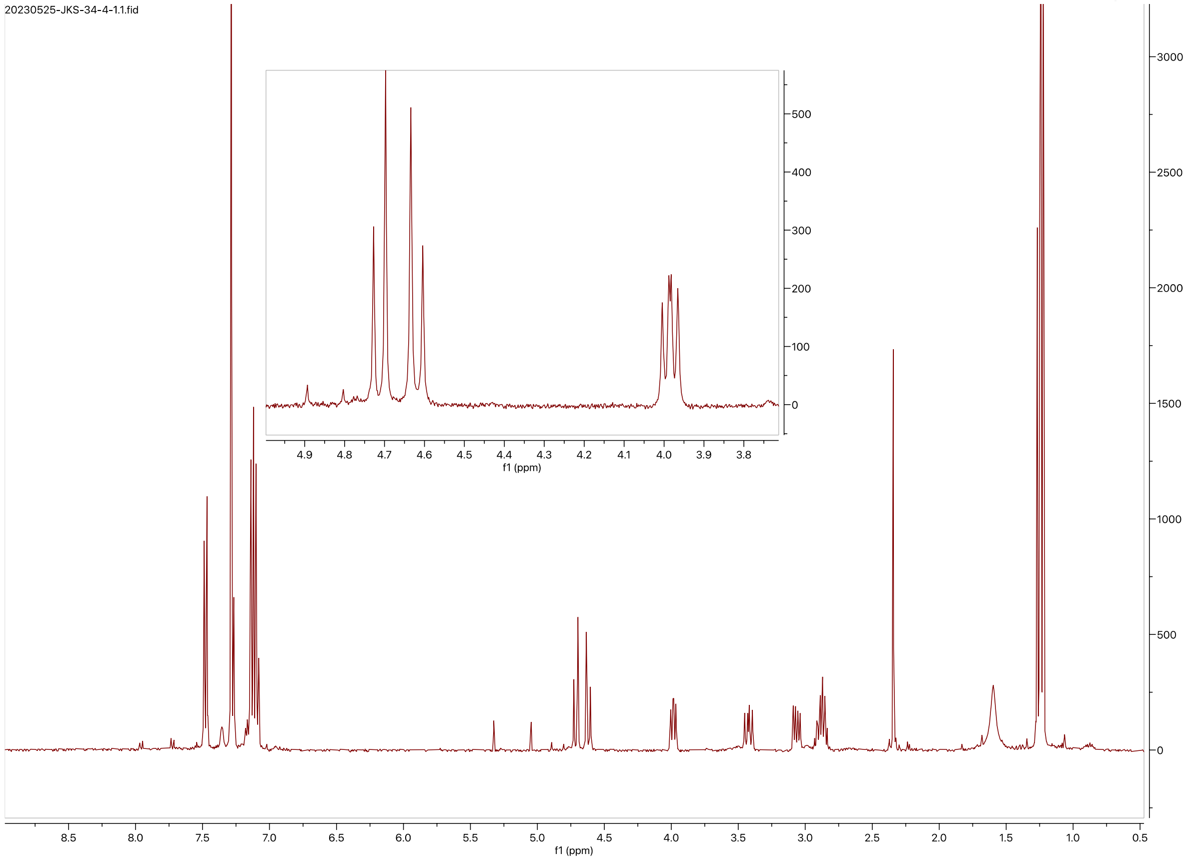
20230525-JKS-34-4-1.1.fid
f1 (ppm)
f1 (ppm)

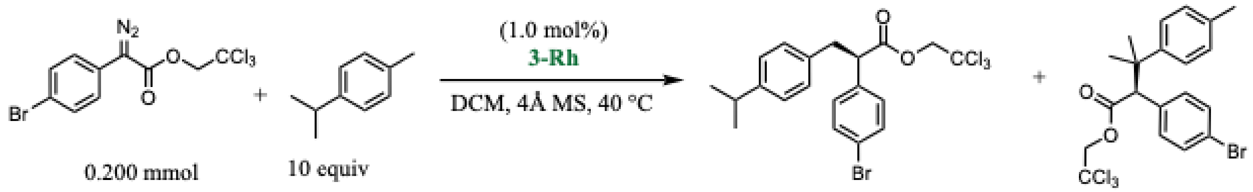
(1.0 mol%)
3-Rh
DCM, 4Å MS, 40 °C
0.200 mmol
10 equiv

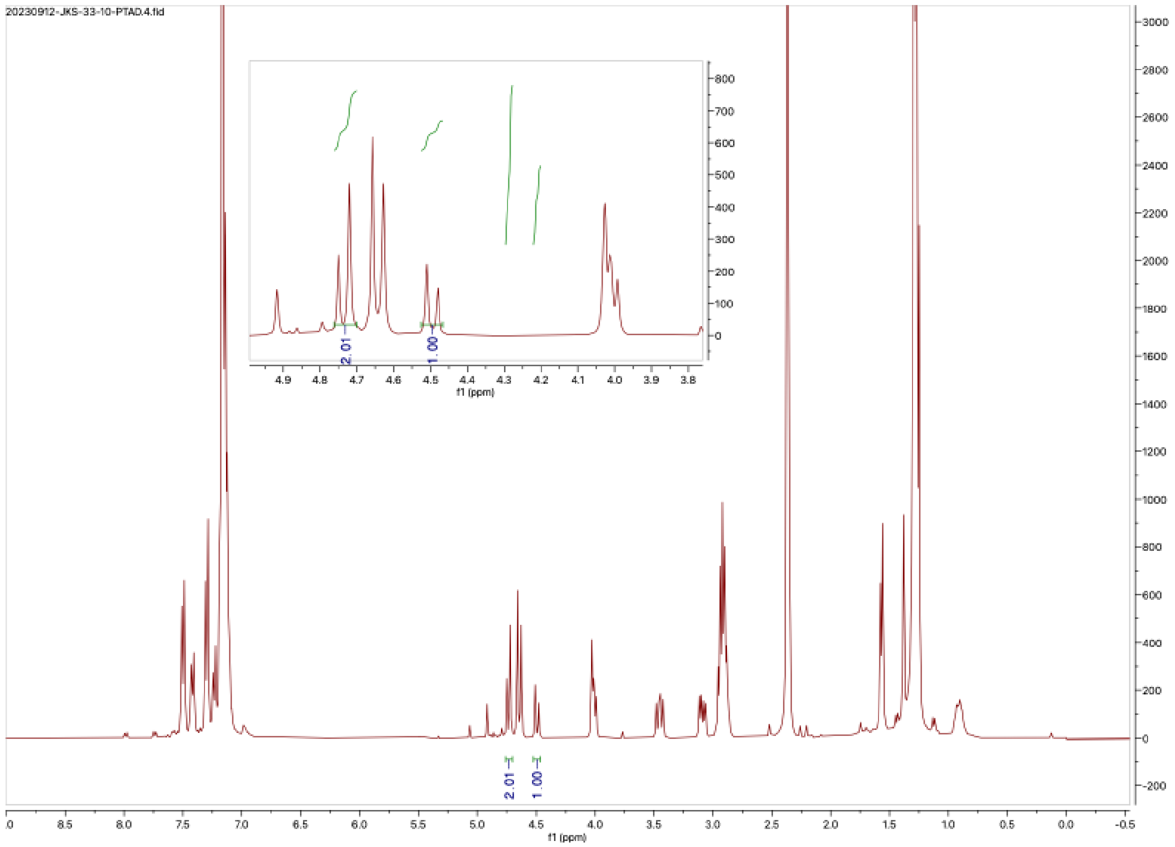
20230912-JKS-33-10-PTAD.4.fid
f1 (ppm)

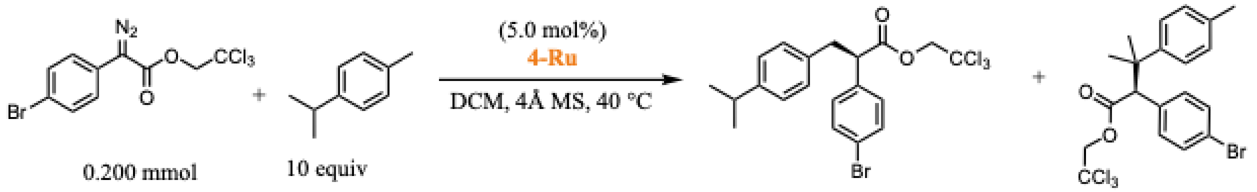
(5.0 mol%)
4-Ru
DCM, 4Å MS, 40 °C
0.200 mmol
10 equiv

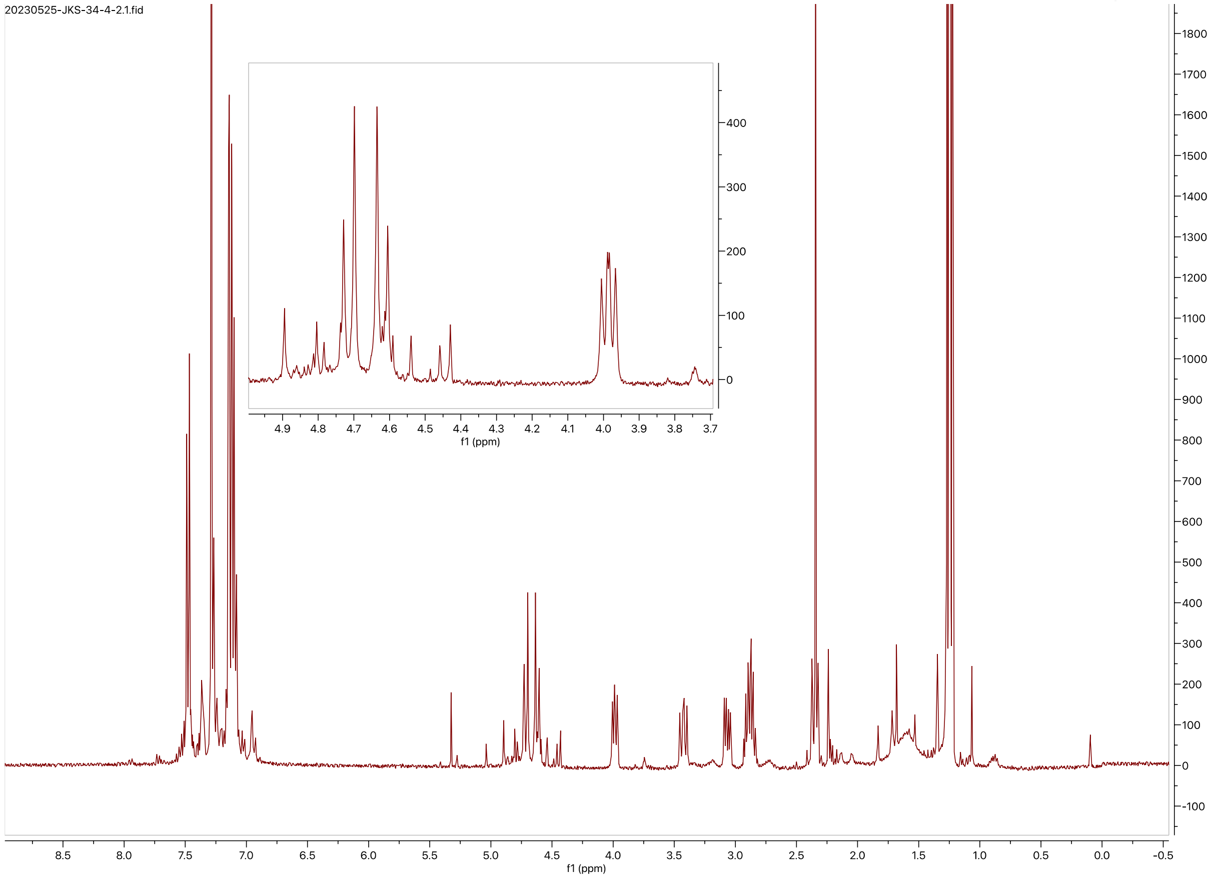
20230525-JKS-34-4-2.1.fid
f1 (ppm)

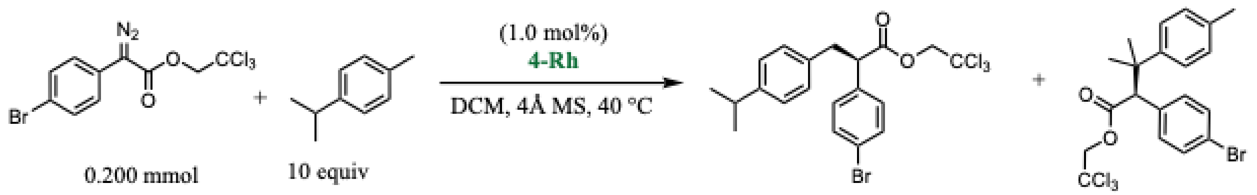
Br
N2
O
CCl3
0.200 mmol
10 equiv
(1.0 mol%)
4-Rh
DCM, 4Å MS, 40 °C
Br
CCl3

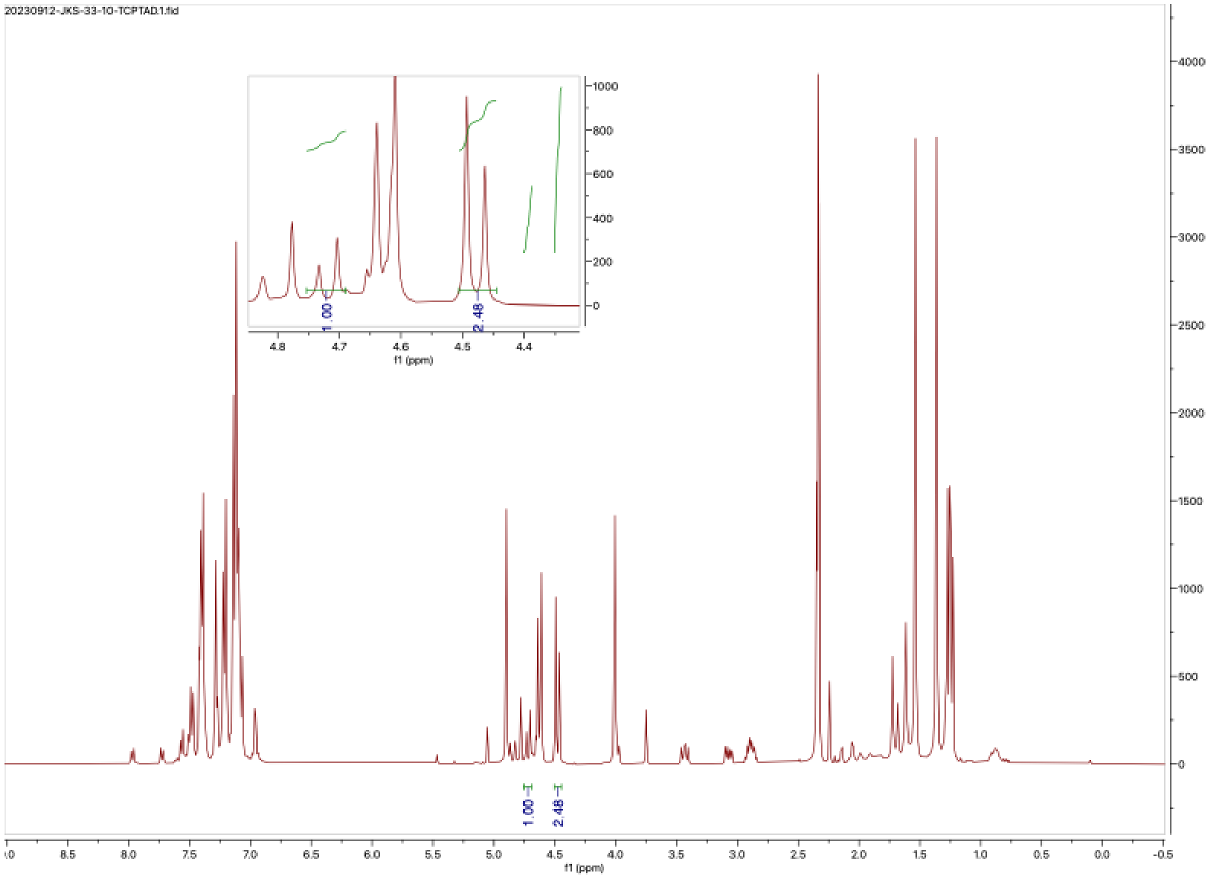
20230912-JKS-33-10-TCPTAD.1.fid
1.00
2.48
f1 (ppm)

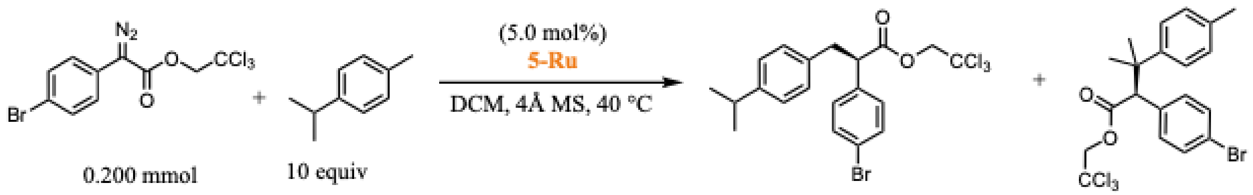

N2
O
CCl3
Br
O
+
(5.0 mol%)
5-Ru
DCM, 4Å MS, 40 °C
+
0.200 mmol
10 equiv

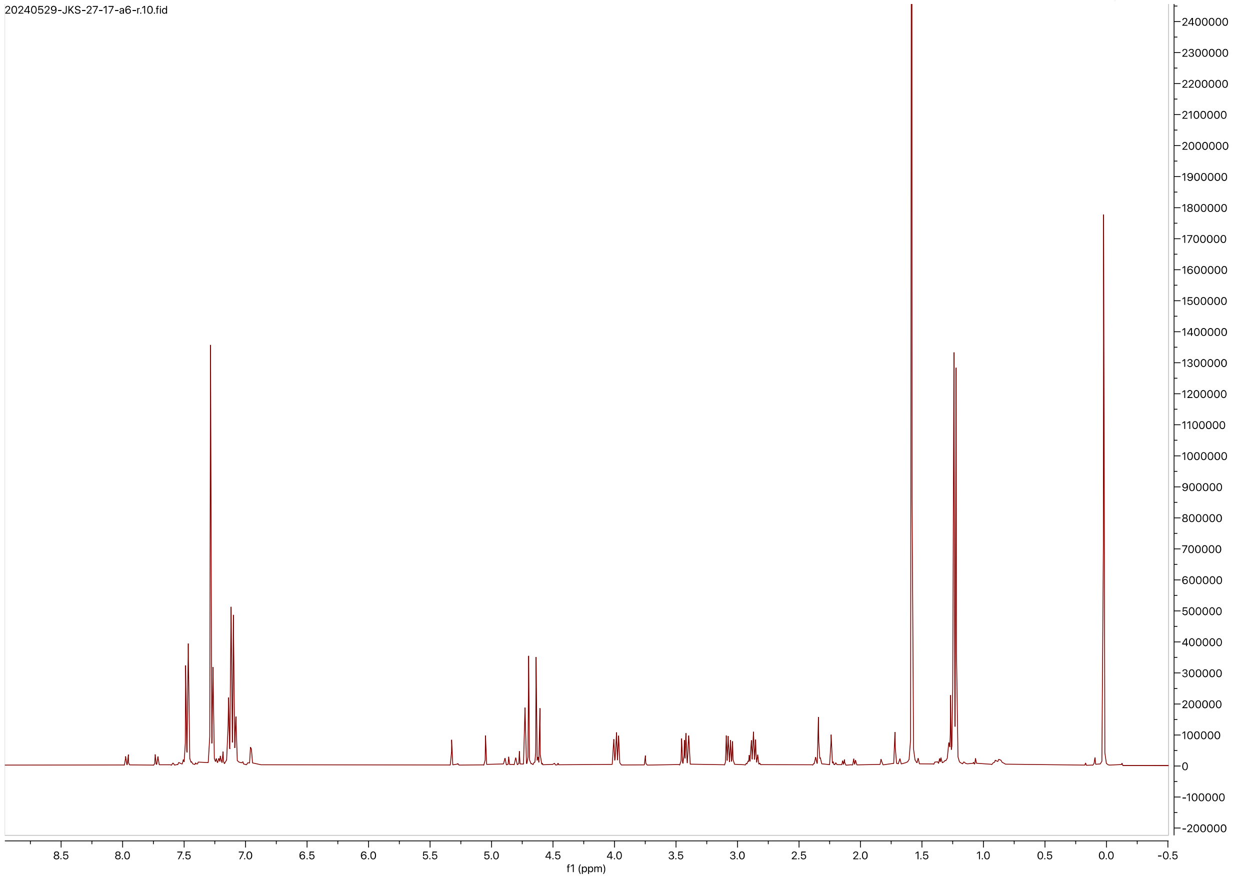

20240529-JKS-27-17-a6-r.10.fid
f1 (ppm)

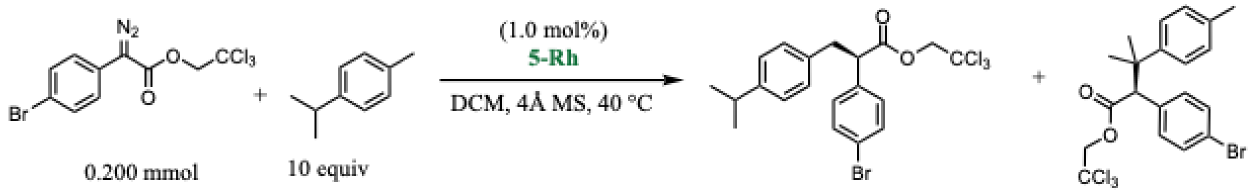

N2
CCl3
Br
0.200 mmol
10 equiv
(1.0 mol%)
5-Rh
DCM, 4Å MS, 40 °C

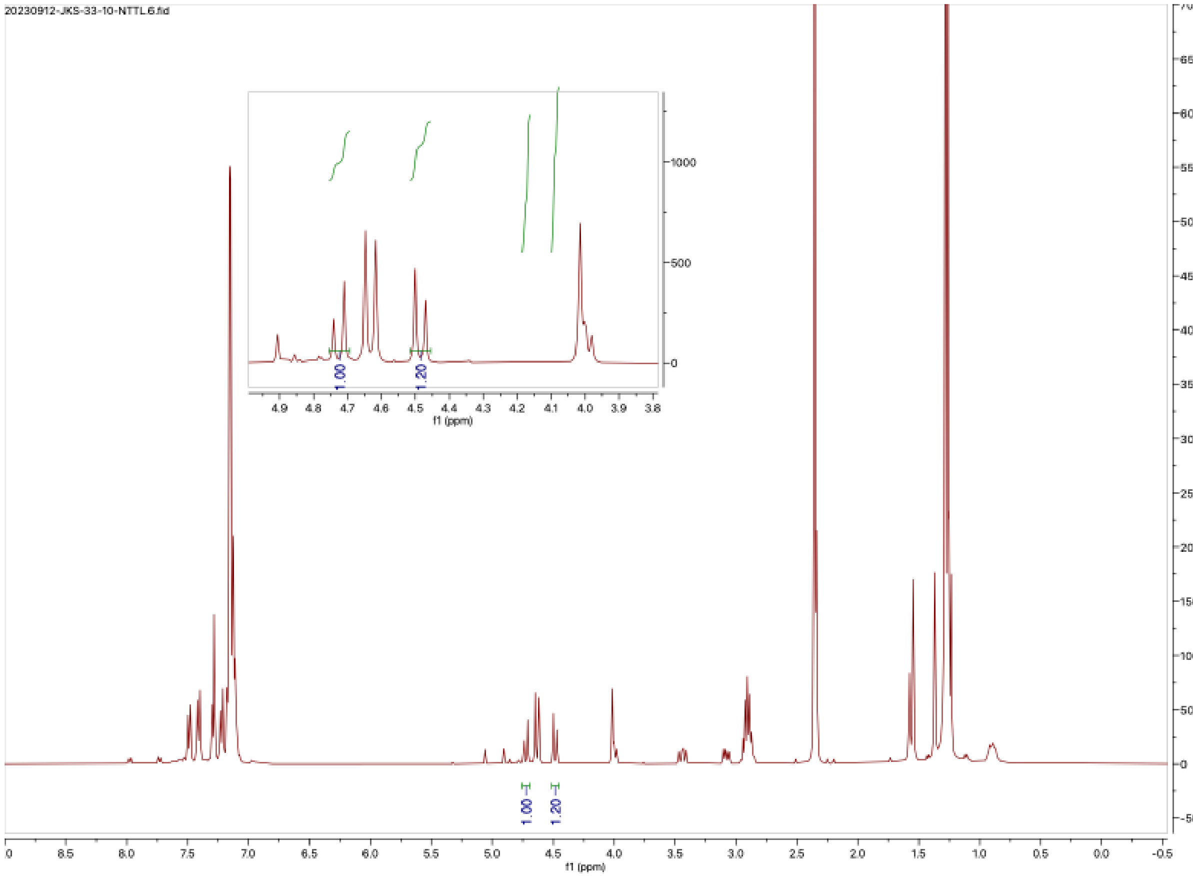

20230912-JKS-33-10-NTTL.6.fid
1.00
1.20
f1 (ppm)

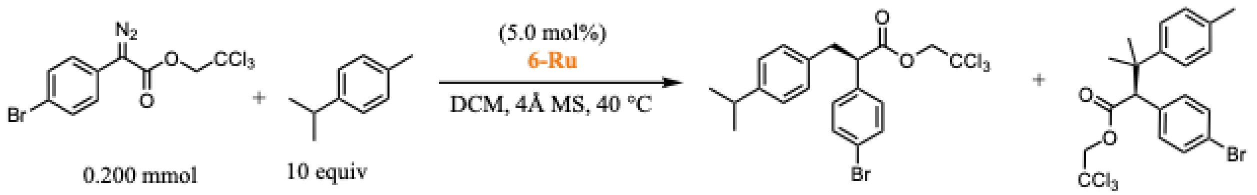
(5.0 mol%)
6-Ru
DCM, 4Å MS, 40 °C
0.200 mmol
10 equiv

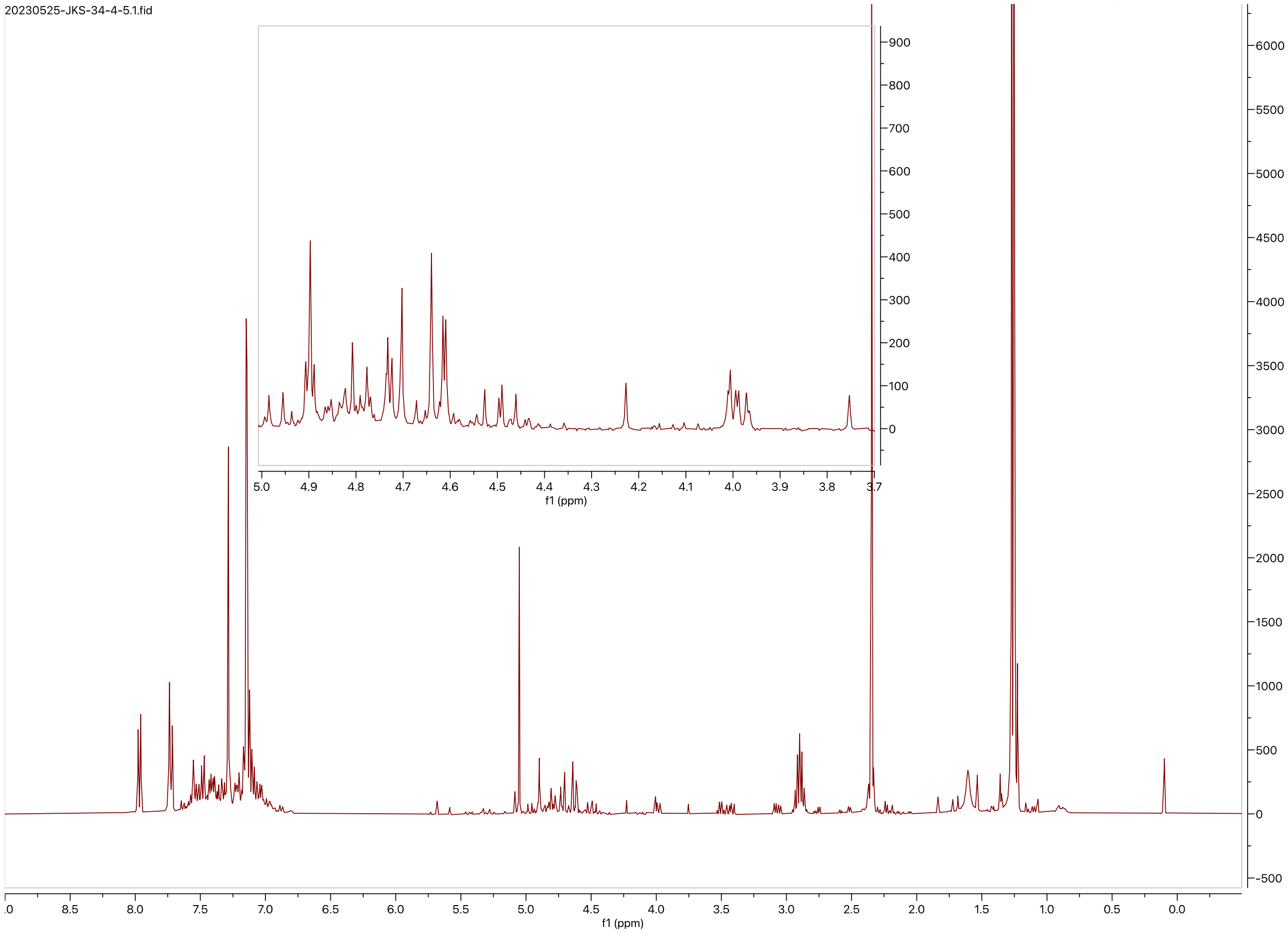
20230525-JKS-34-4-5.1.fid
f1 (ppm)

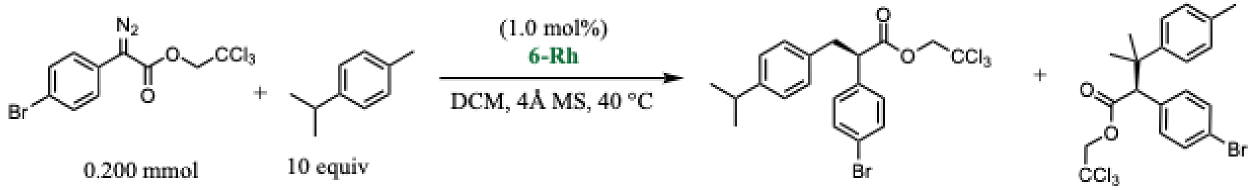

(1.0 mol%)
6-Rh
DCM, 4Å MS, 40 °C
0.200 mmol
10 equiv

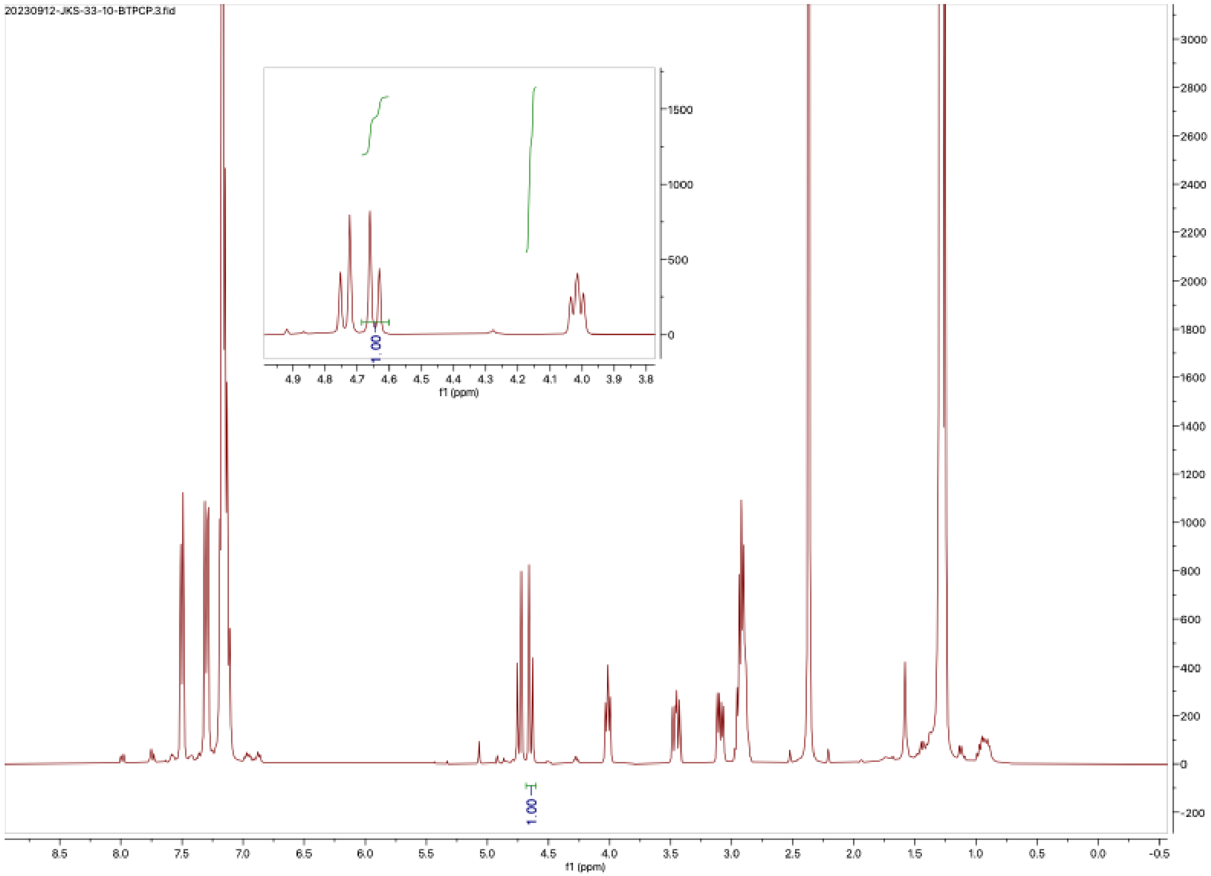

20230912-JKS-33-10-BTPCP.3.fid
f1 (ppm)

Regioselectivity and diastereoselectivity determination for reactions with 4-isopropylethylbenzene (11)

Regioselectivity was determined through the integration of the benzylic alpha-carbonyl hydrogen comparing the 2° and 3° insertion. The 3° insertion peak is located at 3.98 ppm and one of the 2° insertion peak is located at 3.81 ppm. The diastereoselectivity is determined through comparing the methyl peak on the ethyl group. The minor diastereomer is cis with the bromo-substituted phenyl ring, shielding farther up-field.

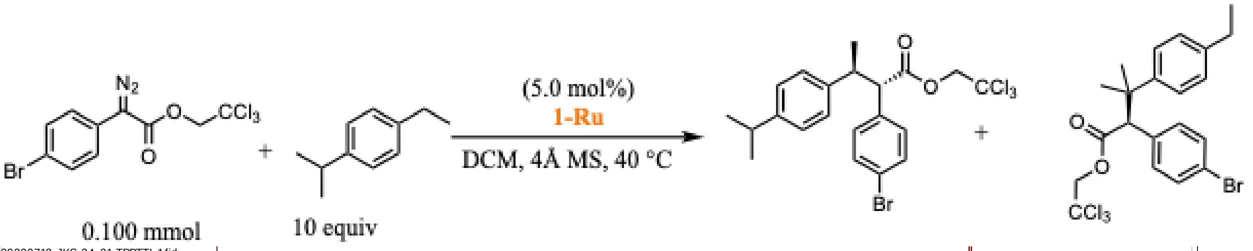

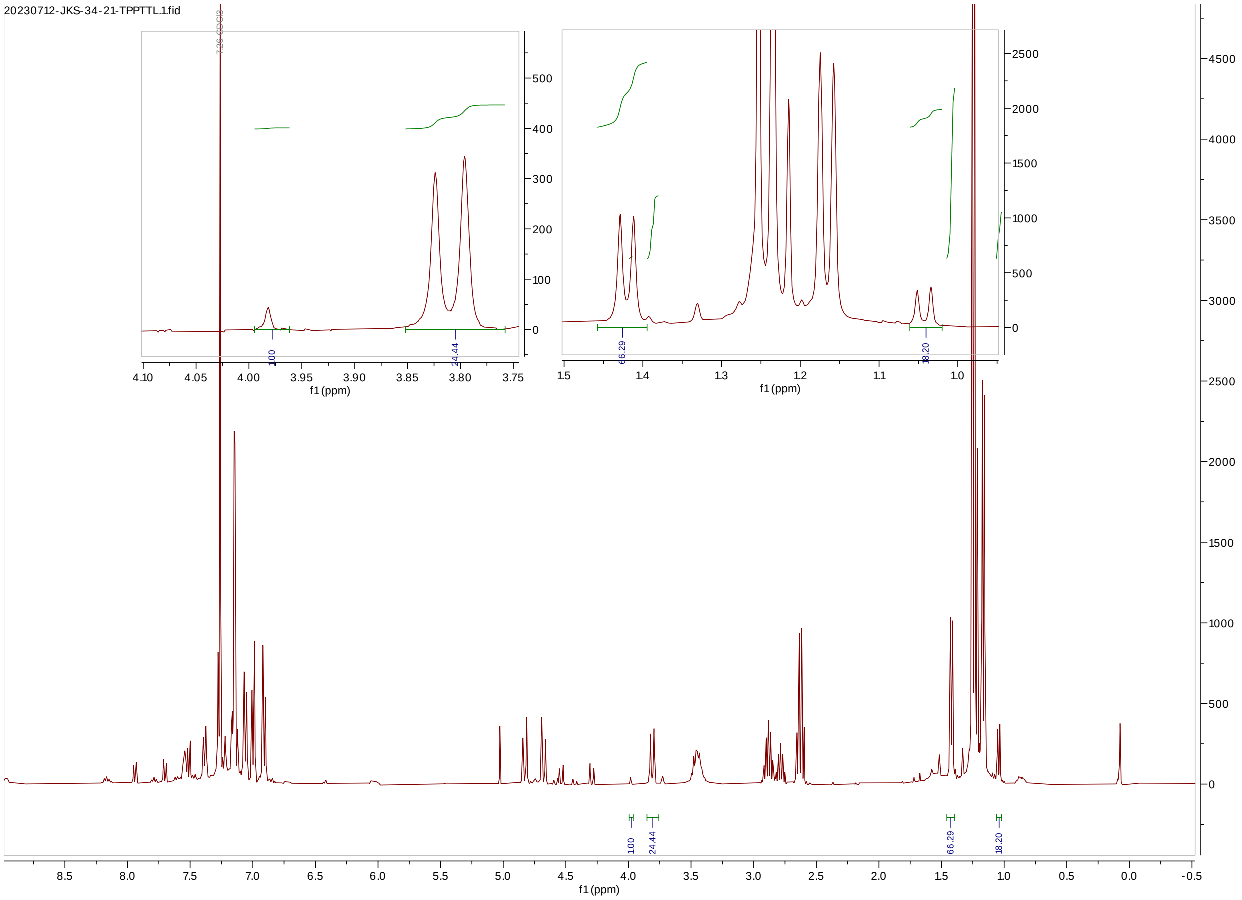

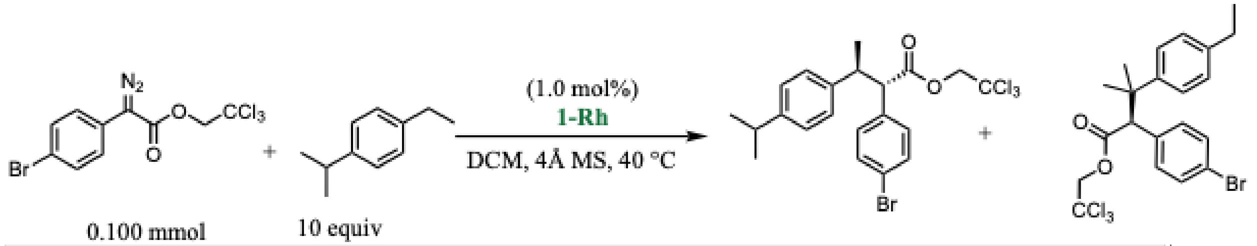

Br
N2
O
CCl3
0.100 mmol
10 equiv
(1.0 mol%)
1-Rh
DCM, 4Å MS, 40 °C

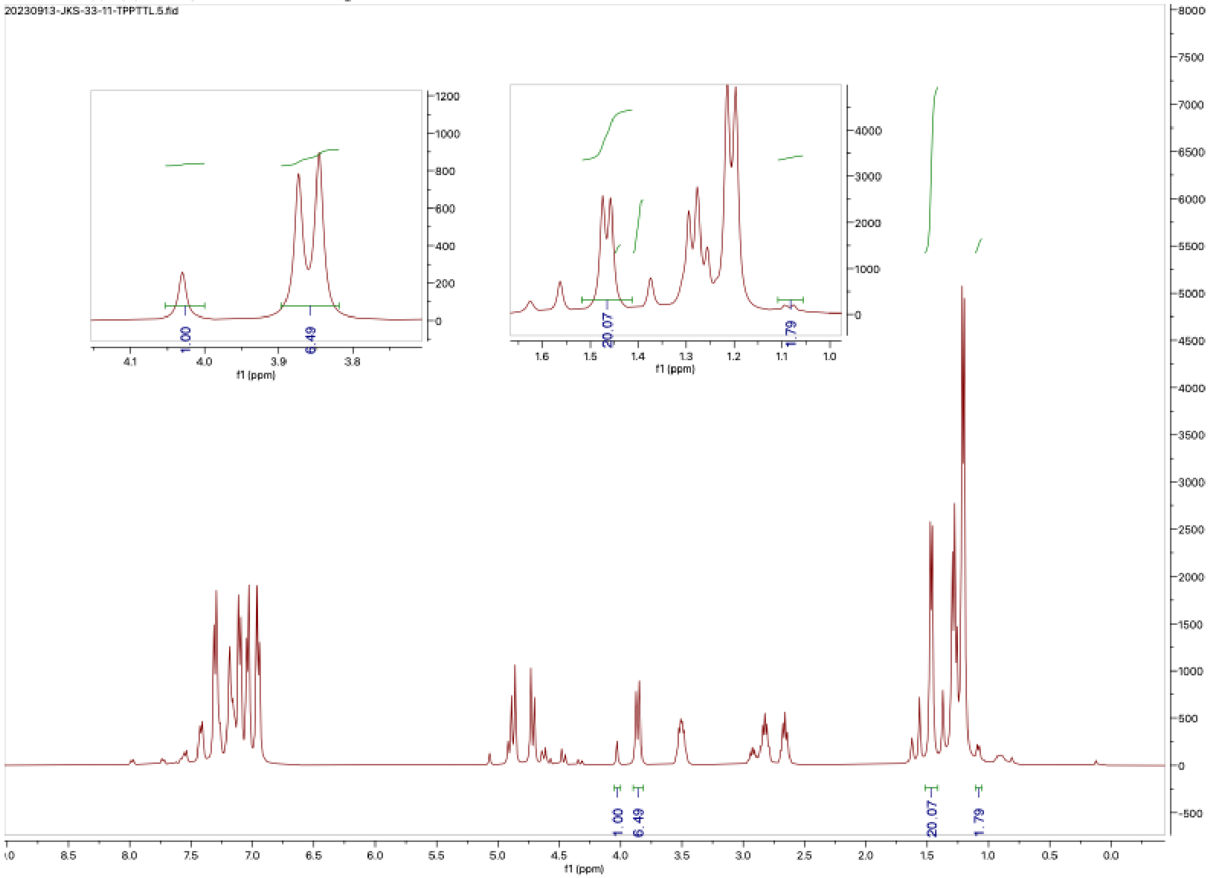

20230913-JKS-33-11-TPPTTL.5.fid
1.00
6.49
20.07
1.79
f1 (ppm)

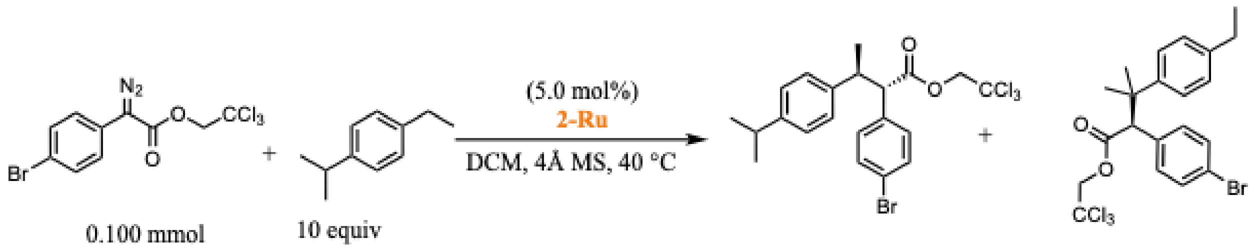
(5.0 mol%)
2-Ru
DCM, 4Å MS, 40 °C
0.100 mmol
10 equiv

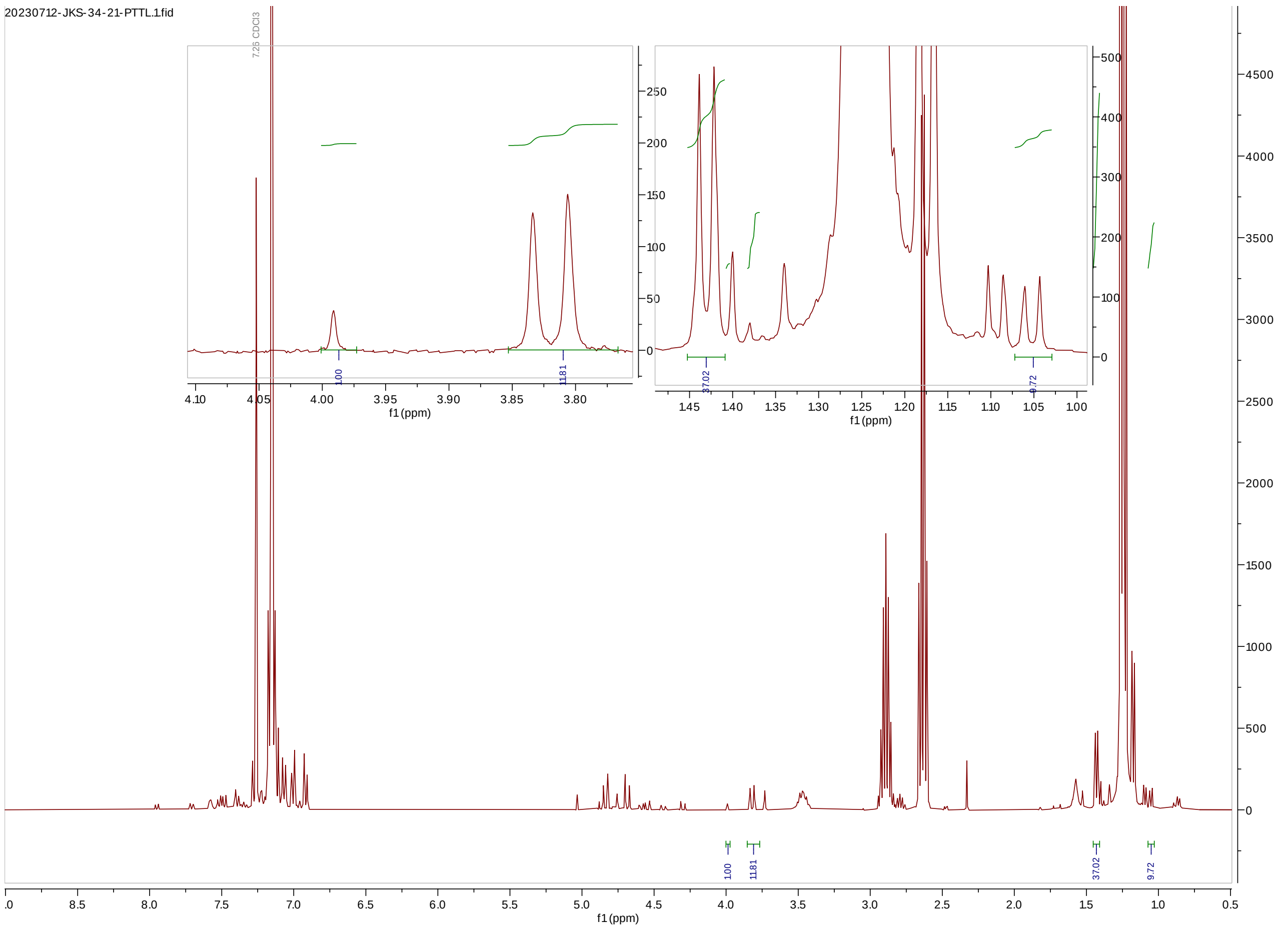
20230712-JKS-34-21-PTTL.1.fid
f1 (ppm)

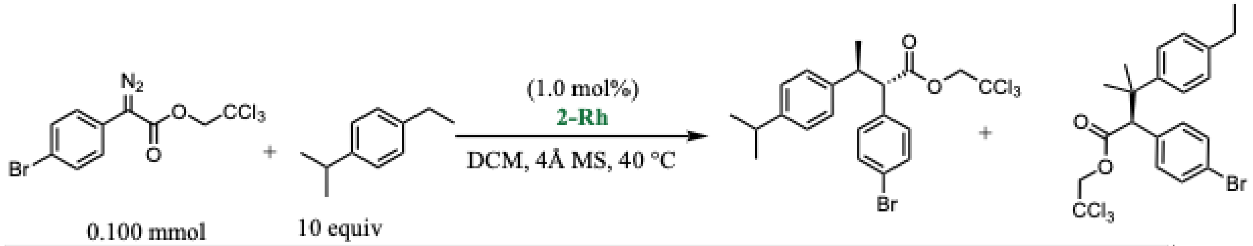

(1.0 mol%)
2-Rh
DCM, 4Å MS, 40 °C
0.100 mmol
10 equiv

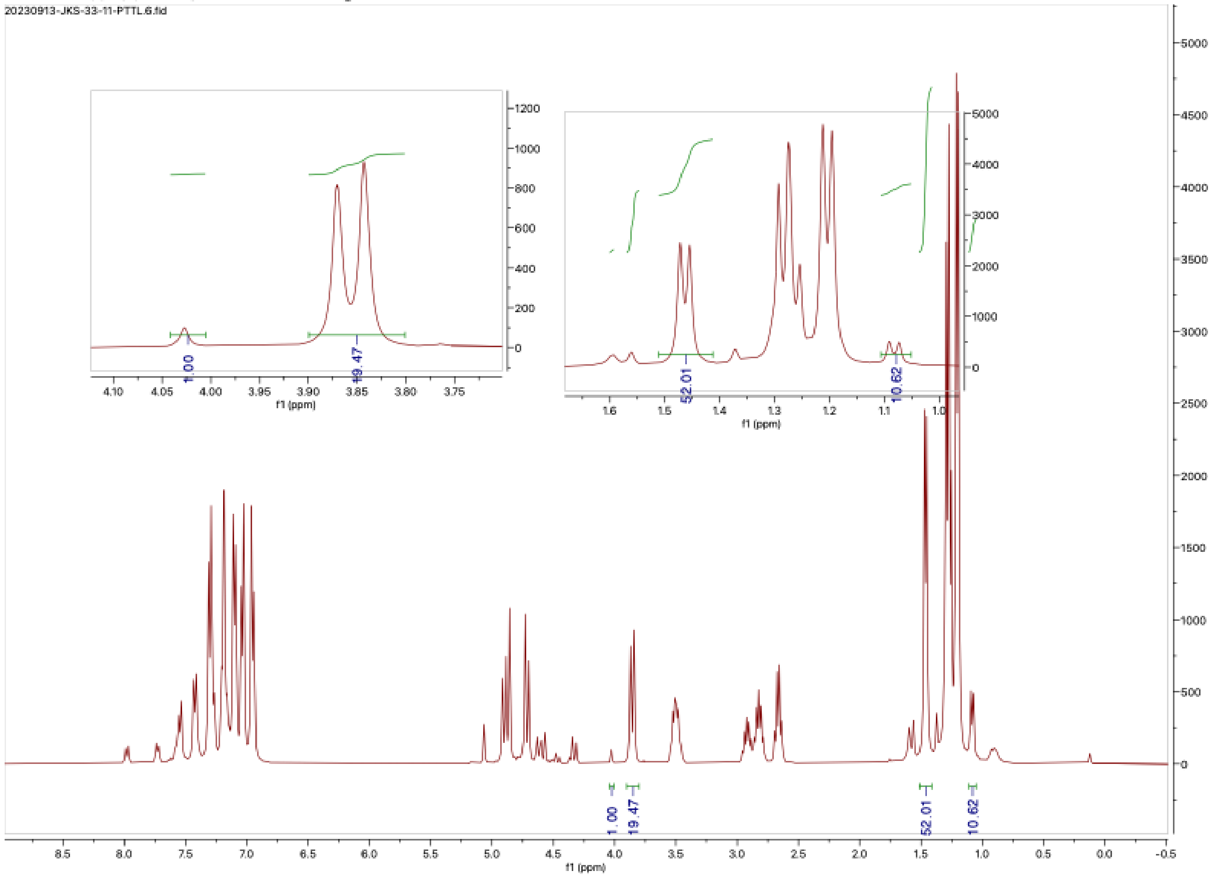

20230913-JKS-33-11-PTTL.6.fid
f1 (ppm)

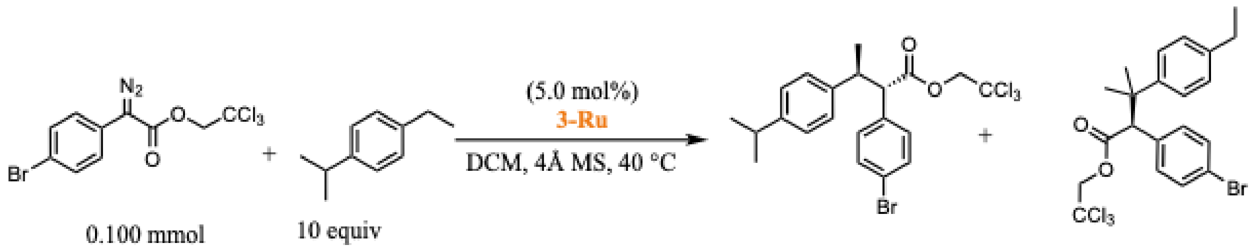

N2
O
CCl3
Br
0.100 mmol
+
10 equiv
(5.0 mol%)
3-Ru
DCM, 4Å MS, 40 °C

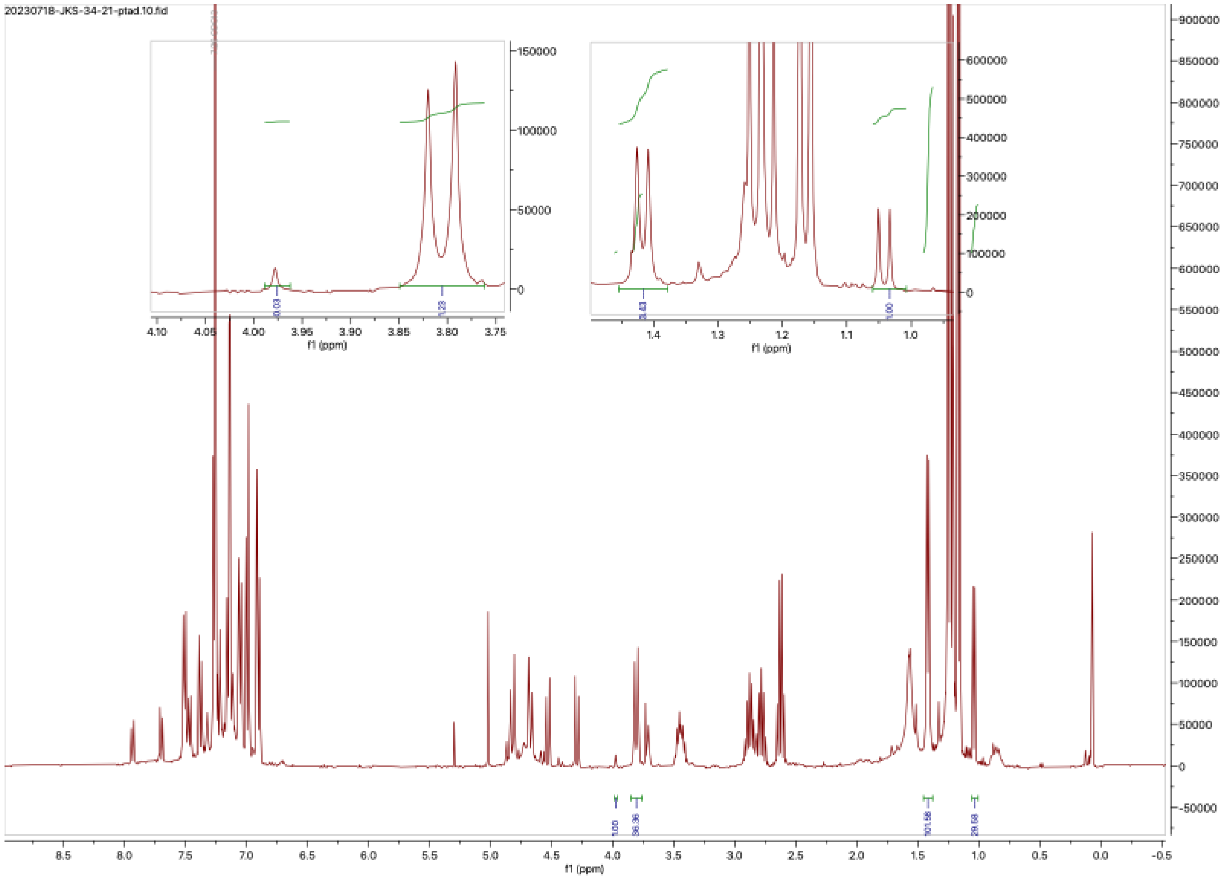

20230718-JKS-34-21-ptad.10.fid
f1 (ppm)

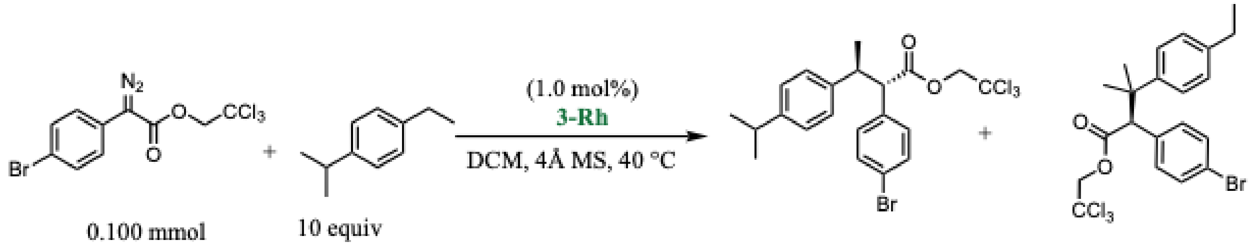
N2
CCl3
Br
O
+
(1.0 mol%)
3-Rh
DCM, 4Å MS, 40 °C
0.100 mmol
10 equiv

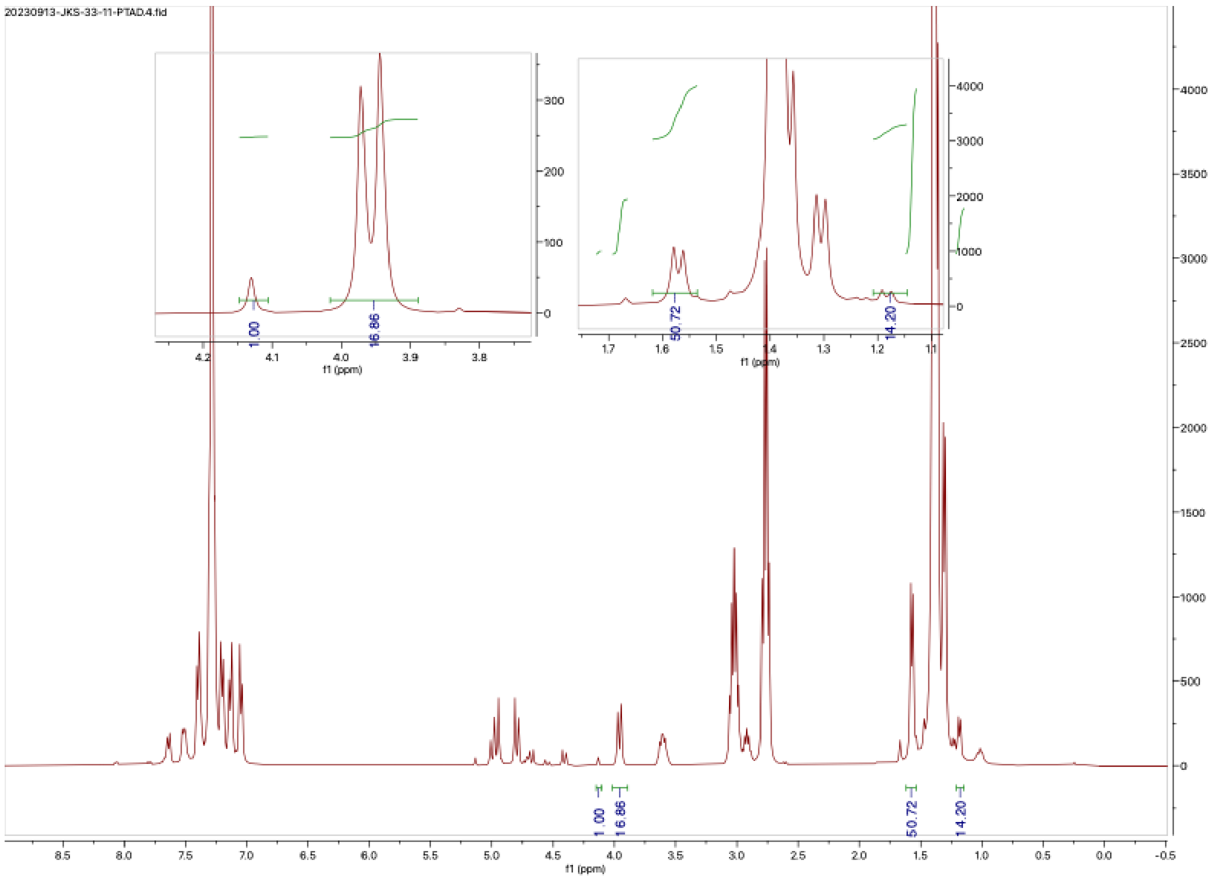
20230913-JKS-33-11-PTAD.4.fid
f1 (ppm)
1.00
16.86
50.72
14.20

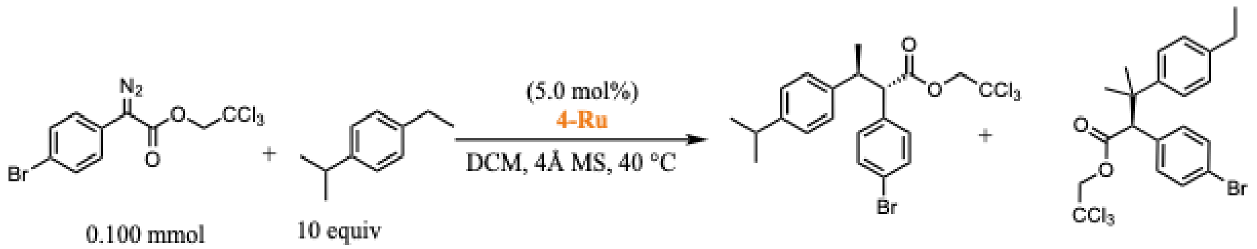

0.100 mmol
10 equiv
(5.0 mol%)
4-Ru
DCM, 4Å MS, 40 °C

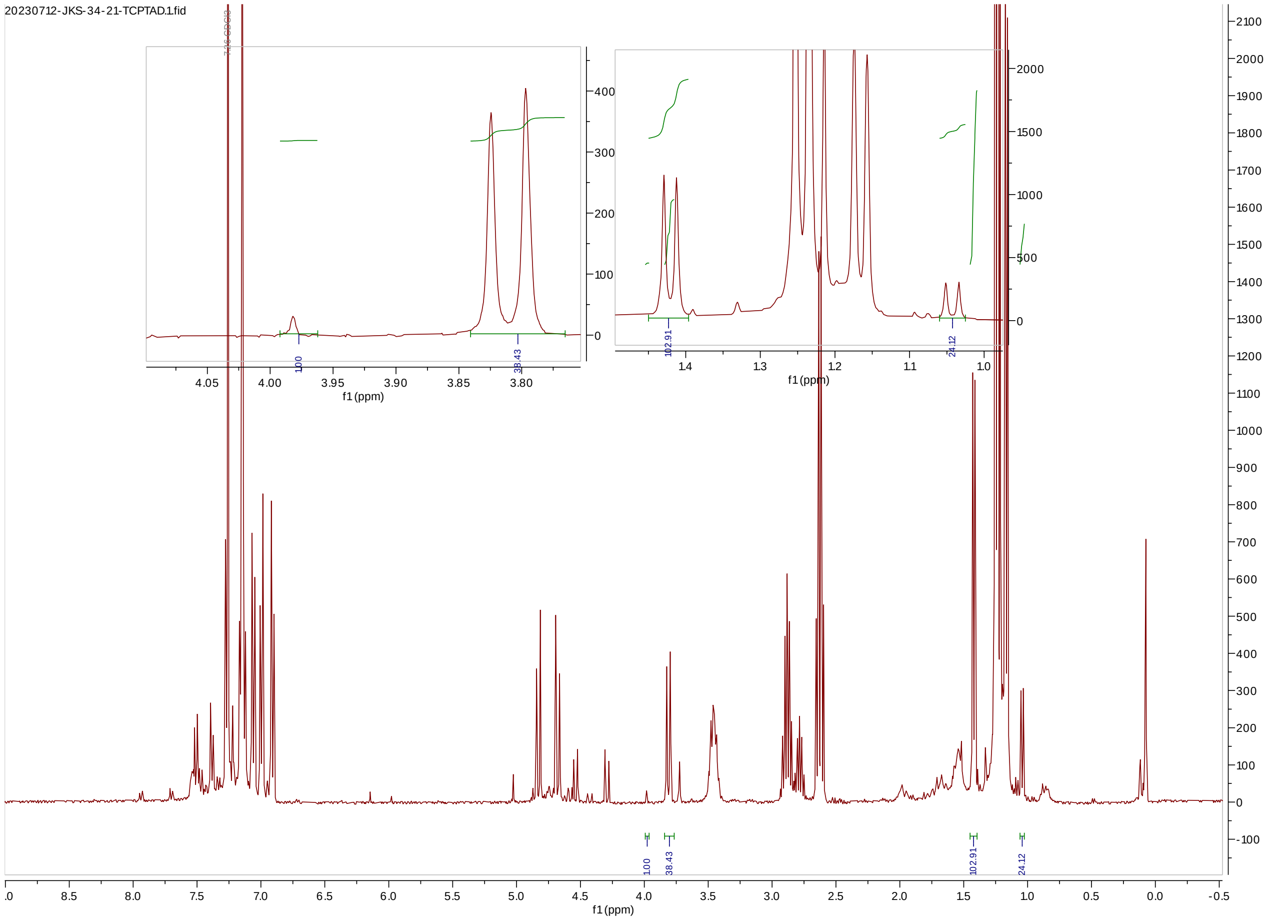

20230712-JKS-34-21-TCPTAD.1.fid
f1 (ppm)

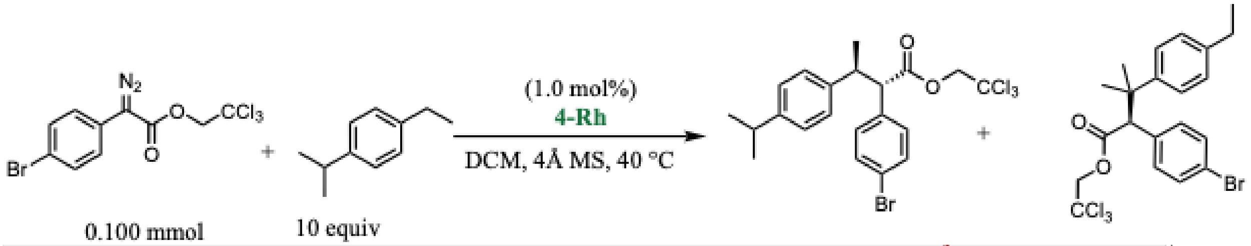
N2
Br
O
O
CCl3
0.100 mmol
10 equiv
(1.0 mol%)
4-Rh
DCM, 4Å MS, 40 °C
Br
CCl3
O
O
CCl3
Br

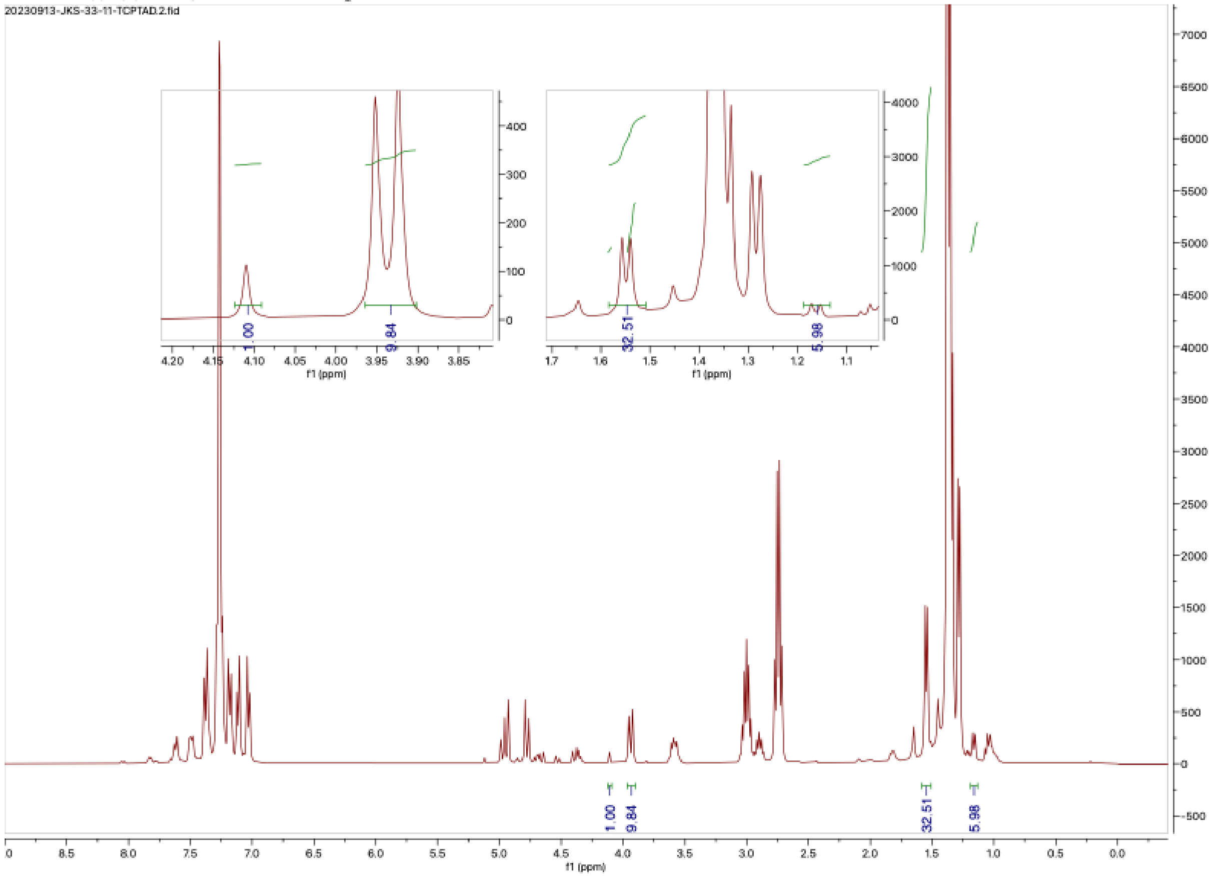
20230913-JKS-33-11-TCPTAD.2.fid
1.00
9.84
32.51
5.98
f1 (ppm)

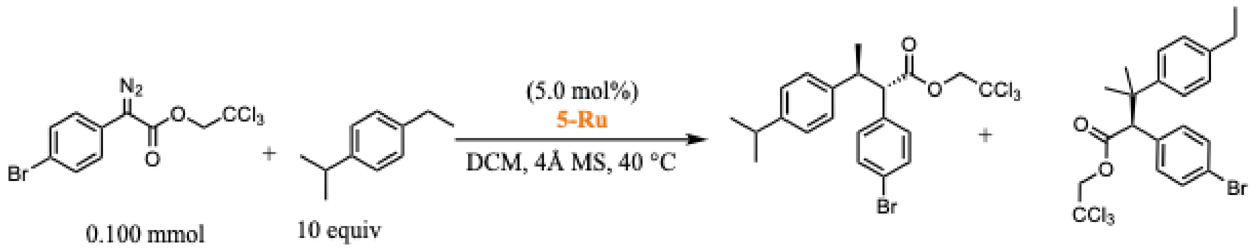
N2
CCl3
Br
O
0.100 mmol
10 equiv
(5.0 mol%)
5-Ru
DCM, 4Å MS, 40 °C
O
CCl3
Br
O
O
CCl3
Br

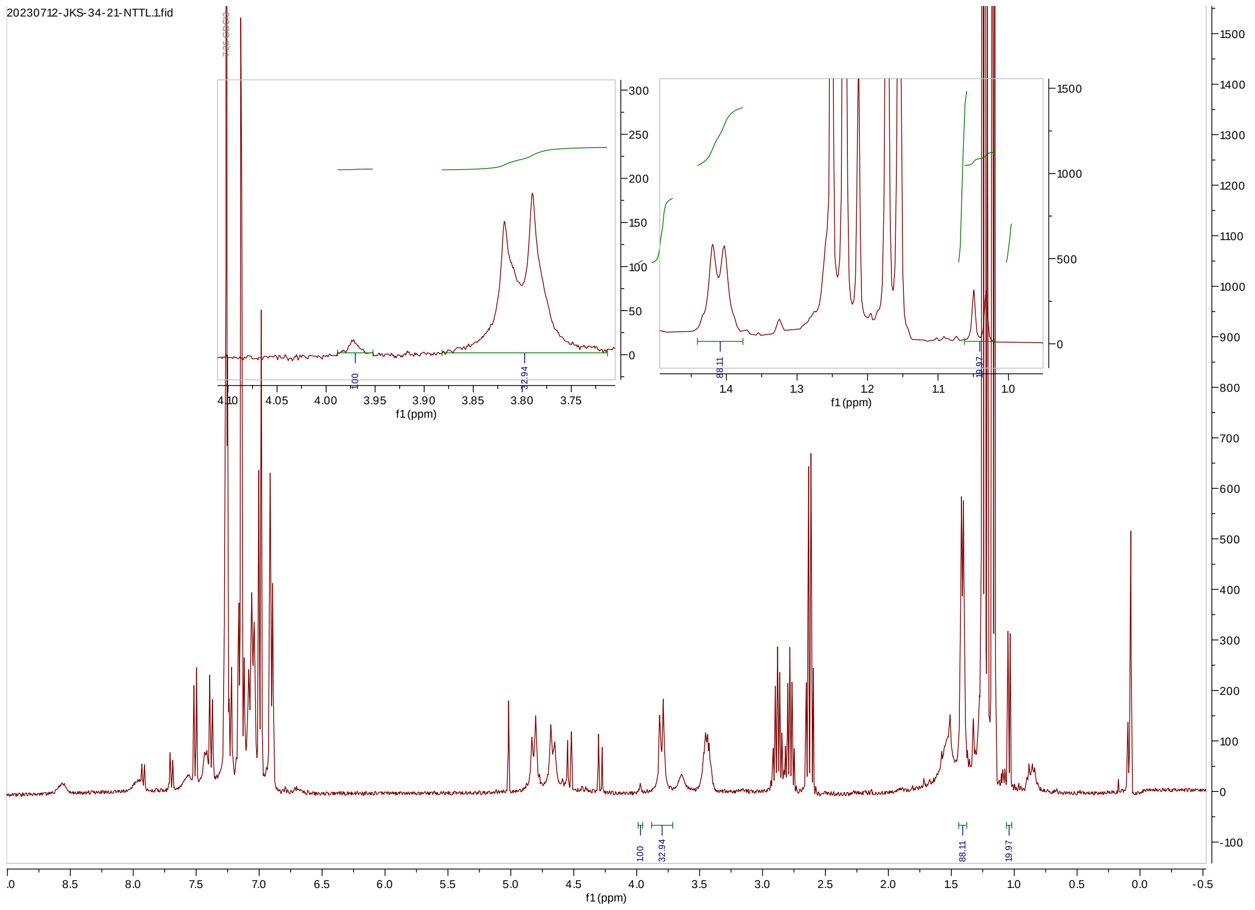
20230712-JKS-34-21-NTTL.1.fid
f1 (ppm)
1.00
32.94
88.11
19.97

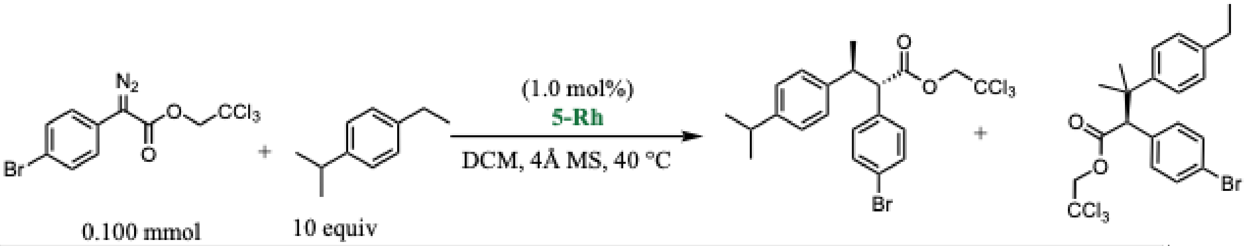

0.100 mmol 10 equiv

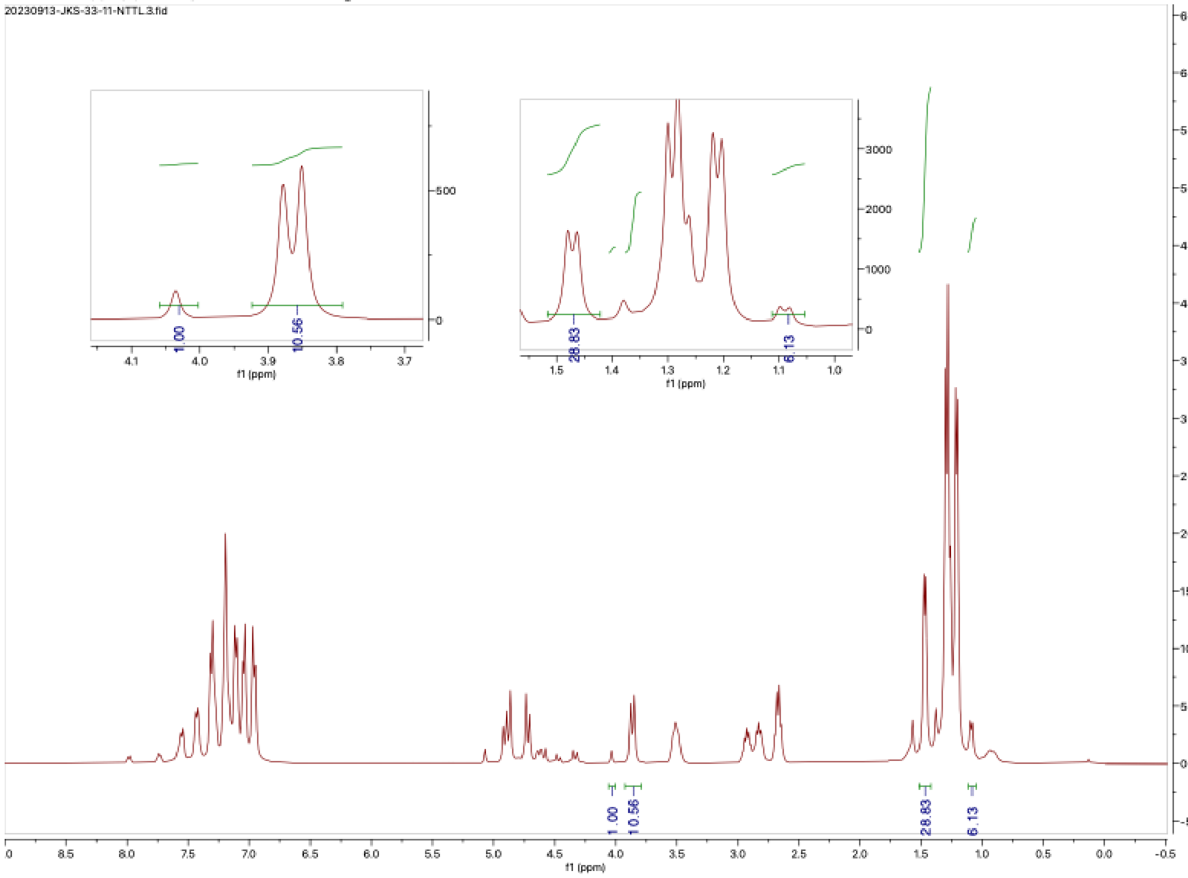

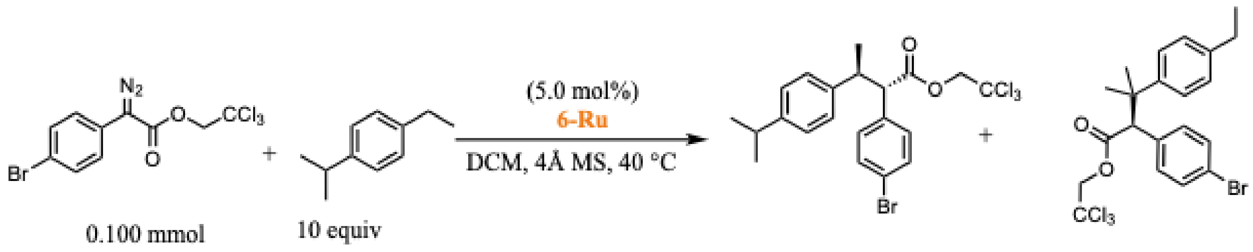

(5.0 mol%)
6-Ru
DCM, 4Å MS, 40 °C
0.100 mmol
10 equiv

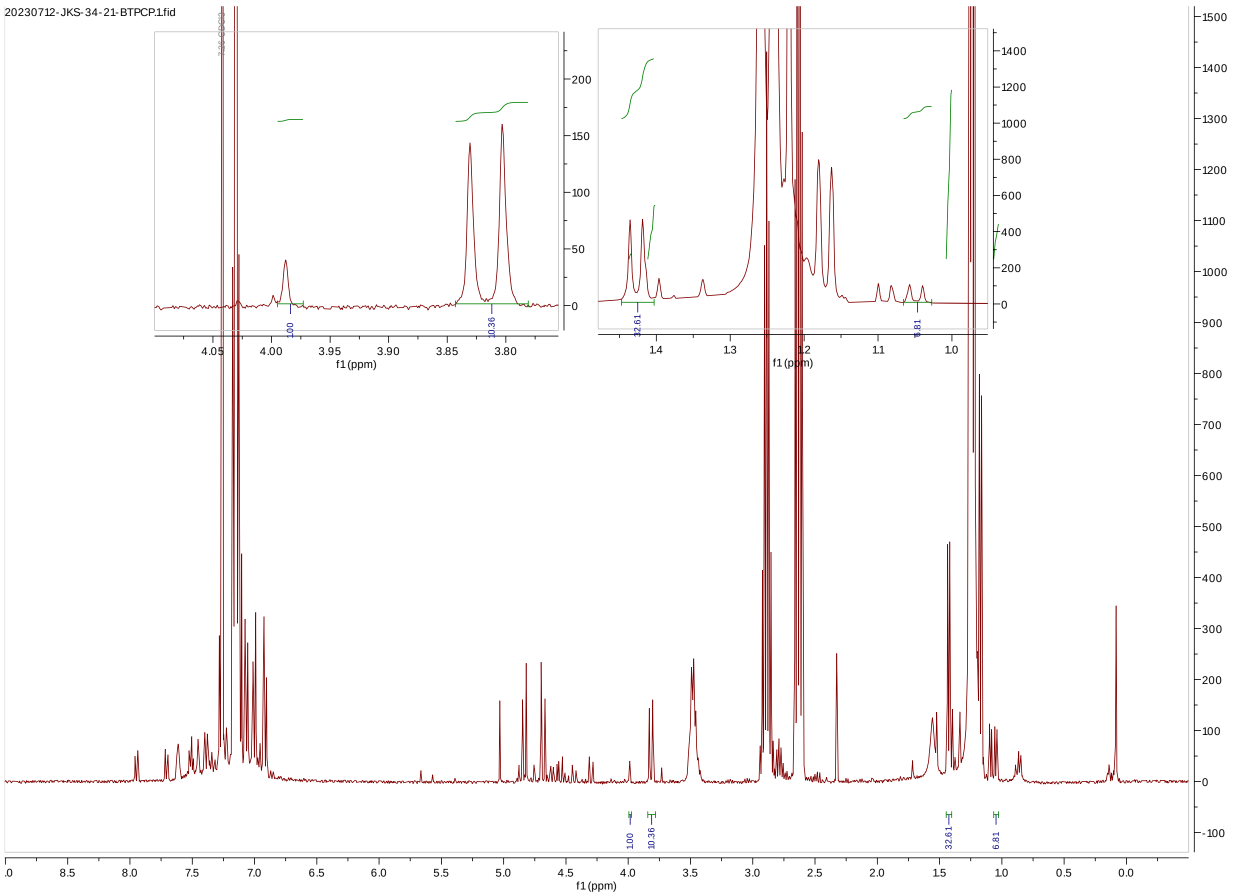

20230712-JKS-34-21-BTPCP.1.fid
f1 (ppm)

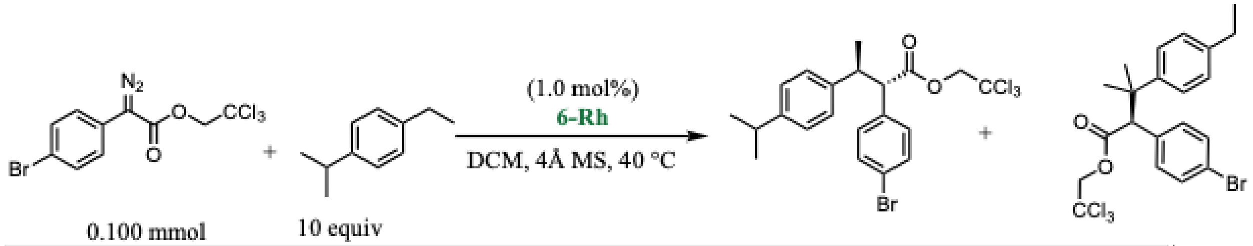

N2
CCl3
Br
(1.0 mol%)
6-Rh
DCM, 4Å MS, 40 °C
0.100 mmol
10 equiv

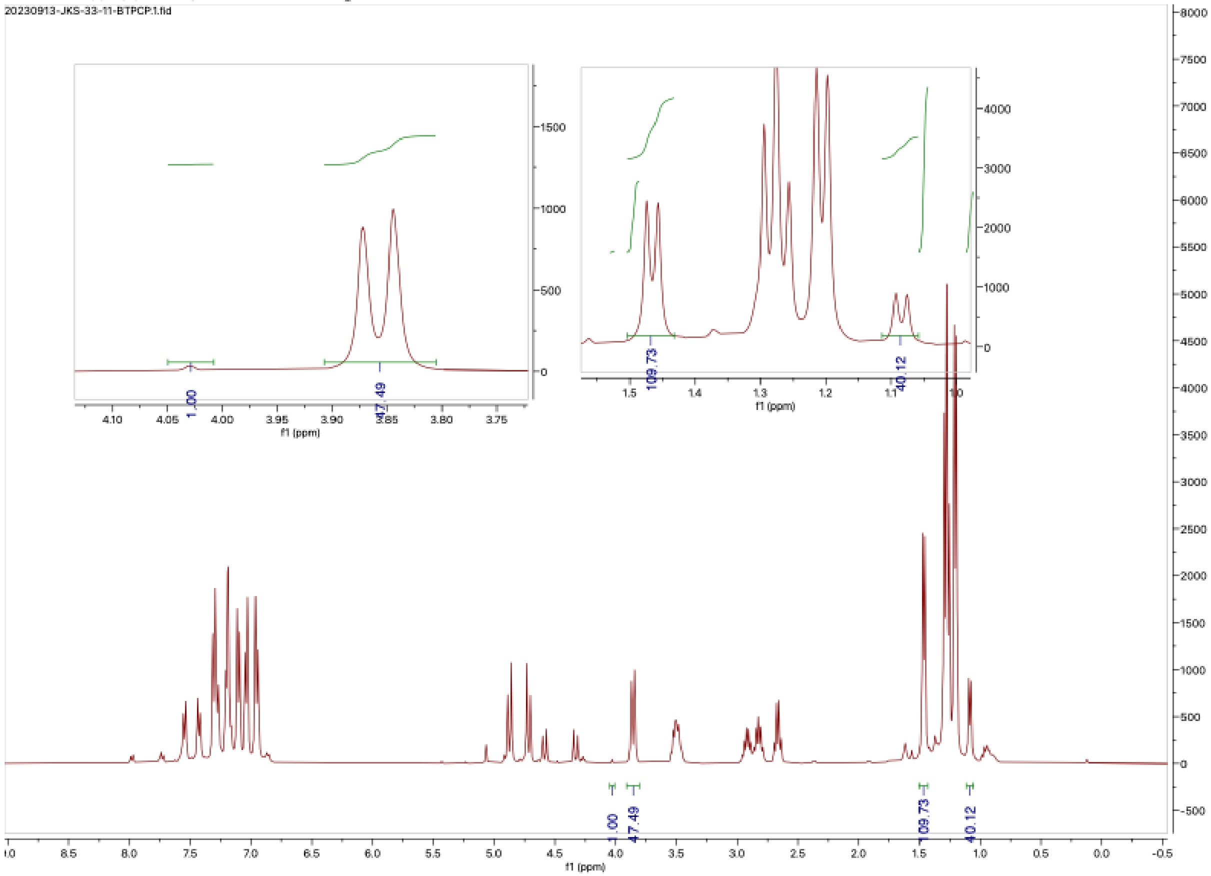

20230913-JKS-33-11-BTPCP.1.fid
f1 (ppm)

Regioselectivity and diastereoselectivity determination for reactions with *tert*-butylcyclohexane

Regioselectivity and diastereoselectivity were determined through previously reported analysis.[4]

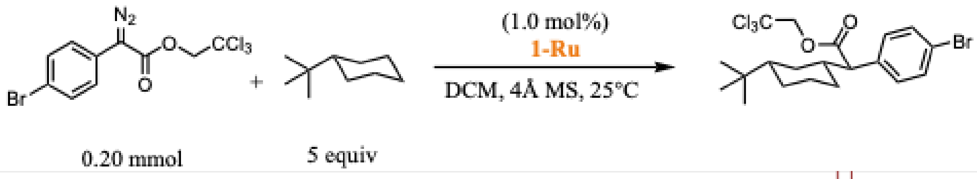

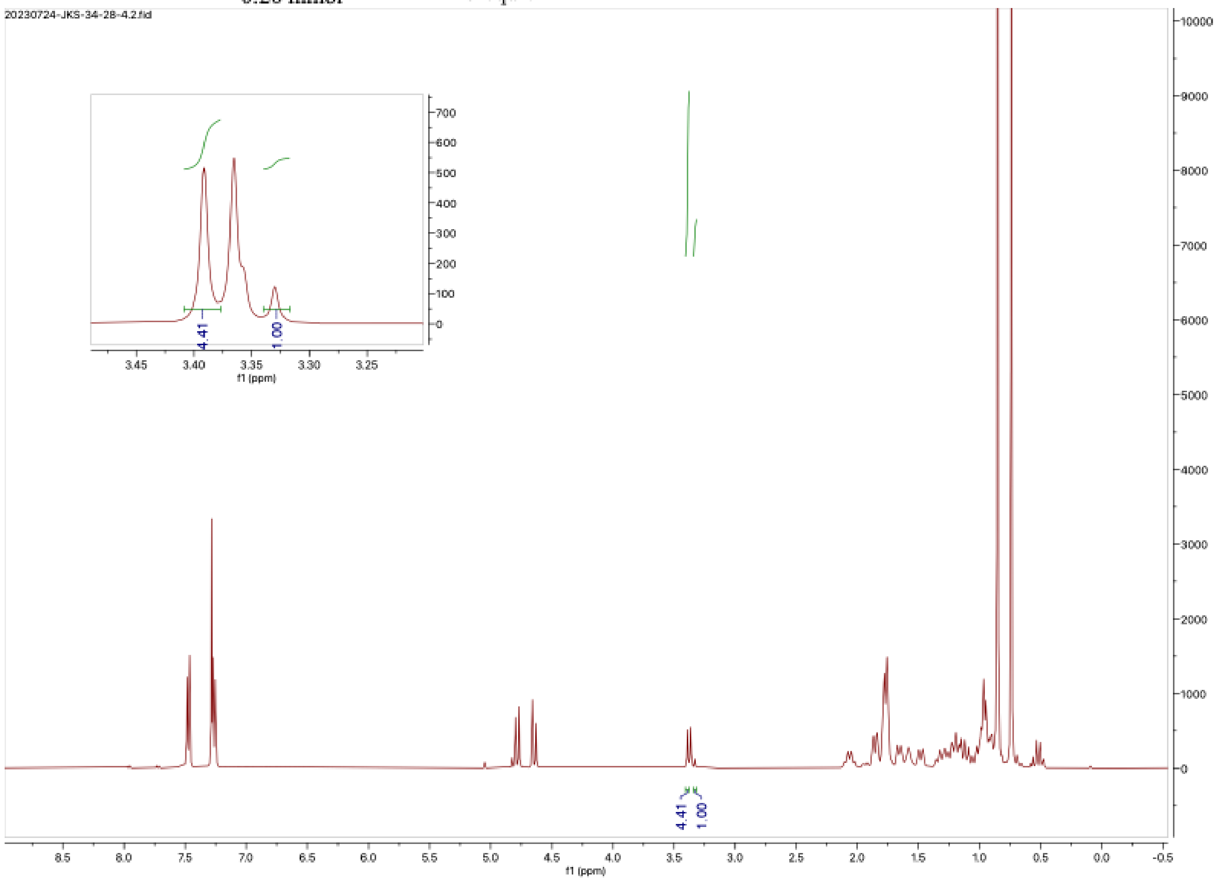

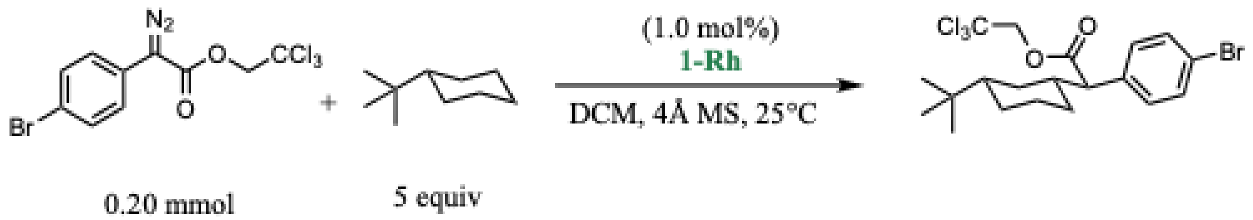
N2
CCl3
Br
O
(1.0 mol%)
1-Rh
DCM, 4Å MS, 25°C
Cl3C
0.20 mmol
5 equiv

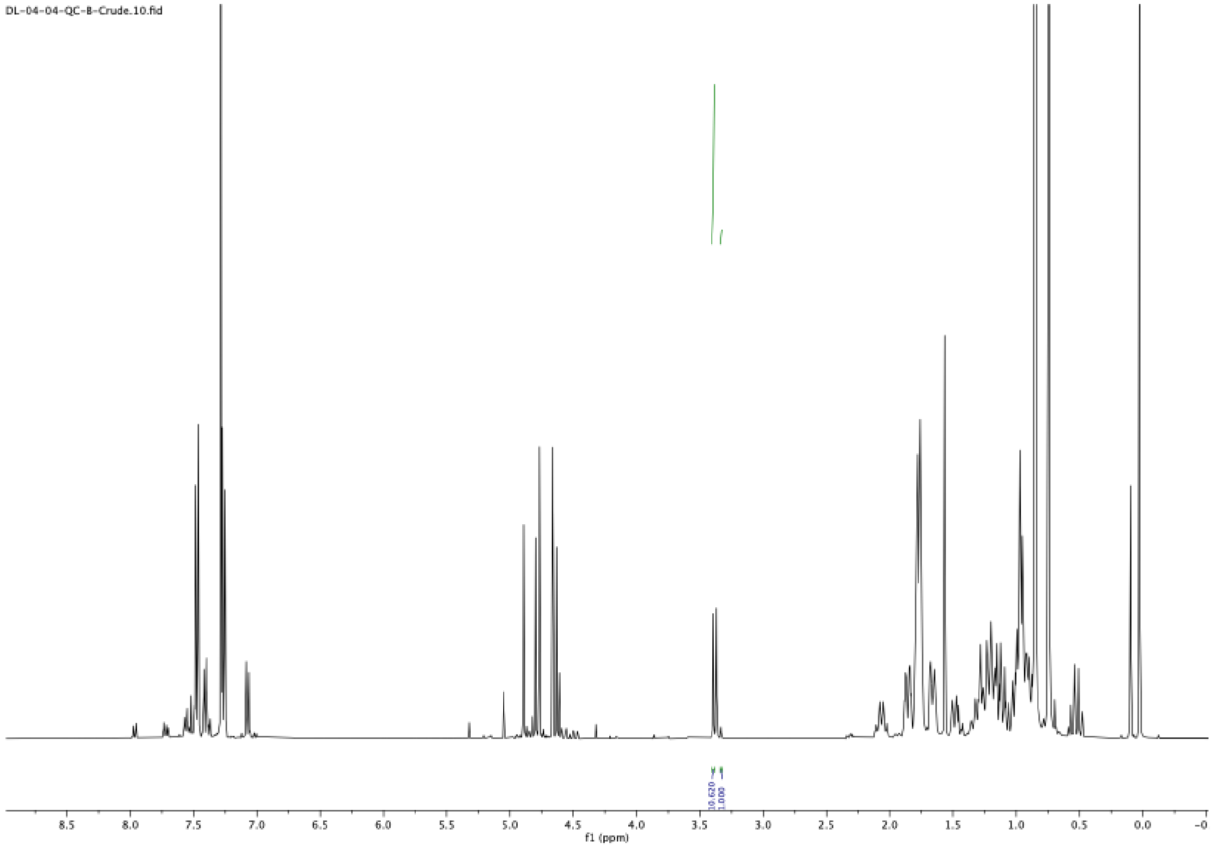
DL-04-04-QC-B-Crude.10.fid
10.620
1.000
8.5 8.0 7.5 7.0 6.5 6.0 5.5 5.0 4.5 4.0 3.5 3.0 2.5 2.0 1.5 1.0 0.5 0.0
f1 (ppm)

Regioselectivity and diastereoselectivity determination for reactions with pentane. Regioselectivity and diastereoselectivity were determined through previously reported analysis.[5]

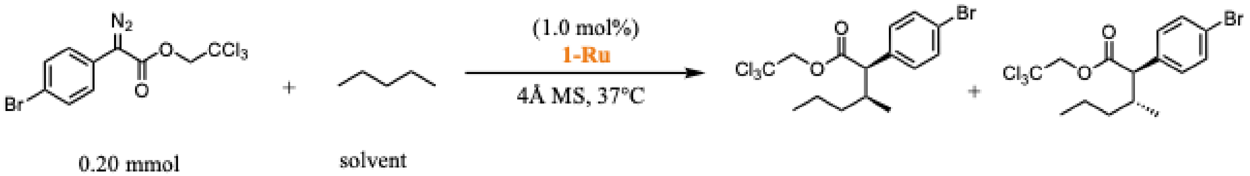

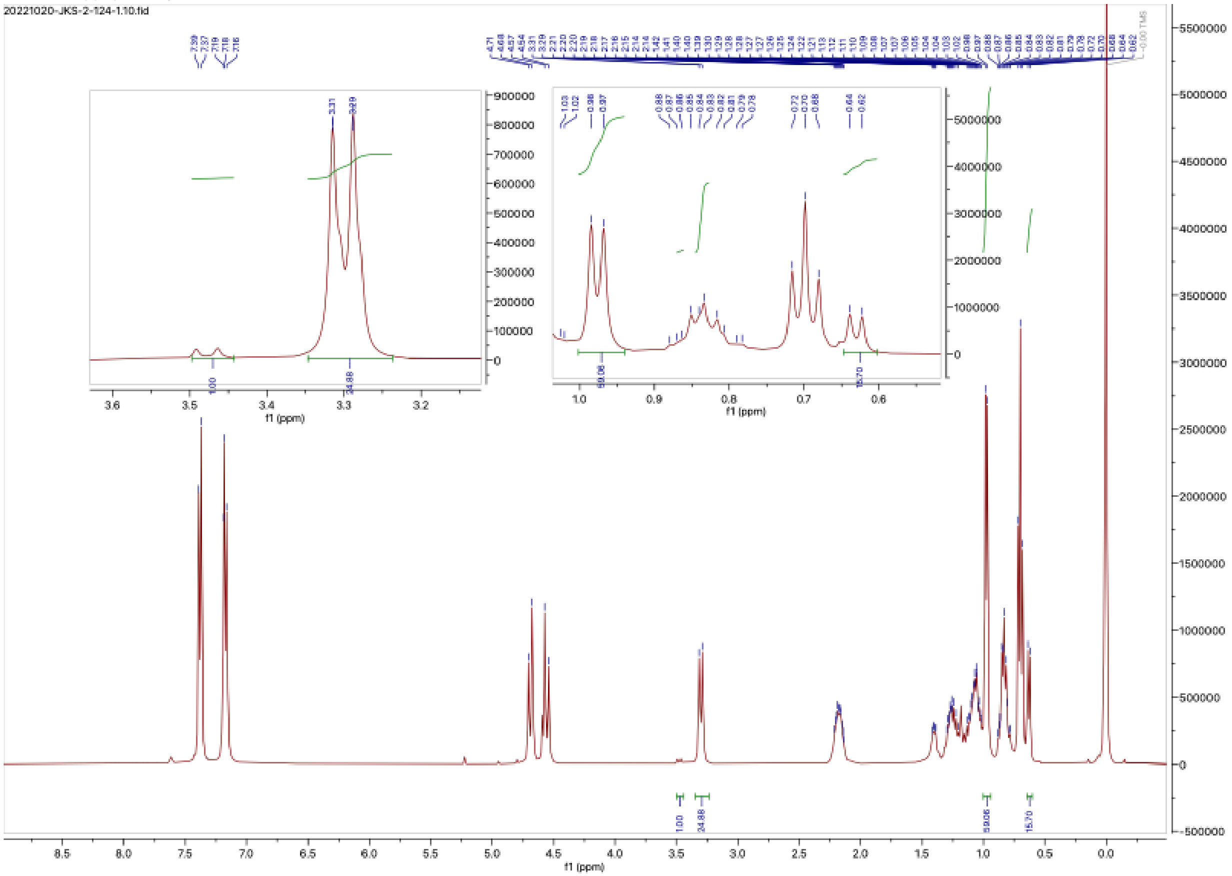

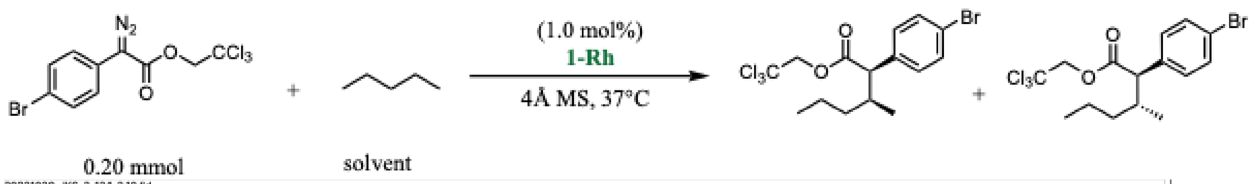
(1.0 mol%)
1-Rh
4Å MS, 37°C
0.20 mmol
solvent

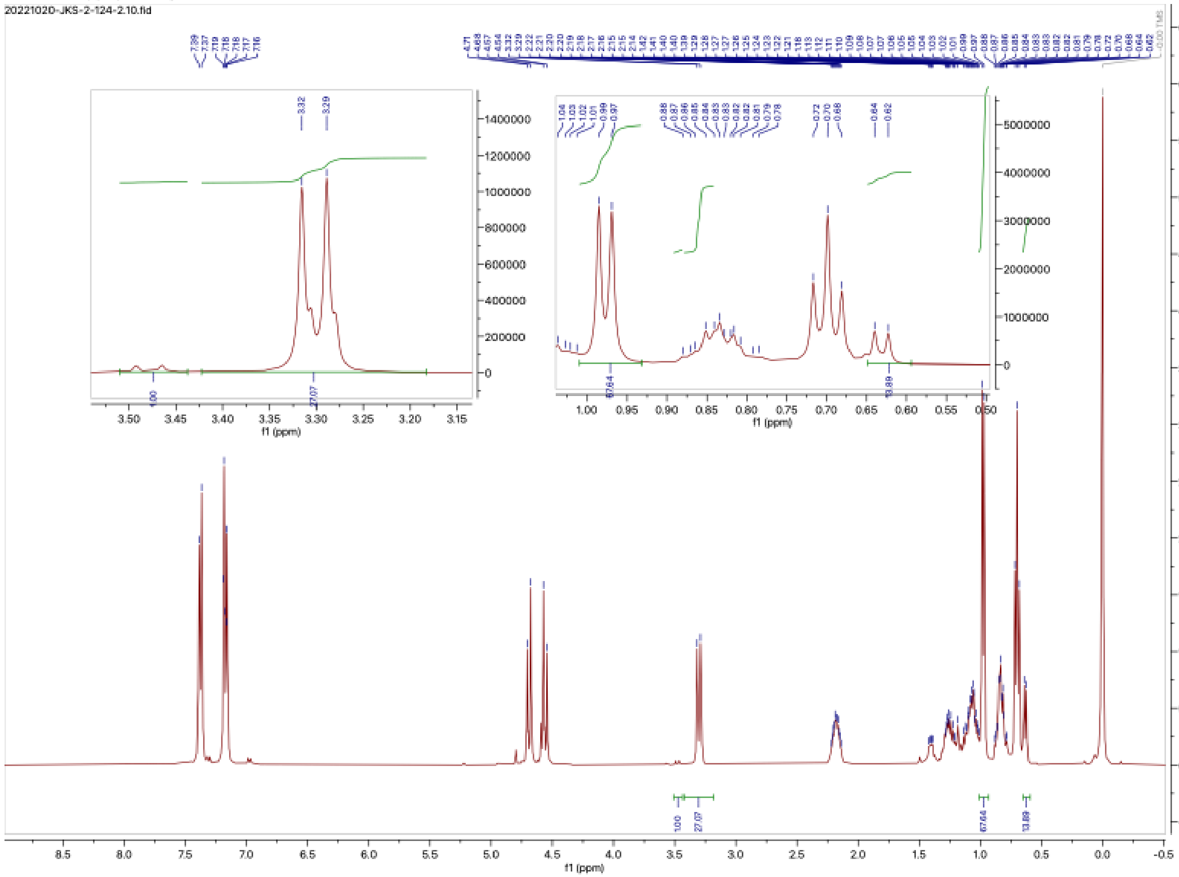
20221020-JKS-2-124-2.10.fid
f1 (ppm)

Regioselectivity and diastereoselectivity determination for reactions with 2-hexene. The regioselectivity was determined through integration between dd at 3.72 ppm (primary insertion) and two doublets at 3.54 ppm (secondary insertion with diastereomer). Diastereomers of secondary insertion product was determined through integration of triplet at 0.77 ppm and triplet at 0.91 ppm.

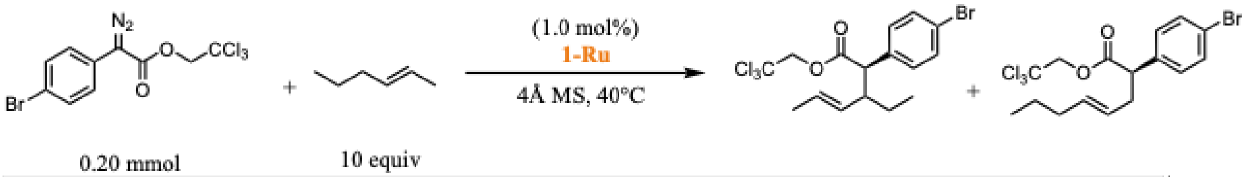

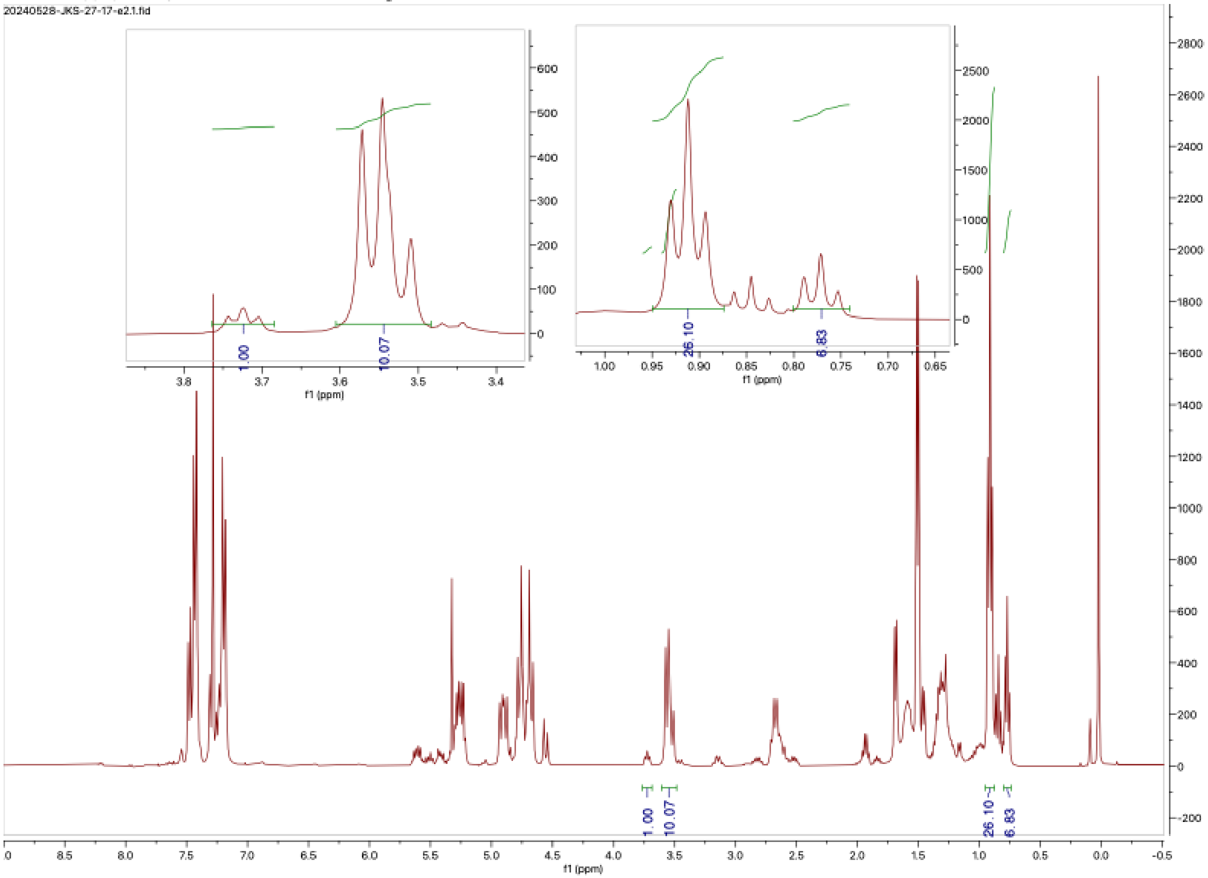

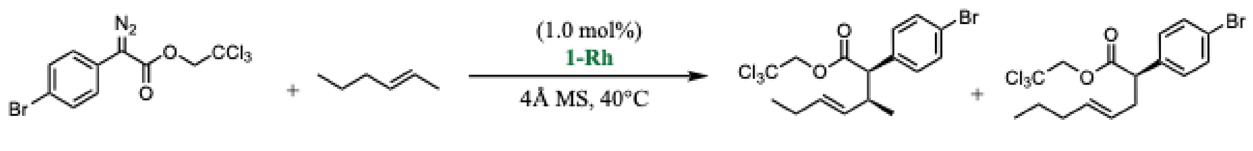
(1.0 mol%)
1-Rh
4Å MS, 40°C
0.20 mmol
10 equiv

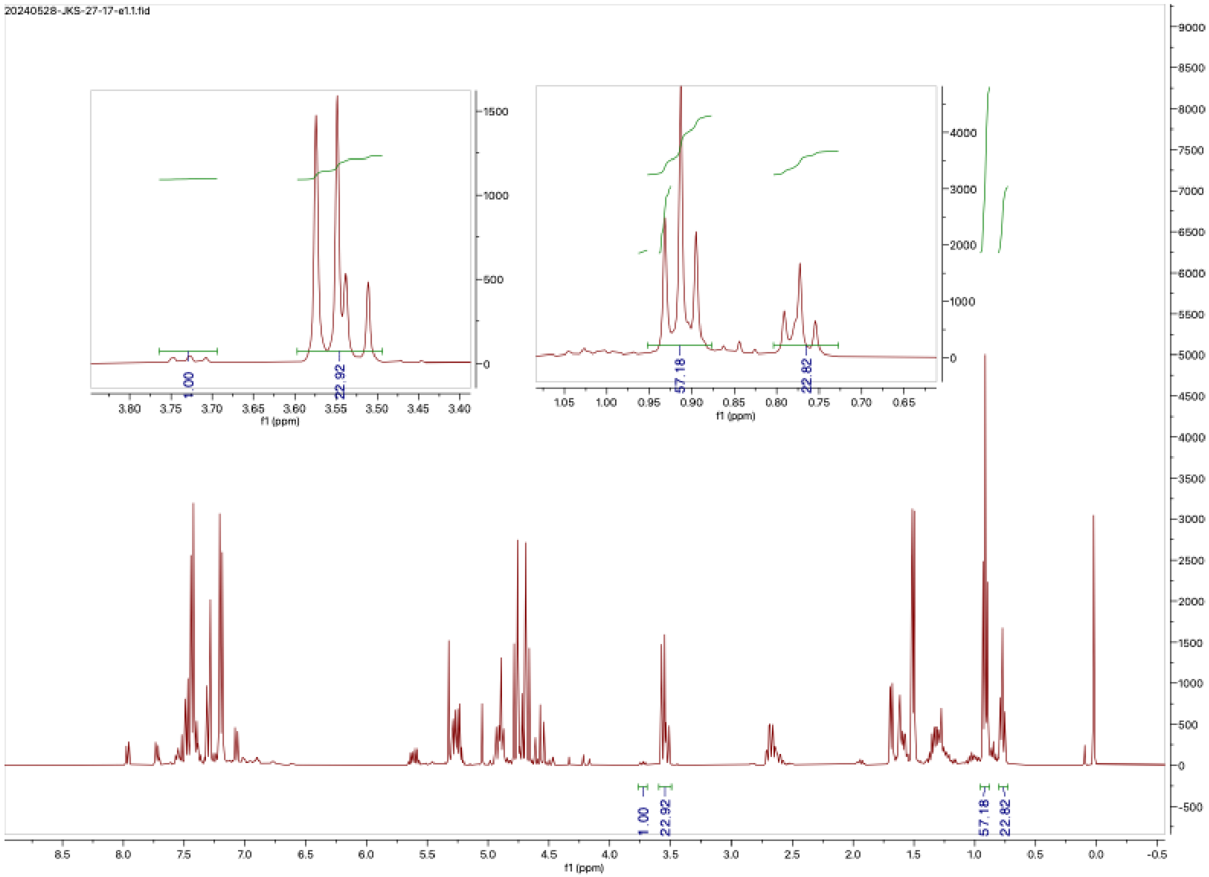
20240528-JKS-27-17-e1.1.fid
f1 (ppm)
1.00
22.92
57.18
22.82

Regioselectivity and diastereoselectivity determination for reactions with 2-hexene. The regioselectivity was determined through comparing integration of the multiplets at 4.75 ppm and 4.52 ppm.

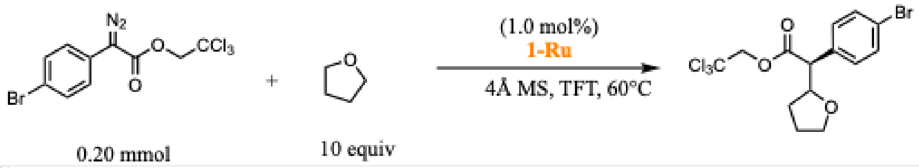

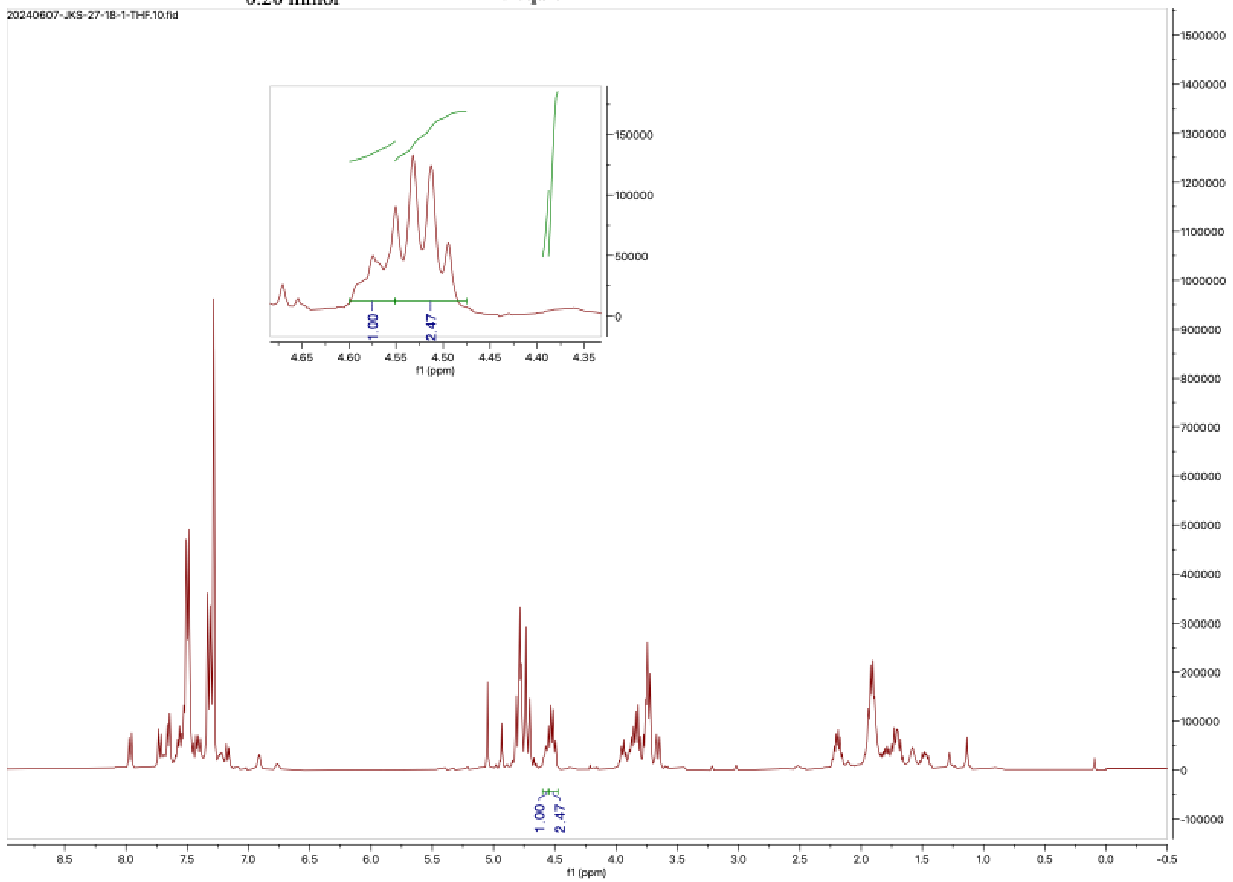

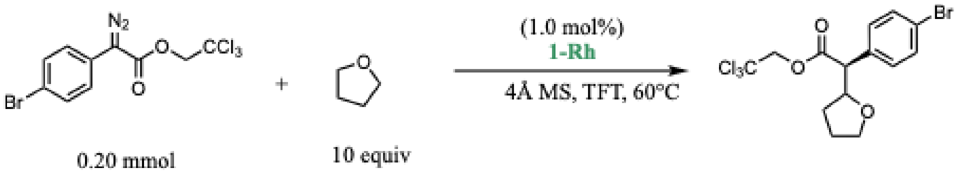
N2
CCl3
Br
0.20 mmol
+
10 equiv
(1.0 mol%)
1-Rh
4Å MS, TFT, 60°C
Cl3C
O
Br

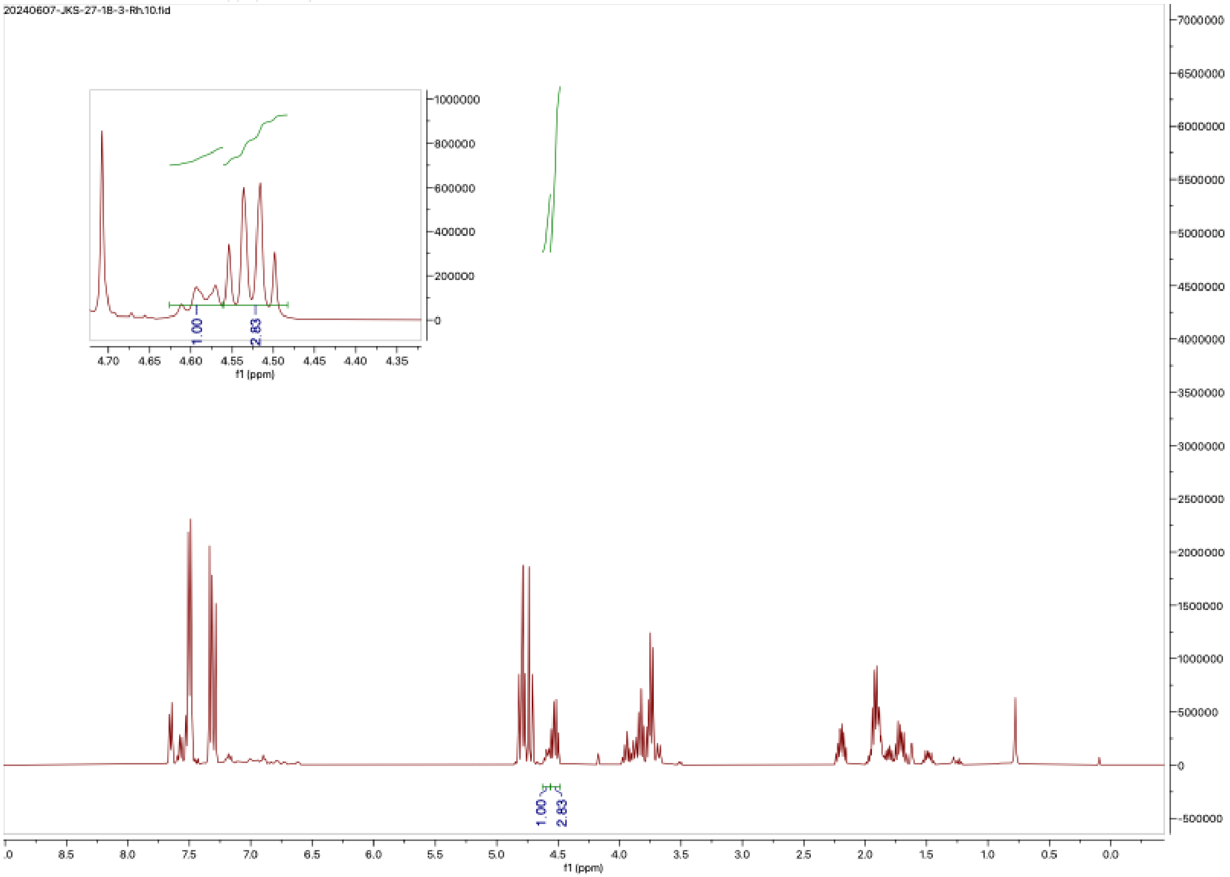
20240607-JKS-27-18-3-Rh.10.fid
f1 (ppm)
1.00
2.83

Regioselectivity Determination for Competition Reactions:

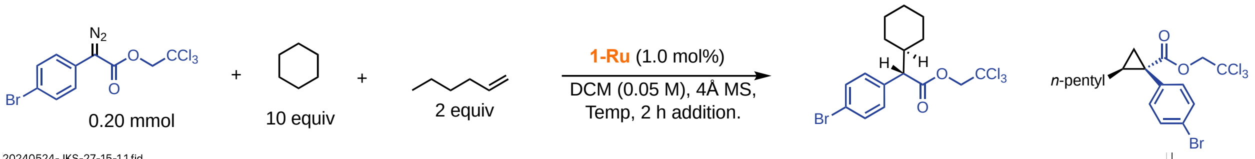

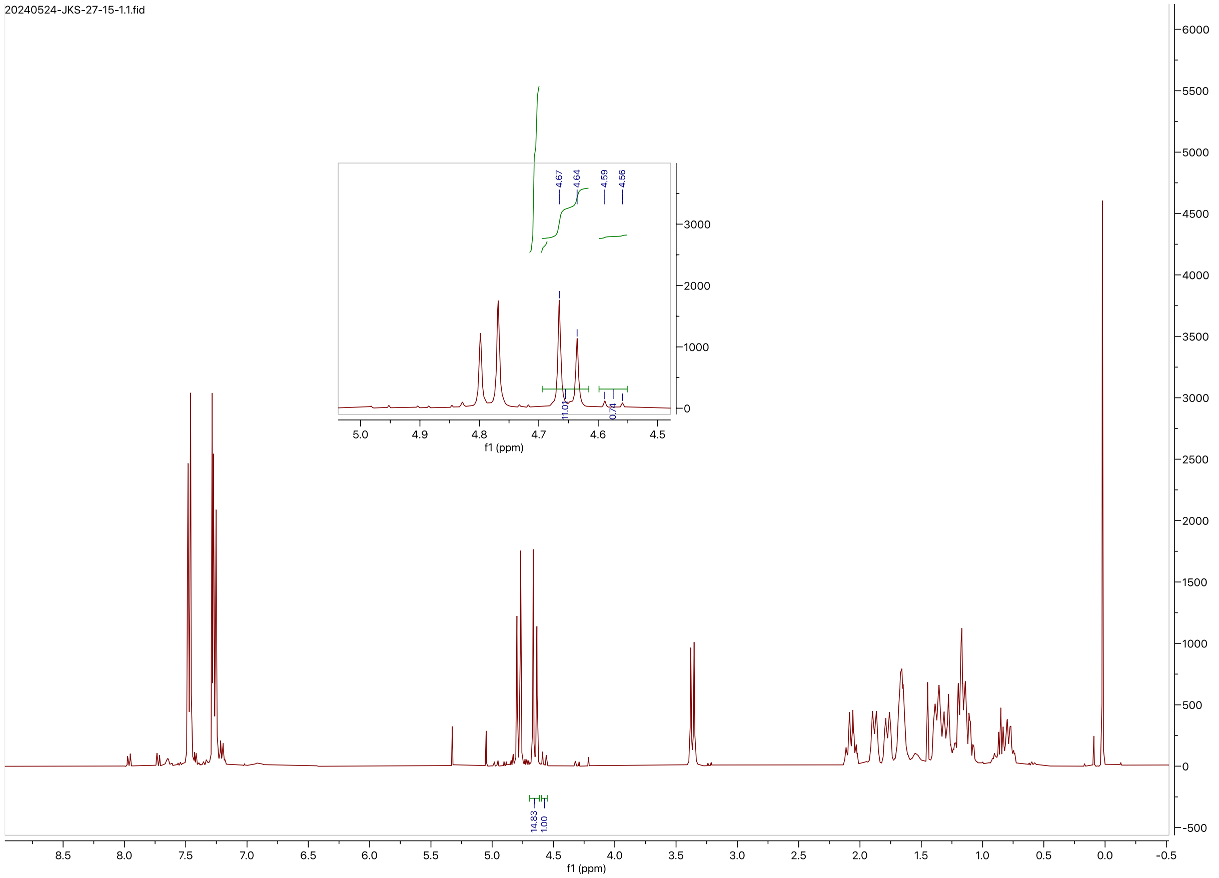

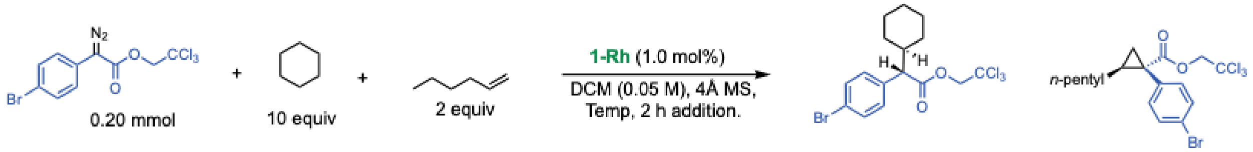
0.20 mmol
10 equiv
2 equiv
1-Rh (1.0 mol%)
DCM (0.05 M), 4Å MS,
Temp, 2 h addition.
n-pentyl

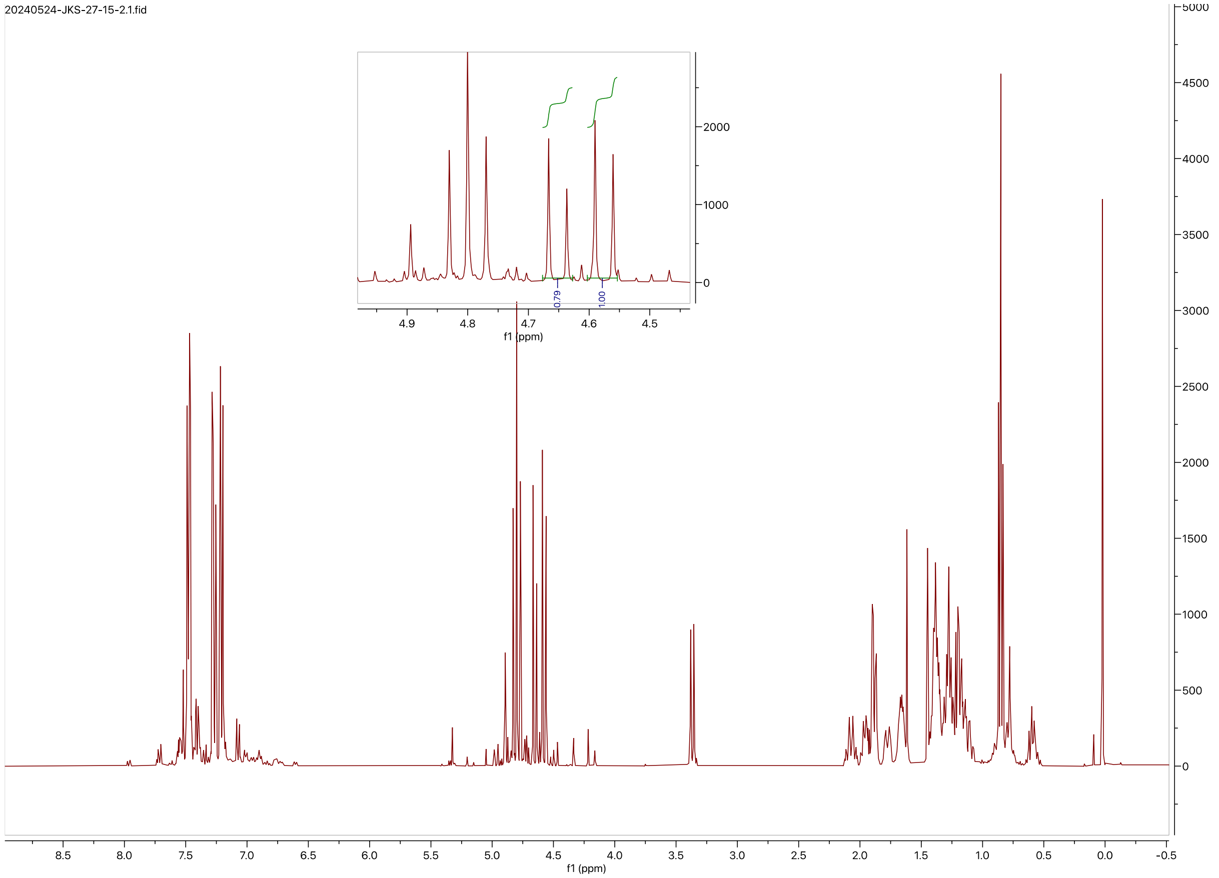
20240524-JKS-27-15-2.1.fid
f1 (ppm)
0.79
1.00

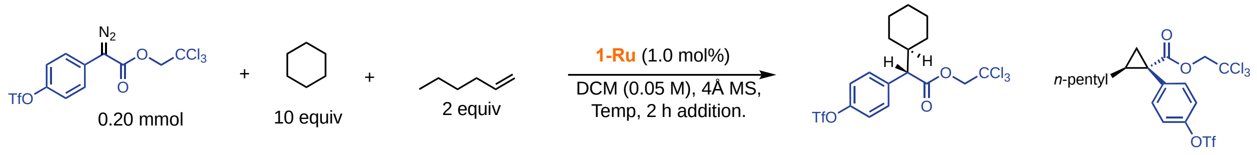
N2
CCl3
TfO
0.20 mmol
+
10 equiv
+
2 equiv
1-Ru (1.0 mol%)
DCM (0.05 M), 4Å MS,
Temp, 2 h addition.
H
H
O
CCl3
TfO
O
n-pentyl
O
O
CCl3
OTf

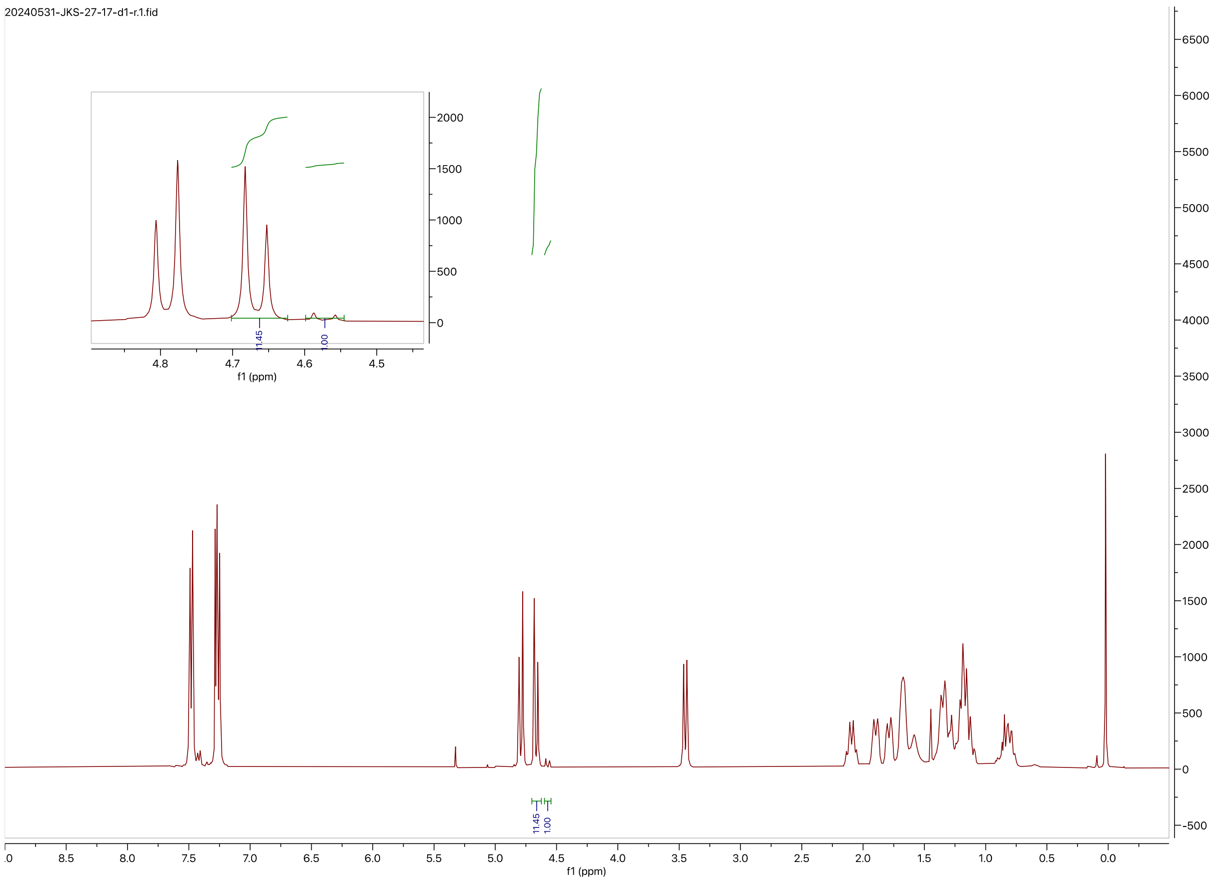
20240531-JKS-27-17-d1-r.1.fid
11.45
1.00
f1 (ppm)

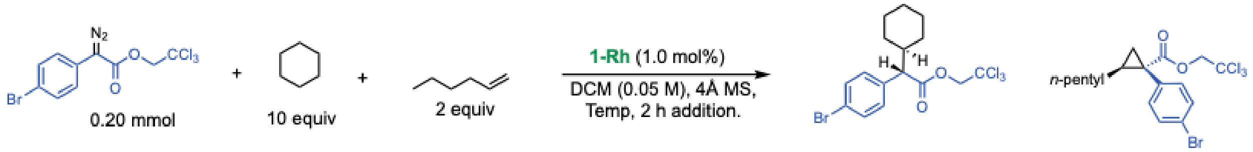
N2
CCl3
Br
0.20 mmol
+
10 equiv
+
2 equiv
1-Rh (1.0 mol%)
DCM (0.05 M), 4Å MS,
Temp, 2 h addition.
H
H
CCl3
Br
n-pentyl
CCl3
Br

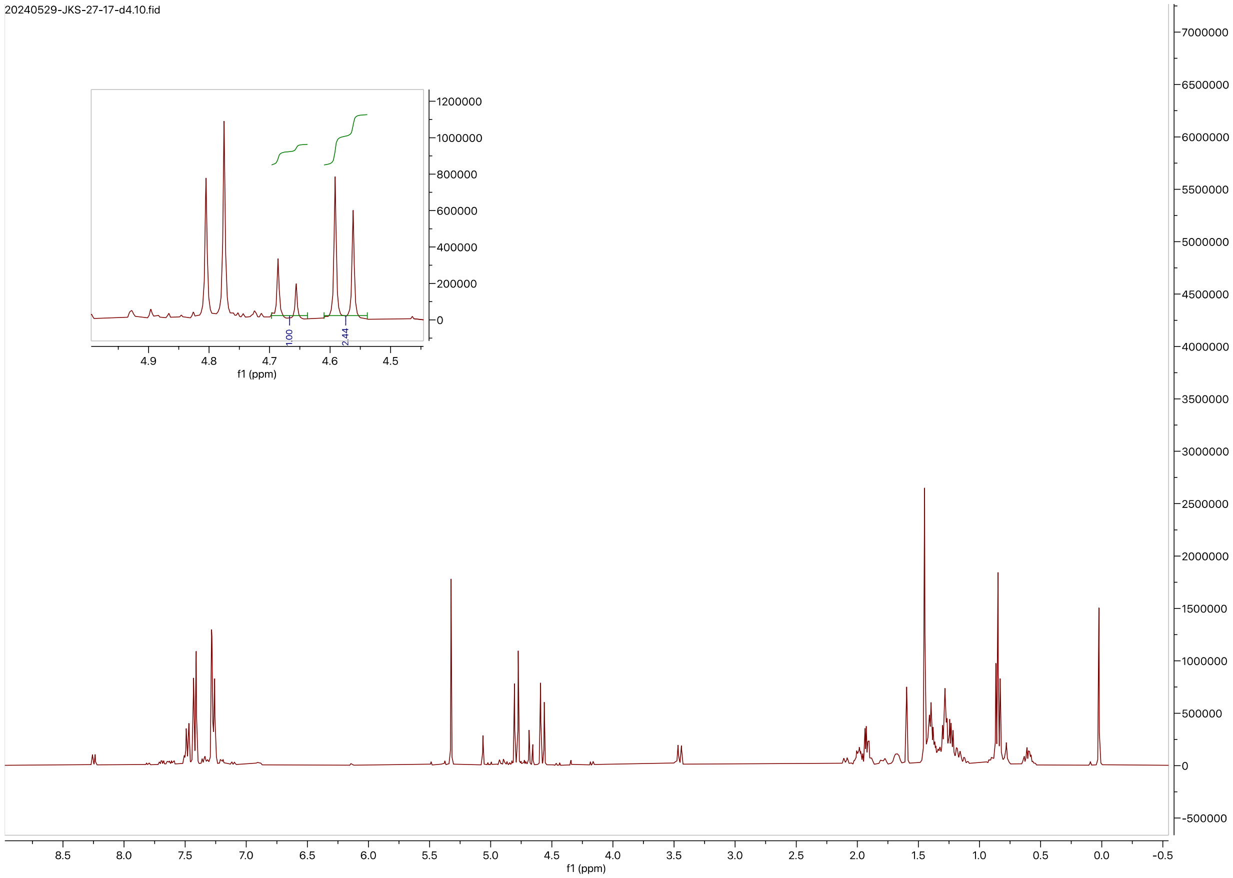
20240529-JKS-27-17-d4.10.fid
1.00
2.44
f1 (ppm)
f1 (ppm)

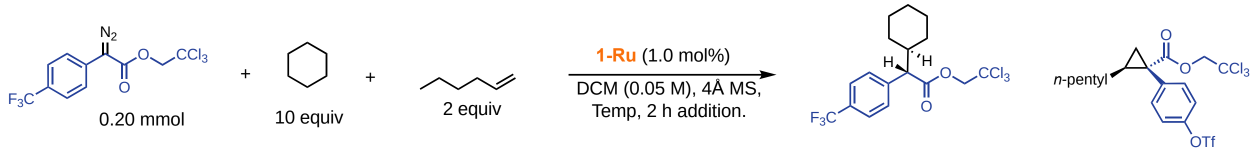
N2
CCl3
F3C
0.20 mmol
10 equiv
2 equiv
1-Ru (1.0 mol%)
DCM (0.05 M), 4Å MS,
Temp, 2 h addition.
n-pentyl
OTf

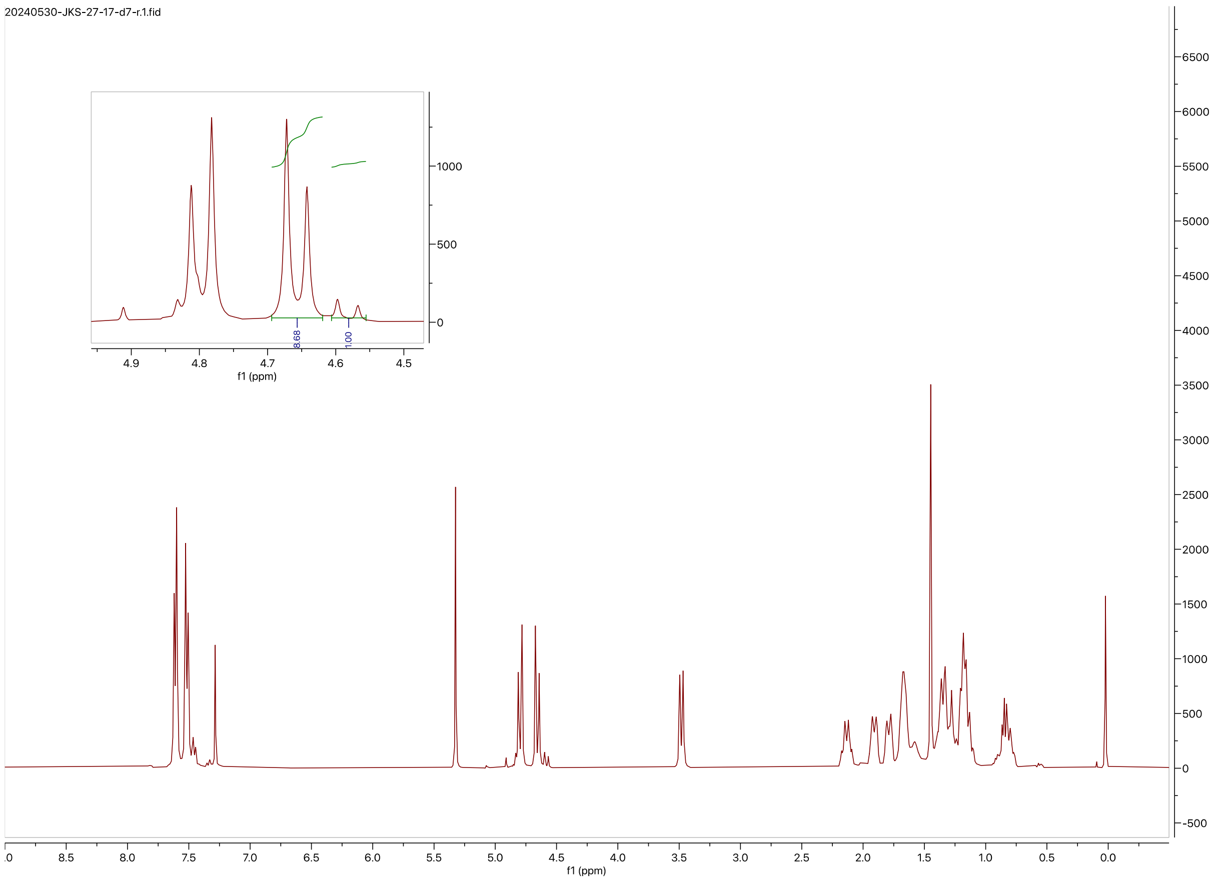
20240530-JKS-27-17-d7-r.1.fid
f1 (ppm)

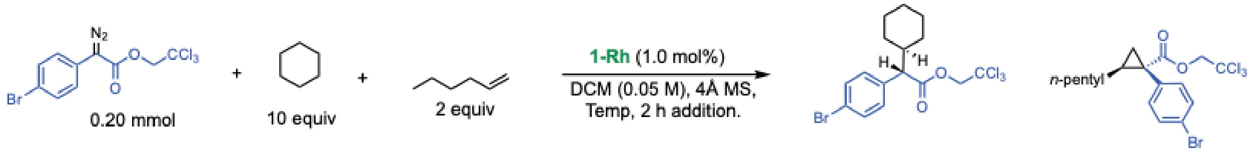
0.20 mmol
10 equiv
2 equiv
1-Rh (1.0 mol%)
DCM (0.05 M), 4Å MS,
Temp, 2 h addition.
n-pentyl

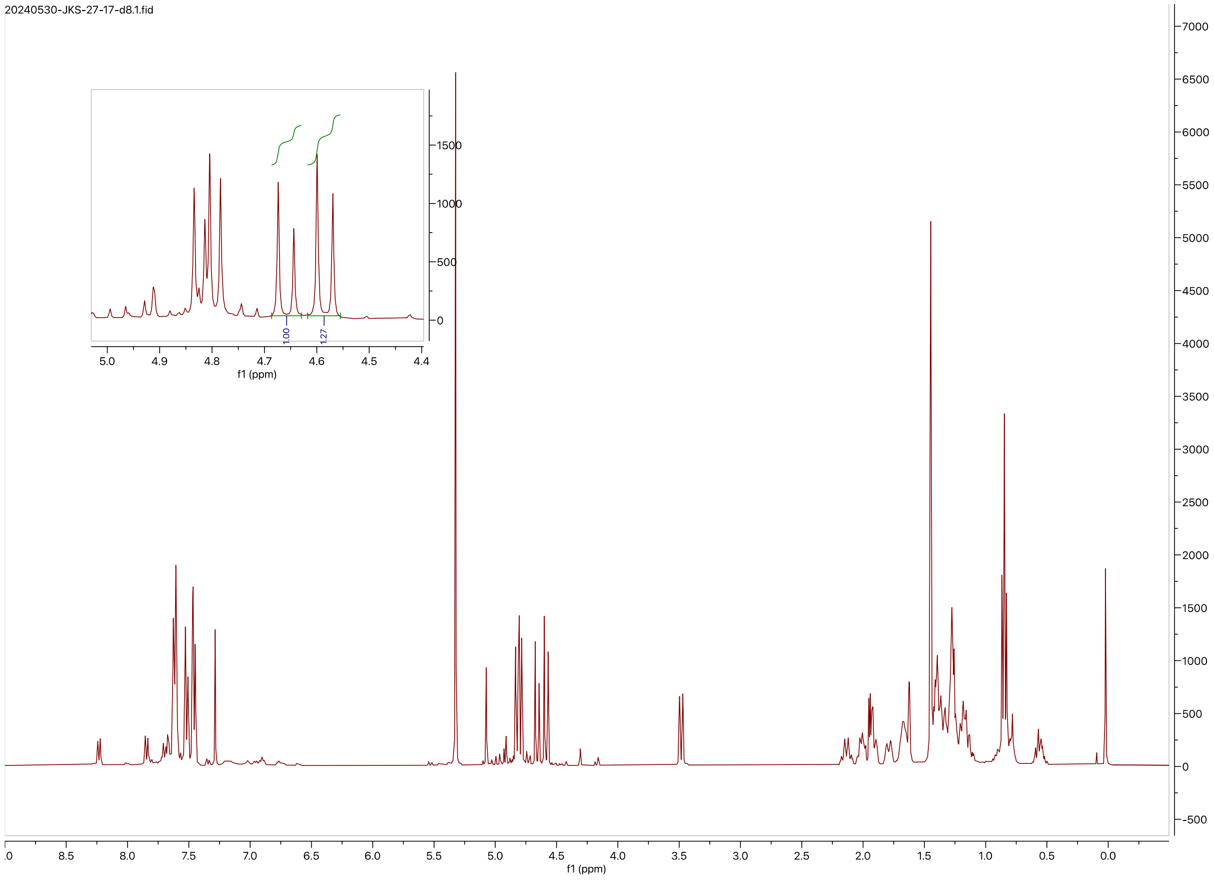
20240530-JKS-27-17-d8.1.fid
f1 (ppm)
1.00
1.27

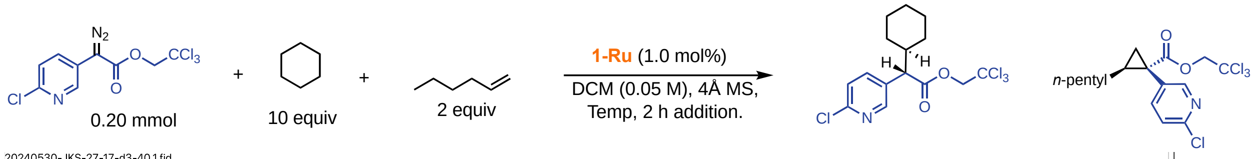
0.20 mmol
10 equiv
2 equiv
1-Ru (1.0 mol%)
DCM (0.05 M), 4Å MS,
Temp, 2 h addition.
n-pentyl

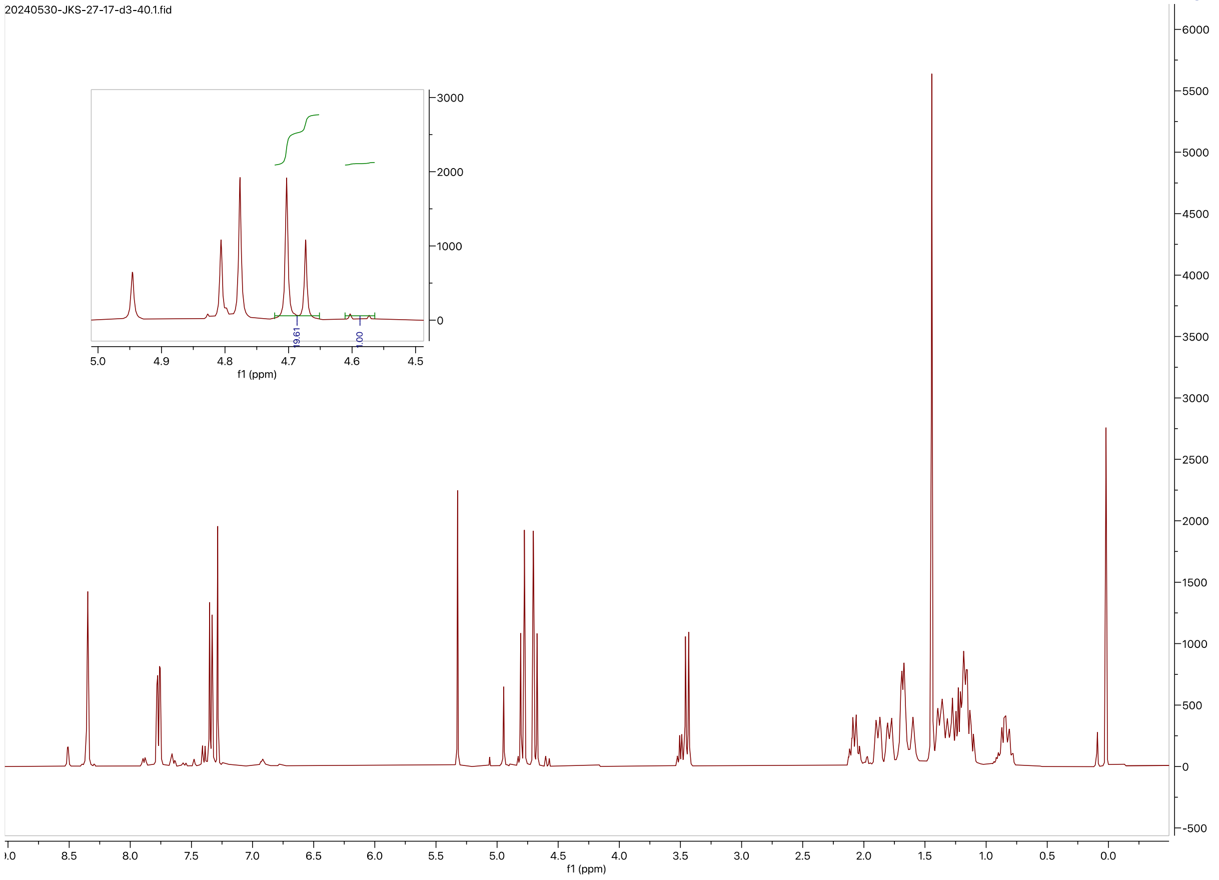
20240530-JKS-27-17-d3-40.1.fid
f1 (ppm)
19.61
1.00

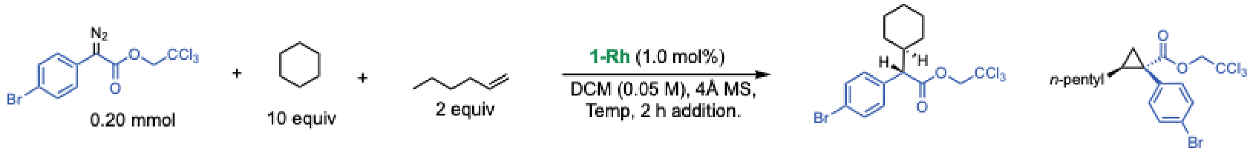
N2
Br
CCl3
O
0.20 mmol
10 equiv
2 equiv
1-Rh (1.0 mol%)
DCM (0.05 M), 4Å MS,
Temp, 2 h addition.
n-pentyl

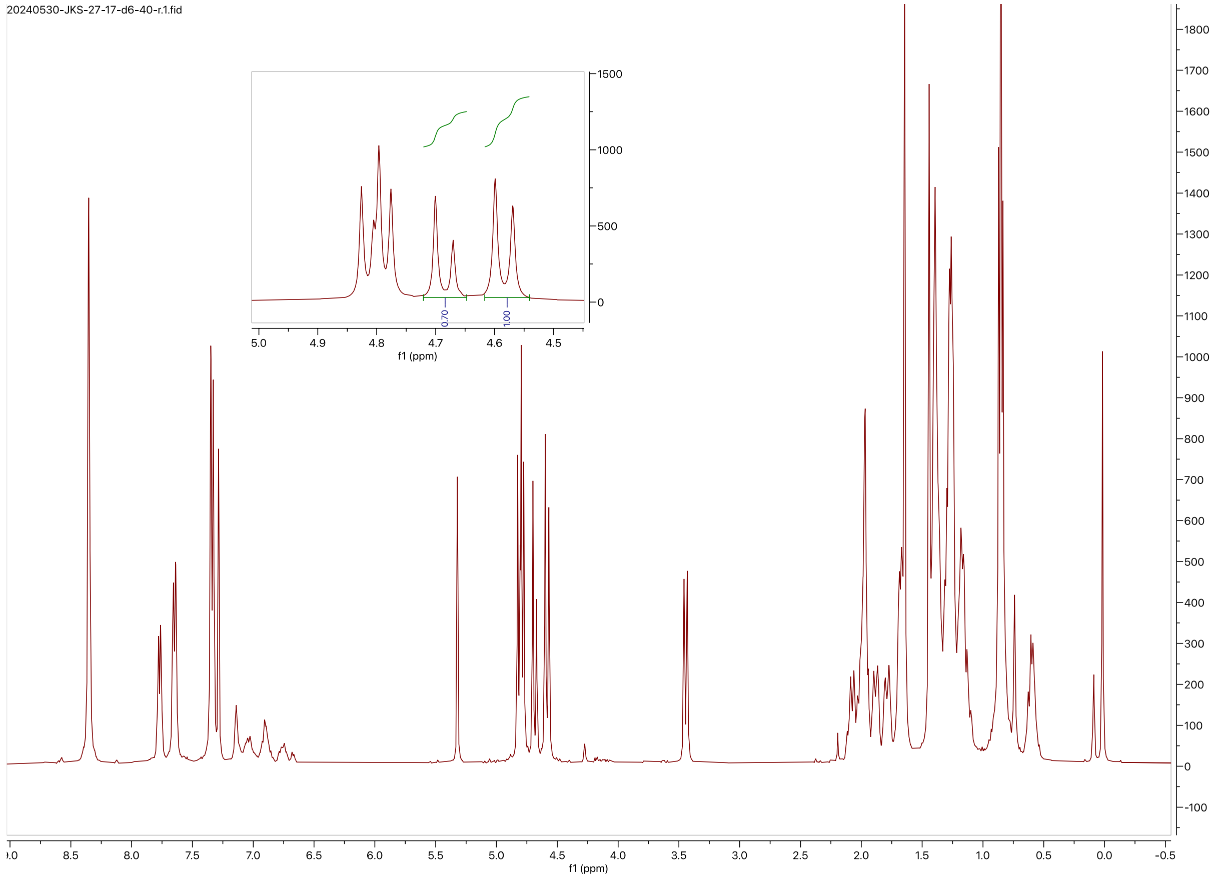
20240530-JKS-27-17-d6-40-r.1.fid
f1 (ppm)
0.70
1.00

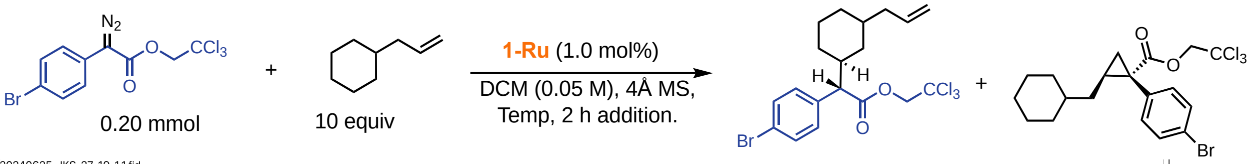
N2
CCl3
Br
0.20 mmol
+
10 equiv
1-Ru (1.0 mol%)
DCM (0.05 M), 4Å MS,
Temp, 2 h addition.
H
H
O
CCl3
Br
+
O
O
CCl3
Br

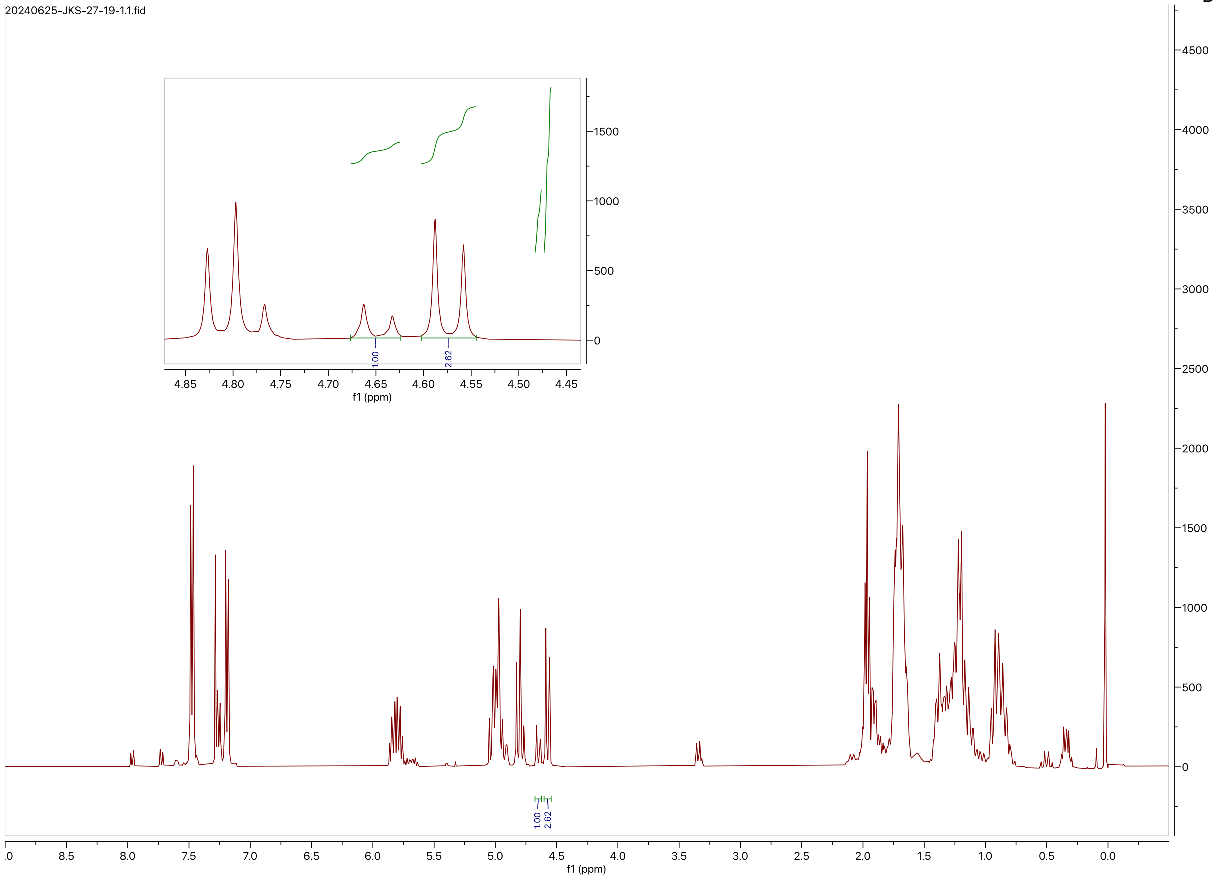
20240625-JKS-27-19-1.1.fid
1.00
2.62
f1 (ppm)

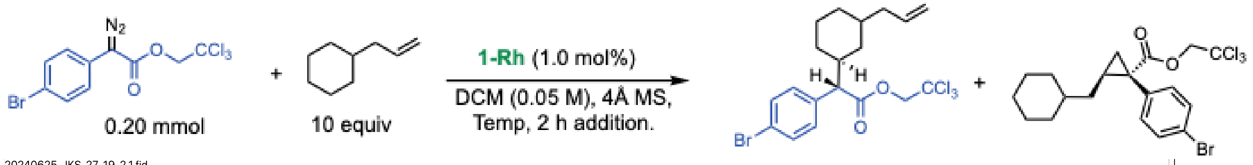
N2
CCl3
Br
0.20 mmol
10 equiv
1-Rh (1.0 mol%)
DCM (0.05 M), 4Å MS,
Temp, 2 h addition.
H
H
O
CCl3
Br
O
O
CCl3
Br

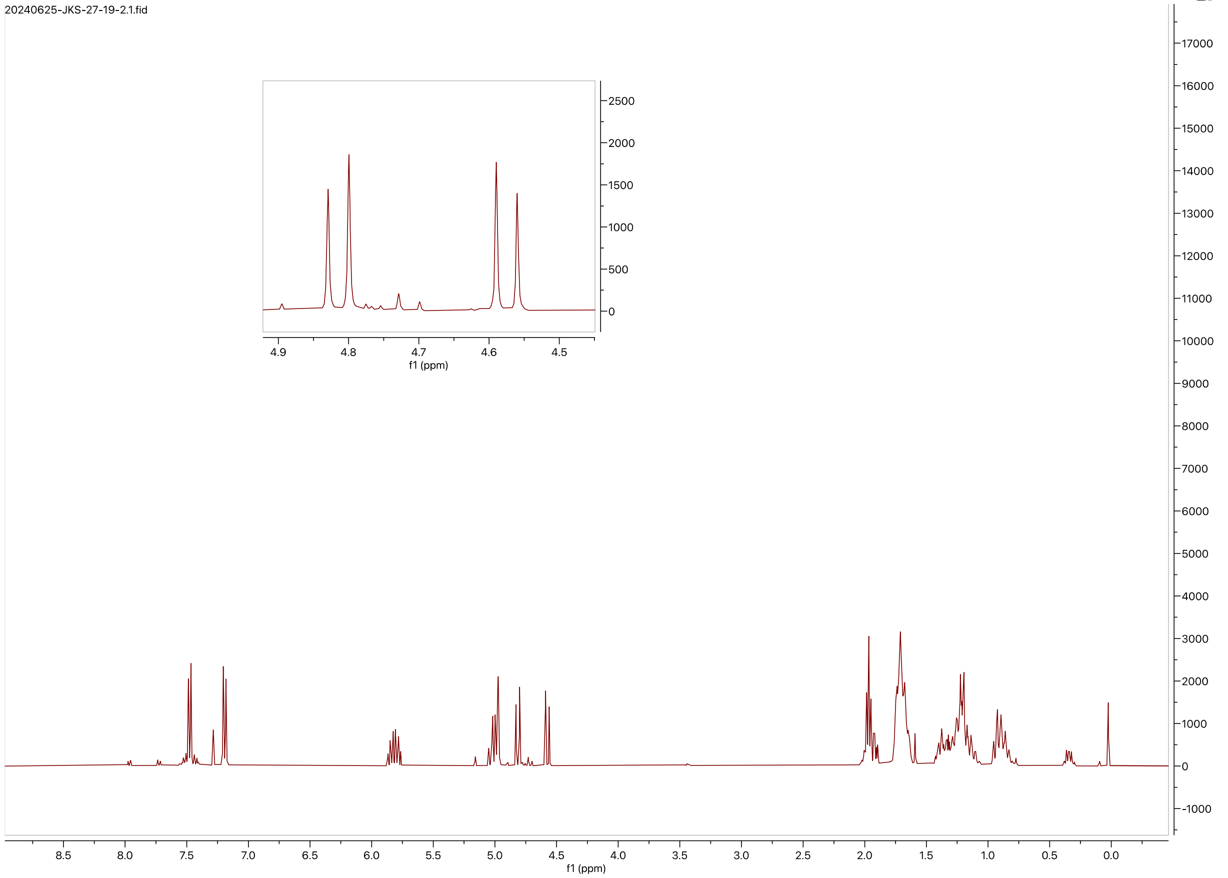
20240625-JKS-27-19-2.1.fid
f1 (ppm)
f1 (ppm)

C–H Insertion Reactions

General Procedure 1
To a flame-dried 16 mL vial equipped with a stir bar and 4 Å MS (1.0 g for 100 mg of diazo) was added catalyst (xx mol %, xx µmol) and substrate (10 equiv, 2.0 mmol). DCM (2 mL) was added to dissolve the sample, and the solution was heated to 40 °C. Then, diazo (1 equiv, 0.200 mmol) was dissolved in DCM (2 mL) and added via syringe pump over the course of 2 h. The solution was left to stir overnight at which point it was stopped, passed over a small plug of celite to remove the mol sieve dust, and concentrated in vacuo for crude NMR analysis.

General Procedure 2
To a flame-dried 16 mL vial equipped with a stir bar and 4 Å MS (1.0 g for 100 mg of diazo) was added catalyst (1.0 mol %, 2.0 µmol) and substrate (5 equiv, 2.0 mmol). DCM (2 mL) was added to dissolve the sample, and the solution was set to stir at 25 °C. Then, diazo (1 equiv, 0.200 mmol) was dissolved in DCM (2 mL) and added via syringe pump over the course of 2 h. The solution was left to stir overnight at which point it was stopped, passed over a small plug of celite to remove the mol sieve dust, and concentrated in vacuo for crude NMR analysis.

General Procedure 3
To a flame-dried 16 mL vial equipped with a stir bar and 4 Å MS (1.0 g for 100 mg of diazo) was added catalyst (1.0 mol%, 2.00 µmol) and substrate (solvent equiv, 2.0 mL). The solution was heated to reflux. Then, diazo (1 equiv, 0.200 mmol) was dissolved in the substrate (2 mL) and added via syringe pump over the course of 2 h. The solution was left to stir overnight at which point it was stopped, passed over a small plug of celite to remove the mol sieve dust, and concentrated in vacuo for crude NMR analysis.

General Procedure 4
To a flame-dried 16 mL vial equipped with a stir bar and 4 Å MS (1.0 g for 100 mg of diazo) was added catalyst (1.0 mol%, 2.00 µmol) and substrate (10 equiv, 2.0 mmol) Trifluorotoluene (TFT) (2 mL) was added to dissolve the sample, and the solution was set to stir at 60 °C. Then, diazo (1 equiv, 0.200 mmol) was dissolved in TFT (2 mL) and added via syringe pump over the course of 2 h. The solution was left to stir overnight at which point it was stopped, passed over a small plug of celite to remove the mol sieve dust, and concentrated in vacuo for crude NMR analysis.

2,2,2-trichloroethyl (R)-2-(4-bromophenyl)-3-(4-isopropylphenyl)propanoate (9)

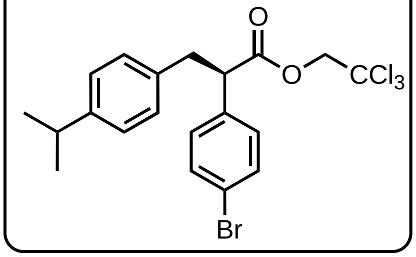

General procedure 1 was employed for the C–H insertion into *p*-cymene (313 µL, 10 equiv, 2.0 mmol), with 2,2,2-trichloroethyl 2-(4-bromophenyl)-2-diazoacetate (74.5 mg, 0.20 mmol, 1.0 equiv) using Ru_2(*S*-PTAD)$_4$BArF (24.2 mg, 5.0 mol%) as catalyst. The crude material was purified via column chromatography (2% diethyl ether/hexanes) to afford the title compound at white crystalline solid (70.1 mg, 73%). Spectra matched literature precedent.[6]

^{1}H NMR (400 MHz, CDCl3) δ 7.45 (d, J = 8.4 Hz, 2H), 7.25 (d, J = 8.5 Hz, 2H), 7.09 (q, J = 8.3 Hz, 4H), 4.69 (d, J = 12.0 Hz, 1H), 4.60 (d, J = 12.0 Hz, 1H), 3.96 (dd, J = 9.1, 6.5 Hz, 1H), 3.40 (dd, J = 13.9, 9.1 Hz, 1H), 3.04 (dd, J = 13.9, 6.6 Hz, 1H), 2.85 (p, J = 6.8 Hz, 1H), 1.21 (d, J = 6.9 Hz, 6H).

Chiral HPLC: The enantiopurity was determined to be 93:7 er by chiral HPLC analysis. (Chiracel AD-H, 1.0% IPA/Hexane, 1.0 mL/min, λ=230 nm, RT: Major: 6.0 min, Minor: 6.6 min.).

2,2,2-trichloroethyl (2S,3S)-2-(4-bromophenyl)-3-(4-isopropylphenyl)butanoate (12a, 12b)

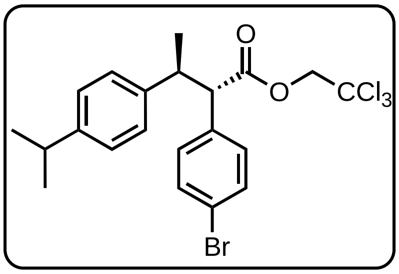

General procedure 1 was employed in the C–H insertion into 1-ethyl-4-isopropylbenzene (148.3 mg, 1.0 mmol, 10 equiv) with 2,2,2-trichloroethyl 2-(4-bromophenyl)-2-diazoacetate (37.2 mg, 0.10 mmol, 1.0 equiv) using Ru_2(*S*-TPPTTL)$_4$BAr^F (16.6 mg, 5.0 mol%) as catalyst. The crude material was purified via prep-TLC (2.5% diethyl ether in hexanes) to afford the title compound as a clear oil (25.5 mg, 52%).
Reported as a 4:1 mixture of diastereomers. Major diastereomer:
^{1}H NMR (400 MHz, $CDCl_3$) δ 7.27 (d, J = 8.5 Hz, 2H), 7.06 (d, J = 8.5 Hz, 2H), 7.00 (d, J = 8.0 Hz, 2H), 6.91 (d, J = 8.0 Hz, 2H), 4.83 (d, J = 12.0 Hz, 1H), 4.68 (d, J = 11.9 Hz, 1H), 3.81 (d, J = 11.0 Hz, 1H), 3.54 – 3.39 (m, 1H), 2.78 (p, J = 6.9 Hz, 1H), 1.42 (d, J = 6.8 Hz, 3H), 1.17 (dd, J = 6.9, 1.1 Hz, 6H).
Minor diastereomer:
^{1}H NMR (400 MHz, $CDCl_3$) δ 7.51 (d, J = 8.4 Hz, 2H), 7.38 (d, J = 8.4 Hz, 2H), 7.23 (d, J = 8.2 Hz, 2H), 7.15 (d, J = 8.0 Hz, 2H), 4.54 (d, J = 11.9 Hz, 1H), 4.29 (d, J = 12.0 Hz, 1H), 3.81 (d, J = 11.0 Hz, 1H), 3.54 – 3.36 (m, 1H), 2.86 (p, J = 7.1 Hz, 1H), 1.22 (d, J = 7.1 Hz, 6H), 1.04 (d, J = 7.0 Hz, 3H).
^{13}C NMR (101 MHz, $CDCl_3$) δ 171.6, 171.1, 147.5, 141.1, 139.9, 135.9, 131.9, 131.3, 130.4, 127.3, 126.7, 126.4, 121.9, 121.3, 94.7, 74.3, 74.0, 58.9, 43.1, 33.7, 33.5, 30.3, 23.9, 21.1, 20.0.
HMRS (-n APCI) calcd for $C_{21}H_{21}O_2{}^{79}Br^{35}Cl_3$ (M–H) 488.9796, found 488.9797.
Chiral HPLC: The enantiopurity for the major diastereomer was determined to be 97:3 er and for the minor diastereomer determined to be 97:3 er by chiral HPLC analysis (Chiracel AD-H, 1.0% IPA/Hexane, 0.50 mL/min. , λ=230 nm, Major diastereomer: RT: 12.3 min. Major, 11.4 min. Minor. Minor diastereomer: RT: 14.0 min, Major. 13.3 min, Minor.).

2,2,2-trichloroethyl (*R*)-2-(4-bromophenyl)-2-cyclohexylacetate (15)

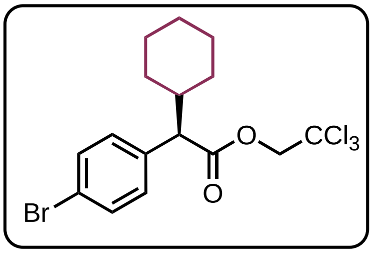

General procedure 2 was used in the C–H insertion into cyclohexane (110 µL, 5 equiv, 1.0 mmol) with 2,2,2-trichloroethyl 2-(4-bromophenyl)-2-diazoacetate (78.5 mg, 1.0 equiv, 0.20 mmol) using Ru_2(*S*-TPPTTL)$_4$BAr^F (6.6 mg, 1.0 mol %) as catalyst. The crude material was then subjected to column chromatography using a 0-2% diethyl ether/hexanes solvent system to afford the title compound as a clear colorless oil. Characterization matched literature reported value.[4]
^{1}H NMR (600 MHz, $CDCl_3$) δ 7.45 (d, J = 8.4 Hz, 2H), 7.24 (d, J = 8.4 Hz, 2H), 4.76 (d, J = 12.0 Hz, 1H), 4.63 (d, J = 12.0 Hz, 1H), 3.35 (d, J = 10.6 Hz, 1H), 2.05 (qt, J = 11.1, 3.4 Hz, 1H), 1.92 – 1.82 (m, 1H), 1.75 (ddt, J = 11.6, 3.6, 1.8 Hz, 1H), 1.64 (dddd, J = 9.2, 7.6, 3.5, 2.1 Hz, 2H), 1.38 – 1.34 (m, 1H), 1.34 – 1.26 (m, 1H), 1.19 – 1.06 (m, 3H), 0.83 – 0.73 (m, 1H).
Chiral HPLC: The enantiopurity was determined to be 97.5:2.5 er by chiral HPLC analysis (Chiracel AD-H, 0.1% IPA/Hexane, 1.0 mL/min., λ=230 nm, RT: Major: 15.6 min., Minor: 8.9 min.)

2,2,2-trichloroethyl (R)-2-(4-bromophenyl)-2-cyclopentylacetate (16)

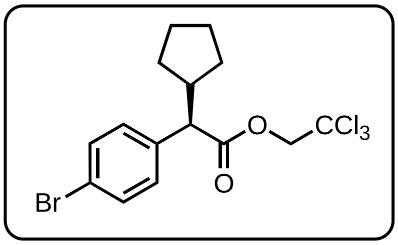

General procedure 2 was used for the C–H insertion into cyclopentane (90 µL, 1.0 mmol, 5.0 equiv) with 2,2,2-trichloroethyl 2-(4-bromophenyl)-2-diazoacetate (74.5 mg, 0.20 mmol, 1.0 equiv) using Ru_2(*S*-TPPTTL)$_4$BAr^F (6.6 mg, 1.0 mol%) as catalyst. Purification by column chromatography (2% diethyl ether/hexanes) afforded an oil (58 mg, 70%). Spectrum matched literature precedent.[4]

^{1}H NMR (400 MHz, $CDCl_3$) δ 7.47 (d, J = 8.0 Hz, 2H), 7.29 (d, J = 7.9 Hz, 2H), 4.79 (d, J = 11.9 Hz, 1H), 4.67 (d, J = 12.0 Hz, 1H), 3.41 (d, J = 11.2 Hz, 1H), 2.62 (q, J = 8.7 Hz, 1H), 1.98 (tt, J = 12.9, 5.4 Hz, 1H), 1.75 – 1.57 (m, 3H), 1.51 (dq, J = 12.3, 6.4 Hz, 2H), 1.40 – 1.23 (m, 1H), 1.10 – 0.94 (m, 1H).

Chiral HPLC: The enantiopurity was determined to be 96% ee by chiral HPLC analysis (Chiracel AD-H, 0.5% IPA/Hexane, 1.0 mL/min., λ=230 nm, RT: Major: 7.5 min., Minor: 6.7)

2,2,2-trichloroethyl (*R*)-2-(4-bromophenyl)-2-cycloheptylacetate (17)

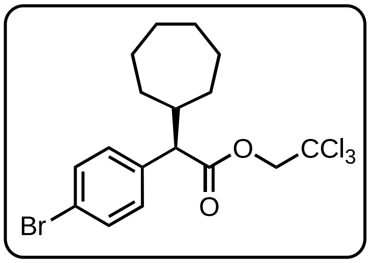

General procedure 2 was used for the C–H insertion into cycloheptane (120 µL, 1.0 mmol, 5.0 equiv) with 2,2,2-trichloroethyl 2-(4-bromophenyl)-2-diazoacetate (74.5 mg, 0.20 mmol, 1.0 equiv) using Ru_2(*S*-TPPTTL)$_4$BAr^F (6.6 mg, 1.0 mol%) as catalyst. Purification by column chromatography (2% diethyl ether/hexanes) afforded an oil (66 mg, 75%). Spectrum matched literature precedent.[4]

^{1}H NMR (400 MHz, $CDCl_3$) δ 7.47 (d, J = 8.5 Hz, 2H), 7.27 (d, J = 8.4 Hz, 2H), 4.76 (d, J = 12.0 Hz, 1H), 4.66 (d, J = 12.0 Hz, 1H), 3.45 (d, J = 11.0 Hz, 1H), 2.32 (dtt, J = 11.0, 9.4, 3.8 Hz, 1H), 1.91 – 1.79 (m, 1H), 1.72 (ddt, J = 13.1, 9.4, 4.9 Hz, 1H), 1.67 – 1.46 (m, 6H), 1.46 – 1.27 (m, 3H), 1.03 (dtd, J = 13.6, 9.5, 2.6 Hz, 1H).

Chiral HPLC: The enantiopurity was determined to be 92% ee by HPLC analysis (S,S-Whelk 0.5% IPA/Hexane, 0.50 mL/min, λ=230 nm, RT: Major: 19.9 min., Minor: 23.0 min.).

2,2,2-trichloroethyl (*R*)-2-(4-bromophenyl)-2-cyclooctylacetate (18)

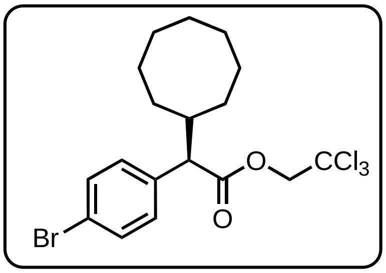

General procedure 2 was used for the C–H insertion into cyclooctane (135 µL, 1.0 mmol, 5.0 equiv) with 2,2,2-trichloroethyl 2-(4-bromophenyl)-2-diazoacetate (74.5 mg, 0.20 mmol, 1.0 equiv) using Ru_2(*S*-TPPTTL)$_4$BAr^F (6.6 mg, 1.0 mol%) as catalyst. Purification by column chromatography (2% diethyl ether/hexanes) afforded the product as a crystalline solid (66 mg, 75%).

^{1}H NMR (400 MHz, $CDCl_3$) δ 7.45 (d, J = 8.4 Hz, 2H), 7.26 (d, J = 8.4 Hz, 2H), 4.75 (d, J = 12.0 Hz, 1H), 4.62 (d, J = 12.0 Hz, 1H), 3.41 (d, J = 11.1 Hz, 1H), 2.37 (tdd, J = 11.6, 6.8, 2.5 Hz, 1H), 1.73 (d, J = 10.8 Hz, 2H), 1.67 – 1.38 (m, 9H), 1.36 – 1.21 (m, 2H), 1.09 (dtd, J = 15.7, 9.4, 3.6 Hz, 1H).

^{13}C NMR (101 MHz, $CDCl_3$) δ 172.0, 136.5, 131.7, 130.6, 121.6, 94.8, 74.2, 58.5, 40.1, 31.1, 29.1, 26.9, 26.9, 26.4, 25.4, 25.1.

HRMS (+p APCI) calcd for $C_{18}H_{23}O_2BrCl_3$ (M+H) 454.9941, found 454.9940.

IR (neat): 2920, 1748, 1488, 1446, 1407, 1370, 1273, 1216, 1120, 1073, 1011, 829, 758, 718, 572, 505 (cm^{-1}).
Chiral HPLC: The enantiopurity was determined to be 90% ee by HPLC analysis (Chiracel AD-H 1.0 IPA/Hexane, 1.0 mL/min, λ=230 nm, RT: Major: 19.9 min., Minor: 23.0 min.).

2,2,2-trichloroethyl (*R*)-2-((3*R*,5*R*,7*R*)-adamantan-1-yl)-2-(4-bromophenyl)acetate (19)

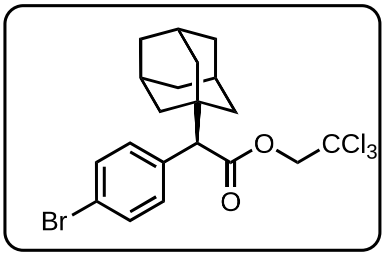

General procedure 2 was used for the C–H insertion into adamantane (136 mg, 1.0 mmol, 5.0 equiv) with 2,2,2-trichloroethyl 2-(4-bromophenyl)-2-diazoacetate (74.5 mg, 0.20 mmol, 1.0 equiv) using Ru_2(*S*-TPPTTL)$_4$BAr^F (6.6 mg, 1.0 mol%) as catalyst. Purification by column chromatography (2% diethyl ether/hexanes) afforded a clear colorless oil (64 mg, 66%).Spectrum matched literature precedent.[4]
^{1}H NMR (400 MHz, $CDCl_3$) δ 7.46 (d, J = 8.5 Hz, 2H), 7.30 (d, J = 8.5 Hz, 2H), 4.83 (d, J = 12.0 Hz, 1H), 4.64 (d, J = 12.0 Hz, 1H), 3.41 (s, 1H), 1.99 (t, J = 3.2 Hz, 3H), 1.77 – 1.64 (m, 6H), 1.62 – 1.53 (m, 6H).
Chiral HPLC: The enantiopurity was determined to be 92% ee by HPLC analysis (S,S-Whelk, 1.0% IPA/Hexane, 1.0 mL/min, λ=230 nm, RT: Major: 6.4 min., Minor: 7.1 min.).

2,2,2-trichloroethyl (*R*)-2-(4-bromophenyl)-2-((1*R*,3*S*)-3-(*tert*-butyl)cyclohexyl)acetate (20)

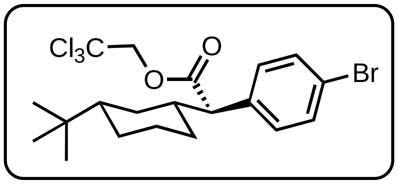

General procedure 2 was used for the C–H insertion of *t*-butylcyclohexane (169 µL, 1.0 mmol, 5 equiv) with 2,2,2-trichloroethyl 2-(4-bromophenyl)-2-diazoacetate (74.5 mg, 0.20 mmol, 1.0 equiv) using Ru_2(*S*-TPPTTL)$_4$BAr^F (6.6 mg, 1.0 mol%) as catalyst. Purification by column chromatography (2% diethyl ether/hexanes) afforded an oil (87 mg, 90%). Spectra matched literature precedent.[4]
^{1}H NMR (400 MHz, $CDCl_3$) δ 7.45 (d, J = 8.4 Hz, 2H), 7.24 (d, J = 8.5 Hz, 2H), 4.76 (d, J = 11.9 Hz, 1H), 4.62 (d, J = 12.0 Hz, 1H), 3.36 (d, J = 10.3 Hz, 1H), 2.12 – 1.99 (m, 1H), 1.94 – 1.79 (m, 2H), 1.79 – 1.68 (m, 1H), 1.46 (dt, J = 12.6, 2.7 Hz, 1H), 1.28 (tdd, J = 12.7, 9.2, 3.5 Hz, 1H), 1.06 – 0.90 (m, 2H), 0.90 – 0.78 (m, 1H), 0.72 (s, 9H), 0.50 (q, J = 12.0 Hz, 1H).
Chiral HPLC: The enantiopurity for the major diastereomer was determined to be 94% ee by HPLC analysis (Chiracel AD-H, 2.0% IPA/Hexane, 1.0 mL/min, λ=230 nm, RT Major Diastereomer: Major: 16.5 min, Minor: 14.5 min. Minor Diastereomer: Major: 18.8 min. Minor: 21.1 min.

2,2,2-trichloroethyl (2*R*,3*S*)-2-(4-bromophenyl)-3-methylhexanoate (21)

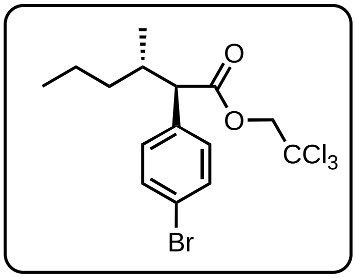

General procedure 3 was used for the C–H functionalization of pentane (2.0 mL) with 2,2,2-trichloroethyl 2-(4-bromophenyl)-2-diazoacetate (74.5 mg, 0.20 mmol, 1.0 equiv) using Ru_2(*S*-TPPTTL)$_4$BAr^F (6.6 mg, 1.0 mol%) as catalyst. Purification by column chromatography (2% diethyl ether/hexanes) afforded the title compound as a mixture of diastereomers (73 mg, 88%). Spectra matched literature precedent.[5]

^{1}H NMR (400 MHz, CDCl$_3$) δ 7.45 (d, J = 8.5 Hz, 2H), 7.24 (d, J = 8.5 Hz, 2H), 4.77 (d, J = 12.0 Hz, 1H), 4.63 (d, J = 12.0 Hz, 1H), 3.37 (d, J = 10.6 Hz, 1H), 2.24 (dddd, J = 17.2, 13.1, 6.4, 3.0 Hz, 1H), 1.51 – 1.45 (m, 1H), 1.41 – 1.27 (m, 1H), 1.25 – 1.07 (m, 1H), 1.05 (d, J = 6.5 Hz, 3H), 0.96 – 0.82 (m, 1H), 0.77 (t, J = 7.2 Hz, 3H).
Chiral HPLC: The enantiopurity of the major diastereomer was determined to be 95:5 er, and the minor diastereomer to be 90% er by chiral HPLC analysis. (S,*S*-Whelk, 1.0% IPA/Hexane, 1.0 mL/min, λ=230 nm, Major diastereomer: Major: 69.5 min., Minor: 40.1 min. Minor diastereomer: Major: 75.4 min., Minor: 44.6 min.).

2,2,2-trichloroethyl (2R,3R,E)-2-(4-bromophenyl)-3-methylhex-4-enoate (22)

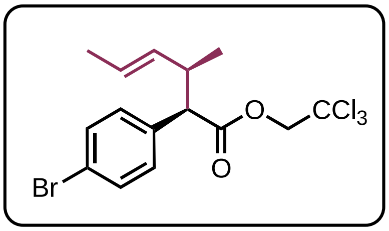

General procedure 1 was used for the C–H functionalization of (*E*)-hex-2-ene (0.25 mL, 2.0 mmol, 10 equiv) with 2,2,2-trichloroethyl 2-(4-bromophenyl)-2-diazoacetate (74.5 mg, 0.20 mmol, 1.0 equiv) using Ru_2(*S*-TPPTTL)$_4$BAr^F (6.6 mg, 1.0 mol%). Purification using 0-3% diethyl ether/hexanes column afforded the title compound as a mixture of diastereomers as a clear, colorless oil (71 mg, 83%).

Reported as a mixture of diastereomers.
^{1}H NMR (400 MHz, CDCl$_3$) δ 7.45 (d, J = 8.5 Hz, 0.75H), 7.43 – 7.38 (m, 2H), 7.28 (d, J = 8.5 Hz, 0.62H), 7.17 (d, J = 8.5 Hz, 2H), 5.59 (dq, J = 15.3, 6.4 Hz, 0.3H), 5.35 – 5.14 (m, 1.4H), 4.88 (ddd, J = 15.2, 9.3, 1.7 Hz, 1H), 4.75 (d, J = 12.0 Hz, 1H), 4.71 (d, J = 11.9 Hz, 0.4H), 4.65 (d, J = 12.0 Hz, 1H), 4.53 (d, J = 12.0 Hz, 0.3H), 3.54 (d, J = 10.3 Hz, 1H), 3.50 (d, J = 11.0 Hz, 0.4H), 2.63 (tdd, J = 15.8, 10.0, 3.2 Hz, 1.4H), 1.66 (dd, J = 6.4, 1.7 Hz, 1H), 1.63 – 1.50 (m, 1.8H), 1.48 (dd, J = 6.4, 1.7 Hz, 3H), 1.39 – 1.12 (m, 2H), 0.89 (t, J = 7.3 Hz, 3H), 0.75 (t, J = 7.3 Hz, 1H).
^{13}C NMR (101 MHz, CDCl$_3$) δ 171.6, 171.2, 135.9, 135.7, 131.7, 131.4, 131.3, 130.8, 130.6, 130.3, 128.7, 128.6, 121.7, 121.4, 94.7, 74.3, 74.2, 56.8, 47.9, 26.5, 24.8, 18.1, 17.9, 11.6, 11.3.
HRMS (-n APCI) calcd for $C_{16}H_{17}O_2{}^{79}Br^{35}Cl_3$ [M–H] 424.9483, found 424.9479.
Chiral HPLC: The enantiopurity of the major diastereomer was determined to be 46% ee by chiral HPLC analysis.. (Chiracel AD-H, 0.5% IPA/Hexane, 1.0 mL/min, λ=230 nm, Major diastereomer: Major: 5.6 min., Minor: 4.8 min. Minor diastereomer: Major: 6.5 min., Minor: 5.2 min.).

2,2,2-trichloroethyl (S)-2-(4-bromophenyl)-2-((R)-tetrahydrofuran-2-yl)acetate (23)

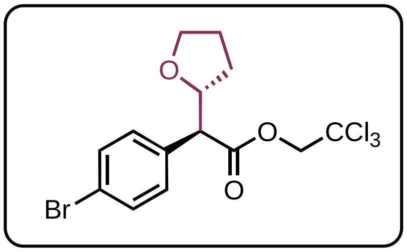

General procedure 4 was used for the C–H functionalization of tetrhydrofuran (0.16 mL, 2.0 mmol, 10 equiv) with 2,2,2-trichloroethyl 2-(4-bromophenyl)-2-diazoacetate (74.5 mg, 0.20 mmol, 1.0 equiv) using Ru_2(*S*-TPPTTL)$_4$BAr^F (6.6 mg, 1.0 mol%). Purification using 0-10% diethyl ether/hexanes afforded the title compound as a mixture of diastereomers as a clear colorless oil (47 mg, 56%).

Reported as a mixture of diastereomers:
^{1}H NMR (400 MHz, CDCl$_3$) δ 7.49 (dd, J = 8.5, 3.6 Hz, 2.8H), 7.36 – 7.26 (m, 3.1H), 4.83 – 4.77 (m, 1.8H), 4.72 (d, J = 12.0 Hz, 1.3H), 4.54 (ddt, J = 15.5, 8.5, 6.9 Hz, 1.5H), 3.95 (dt, J = 8.4, 6.8 Hz, 0.5H), 3.91 – 3.79 (m, 1.5H), 3.79 – 3.70 (m, 2H), 3.66 (d, J = 9.9 Hz, 0.5H), 2.25 – 2.13 (m, 1H), 1.92 (dddd, J = 12.8, 8.2, 6.4, 4.6 Hz, 2.8H), 1.84 – 1.75 (m, 0.6H), 1.64 – 1.58 (m, 1H), 1.56 – 1.41 (m, 0.5H).
^{13}C NMR (101 MHz, CDCl$_3$) δ 170.4, 170.0, 134.6, 134.0, 132.0, 131.7, 131.5, 130.9, 130.6, 130.3, 122.2, 122.0, 94.7, 94.7, 80.1, 79.4, 74.2, 74.1, 68.6, 68.5, 56.9, 56.3, 30.3, 29.5, 25.7, 25.4.
HRMS (+p APCI) calcd for $C_{14}H_{13}O_3{}^{79}Br^{35}Cl_3$ [M–H] 412.9119, found 412.9117.
Chiral HPLC: The enantiopurity of the major diastereomer was determined to be 94% ee, and the minor diastereomer to be 94% ee by chiral HPLC analysis. (Chiracel AD-H, 0.5% IPA/Hexane, 1.0 mL/min, λ=230 nm, Major diastereomer: Major: 16.9 min., Minor: 15.1 min. Minor diastereomer: Major: 26.7 min., Minor: 21.6 min.).

tert-butyl (R)-2-((S)-1-(4-bromophenyl)-2-oxo-2-(2,2,2-trichloroethoxy)ethyl)pyrrolidine-1-carboxylate (24)

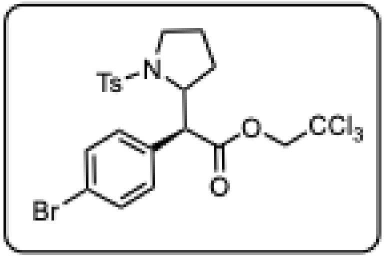

General procedure 2 was used for the C–H functionalization of N-Tosyl-pyrrolidine (67 mg, 0.3 mmol, 1.5 equiv) with 2,2-trichloroethyl 2-(4-bromophenyl)-2-diazoacetate (74.5 mg, 0.20 mmol, 1.0 equiv) using Rh_2(*S*-TPPTTL)$_4$ (4.9 mg, 1.0 mol%) as catalyst. The crude material was purified using 20% diethyl ether/hexanes column to afford a white powder (72.5 mg, 64%)

^{1}H NMR (400 MHz, CDCl$_3$) δ 7.62 (d, J = 8.3 Hz, 2H), 7.46 (d, J = 8.4 Hz, 2H), 7.28 (dd, J = 8.6, 6.8 Hz, 4H), 4.84 (d, J = 12.0 Hz, 1H), 4.75 (d, J = 12.0 Hz, 1H), 4.34 – 4.24 (m, 1H), 4.16 (d, J = 6.0 Hz, 1H), 3.40 (ddd, J = 12.2, 7.5, 5.1 Hz, 1H), 3.27 – 3.14 (m, 1H), 2.42 (s, 3H), 2.10 – 1.93 (m, 1H), 1.71 – 1.55 (m, 2H), 1.33 (dt, J = 12.3, 7.4 Hz, 1H).
^{13}C NMR (101 MHz, CDCl$_3$) δ 169.9, 143.7, 134.8, 133.6, 131.7, 131.1, 129.8, 127.6, 122.2, 94.7, 74.4, 62.7, 55.0, 49.3, 29.3, 24.2, 21.6.
HRMS (+p APCI) calcd for $C_{21}H_{22}O_4N^{79}Br^{35}Cl_3{}^{32}S$ [M+H] 567.9513, found 567.9507.
Chiral SFC: The enantiopurity was determined to be 90% ee by chiral SFC analysis. (OJ-3, 5% MeOH/IPA + 0.2% Formic Acid. 5 min, 2.5 mL/min. λ=230 nm, RT: Major: 3.55 min. Minor: 3.83 min.)

2,2,2-trichloroethyl (*R*)-2-cyclohexyl-2-(4-(((trifluoromethyl)sulfonyl)oxy)phenyl)acetate (25)

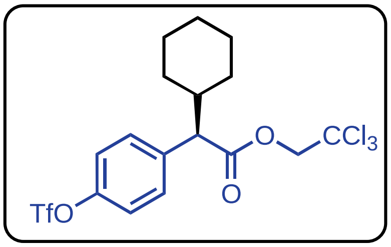

General procedure 2 was used in the C–H functionalization of cyclohexane (0.11 mL, 1.0 mmol, 5.0 equiv) with 2,2,2-trichloroethyl 2-diazo-2-(4-(((trifluoromethyl)sulfonyl)oxy)phenyl)acetate (88.3 mg, 0.20 mmol, 1.0 equiv) with Ru_2(*S*-TPPTTL)$_4$BAr^F (6.6 mg, 1.0 mol%) as catalyst. The product was purified via column chromatography (5% diethyl ether/hexanes) to afford a crystalline solid (81.5 mg, 82%). Spectra matched literature precedent.[2]

^{1}H NMR (400 MHz, $CDCl_3$) δ 7.46 (d, J = 8.8 Hz, 2H), 7.24 (d, J = 8.8 Hz, 2H), 4.77 (d, J = 11.9 Hz, 1H), 4.65 (d, J = 12.0 Hz, 1H), 3.43 (d, J = 10.6 Hz, 1H), 2.07 (qt, J = 11.0, 3.4 Hz, 1H), 1.87 (dt, J = 12.5, 3.3 Hz, 1H), 1.81 – 1.71 (m, 1H), 1.71 – 1.62 (m, 2H), 1.37 – 1.25 (m, 2H), 1.21 – 1.05 (m, 3H), 0.85 – 0.70 (m, 1H).
Chiral HPLC: The enantiopurity was determined to be 86% ee by chiral HPLC analysis (Chiracel AD-H, 1.0% IPA/Hexanes, 1.0 mL/min, λ=230 nm, RT: 6.5 Major, 5.6 Minor).

2,2,2-trichloroethyl (*R*)-2-cyclohexyl-2-(4-fluorophenyl)acetate (26)

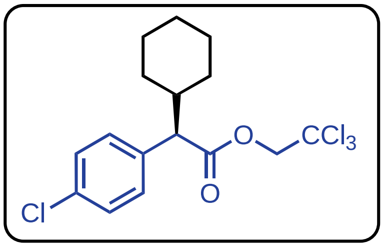

General procedure 2 was used for the C–H functionalization of cyclohexane (0.11 mL, 1.0 mmol, 5.0 equiv) with 2,2,2-trichloroethyl 2-(4-chlorophenyl)-2-diazoacetate (65.8 mg, 0.20 mmol, 1.0 equiv) using Ru_2(*S*-TPPTTL)$_4$BAr^F (6.6 mg, 1.0 mol%) as catalyst. Purification by column chromatography (2% diethyl ether/hexanes) afforded an oil (61 mg, 79%). Spectra matched literature precedent.[2]
^{1}H NMR (400 MHz, $CDCl_3$) δ 7.32 (s, 4H), 4.78 (d, J = 12.0 Hz, 1H), 4.65 (d, J = 12.0 Hz, 1H), 3.38 (d, J = 10.7 Hz, 1H), 2.07 (qt, J = 11.0, 3.4 Hz, 1H), 1.88 (dt, J = 12.6, 3.5 Hz, 1H), 1.84 – 1.74 (m, 1H), 1.74 – 1.59 (m, 2H), 1.47 – 1.25 (m, 2H), 1.25 – 1.03 (m, 3H), 0.79 (qd, J = 12.1, 3.5 Hz, 1H).
Chiral HPLC: The enantiopurity was determined to be 96% ee by chiral HPLC analysis (Chiracel AD-H, 0.5% IPA/Hexane, 0.5 mL/min, , λ=230 nm, RT: Major: 16.7 min, Minor: 11.7 min)

2,2,2-trichloroethyl (*R*)-2-cyclohexyl-2-(4-(trifluoromethyl)phenyl)acetate (27)

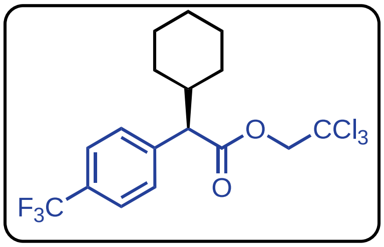

General procedure 2 was used for the C–H functionalization of cyclohexane (0.11 mL, 1.0 mmol, 5.0 equiv) with 2,2,2-trichloroethyl 2-diazo-2-(4-(trifluoromethyl)phenyl)acetate (72.3 mg, 0.20 mmol, 1.0 equiv) using Ru_2(*S*-TPPTTL)$_4$BAr^F (6.6 mg, 1.0 mol%) as catalyst. Purification by column chromatography (2% diethyl ether/hexanes) afforded an oil (59 mg, 70%). Spectra matched literature precedent.[2]
^{1}H NMR (400 MHz, $CDCl_3$) δ 7.61 (d, J = 8.0 Hz, 2H), 7.52 (d, J = 8.1 Hz, 2H), 4.80 (d, J = 12.0 Hz, 1H), 4.66 (d, J = 12.0 Hz, 1H), 3.49 (d, J = 10.7 Hz, 1H), 2.14 (qt, J = 11.0, 3.4 Hz, 1H), 1.95 – 1.87 (m, 1H), 1.83 – 1.74 (m, 1H), 1.71 – 1.60 (m, 2H), 1.39 – 1.27 (m, 2H), 1.25 – 1.08 (m, 3H), 0.81 (qd, J = 12.1, 3.5 Hz, 1H).
Chiral HPLC: The enantiopurity was determined to be 90% ee by chiral HPLC analysis (Chiracel AD-H, 0.1% IPA/Hexanes, 1.0 mL/min, λ=210 nm, RT: Major: 11.5 min, Minor: 6.8 min)

2,2,2-trichloroethyl (R)-2-(6-chloropyridin-3-yl)-2-cyclohexylacetate (28)

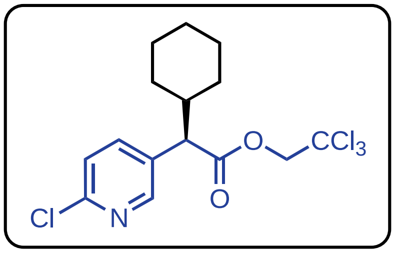

General procedure 3 was used for the C–H functionalization of cycloehxane (0.11 mL, 1.0 mmol, 5.0 equiv) with 2,2,2-trichloroethyl 2-(6-chloropyridin-3-yl)-2-diazoacetate (66 mg, 0.20 mmol, 1.0 equiv) using Ru_2(*S*-TPPTTL)$_4$BAr^F (6.6 mg, 1.0 mol%) as catalyst. Purification by column chromatography (10% diethyl ether/hexanes) afforded an oil (43.7 mg, 58%). Spectra matched literature precedent.[7]

^{1}H NMR (400 MHz, $CDCl_3$) δ 8.32 (d, J = 2.5 Hz, 1H), 7.74 (dd, J = 8.3, 2.5 Hz, 1H), 7.31 (d, J = 8.3 Hz, 1H), 4.76 (d, J = 12.0 Hz, 1H), 4.66 (d, J = 12.0 Hz, 1H), 3.42 (d, J = 10.4 Hz, 1H), 2.14 – 1.94 (m, 1H), 1.92 – 1.72 (m, 1H), 1.71 – 1.57 (m, 2H), 1.39 – 1.22 (m, 2H), 1.14 (ddt, J = 15.0, 11.3, 4.7 Hz, 2H), 0.81 (dt, J = 12.8, 5.0 Hz, 1H).
Chiral HPLC: The enantiopurity was determined to be 96% ee by chiral HPLC analysis (R,R-Whelk, 1% IPA/Hexanes, 0.5 mL/min, λ=230 nm, RT: Major: 21.9 min, Minor: 19.7 min)

2,2,2-trichloroethyl (*R*)-2-([1,1'-biphenyl]-4-yl)-2-cyclohexylacetate (29)

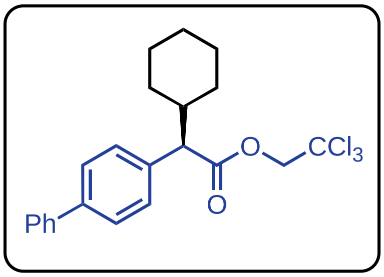

General procedure 3 was used for the C–H functionalization of cyclohexane (4.0 mL) with 2,2,2-trichloroethyl 2-diazo-2-(4-fluorophenyl)acetate (62.3 mg, 0.20 mmol, 1.0 equiv) using Ru_2(*S*-TPPTTL)$_4$BAr^F (6.6 mg, 1.0 mol%) as catalyst. Purification by column chromatography (2% diethyl ether/hexanes) afforded a white solid (63 mg, 74%). Spectra matched literature precedent.[2]

^{1}H NMR (400 MHz, $CDCl_3$) δ 7.63 – 7.53 (m, 2H), 7.50 – 7.41 (m, 2H), 7.40 – 7.31 (m, 1H), 4.82 (d, J = 12.0 Hz, 1H), 4.64 (d, J = 12.0 Hz, 1H), 3.45 (d, J = 10.7 Hz, 1H), 2.16 (qt, J = 11.1, 3.4 Hz, 1H), 1.93 (dt, J = 12.5, 3.2 Hz, 1H), 1.82 – 1.73 (m, 1H), 1.71 – 1.62 (m, 2H), 1.47 (dt, J = 11.5, 2.7 Hz, 1H), 1.40 – 1.30 (m, 1H), 1.23 – 1.13 (m, 3H), 0.85 (pd, J = 10.7, 3.9 Hz, 1H).
Chiral HPLC: The enantiopurity was determined to be 92% ee by chiral HPLC analysis (S,S-Whelk, 1.0% IPA/Hexanes, 1.0 mL/min, Major: 13.0 min. Minor: 14.5 min.).

2,2,2-trichloroethyl (*R*)-2-(4-(*tert*-butyl)phenyl)-2-cyclohexylacetate (30)

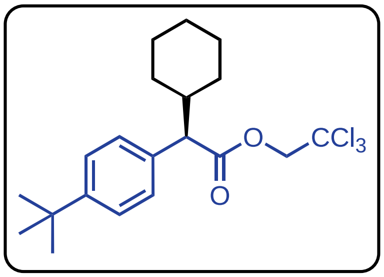

General procedure 3 was used for the C–H functionalization of cyclohexane (4.0 mL) with 2,2,2-trichloroethyl 2-diazo-2-(4-fluorophenyl)acetate (62.3 mg, 0.20 mmol, 1.0 equiv) using Ru_2(*S*-TPPTTL)$_4$BAr^F (6.6 mg, 1.0 mol%) as catalyst. Purification by column chromatography (2% diethyl ether/hexanes) afforded an oil (53 mg, 73%). Spectra matched literature precedent.[7]

^{1}H NMR (400 MHz, $CDCl_3$) δ 7.37 – 7.33 (m, 2H), 7.30 (d, J = 9.2 Hz, 2H), 4.81 (d, J = 12.0 Hz, 1H), 4.60 (d, J = 12.0 Hz, 1H), 3.38 (d, J = 10.8 Hz, 1H), 2.10 (qt, J = 11.0, 3.4 Hz, 1H), 1.90 (d, J = 12.7 Hz, 1H), 1.81 – 1.72 (m, 1H), 1.66 (dd, J = 9.7, 5.0 Hz, 2H), 1.45 (m, 2H), 1.33 (s, 9H), 1.24 – 1.08 (m, 3H), 0.85 – 0.74 (m, 1H).
Chiral HPLC: The enantiopurity was determined to be 90% ee by chiral HPLC analysis (S,S-Whelk, 0.1% IPA/ Hexane, 0.5 mL/min. Major: 21.7 min., Minor: 18.5 min.).

2,2,2-trichloroethyl (*R*)-2-cyclohexyl-2-(4-methoxyphenyl)acetate (31)

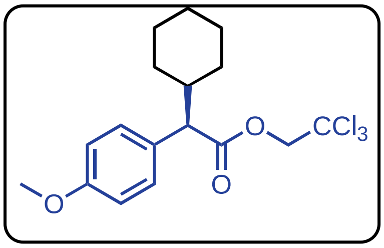

General procedure 3 was used for the C–H functionalization of cyclohexane (4.0 mL) with 2,2,2-trichloroethyl 2-diazo-2-(4-methoxyphenyl)acetate (64.7 mg, 0.200 mmol, 1.0 equiv) using Ru_2(*S*-TPPTTL)$_4$BAr^F (6.6 mg, 1.0 mol%) as catalyst. Purification by column chromatography (2% diethyl ether/hexanes) afforded a white solid (38.1 mg, 51%). Spectra matched literature precedent.[7]

^{1}H NMR (400 MHz, CDCl$_3$) δ 7.26 (d, J = 8.7 Hz, 2H), 6.85 (d, J = 8.7 Hz, 2H), 4.76 (d, J = 12.0 Hz, 1H), 4.61 (d, J = 12.0 Hz, 1H), 3.79 (s, 3H), 3.32 (d, J = 10.7 Hz, 1H), 2.04 (dddd, J = 14.4, 11.1, 7.3, 3.4 Hz, 1H), 1.86 (d, J = 12.7 Hz, 1H), 1.75 (d, J = 13.5 Hz, 1H), 1.67 – 1.57 (m, 2H), 1.39 (d, J = 15.0 Hz, 1H), 1.34 – 1.24 (m, 1H), 1.21 – 1.02 (m, 3H), 0.82 – 0.69 (m, 1H).
Chiral HPLC: The enantiopurity was determined to be 28% ee by chiral HPLC analysis (Chiracel AD-H, 1.0% IPA/Hexane, 1.0 mL/min., λ=230 nm, RT: Major: 13.2 min., Minor: 14.4 min.).

2,2,2-trichloroethyl (R)-2-cyclohexyl-2-(naphthalen-2-yl)acetate (32)

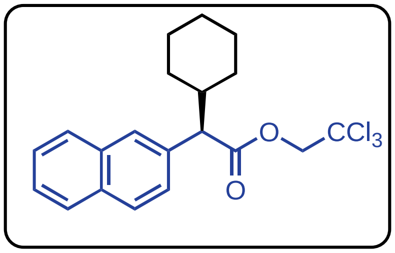

General procedure 3 was used for the reaction of cyclohexene (200 µL, 5 equiv, 2.0 mmol) with, 2,2,2-trichloroethyl (R)-2-cyclohexyl-2-(naphthalen-2-yl)acetate (69 mg, 0.20 mmol, 1.0 equiv) using Ru_2(*S*-TPPTTL)$_4$BAr^F (6.6 mg, 1.0 mol%) as catalyst. Purification by column chormatogarphy (2% diethyl ether/hexanes) afforded a clear colorless oil (60.2 mg). Spectra matched literature precedent.[2]

^{1}H NMR (400 MHz, CDCl$_3$) δ 7.84 – 7.79 (m, 4H), 7.53 (dd, J = 8.7, 1.7 Hz, 1H), 7.50 – 7.43 (m, 2H), 4.81 (d, J = 12.0 Hz, 1H), 4.62 (d, J = 12.0 Hz, 1H), 3.56 (d, J = 10.7 Hz, 1H), 2.31 – 2.15 (m, 1H), 1.95 (ddd, J = 12.7, 4.5, 2.2 Hz, 1H), 1.82 – 1.74 (m, 1H), 1.71 – 1.57 (m, 2H), 1.41 – 1.30 (m, 2H), 1.24 – 1.09 (m, 3H), 0.94 – 0.74 (m, 1H).
Chiral SFC: The enantiopurity was determined to be 99% ee by chiral SFC analysis. (OJ-3, 3% MeOH/IPA + 0.2% Formic Acid. 5 min, 2.5 mL/min. λ=230 nm, RT: Major: 1.81 min. Minor: 2.01 min.

2,2,2-trichloroethyl (1*S*,2*S*)-1-(4-bromophenyl)-2-(cyclohexylmethyl)cyclopropane-1-carboxylate (34)

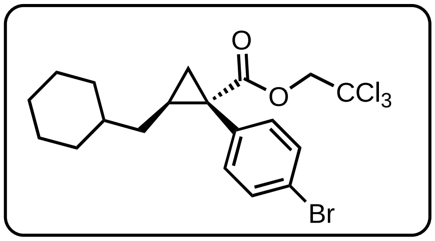

^{1}H NMR (400 MHz, CDCl$_3$) δ 7.45 (d, J = 8.4 Hz, 2H), 7.17 (d, J = 8.4 Hz, 2H), 4.79 (d, J = 11.9 Hz, 1H), 4.55 (d, J = 11.9 Hz, 1H), 1.97 (tdd, J = 10.1, 6.6, 3.6 Hz, 1H), 1.89 (dd, J = 9.0, 4.1 Hz, 1H), 1.76 – 1.59 (m, 5H), 1.42 – 1.28 (m, 2H), 1.27 – 1.06 (m, 4H), 0.92 – 0.76 (m, 2H), 0.38 – 0.27 (m, 1H).
^{13}C NMR (101 MHz, CDCl$_3$) δ 172.7, 134.4, 133.2, 131.2, 121.4, 95.0, 74.3, 38.0, 37.9, 33.3, 33.3, 32.4, 27.9, 26.5, 26.31, 26.29, 22.7.
HRMS (+p APCI) calcd for $C_{19}H_{23}O_2{}^{79}Br^{35}Cl_3$ [M+H] 466.9942 found 466.9944.

2,2,2-trichloroethyl (*S*)-2-((1*R*,3*R*)-3-allylcyclohexyl)-2-(4-bromophenyl)acetate (35)

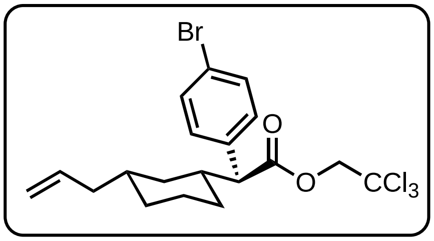

Reported as a 2:1 mixture of diastereomers:
^{1}H NMR (400 MHz, CDCl$_3$) δ 7.45 (dd, J = 8.5, 2.7 Hz, 2H), 7.23 (d, J = 8.4 Hz, 3H), 5.81 – 5.72 (m, 0.2H), 5.73 – 5.59 (m, 1H), 5.01 – 4.93 (m, 0.5H), 4.92 (s, 1H), 4.88 (q, J = 1.9 Hz, 1H), 4.76 (d, J = 12.0 Hz, 1H), 4.63 (d, J = 12.0 Hz, 1H), 3.32 (d, J = 10.4 Hz, 1H), 2.13 – 1.99 (m, 1H), 1.93 (dt, J = 13.8, 6.5 Hz, 1H), 1.86 (dd, J = 12.6, 6.2 Hz, 2H) 1.83 – 1.61 (m, 3H), 1.37 (d, J = 12.7 Hz, 1H), 1.34 – 1.17 (m, 3H), 1.00 (qd, J = 12.6, 3.6 Hz, 1H), 0.88 – 0.70 (m, 1.5H), 0.48 (q, J = 12.1 Hz, 1H).
^{13}C NMR (101 MHz, CDCl$_3$) δ 135.7, 131.7, 130.4, 121.6, 115.7, 94.8, 74.1, 58.3, 41.7, 40.8, 37.3, 36.8, 32.2, 31.7, 25.7.
HRMS (+p APCI) calcd for $C_{19}H_{23}O_2{}^{79}Br^{35}Cl_3$ [M+H] 466.9942 found 466.9941.

HPLC Traces

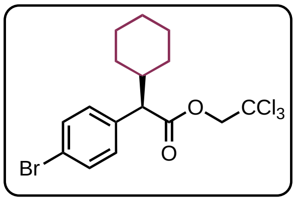

Compound **15**

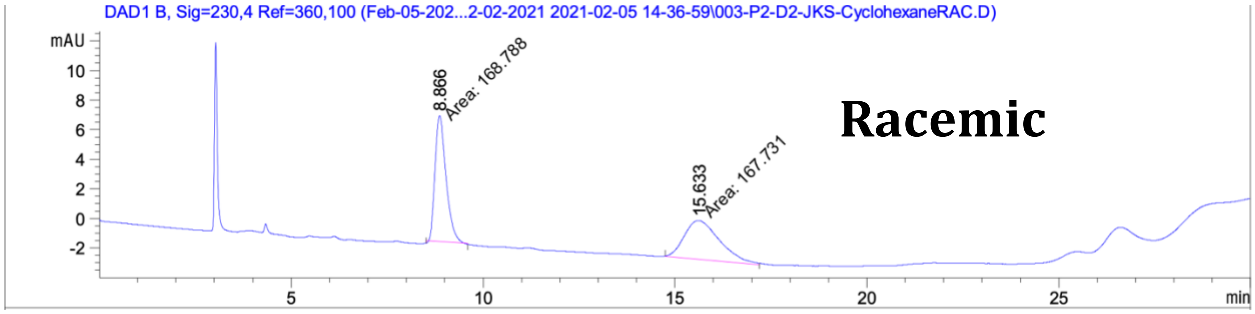

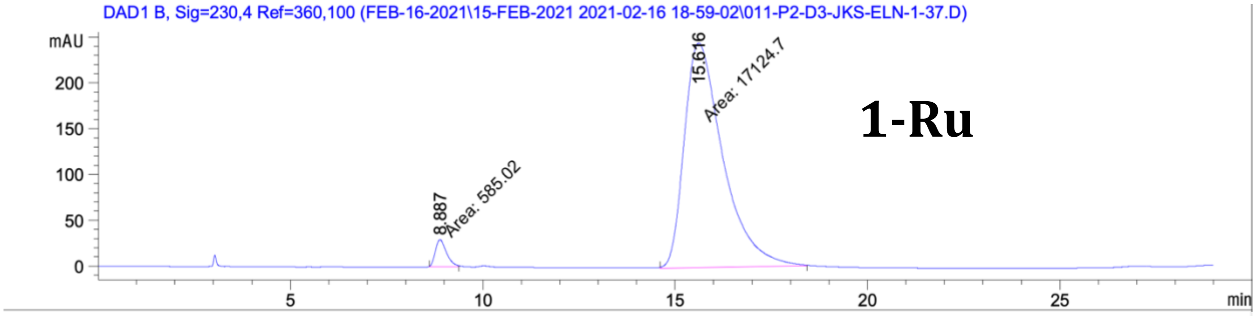

Signal 2: DAD1 B, Sig=230,4 Ref=360,100

Peak #	RetTime [min]	Type	Width [min]	Area [mAU*s]	Height [mAU]	Area %
1	8.887	MM	0.3316	585.01965	29.40377	3.3034
2	15.616	MM	1.1627	1.71247e4	245.47913	96.6966

Totals : 1.77097e4 274.88289

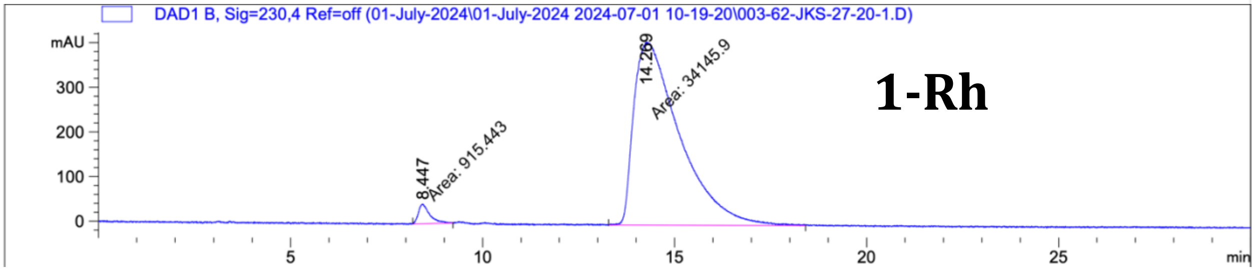

Signal 2: DAD1 B, Sig=230,4 Ref=off

Peak #	RetTime [min]	Type	Width [min]	Area [mAU*s]	Height [mAU]	Area %
1	8.447	MM	0.3431	915.44293	44.47035	2.6110
2	14.269	MM	1.3859	3.41459e4	410.64337	97.3890

Totals : 3.50613e4 455.11372

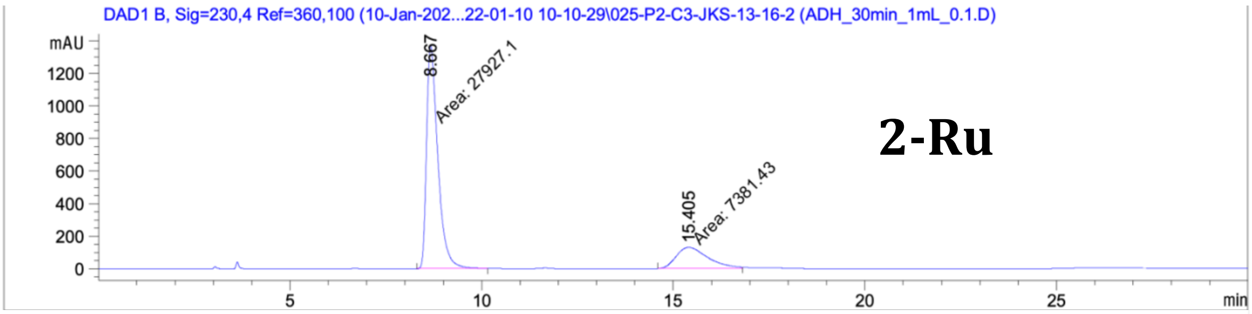

Signal 2: DAD1 B, Sig=230,4 Ref=360,100

Peak #	RetTime [min]	Type	Width [min]	Area [mAU*s]	Height [mAU]	Area %
1	8.667	MM	0.3393	2.79271e4	1371.97913	79.0945
2	15.405	MM	0.9555	7381.43066	128.74814	20.9055
Totals :				3.53086e4	1500.72726	

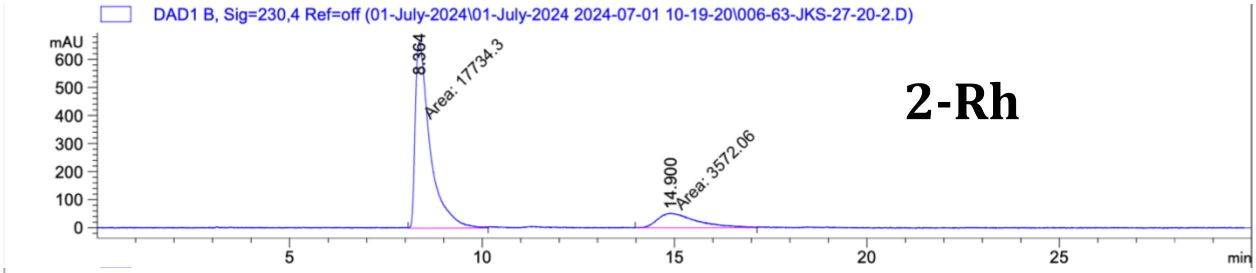

Signal 2: DAD1 B, Sig=230,4 Ref=off

Peak #	RetTime [min]	Type	Width [min]	Area [mAU*s]	Height [mAU]	Area %
1	8.364	MM	0.4456	1.77343e4	663.34113	83.2348
2	14.900	MM	1.1611	3572.06177	51.27624	16.7652
Totals :				2.13064e4	714.61736	

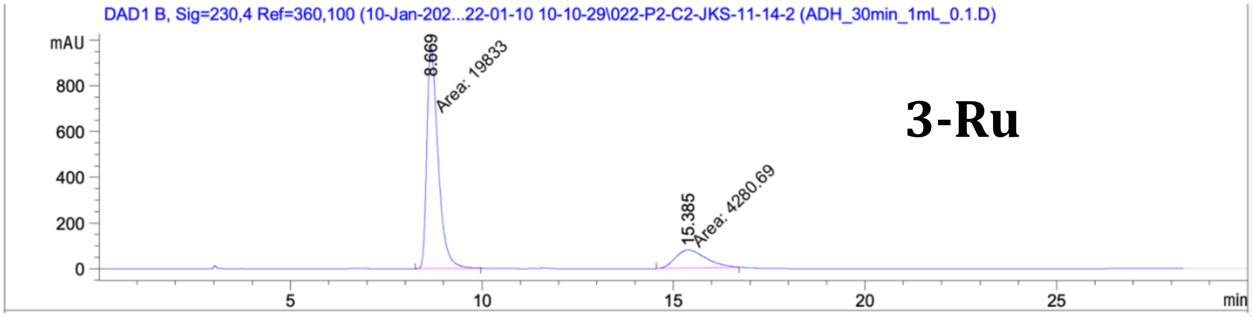

Signal 2: DAD1 B, Sig=230,4 Ref=360,100

Peak #	RetTime [min]	Type	Width [min]	Area [mAU*s]	Height [mAU]	Area %
1	8.669	MM	0.3376	1.98330e4	979.10876	82.2479
2	15.385	MM	0.9136	4280.68750	78.09247	17.7521
Totals :				2.41137e4	1057.20123	

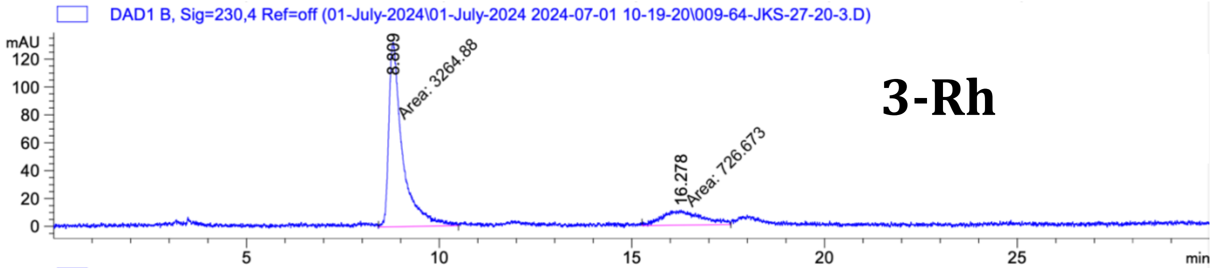

Signal 2: DAD1 B, Sig=230,4 Ref=off

Peak #	RetTime [min]	Type	Width [min]	Area [mAU*s]	Height [mAU]	Area %
1	8.809	MM	0.4103	3264.87573	132.62936	81.7947
2	16.278	MM	1.1151	726.67279	10.86073	18.2053
Totals :				3991.54852	143.49010	

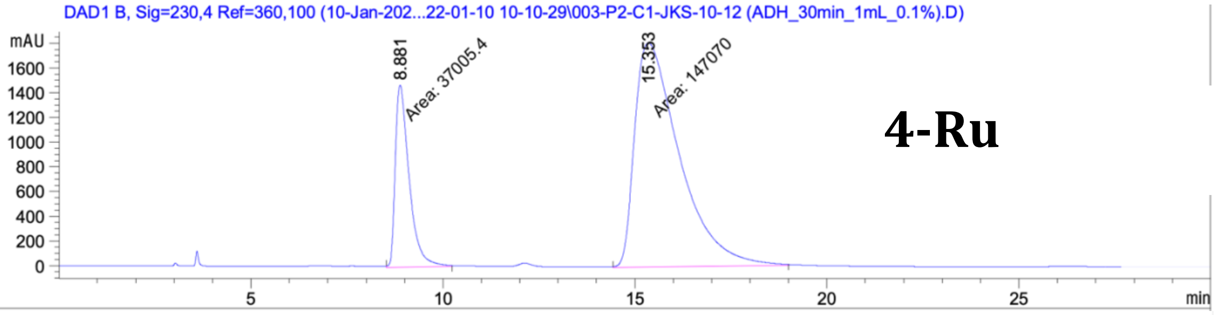

Signal 2: DAD1 B, Sig=230,4 Ref=360,100

Peak #	RetTime [min]	Type	Width [min]	Area [mAU*s]	Height [mAU]	Area %
1	8.881	MM	0.4183	3.70054e4	1474.28943	20.1034
2	15.353	MM	1.3506	1.47070e5	1814.82178	79.8966
Totals :				1.84075e5	3289.11121	

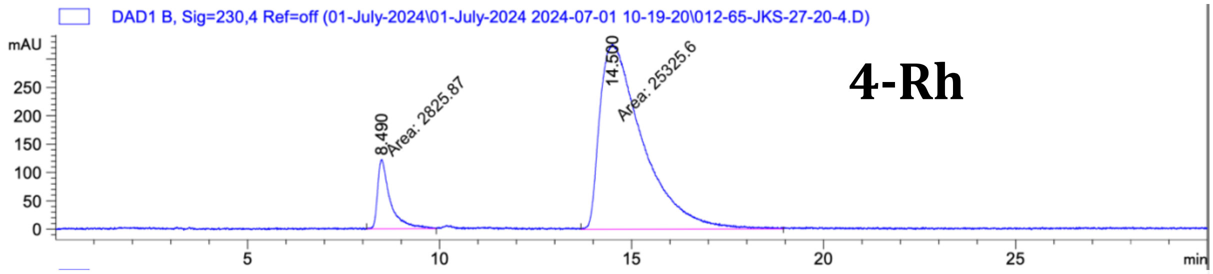

Peak #	RetTime [min]	Type	Width [min]	Area [mAU*s]	Height [mAU]	Area %
1	8.490	MM	0.3841	2825.86792	122.63290	10.0381
2	14.500	MM	1.2933	2.53256e4	326.35834	89.9619
Totals :				2.81514e4	448.99123	

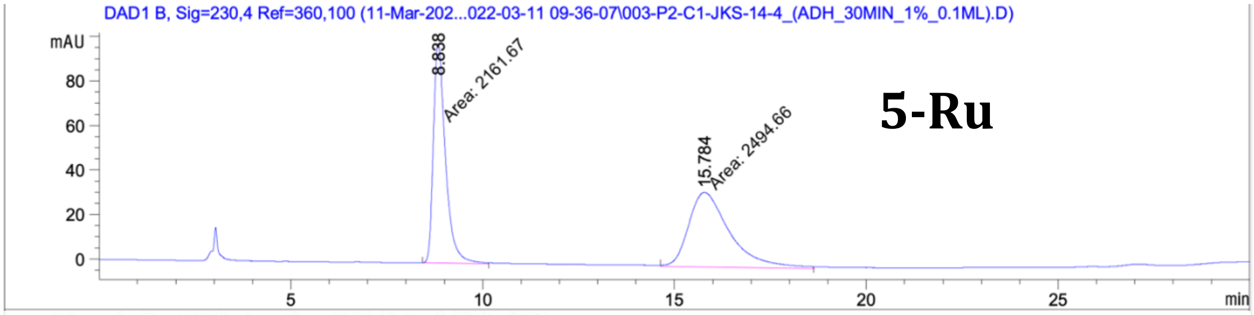

5-Ru

Signal 2: DAD1 B, Sig=230,4 Ref=360,100

Peak #	RetTime [min]	Type	Width [min]	Area [mAU*s]	Height [mAU]	Area %
1	8.838	MM	0.3653	2161.67261	98.62448	46.4244
2	15.784	MM	1.2428	2494.65674	33.45412	53.5756
Totals :				4656.32935	132.07861	

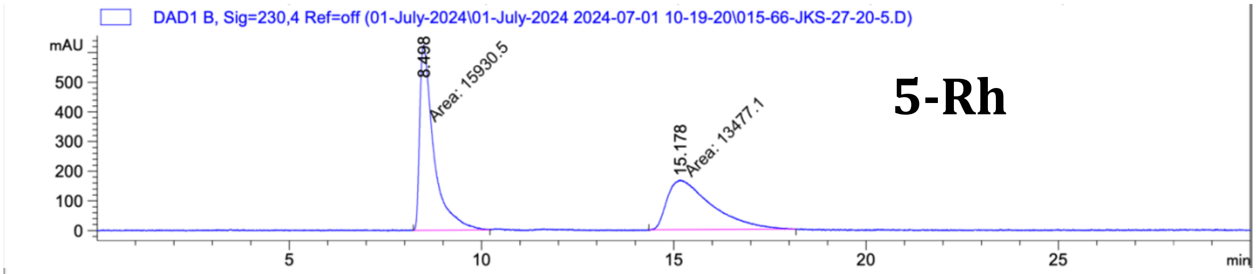

5-Rh

Signal 2: DAD1 B, Sig=230,4 Ref=off

Peak #	RetTime [min]	Type	Width [min]	Area [mAU*s]	Height [mAU]	Area %
1	8.498	MM	0.4245	1.59305e4	625.40485	54.1714
2	15.178	MM	1.3357	1.34771e4	168.16658	45.8286

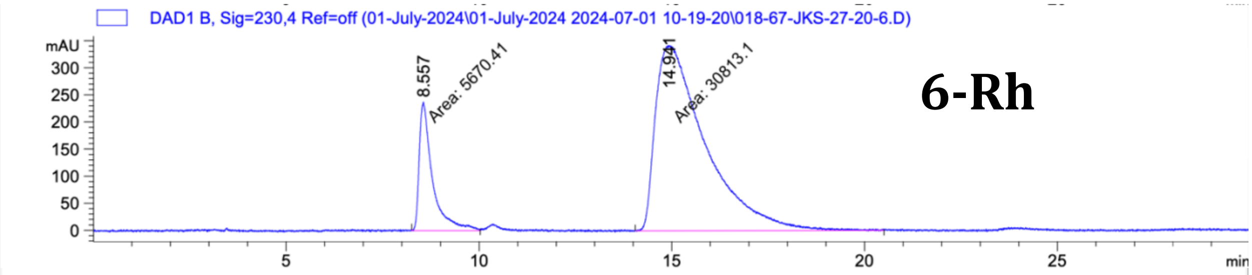

6-Rh

Signal 2: DAD1 B, Sig=230,4 Ref=off

Peak #	RetTime [min]	Type	Width [min]	Area [mAU*s]	Height [mAU]	Area %
1	8.557	MM	0.3972	5670.40527	237.92604	15.5424
2	14.941	MM	1.5018	3.08131e4	341.96072	84.4576
Totals :				3.64835e4	579.88676	

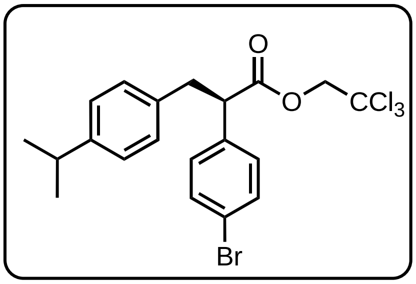

Compound **9**

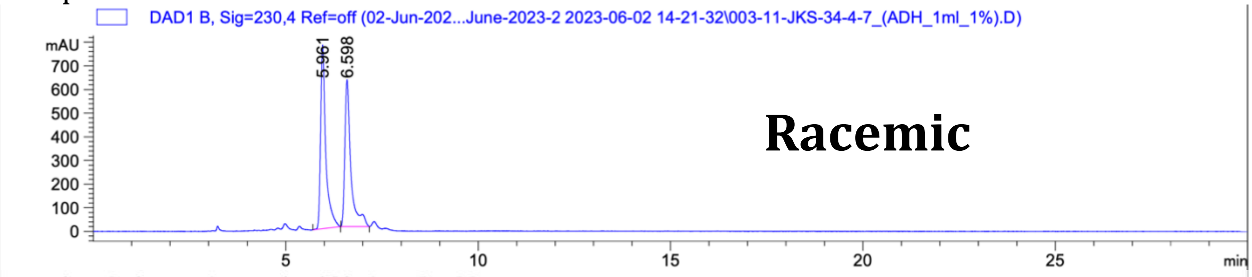

Signal 2: DAD1 B, Sig=230,4 Ref=off

Peak #	RetTime [min]	Type	Width [min]	Area [mAU*s]	Height [mAU]	Area %
1	5.961	VV R	0.1315	7262.04541	777.55066	51.6840
2	6.598	BV R	0.1303	6788.81104	621.82666	48.3160

Totals : 1.40509e4 1399.37732

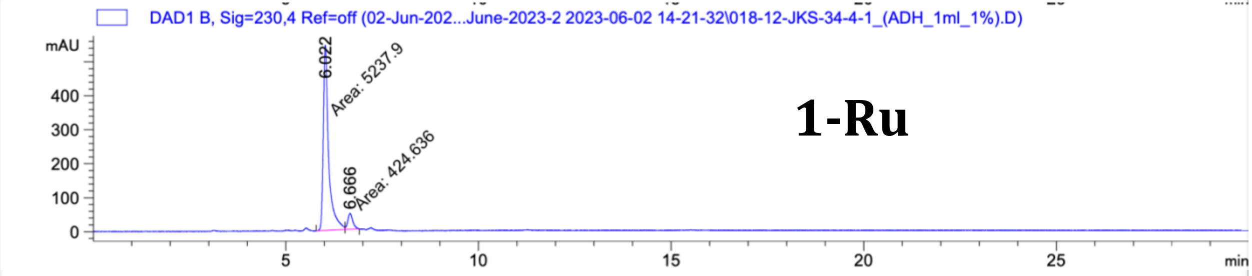

Signal 2: DAD1 B, Sig=230,4 Ref=off

Peak #	RetTime [min]	Type	Width [min]	Area [mAU*s]	Height [mAU]	Area %
1	6.022	MF	0.1601	5237.90234	545.31964	92.5010
2	6.666	FM	0.1499	424.63586	47.20139	7.4990

Totals : 5662.53821 592.52103

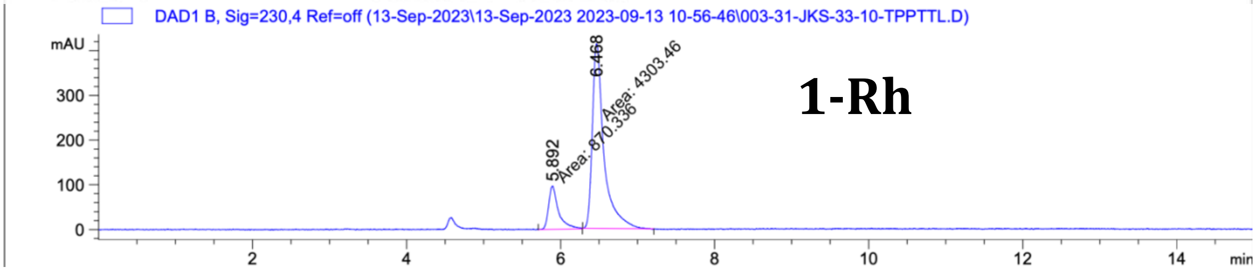

Signal 2: DAD1 B, Sig=230,4 Ref=off

Peak #	RetTime [min]	Type	Width [min]	Area [mAU*s]	Height [mAU]	Area %
1	5.892	MM	0.1501	870.33575	96.65264	16.8220
2	6.468	MM	0.1735	4303.46338	413.30762	83.1780

Totals : 5173.79913 509.96026

Signal 3: DAD1 C, Sig=254,4 Ref=off

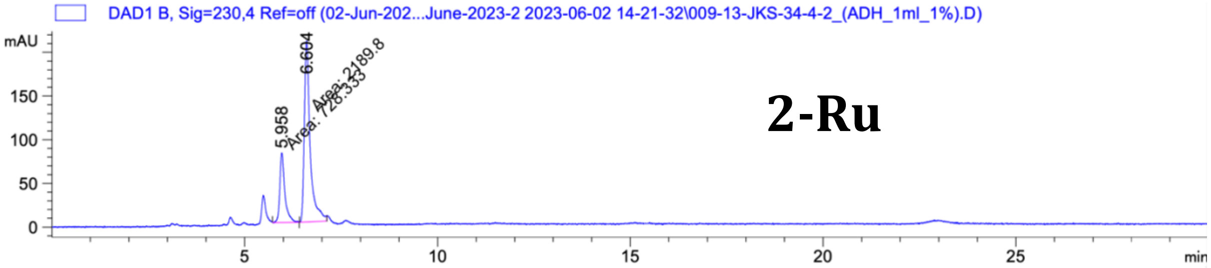

Signal 2: DAD1 B, Sig=230,4 Ref=off

Peak #	RetTime [min]	Type	Width [min]	Area [mAU*s]	Height [mAU]	Area %
1	5.958	MM	0.1521	728.33270	79.78551	24.9589
2	6.604	MM	0.1758	2189.79541	207.56432	75.0411

Totals : 2918.12811 287.34982

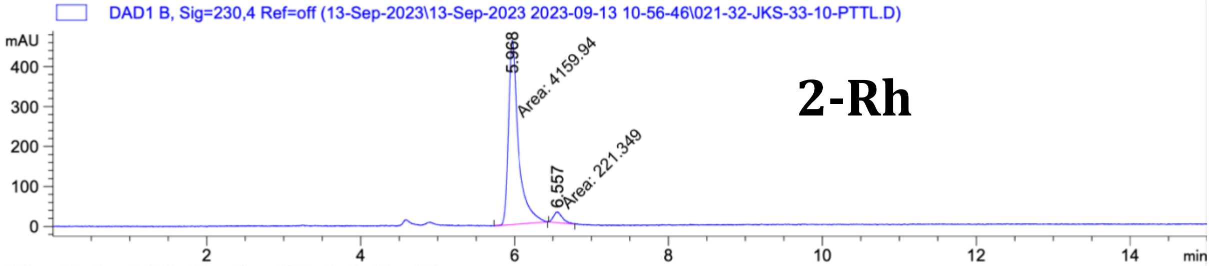

Signal 2: DAD1 B, Sig=230,4 Ref=off

Peak #	RetTime [min]	Type	Width [min]	Area [mAU*s]	Height [mAU]	Area %
1	5.968	MM	0.1503	4159.93652	461.19974	94.9478
2	6.557	MM	0.1373	221.34937	26.87223	5.0522

Totals : 4381.28589 488.07197

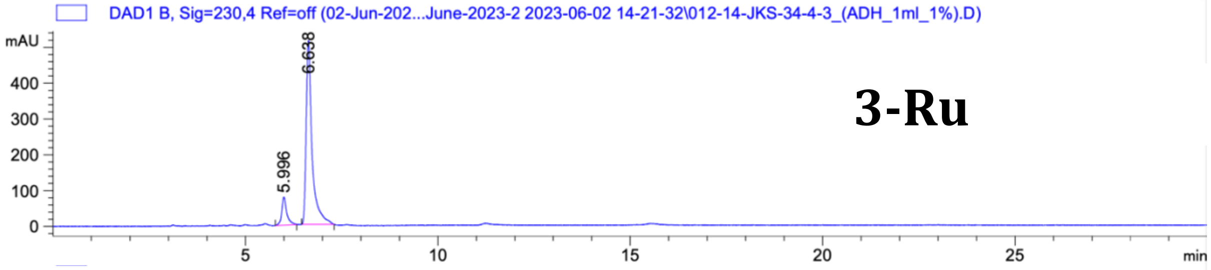

Signal 2: DAD1 B, Sig=230,4 Ref=off

Peak #	RetTime [min]	Type	Width [min]	Area [mAU*s]	Height [mAU]	Area %
1	5.996	BV R	0.1112	717.43719	78.40846	11.6098
2	6.638	BV R	0.1477	5462.13477	513.43555	88.3902

Totals : 6179.57196 591.84401

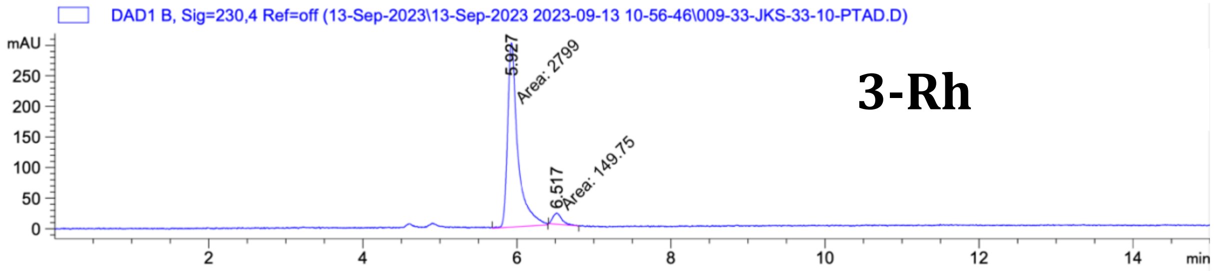

3-Rh

Signal 2: DAD1 B, Sig=230,4 Ref=off

Peak #	RetTime [min]	Type	Width [min]	Area [mAU*s]	Height [mAU]	Area %
1	5.927	MM	0.1547	2799.00171	301.45557	94.9216
2	6.517	MM	0.1351	149.75041	18.47223	5.0784
Totals :				2948.75212	319.92779	

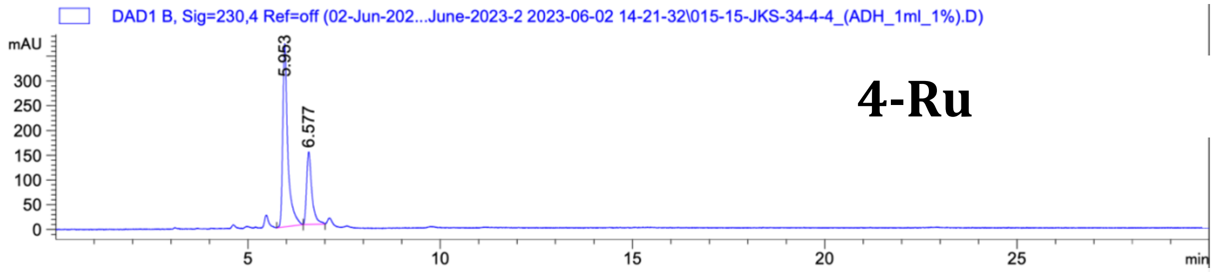

4-Ru

Signal 2: DAD1 B, Sig=230,4 Ref=off

Peak #	RetTime [min]	Type	Width [min]	Area [mAU*s]	Height [mAU]	Area %
1	5.953	VV R	0.1295	3358.67822	369.78372	70.7545
2	6.577	BV R	0.1230	1388.26697	146.45894	29.2455
Totals :				4746.94519	516.24266	

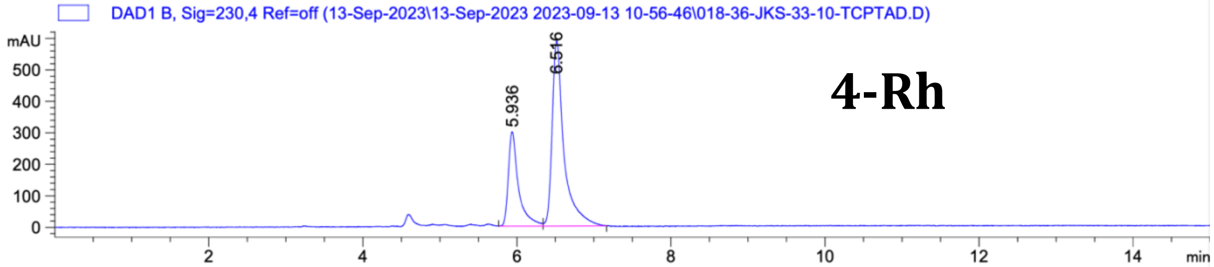

4-Rh

Signal 2: DAD1 B, Sig=230,4 Ref=off

Peak #	RetTime [min]	Type	Width [min]	Area [mAU*s]	Height [mAU]	Area %
1	5.936	BV R	0.1269	2756.40015	299.46103	30.6598
2	6.516	VV R	0.1404	6233.86621	588.56726	69.3402
Totals :				8990.26636	888.02829	

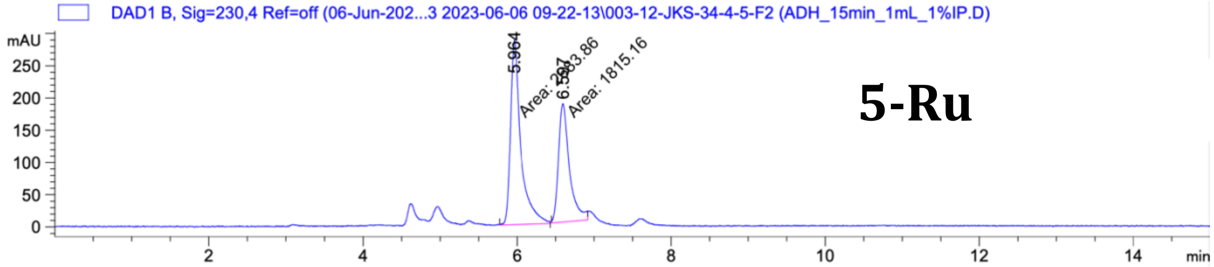

5-Ru

Signal 2: DAD1 B, Sig=230,4 Ref=off

Peak #	RetTime [min]	Type	Width [min]	Area [mAU*s]	Height [mAU]	Area %
1	5.964	MM	0.1565	2683.85938	285.83459	59.6543
2	6.597	MM	0.1650	1815.15979	183.32645	40.3457
Totals :				4499.01917	469.16104	

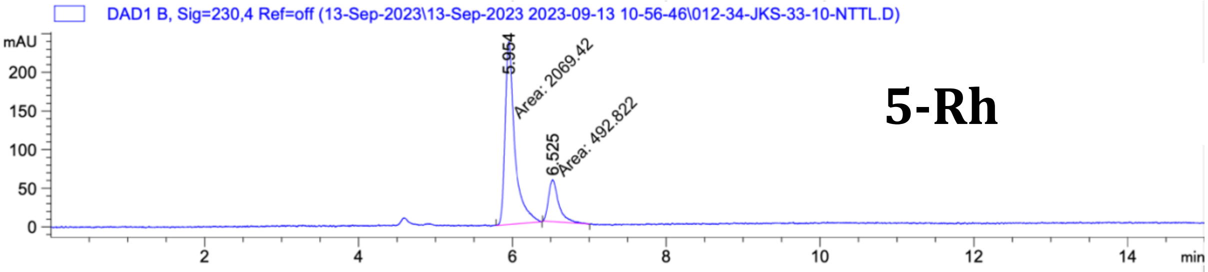

Signal 2: DAD1 B, Sig=230,4 Ref=off

Peak #	RetTime [min]	Type	Width [min]	Area [mAU*s]	Height [mAU]	Area %
1	5.954	MM	0.1455	2069.42065	237.00862	80.7660
2	6.525	MM	0.1508	492.82181	54.46803	19.2340
Totals :				2562.24246	291.47665	

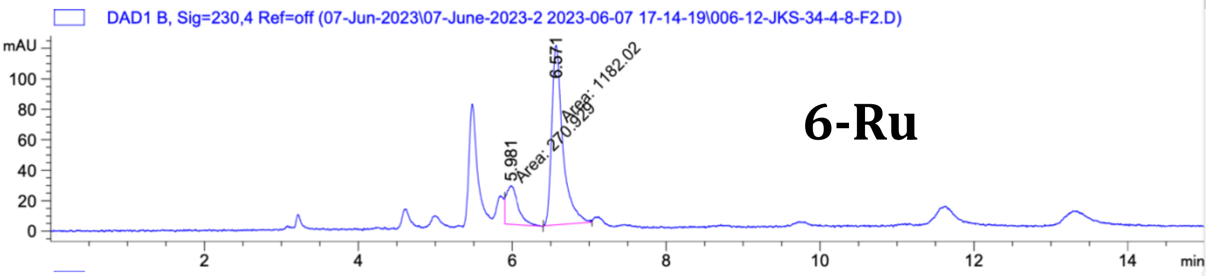

Signal 2: DAD1 B, Sig=230,4 Ref=off

Peak #	RetTime [min]	Type	Width [min]	Area [mAU*s]	Height [mAU]	Area %
1	5.981	MM	0.1777	270.92871	25.41737	18.6468
2	6.571	MM	0.1671	1182.02234	117.91237	81.3532
Totals :				1452.95105	143.32974	

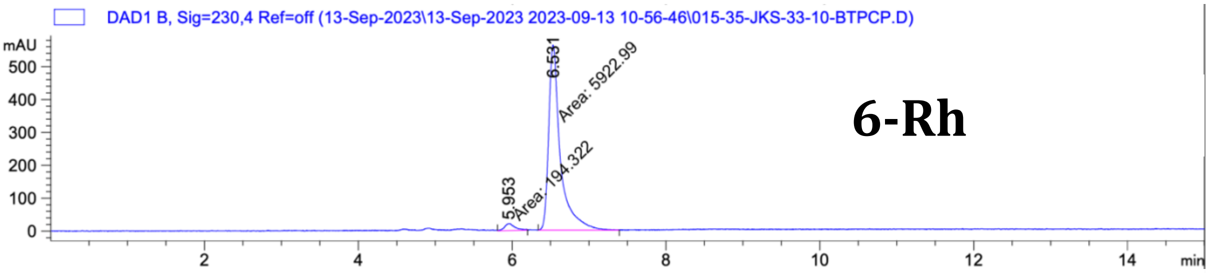

Signal 2: DAD1 B, Sig=230,4 Ref=off

Peak #	RetTime [min]	Type	Width [min]	Area [mAU*s]	Height [mAU]	Area %
1	5.953	MM	0.1543	194.32161	20.98520	3.1766
2	6.531	MM	0.1753	5922.98779	563.09705	96.8234
Totals :				6117.30940	584.08224	

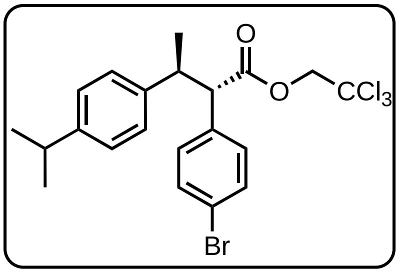

Compound **12a, 12b**

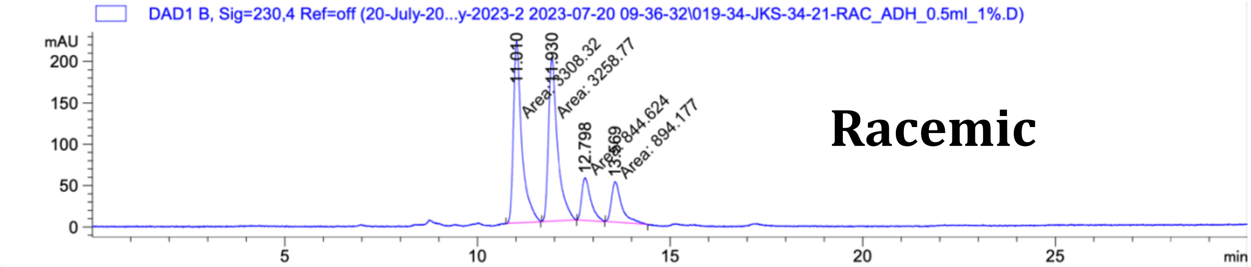

Signal 2: DAD1 B, Sig=230,4 Ref=off

Peak #	RetTime [min]	Type	Width [min]	Area [mAU*s]	Height [mAU]	Area %
1	11.010	MM	0.2512	3308.32300	219.51834	39.8310
2	11.930	MM	0.2740	3258.77319	198.24152	39.2344
3	12.798	MM	0.2725	844.62445	51.66417	10.1690
4	13.569	MM	0.3061	894.17694	48.68564	10.7656
Totals :				8305.89758	518.10967	

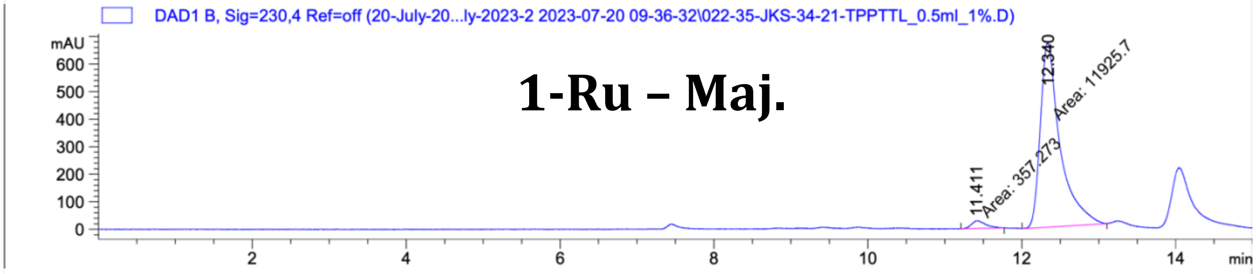

Signal 2: DAD1 B, Sig=230,4 Ref=off

Peak #	RetTime [min]	Type	Width [min]	Area [mAU*s]	Height [mAU]	Area %
1	11.411	MM	0.2128	357.27301	27.97917	2.9087
2	12.340	MM	0.2978	1.19257e4	667.49274	97.0913
Totals :				1.22830e4	695.47190	

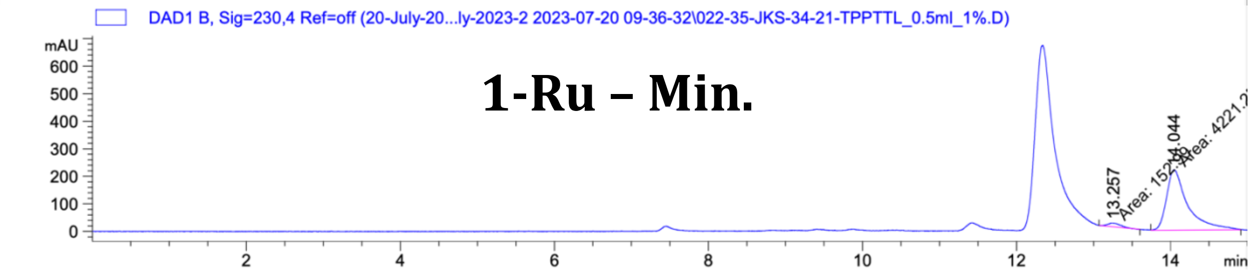

Signal 2: DAD1 B, Sig=230,4 Ref=off

Peak #	RetTime [min]	Type	Width [min]	Area [mAU*s]	Height [mAU]	Area %
1	13.257	MM	0.1941	152.98984	13.13601	3.4975
2	14.044	MM	0.3217	4221.24756	218.68088	96.5025
Totals :				4374.23740	231.81688	

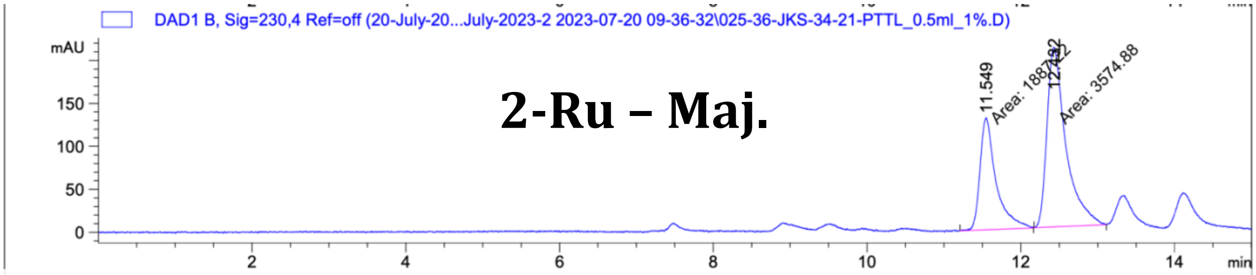

Signal 2: DAD1 B, Sig=230,4 Ref=off

Peak #	RetTime [min]	Type	Width [min]	Area [mAU*s]	Height [mAU]	Area %
1	11.549	MM	0.2411	1887.22473	130.47174	34.5513
2	12.432	MM	0.2849	3574.87720	209.15363	65.4487
Totals :				5462.10193	339.62537	

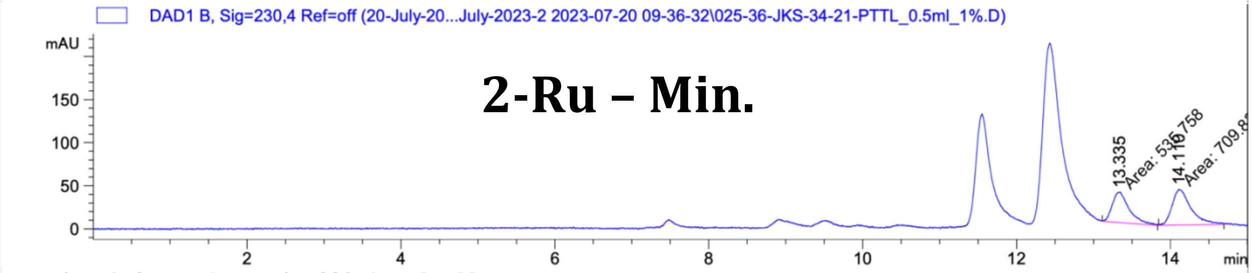

Signal 2: DAD1 B, Sig=230,4 Ref=off

Peak #	RetTime [min]	Type	Width [min]	Area [mAU*s]	Height [mAU]	Area %
1	13.335	MM	0.2533	535.75818	35.24865	43.0128
2	14.110	MM	0.2860	709.82086	41.35924	56.9872
Totals :				1245.57904	76.60789	

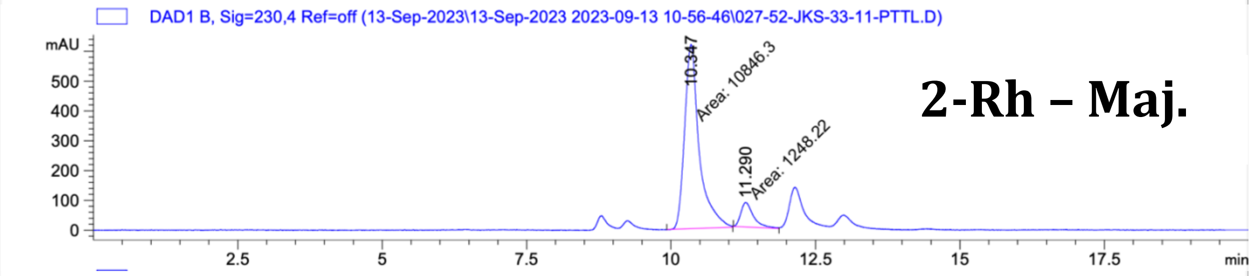

Signal 2: DAD1 B, Sig=230,4 Ref=off

Peak #	RetTime [min]	Type	Width [min]	Area [mAU*s]	Height [mAU]	Area %
1	10.347	MM	0.2923	1.08463e4	618.37231	89.6795
2	11.290	MM	0.2525	1248.21838	82.39369	10.3205
Totals :				1.20946e4	700.76601	

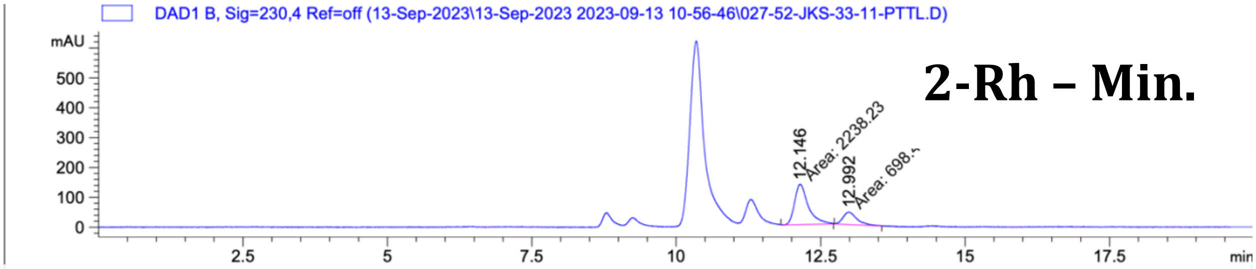

Signal 2: DAD1 B, Sig=230,4 Ref=off

Peak #	RetTime [min]	Type	Width [min]	Area [mAU*s]	Height [mAU]	Area %
1	12.146	MM	0.2755	2238.23438	135.38463	76.2155
2	12.992	MM	0.2756	698.48236	42.23464	23.7845
Totals :				2936.71674	177.61927	

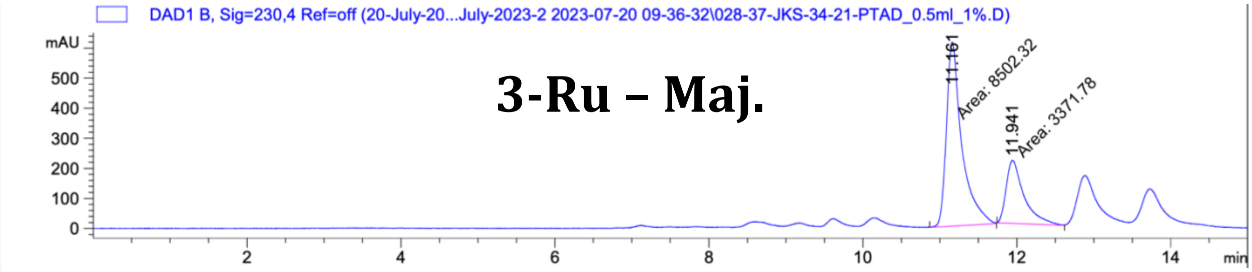

Signal 2: DAD1 B, Sig=230,4 Ref=off

Peak #	RetTime [min]	Type	Width [min]	Area [mAU*s]	Height [mAU]	Area %
1	11.161	MM	0.2315	8502.32324	612.02429	71.6039
2	11.941	MM	0.2677	3371.78076	209.95772	28.3961
Totals :				1.18741e4	821.98201	

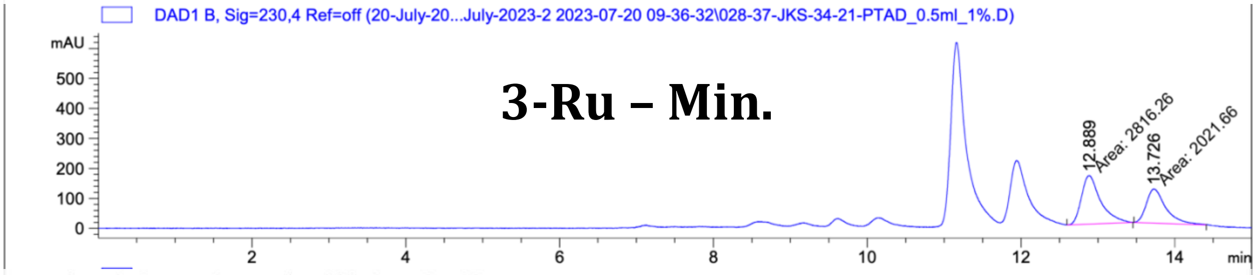

Signal 2: DAD1 B, Sig=230,4 Ref=off

Peak #	RetTime [min]	Type	Width [min]	Area [mAU*s]	Height [mAU]	Area %
1	12.889	MM	0.2895	2816.26245	162.15532	58.2123
2	13.726	MM	0.2946	2021.65747	114.37710	41.7877
Totals :				4837.91992	276.53242	

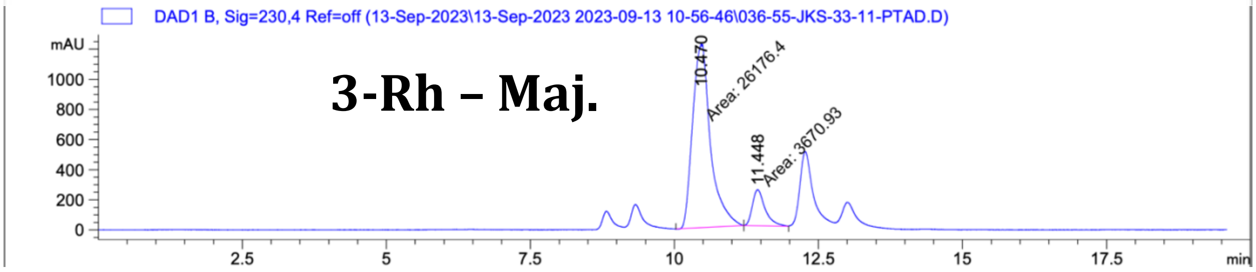

Signal 2: DAD1 B, Sig=230,4 Ref=off

Peak #	RetTime [min]	Type	Width [min]	Area [mAU*s]	Height [mAU]	Area %
1	10.470	MM	0.3581	2.61764e4	1218.39026	87.7010
2	11.448	MM	0.2545	3670.92578	240.37245	12.2990

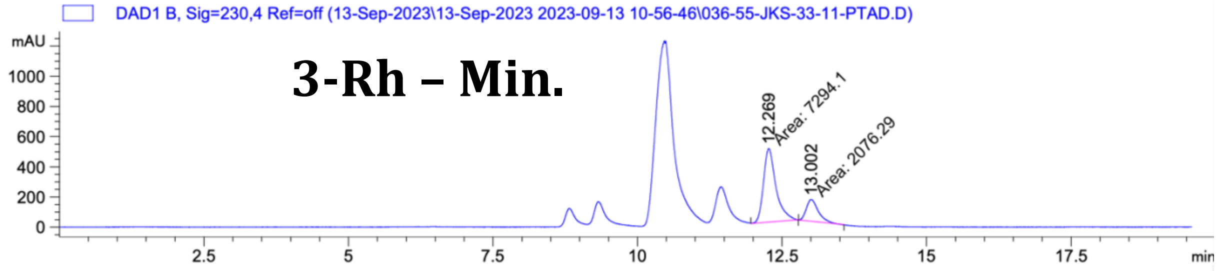

Signal 2: DAD1 B, Sig=230,4 Ref=off

Peak #	RetTime [min]	Type	Width [min]	Area [mAU*s]	Height [mAU]	Area %
1	12.269	MM	0.2496	7294.10254	486.96356	77.8420
2	13.002	MM	0.2400	2076.28882	144.18231	22.1580

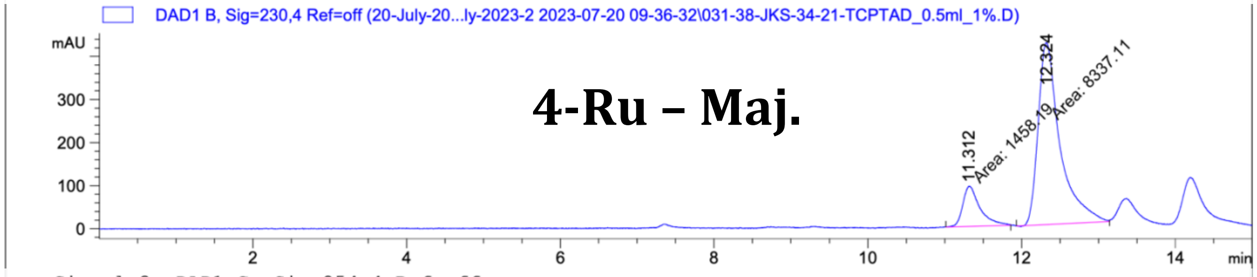

Signal 3: DAD1 C, Sig=254,4 Ref=off

Peak #	RetTime [min]	Type	Width [min]	Area [mAU*s]	Height [mAU]	Area %
1	12.237	BV	0.0355	25.27437	8.92675	15.8721
2	12.305	VV	0.0911	102.95455	13.55901	64.6546
3	12.397	VV	0.0386	31.00882	10.14883	19.4733
Totals :				159.23774	32.63458	

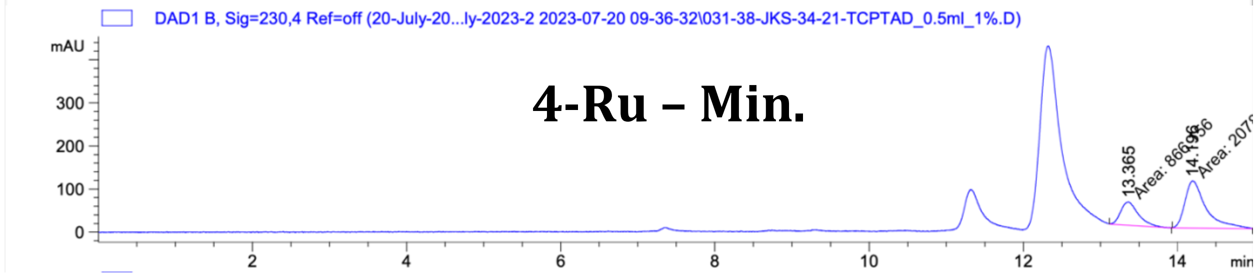

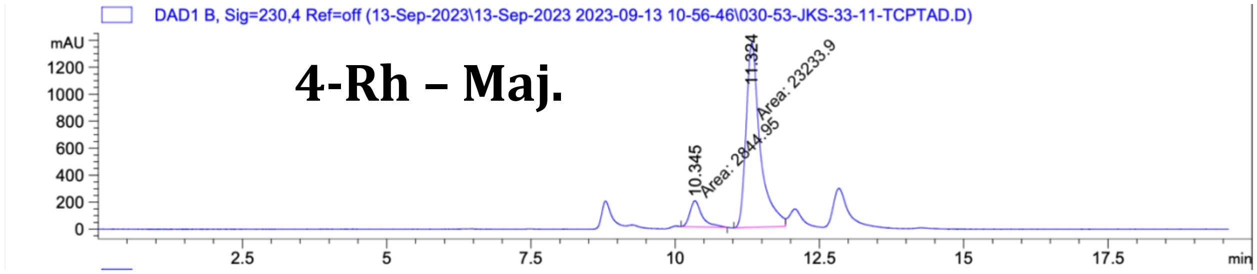

Signal 2: DAD1 B, Sig=230,4 Ref=off

Peak #	RetTime [min]	Type	Width [min]	Area [mAU*s]	Height [mAU]	Area %
1	10.345	MM	0.2450	2844.94897	193.52136	10.9090
2	11.324	MM	0.2828	2.32339e4	1369.13428	89.0910

Totals : 2.60788e4 1562.65564

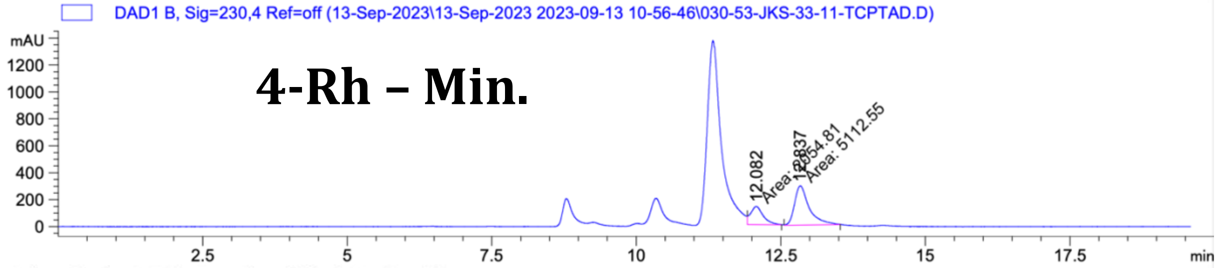

Signal 2: DAD1 B, Sig=230,4 Ref=off

Peak #	RetTime [min]	Type	Width [min]	Area [mAU*s]	Height [mAU]	Area %
1	12.082	MM	0.2547	2054.81299	134.45419	28.6690
2	12.837	MM	0.2925	5112.55029	291.32901	71.3310

Totals : 7167.36328 425.78320

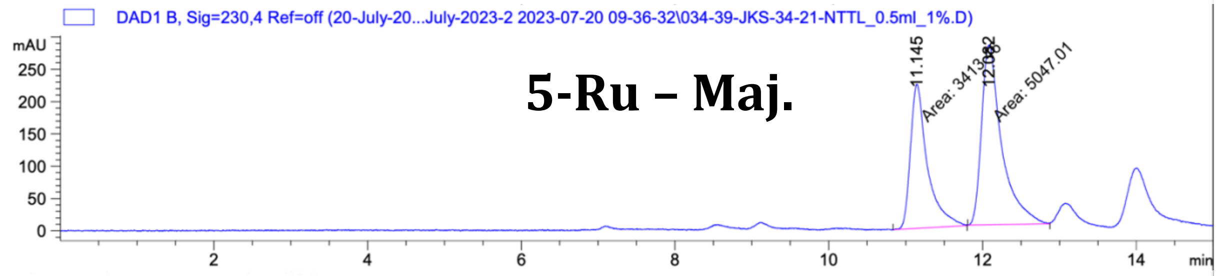

Signal 2: DAD1 B, Sig=230,4 Ref=off

Peak #	RetTime [min]	Type	Width [min]	Area [mAU*s]	Height [mAU]	Area %
1	11.145	MM	0.2548	3413.45874	223.31889	40.3460
2	12.082	MM	0.3022	5047.00635	278.37375	59.6540

Totals : 8460.46509 501.69264

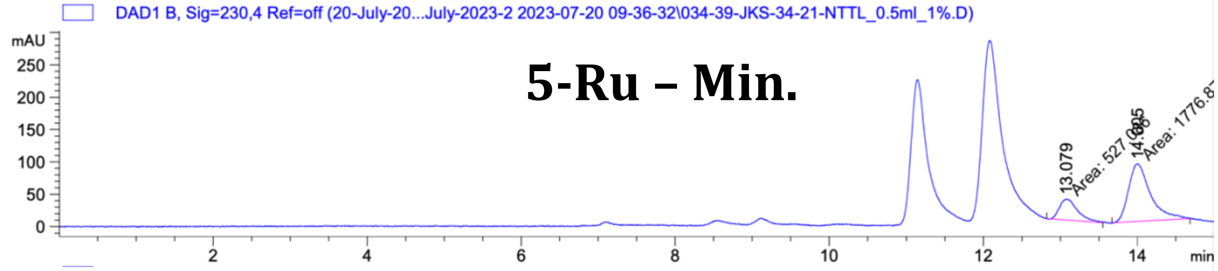

Signal 2: DAD1 B, Sig=230,4 Ref=off

Peak #	RetTime [min]	Type	Width [min]	Area [mAU*s]	Height [mAU]	Area %
1	13.079	MM	0.2686	527.08643	32.70362	22.8775
2	14.005	MM	0.3334	1776.86951	88.83726	77.1225

Totals : 2303.95593 121.54088

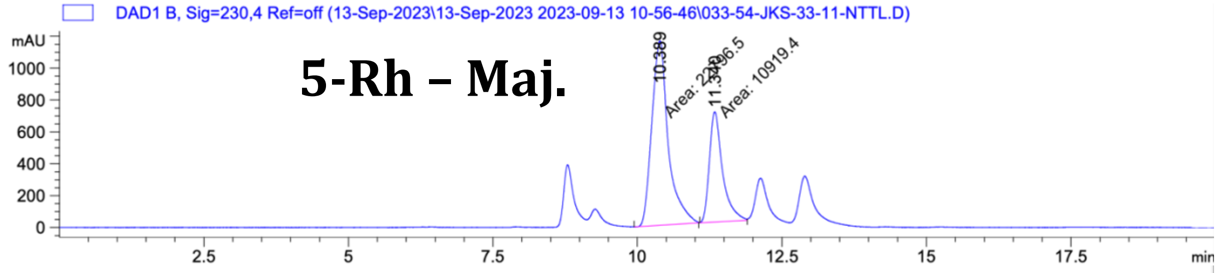

Signal 2: DAD1 B, Sig=230,4 Ref=off

Peak #	RetTime [min]	Type	Width [min]	Area [mAU*s]	Height [mAU]	Area %
1	10.389	MM	0.3279	2.27965e4	1158.75464	67.6135
2	11.340	MM	0.2631	1.09194e4	691.66125	32.3865
Totals :				3.37159e4	1850.41589	

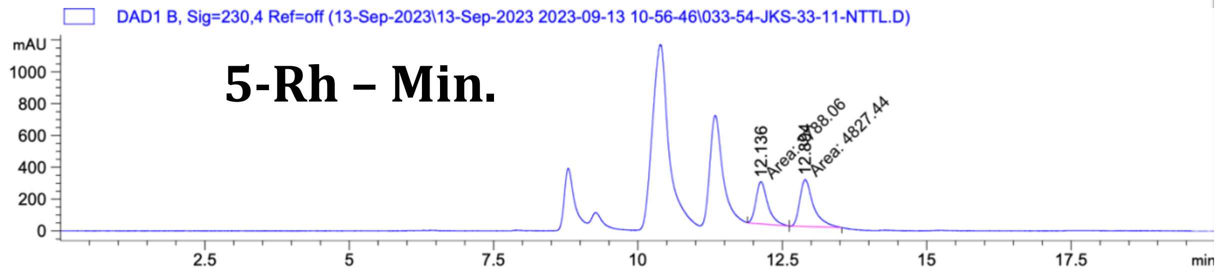

Signal 2: DAD1 B, Sig=230,4 Ref=off

Peak #	RetTime [min]	Type	Width [min]	Area [mAU*s]	Height [mAU]	Area %
1	12.136	MM	0.2381	3788.06030	265.18704	43.9679
2	12.894	MM	0.2719	4827.44482	295.86142	56.0321
Totals :				8615.50513	561.04846	

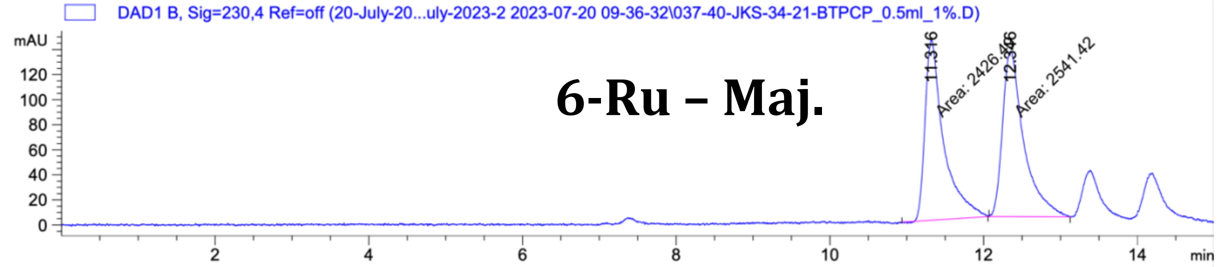

Signal 2: DAD1 B, Sig=230,4 Ref=off

Peak #	RetTime [min]	Type	Width [min]	Area [mAU*s]	Height [mAU]	Area %
1	11.316	MM	0.2815	2426.48779	143.65936	48.8432
2	12.346	MM	0.3211	2541.42163	131.92711	51.1568
Totals :				4967.90942	275.58647	

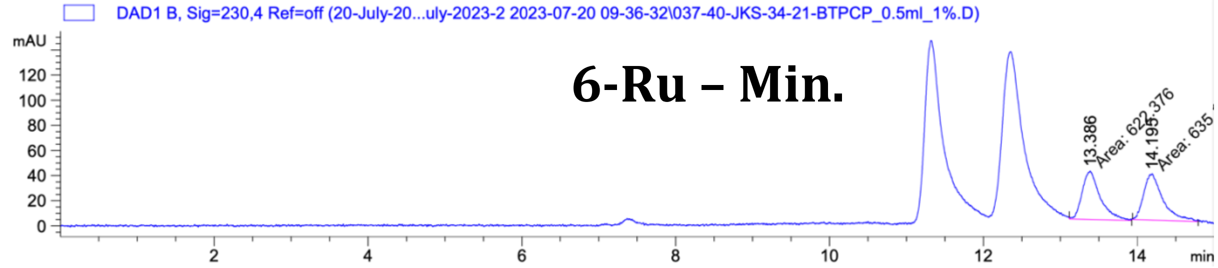

Signal 2: DAD1 B, Sig=230,4 Ref=off

Peak #	RetTime [min]	Type	Width [min]	Area [mAU*s]	Height [mAU]	Area %
1	13.386	MM	0.2699	622.37555	38.43711	49.4927
2	14.195	MM	0.2874	635.13385	36.83137	50.5073
Totals :				1257.50940	75.26849	

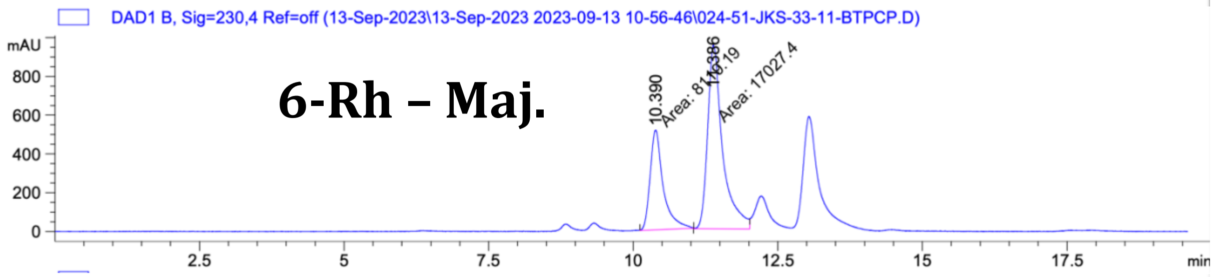

Signal 2: DAD1 B, Sig=230,4 Ref=off

Peak #	RetTime [min]	Type	Width [min]	Area [mAU*s]	Height [mAU]	Area %
1	10.390	MM	0.2626	8119.18652	515.23315	32.2875
2	11.386	MM	0.2996	1.70274e4	947.23621	67.7125
Totals :				2.51466e4	1462.46936	

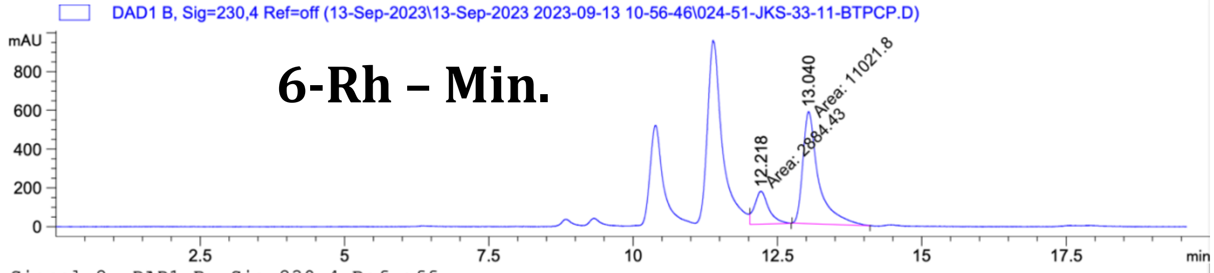

Signal 2: DAD1 B, Sig=230,4 Ref=off

Peak #	RetTime [min]	Type	Width [min]	Area [mAU*s]	Height [mAU]	Area %
1	12.218	MM	0.2829	2884.42603	169.90578	20.7420
2	13.040	MM	0.3171	1.10218e4	579.37549	79.2580
Totals :				1.39062e4	749.28127	

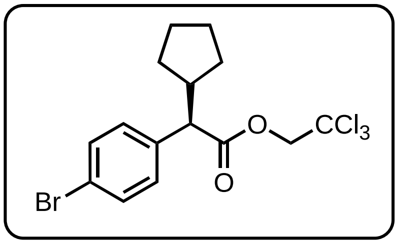

Compound **16**

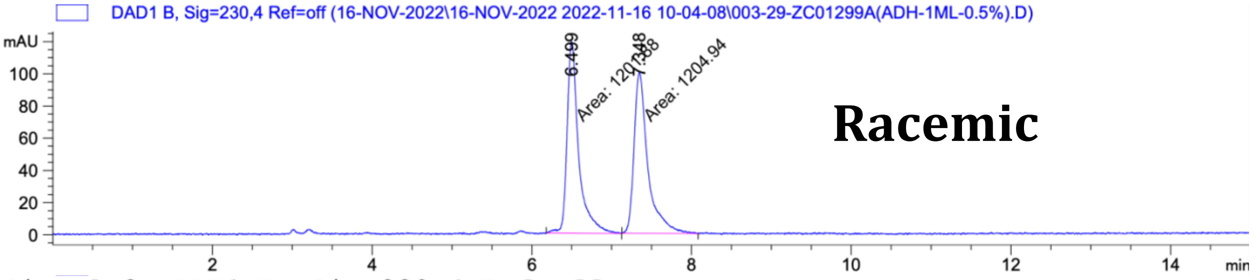

Signal 2: DAD1 B, Sig=230,4 Ref=off

Peak #	RetTime [min]	Type	Width [min]	Area [mAU*s]	Height [mAU]	Area %
1	6.499	MM	0.1682	1201.87817	119.10645	49.9364
2	7.348	MM	0.2007	1204.93994	100.07652	50.0636
Totals :				2406.81812	219.18298	

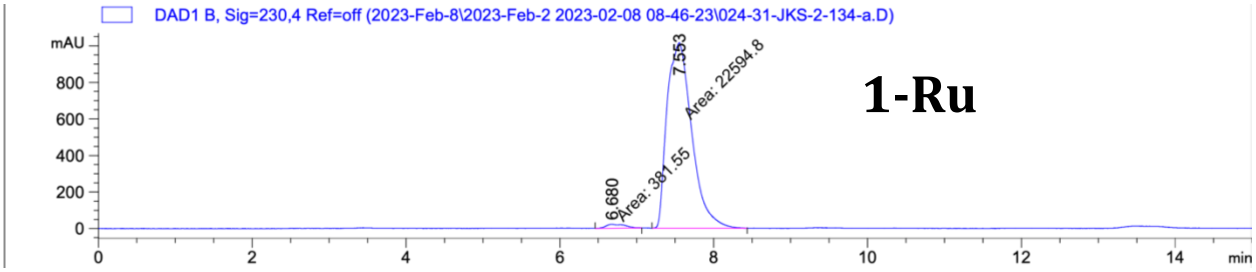

Signal 2: DAD1 B, Sig=230,4 Ref=off

Peak #	RetTime [min]	Type	Width [min]	Area [mAU*s]	Height [mAU]	Area %
1	6.680	MM	0.2961	381.54971	21.47387	1.6606
2	7.553	MM	0.3706	2.25948e4	1016.03406	98.3394
Totals :				2.29764e4	1037.50793	

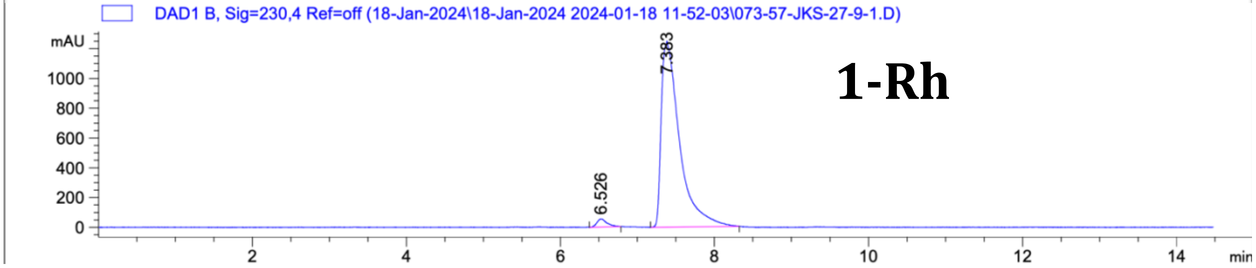

Signal 2: DAD1 B, Sig=230,4 Ref=off

Peak #	RetTime [min]	Type	Width [min]	Area [mAU*s]	Height [mAU]	Area %
1	6.526	VV R	0.1147	496.69824	53.80212	2.3631
2	7.383	VV R	0.1950	2.05225e4	1249.94275	97.6369
Totals :				2.10192e4	1303.74487	

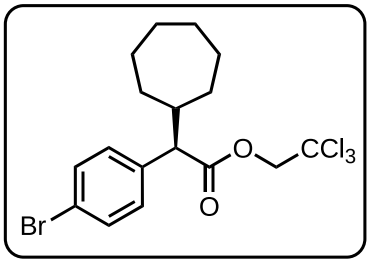

Compound **17**

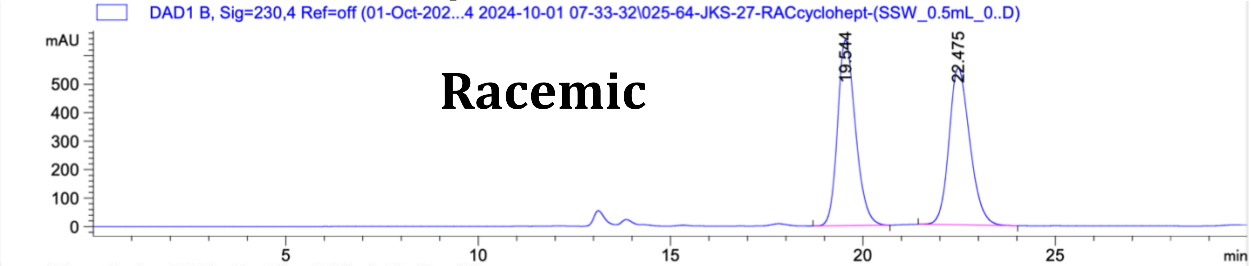

Signal 2: DAD1 B, Sig=230,4 Ref=off

Peak #	RetTime [min]	Type	Width [min]	Area [mAU*s]	Height [mAU]	Area %
1	19.544	BB	0.4846	2.10776e4	651.31793	50.3942
2	22.475	BB	0.5466	2.07478e4	550.01404	49.6058
Totals :				4.18255e4	1201.33197	

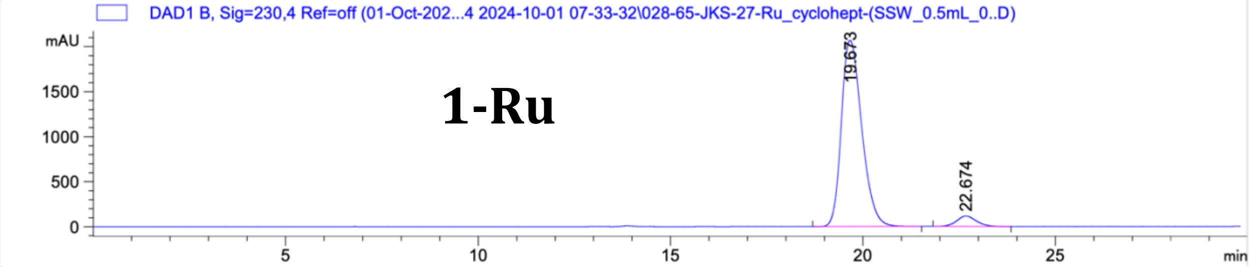

Signal 2: DAD1 B, Sig=230,4 Ref=off

Peak #	RetTime [min]	Type	Width [min]	Area [mAU*s]	Height [mAU]	Area %
1	19.673	BB	0.4343	7.60302e4	2066.04028	94.9019
2	22.674	BB	0.4722	4084.35864	114.90491	5.0981
Totals :				8.01145e4	2180.94520	

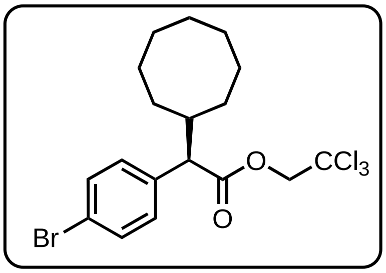

Compound **18**

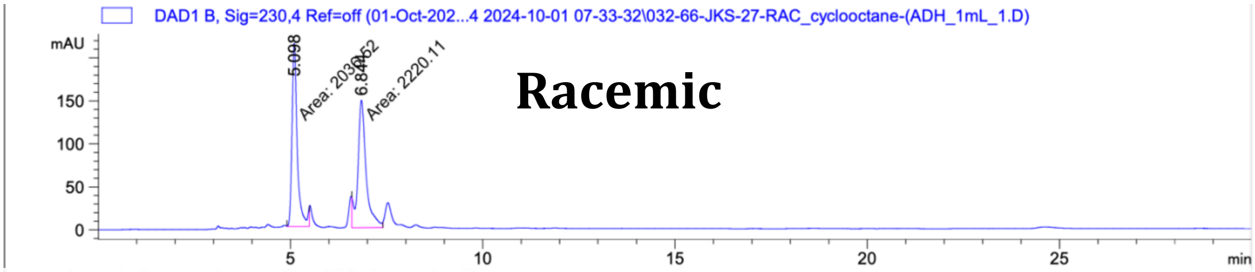

Signal 2: DAD1 B, Sig=230,4 Ref=off

Peak #	RetTime [min]	Type	Width [min]	Area [mAU*s]	Height [mAU]	Area %
1	5.098	MM	0.1593	2036.51501	213.05951	47.8434
2	6.844	MM	0.2498	2220.10986	148.14349	52.1566

Totals : 4256.62488 361.20300

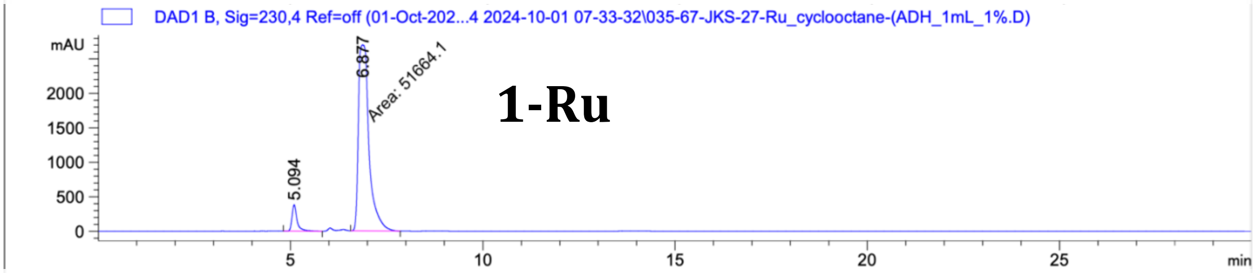

Signal 2: DAD1 B, Sig=230,4 Ref=off

Peak #	RetTime [min]	Type	Width [min]	Area [mAU*s]	Height [mAU]	Area %
1	5.094	BB	0.1365	3498.30005	383.29721	6.3418
2	6.877	MM	0.3184	5.16641e4	2704.20752	93.6582

Totals : 5.51624e4 3087.50473

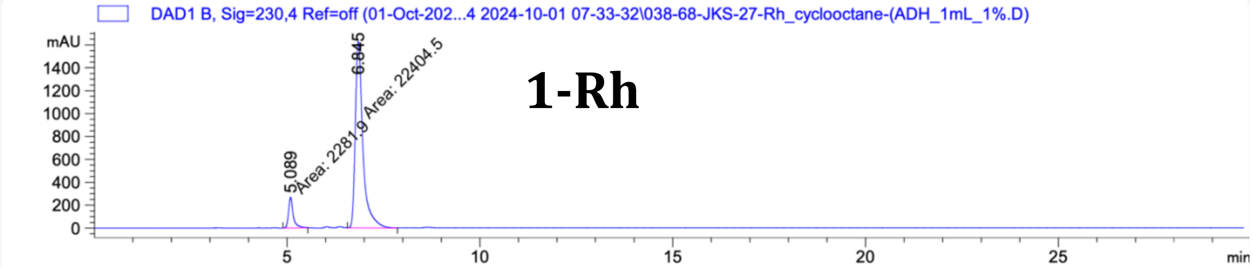

Signal 2: DAD1 B, Sig=230,4 Ref=off

Peak #	RetTime [min]	Type	Width [min]	Area [mAU*s]	Height [mAU]	Area %
1	5.089	MM	0.1416	2281.90063	268.63290	9.2436
2	6.845	MM	0.2291	2.24045e4	1629.59851	90.7564

Totals : 2.46864e4 1898.23141

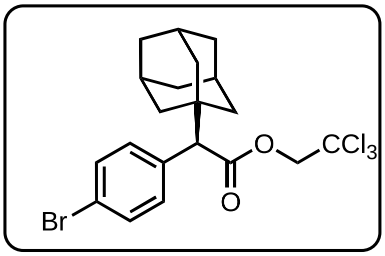

Compound **19**

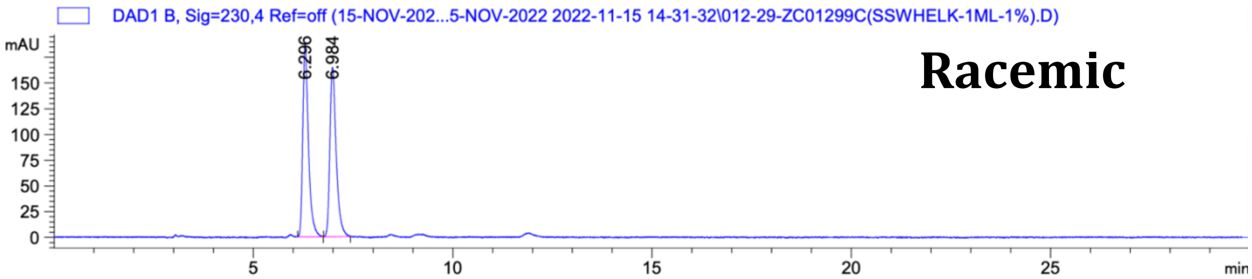

Signal 2: DAD1 B, Sig=230,4 Ref=off

Peak #	RetTime [min]	Type	Width [min]	Area [mAU*s]	Height [mAU]	Area %
1	6.296	BV R	0.1340	1842.88098	186.89391	50.3686
2	6.984	VV R	0.1352	1815.90515	164.64194	49.6314

Totals : 3658.78613 351.53584

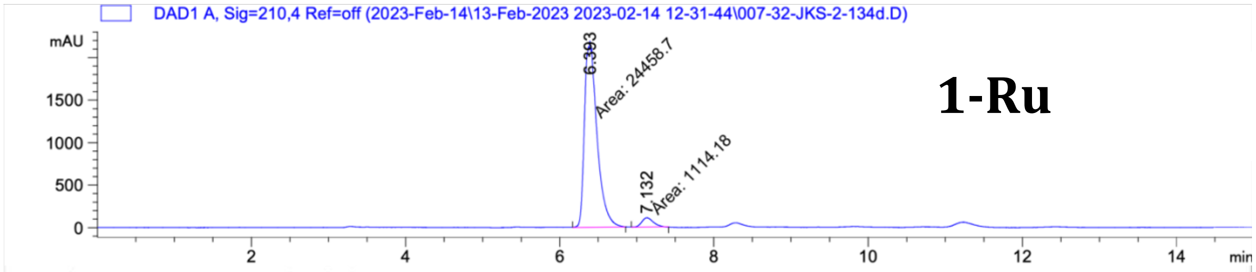

Signal 1: DAD1 A, Sig=210,4 Ref=off

Peak #	RetTime [min]	Type	Width [min]	Area [mAU*s]	Height [mAU]	Area %
1	6.393	MM	0.1859	2.44587e4	2192.61743	95.6431
2	7.132	MM	0.1712	1114.18347	108.44734	4.3569

Totals : 2.55729e4 2301.06477

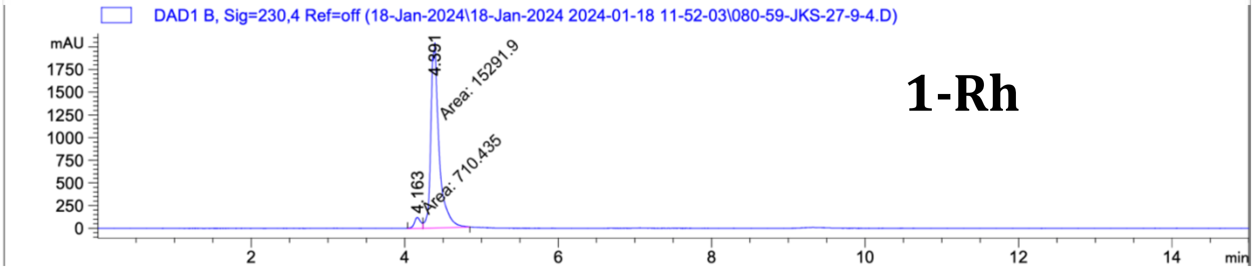

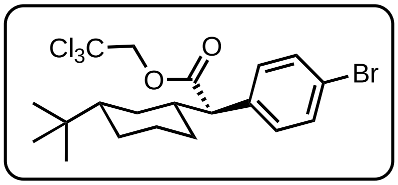

Compound **20**

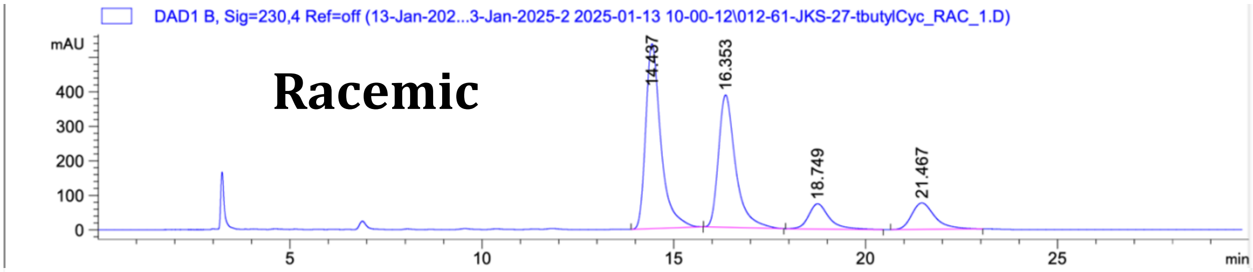

Signal 2: DAD1 B, Sig=230,4 Ref=off

Peak #	RetTime [min]	Type	Width [min]	Area [mAU*s]	Height [mAU]	Area %
1	14.437	BB	0.4059	1.46971e4	536.03296	45.2817
2	16.353	BB	0.4497	1.18732e4	383.55173	36.5815
3	18.749	BB	0.4545	2692.45996	72.88506	8.2955
4	21.467	BB	0.4931	3194.18481	76.34607	9.8413
Totals :				3.24570e4	1068.81582	

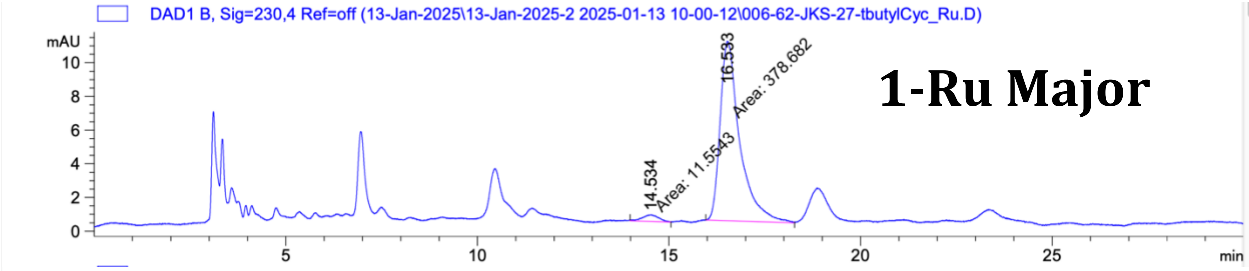

Signal 2: DAD1 B, Sig=230,4 Ref=off

Peak #	RetTime [min]	Type	Width [min]	Area [mAU*s]	Height [mAU]	Area %
1	14.534	MM	0.4844	11.55427	3.97575e-1	2.9608
2	16.533	MM	0.5925	378.68228	10.65221	97.0392
Totals :				390.23655	11.04978	

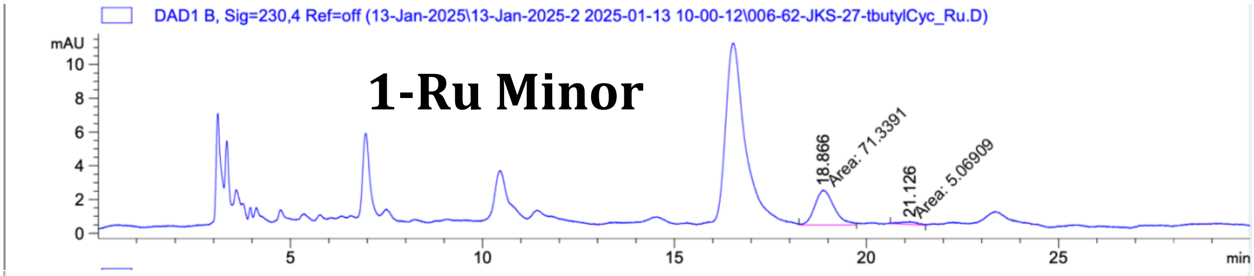

Signal 2: DAD1 B, Sig=230,4 Ref=off

Peak #	RetTime [min]	Type	Width [min]	Area [mAU*s]	Height [mAU]	Area %
1	18.866	MM	0.5742	71.33913	2.07054	93.3658
2	21.126	MM	0.4515	5.06909	1.87129e-1	6.6342
Totals :				76.40821	2.25767	

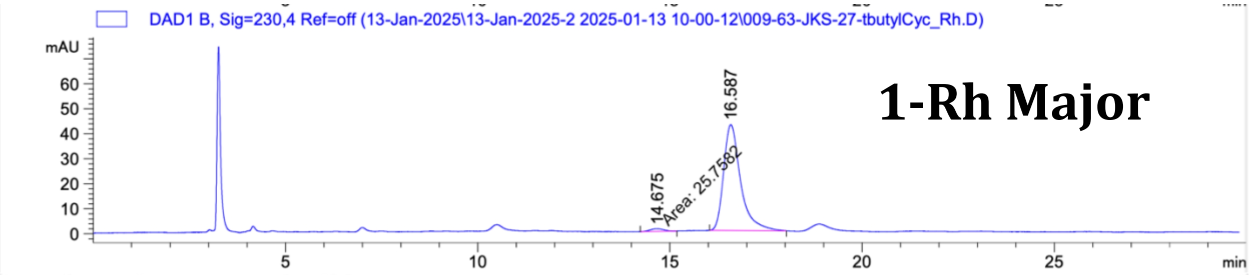

Signal 2: DAD1 B, Sig=230,4 Ref=off

Peak #	RetTime [min]	Type	Width [min]	Area [mAU*s]	Height [mAU]	Area %
1	14.675	MM	0.4057	25.75818	1.05826	1.9406
2	16.587	BB	0.3676	1301.54248	42.34519	98.0594
Totals :				1327.30066	43.40345	

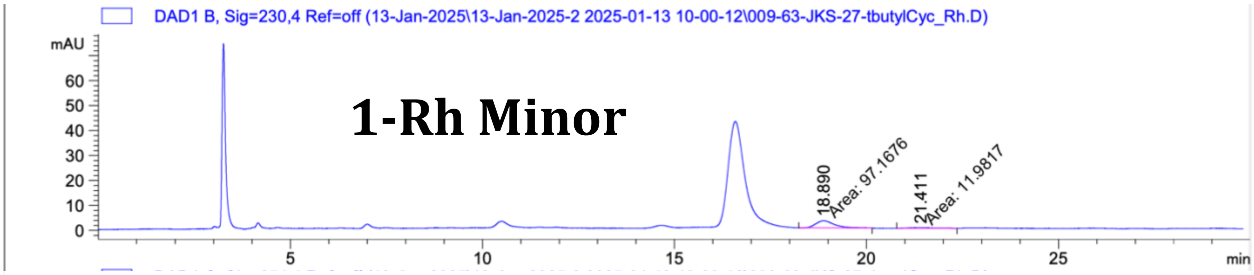

Signal 2: DAD1 B, Sig=230,4 Ref=off

Peak #	RetTime [min]	Type	Width [min]	Area [mAU*s]	Height [mAU]	Area %
1	18.890	MM	0.5588	97.16756	2.89803	89.0227
2	21.411	MM	0.8451	11.98167	2.36291e-1	10.9773
Totals :				109.14924	3.13432	

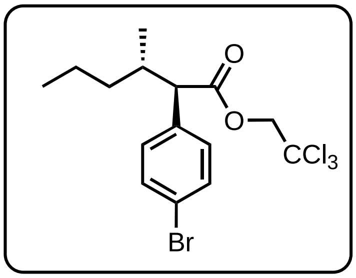

Compound **21**

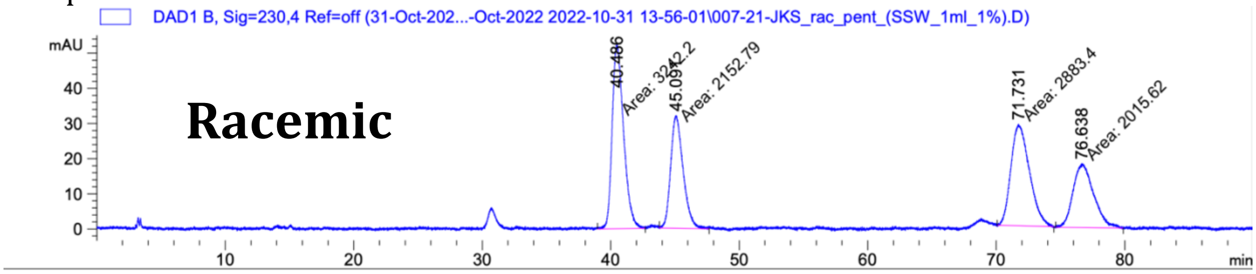

Signal 2: DAD1 B, Sig=230,4 Ref=off

Peak #	RetTime [min]	Type	Width [min]	Area [mAU*s]	Height [mAU]	Area %
1	40.486	MM	1.0320	3242.19922	52.36084	31.4960
2	45.091	MM	1.1185	2152.78516	32.07850	20.9130
3	71.731	MM	1.6665	2883.39941	28.83657	28.0105
4	76.638	MM	1.8445	2015.61963	18.21255	19.5805

Totals : 1.02940e4 131.48845

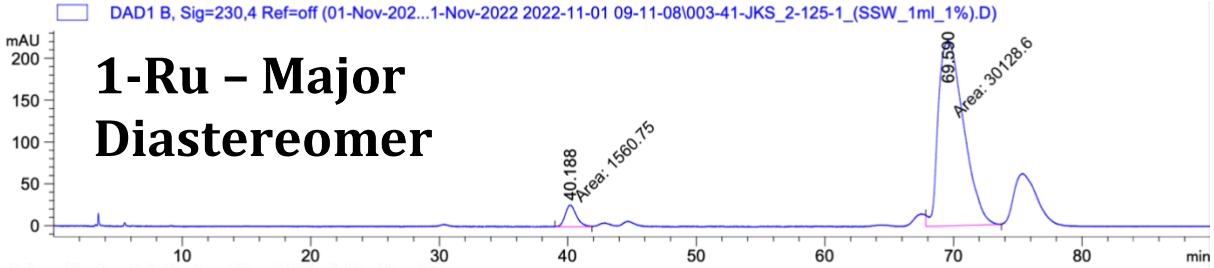

Signal 2: DAD1 B, Sig=230,4 Ref=off

Peak #	RetTime [min]	Type	Width [min]	Area [mAU*s]	Height [mAU]	Area %
1	40.188	MM	1.0053	1560.75403	25.87547	4.9252
2	69.590	MM	2.2661	3.01286e4	221.58920	95.0748

Totals : 3.16893e4 247.46467

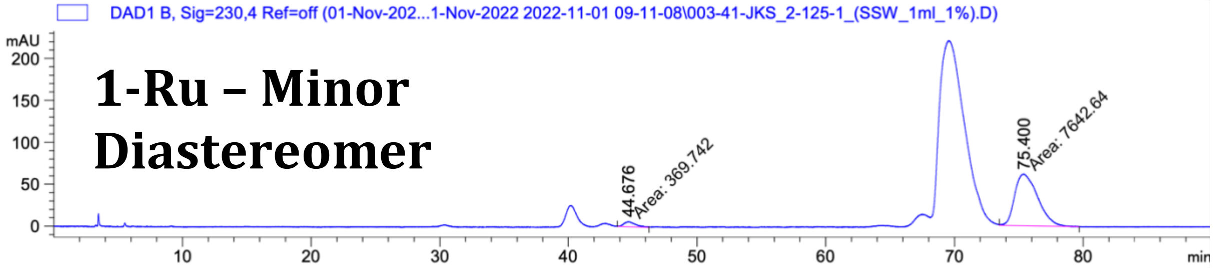

Signal 2: DAD1 B, Sig=230,4 Ref=off

Peak #	RetTime [min]	Type	Width [min]	Area [mAU*s]	Height [mAU]	Area %
1	44.676	MM	0.9953	369.74167	6.19154	4.6146
2	75.400	MM	2.0623	7642.63623	61.76421	95.3854

Totals : 8012.37790 67.95575

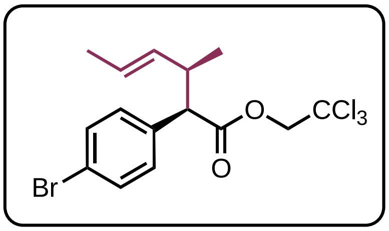

Compound **22**

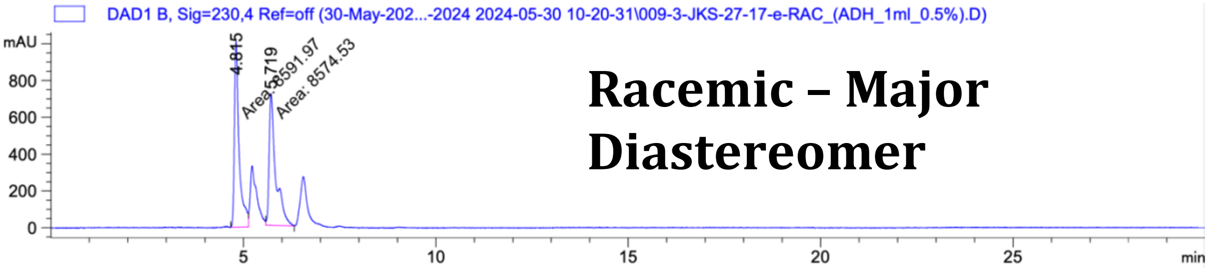

Racemic – Major Diastereomer

Signal 2: DAD1 B, Sig=230,4 Ref=off

Peak #	RetTime [min]	Type	Width [min]	Area [mAU*s]	Height [mAU]	Area %
1	4.815	MM	0.1422	8591.96875	1007.25586	50.0508
2	5.719	MM	0.1995	8574.52637	716.43311	49.9492

Totals : 1.71665e4 1723.68896

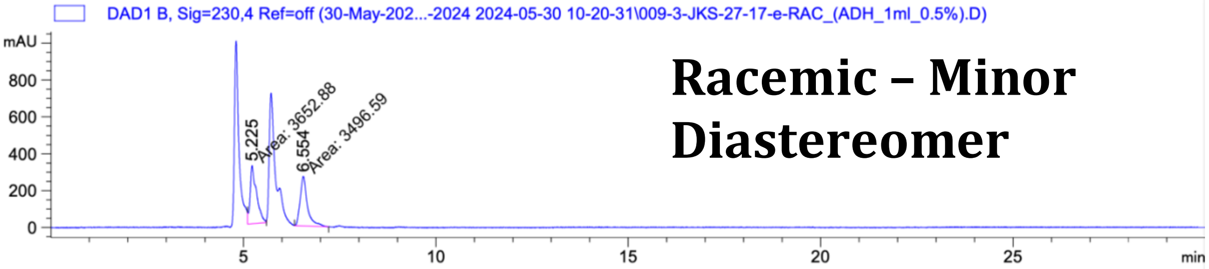

Racemic – Minor Diastereomer

Signal 2: DAD1 B, Sig=230,4 Ref=off

Peak #	RetTime [min]	Type	Width [min]	Area [mAU*s]	Height [mAU]	Area %
1	5.225	MM	0.1929	3652.88403	315.57245	51.0931
2	6.554	MM	0.2168	3496.58521	268.85019	48.9069

Totals : 7149.46924 584.42264

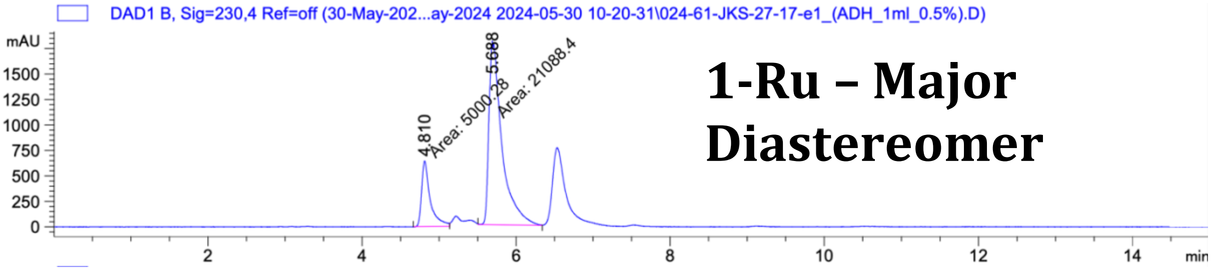

1-Ru – Major Diastereomer

Signal 2: DAD1 B, Sig=230,4 Ref=off

Peak #	RetTime [min]	Type	Width [min]	Area [mAU*s]	Height [mAU]	Area %
1	4.810	MM	0.1295	5000.28125	643.57147	19.1665
2	5.688	MM	0.1947	2.10884e4	1805.57666	80.8335

Totals : 2.60887e4 2449.14813

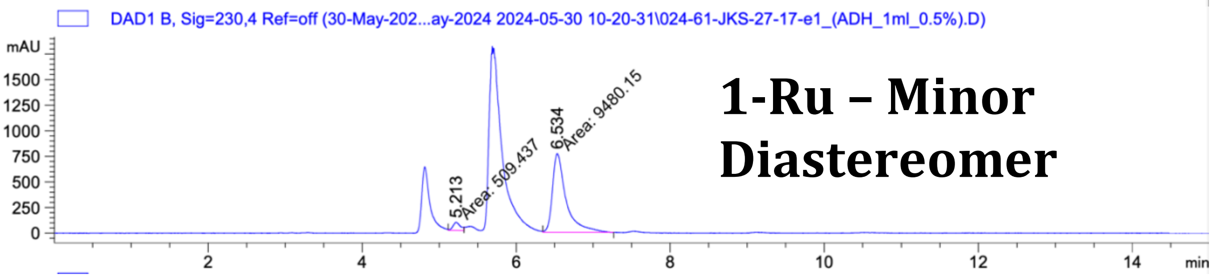

1-Ru – Minor Diastereomer

Signal 2: DAD1 B, Sig=230,4 Ref=off

Peak #	RetTime [min]	Type	Width [min]	Area [mAU*s]	Height [mAU]	Area %
1	5.213	MM	0.1101	509.43701	77.09387	5.0997
2	6.534	MM	0.2052	9480.14844	770.14447	94.9003
Totals :				9989.58545	847.23834	

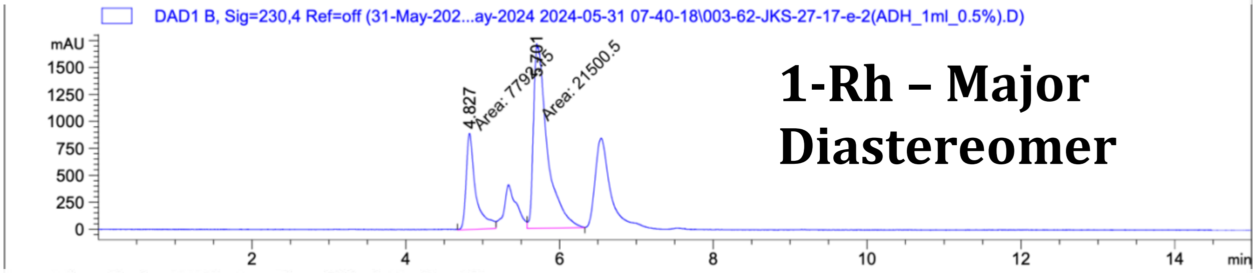

Signal 2: DAD1 B, Sig=230,4 Ref=off

Peak #	RetTime [min]	Type	Width [min]	Area [mAU*s]	Height [mAU]	Area %
1	4.827	MM	0.1451	7792.15430	894.80145	26.6010
2	5.701	MM	0.2091	2.15005e4	1714.09851	73.3990
Totals :				2.92927e4	2608.89996	

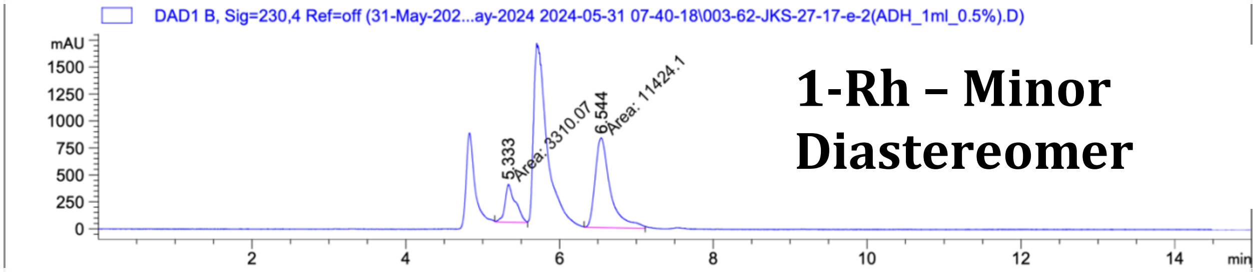

Signal 2: DAD1 B, Sig=230,4 Ref=off

Peak #	RetTime [min]	Type	Width [min]	Area [mAU*s]	Height [mAU]	Area %
1	5.333	MM	0.1571	3310.06958	351.16541	22.4653
2	6.544	MM	0.2290	1.14241e4	831.31274	77.5347
Totals :				1.47342e4	1182.47815	

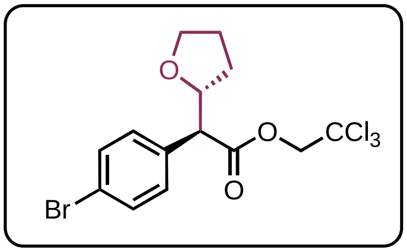

Compound **23**

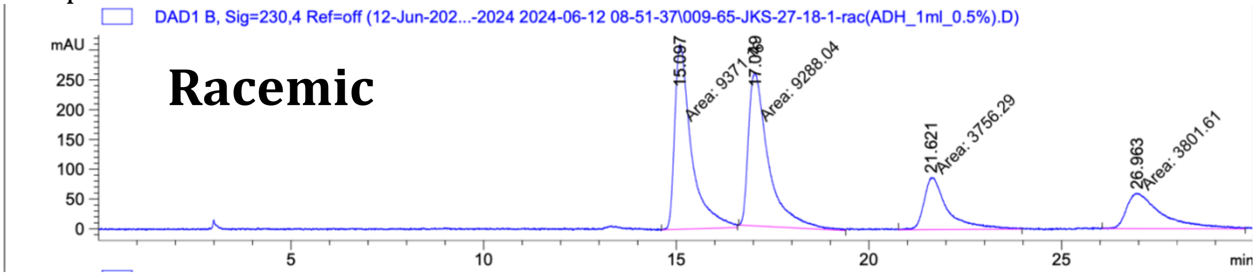

Signal 2: DAD1 B, Sig=230,4 Ref=off

Peak #	RetTime [min]	Type	Width [min]	Area [mAU*s]	Height [mAU]	Area %
1	15.097	MM	0.5031	9371.16113	310.42740	35.7444
2	17.049	MM	0.6026	9288.04492	256.87198	35.4274
3	21.621	MM	0.7109	3756.29053	88.06490	14.3276
4	26.963	MM	1.0703	3801.61182	59.19608	14.5005
Totals :				2.62171e4	714.56035	

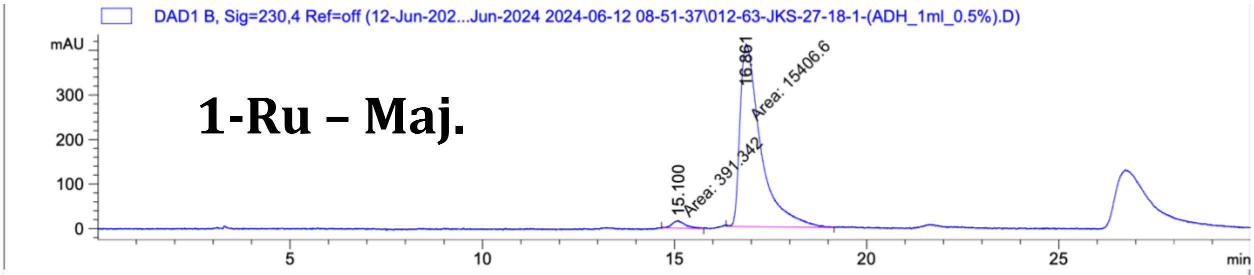

Signal 2: DAD1 B, Sig=230,4 Ref=off

Peak #	RetTime [min]	Type	Width [min]	Area [mAU*s]	Height [mAU]	Area %
1	15.100	MM	0.3686	391.34244	17.69704	2.4772
2	16.861	MM	0.6254	1.54066e4	410.60364	97.5228
Totals :				1.57980e4	428.30068	

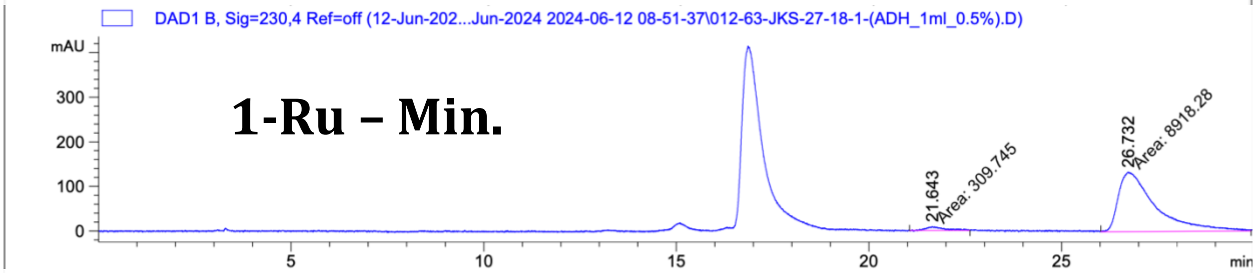

Signal 2: DAD1 B, Sig=230,4 Ref=off

Peak #	RetTime [min]	Type	Width [min]	Area [mAU*s]	Height [mAU]	Area %
1	21.643	MM	0.5684	309.74539	9.08172	3.3566
2	26.732	MM	1.1137	8918.27930	133.45743	96.6434
Totals :				9228.02469	142.53915	

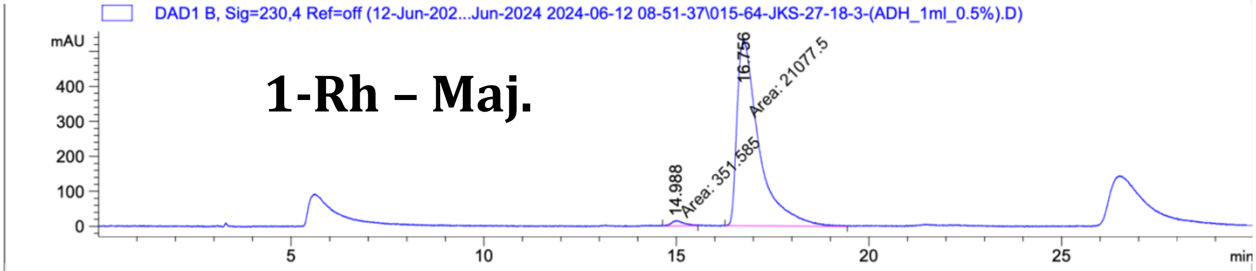

Signal 2: DAD1 B, Sig=230,4 Ref=off

Peak #	RetTime [min]	Type	Width [min]	Area [mAU*s]	Height [mAU]	Area %
1	14.988	MM	0.3787	351.58496	15.47485	1.6407
2	16.756	MM	0.6635	2.10775e4	529.42053	98.3593
Totals :				2.14291e4	544.89539	

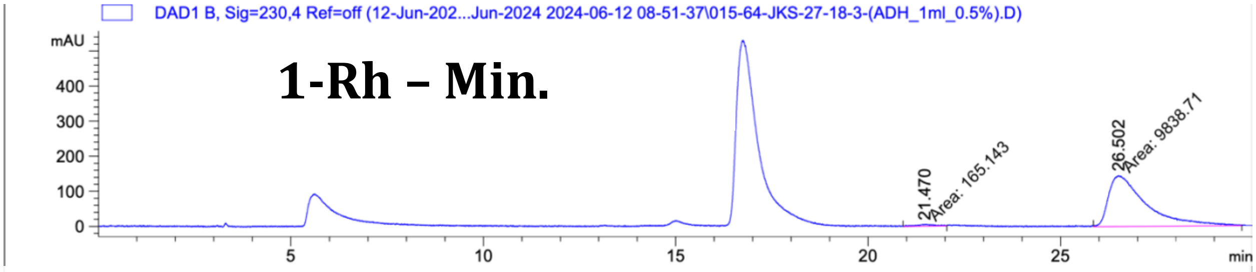

Signal 2: DAD1 B, Sig=230,4 Ref=off

Peak #	RetTime [min]	Type	Width [min]	Area [mAU*s]	Height [mAU]	Area %
1	21.470	MM	0.5203	165.14310	5.29025	1.6508
2	26.502	MM	1.1258	9838.71289	145.65511	98.3492

Totals : 1.00039e4 150.94535

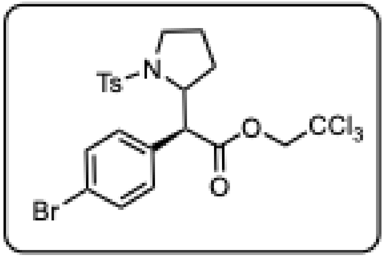

Compound **24**

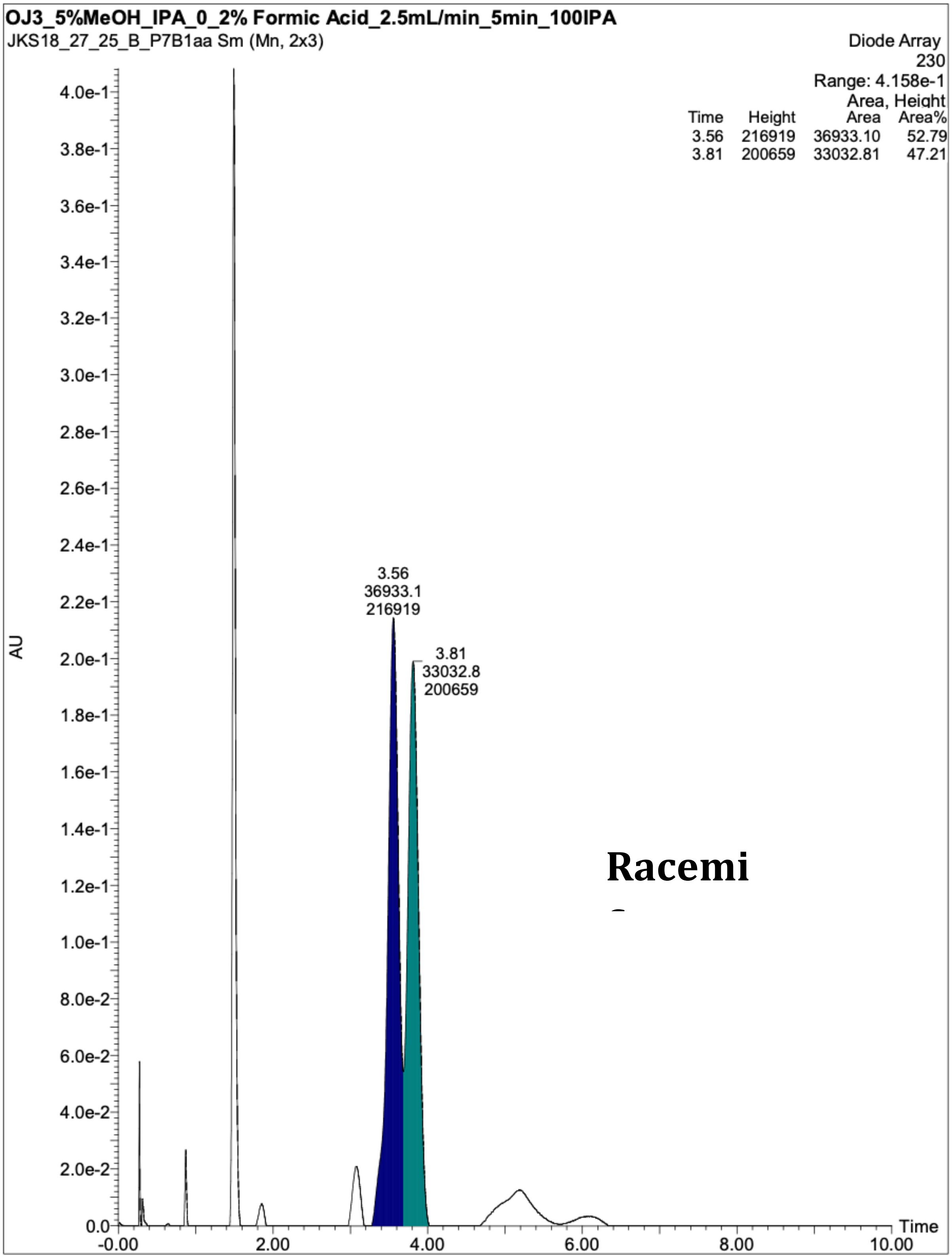
OJ3_5%MeOH_IPA_0_2% Formic Acid_2.5mL/min_5min_100IPA
JKS18_27_25_B_P7B1aa Sm (Mn, 2x3)
Diode Array
230
Range: 4.158e-1
Area, Height
Time Height Area Area%
3.56 216919 36933.10 52.79
3.81 200659 33032.81 47.21
3.56
36933.1
216919
3.81
33032.8
200659
AU
Time
Racemi

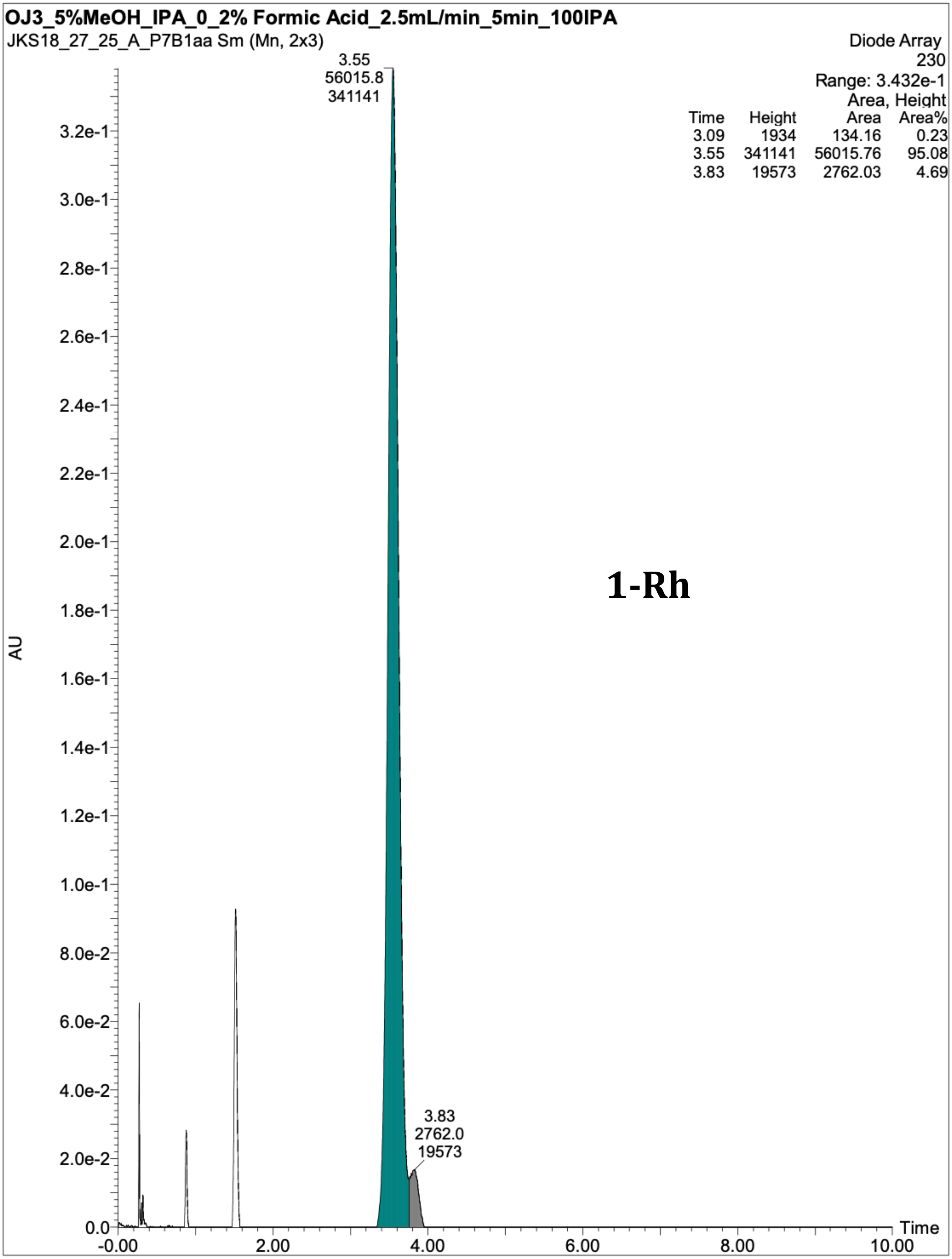
OJ3_5%MeOH_IPA_0_2% Formic Acid_2.5mL/min_5min_100IPA
JKS18_27_25_A_P7B1aa Sm (Mn, 2x3)
Diode Array
230
Range: 3.432e-1
Area, Height
Time Height Area Area%
3.09 1934 134.16 0.23
3.55 341141 56015.76 95.08
3.83 19573 2762.03 4.69
3.55
56015.8
341141
3.83
2762.0
19573
1-Rh
AU
Time
3.2e-1
3.0e-1
2.8e-1
2.6e-1
2.4e-1
2.2e-1
2.0e-1
1.8e-1
1.6e-1
1.4e-1
1.2e-1
1.0e-1
8.0e-2
6.0e-2
4.0e-2
2.0e-2
0.0
-0.00
2.00
4.00
6.00
8.00
10.00

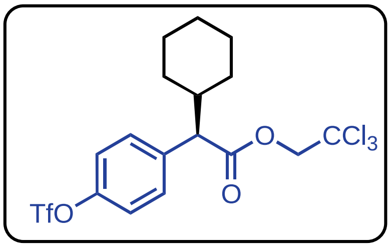

Compound **25**

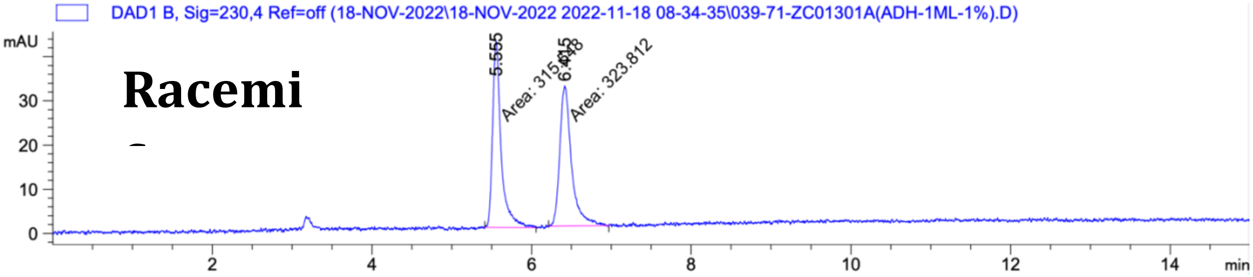

Signal 2: DAD1 B, Sig=230,4 Ref=off

Peak #	RetTime [min]	Type	Width [min]	Area [mAU*s]	Height [mAU]	Area %
1	5.555	MM	0.1249	315.54758	42.11271	49.3537
2	6.415	MM	0.1702	323.81174	31.70454	50.6463
Totals :				639.35931	73.81725	

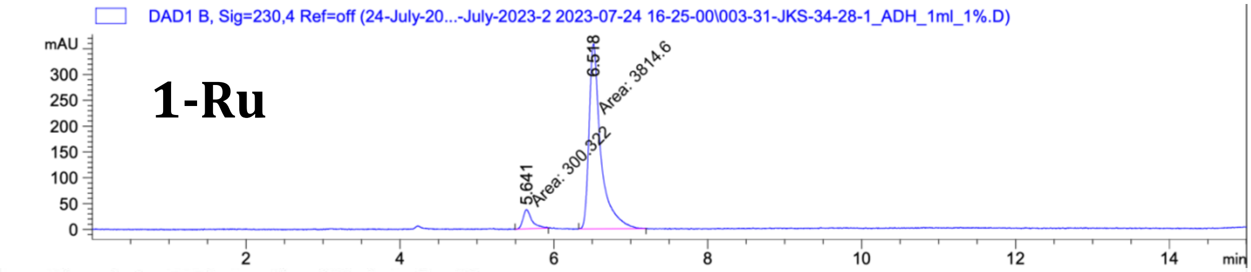

Signal 2: DAD1 B, Sig=230,4 Ref=off

Peak #	RetTime [min]	Type	Width [min]	Area [mAU*s]	Height [mAU]	Area %
1	5.641	MM	0.1346	300.32205	37.19786	7.2984
2	6.518	MM	0.1773	3814.59692	358.64355	92.7016
Totals :				4114.91898	395.84142	

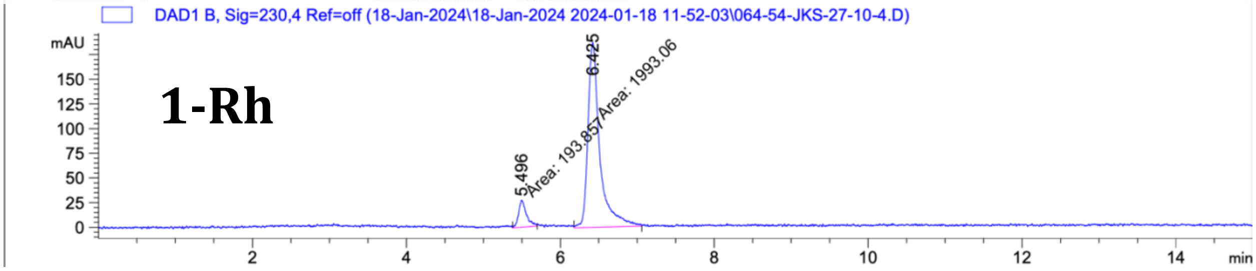

Signal 2: DAD1 B, Sig=230,4 Ref=off

Peak #	RetTime [min]	Type	Width [min]	Area [mAU*s]	Height [mAU]	Area %
1	5.496	MM	0.1186	193.85712	27.25322	8.8644
2	6.425	MM	0.1774	1993.05518	187.23053	91.1356
Totals :				2186.91229	214.48375	

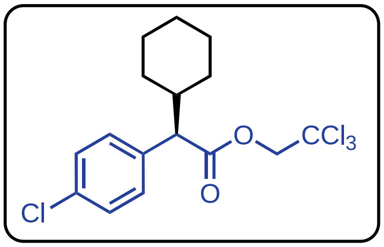

Compound **26**

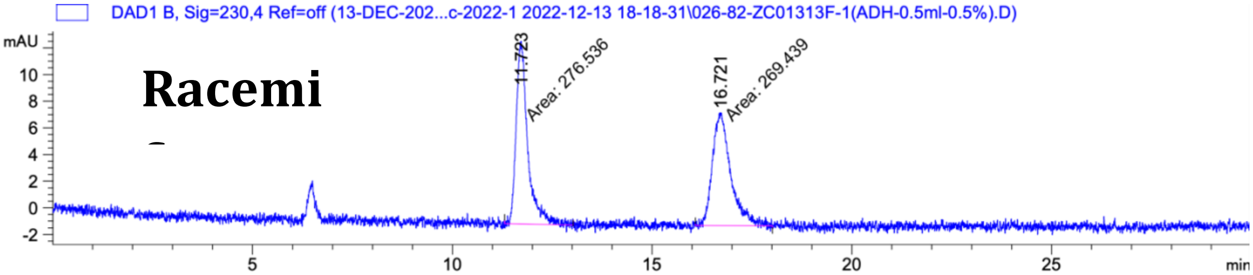

Signal 2: DAD1 B, Sig=230,4 Ref=off

Peak #	RetTime [min]	Type	Width [min]	Area [mAU*s]	Height [mAU]	Area %
1	11.723	MM	0.3359	276.53616	13.72200	50.6499
2	16.721	MM	0.5299	269.43939	8.47415	49.3501
Totals :				545.97556	22.19615	

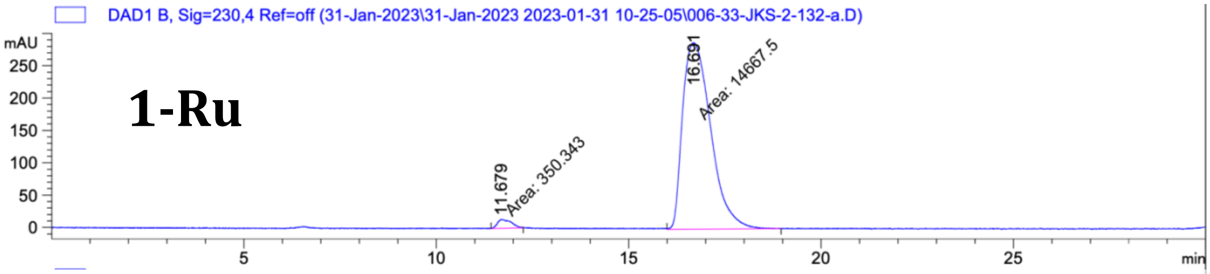

Signal 2: DAD1 B, Sig=230,4 Ref=off

Peak #	RetTime [min]	Type	Width [min]	Area [mAU*s]	Height [mAU]	Area %
1	11.679	MM	0.4140	350.34320	14.10548	2.3328
2	16.691	MM	0.8474	1.46675e4	288.48712	97.6672
Totals :				1.50179e4	302.59260	

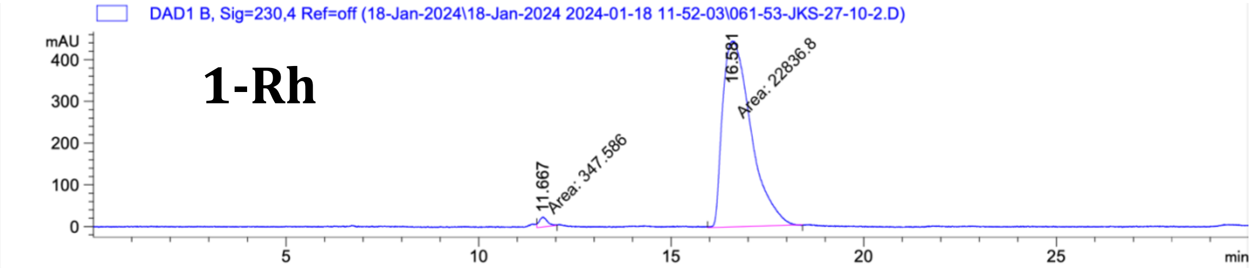

Signal 2: DAD1 B, Sig=230,4 Ref=off

Peak #	RetTime [min]	Type	Width [min]	Area [mAU*s]	Height [mAU]	Area %
1	11.667	MM	0.2409	347.58630	24.05255	1.4992
2	16.581	MM	0.8543	2.28368e4	445.53268	98.5008
Totals :				2.31844e4	469.58524	

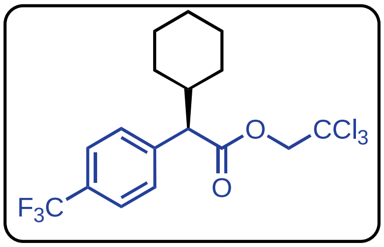

Compound **27**

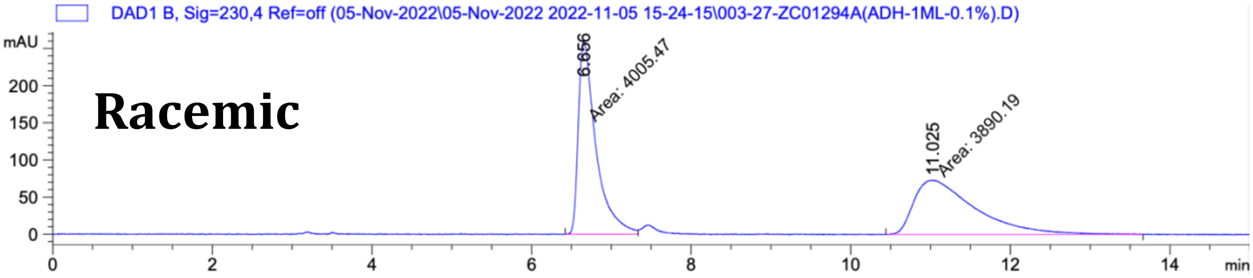

Signal 2: DAD1 B, Sig=230,4 Ref=off

Peak #	RetTime [min]	Type	Width [min]	Area [mAU*s]	Height [mAU]	Area %
1	6.656	MF	0.2576	4005.46729	259.15402	50.7300
2	11.025	MM	0.8841	3890.19043	73.33485	49.2700
Totals :				7895.65771	332.48887	

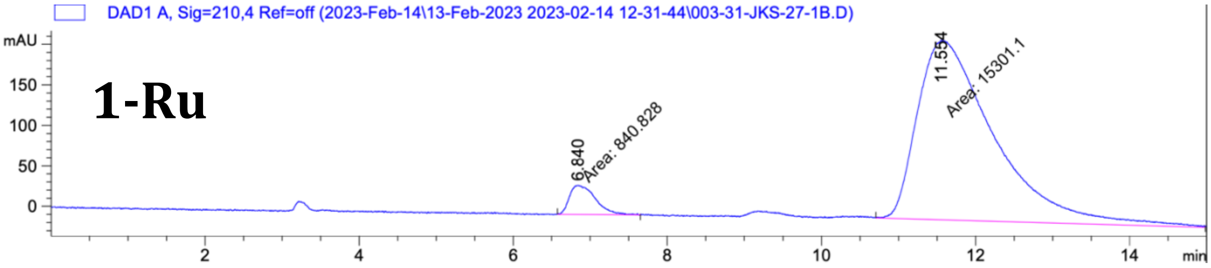

Signal 1: DAD1 A, Sig=210,4 Ref=off

Peak #	RetTime [min]	Type	Width [min]	Area [mAU*s]	Height [mAU]	Area %
1	6.840	MM	0.3892	840.82751	36.00511	5.2090
2	11.554	MM	1.1493	1.53011e4	221.90002	94.7910

Totals : 1.61419e4 257.90513

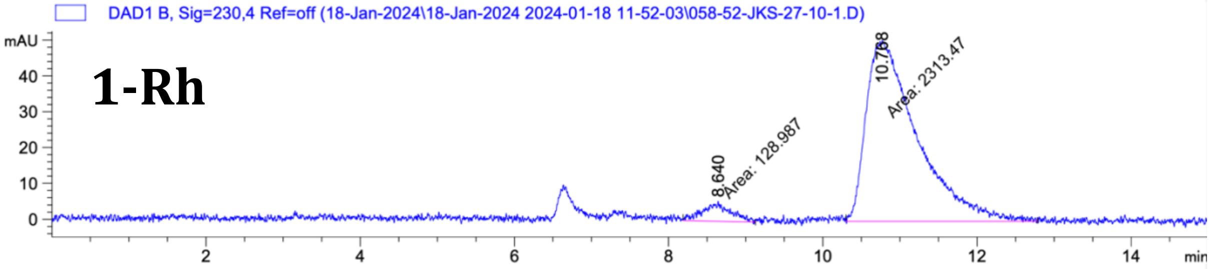

Signal 2: DAD1 B, Sig=230,4 Ref=off

Peak #	RetTime [min]	Type	Width [min]	Area [mAU*s]	Height [mAU]	Area %
1	8.640	MM	0.3882	128.98727	5.53717	5.2811
2	10.768	MM	0.7639	2313.46753	50.47333	94.7189

Totals : 2442.45480 56.01050

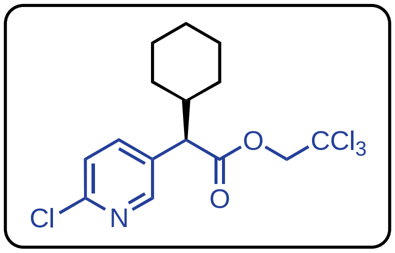

Compound **28**

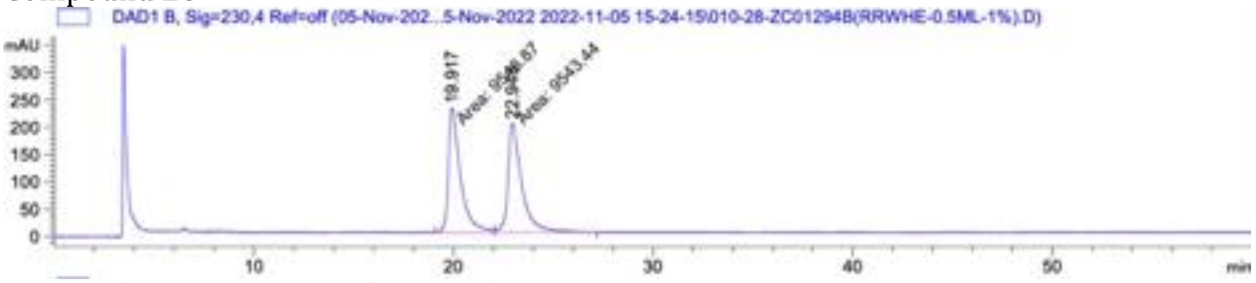

Signal 2: DAD1 B, Sig=230,4 Ref=off

Peak #	RetTime [min]	Type	Width [min]	Area [mAU*s]	Height [mAU]	Area %
1	19.917	MF	0.6999	9548.87305	227.39455	50.0142
2	22.943	FM	0.7978	9543.44238	199.35765	49.9858

Totals : 1.90923e4 426.75220

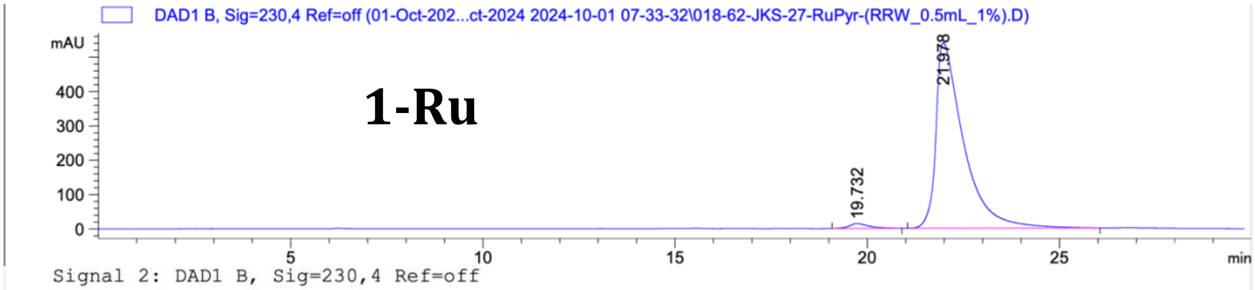

Signal 2: DAD1 B, Sig=230,4 Ref=off

Peak #	RetTime [min]	Type	Width [min]	Area [mAU*s]	Height [mAU]	Area %
1	19.732	BB	0.4238	501.34375	13.93367	1.8219
2	21.978	BB	0.6558	2.70161e4	539.90717	98.1781
Totals :				2.75174e4	553.84084	

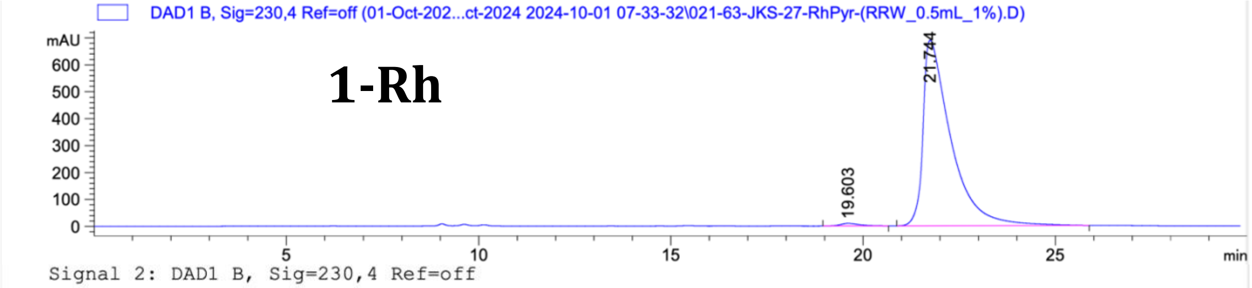

Signal 2: DAD1 B, Sig=230,4 Ref=off

Peak #	RetTime [min]	Type	Width [min]	Area [mAU*s]	Height [mAU]	Area %
1	19.603	BB	0.4089	332.29840	9.52656	0.9472
2	21.744	BB	0.6580	3.47506e4	688.86749	99.0528
Totals :				3.50829e4	698.39406	

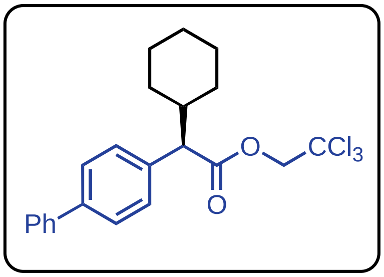

Compound **29**

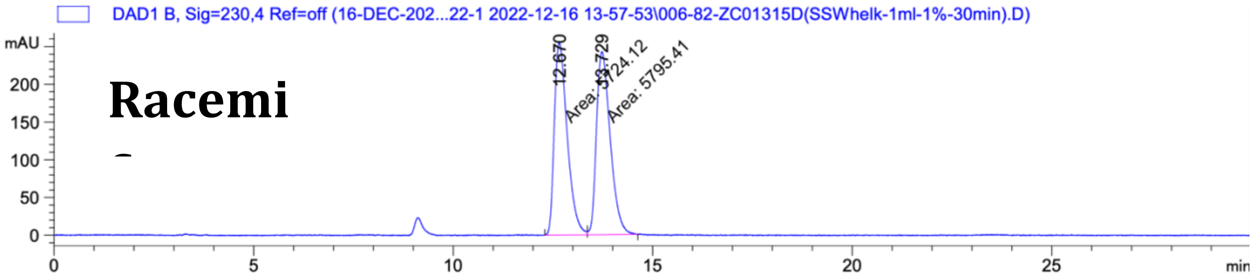

Signal 2: DAD1 B, Sig=230,4 Ref=off

Peak #	RetTime [min]	Type	Width [min]	Area [mAU*s]	Height [mAU]	Area %
1	12.670	MF	0.3746	5724.11621	254.69371	49.6905
2	13.729	FM	0.3983	5795.41357	242.49443	50.3095

Totals : 1.15195e4 497.18814

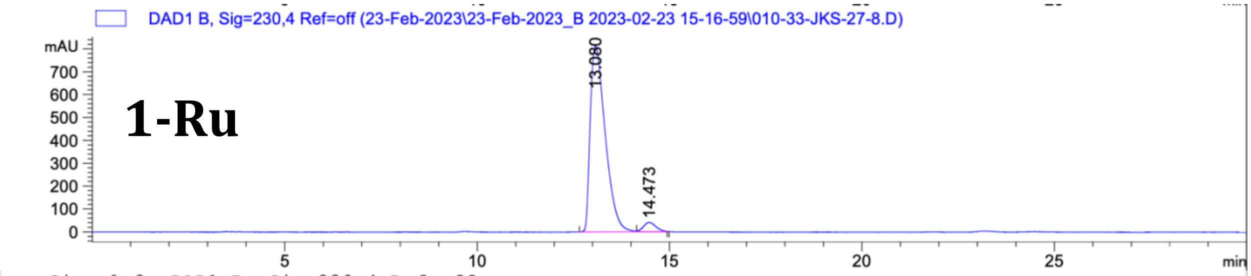

Signal 2: DAD1 B, Sig=230,4 Ref=off

Peak #	RetTime [min]	Type	Width [min]	Area [mAU*s]	Height [mAU]	Area %
1	13.080	VV R	0.3216	2.19960e4	811.79053	96.0871
2	14.473	VV E	0.2610	895.71912	40.27641	3.9129

Totals : 2.28917e4 852.06694

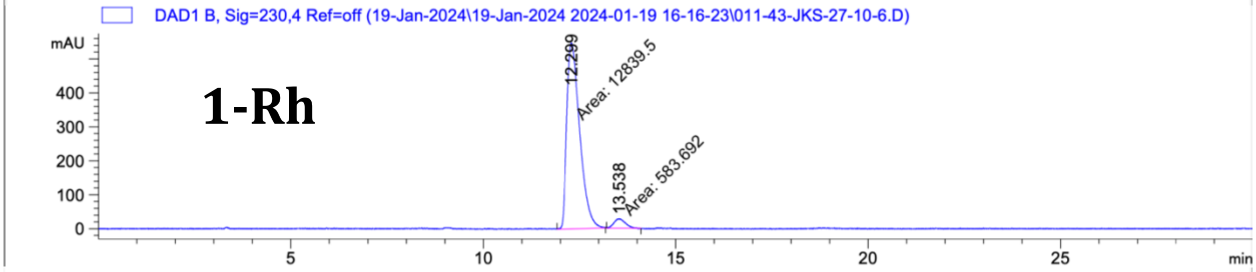

Signal 2: DAD1 B, Sig=230,4 Ref=off

Peak #	RetTime [min]	Type	Width [min]	Area [mAU*s]	Height [mAU]	Area %
1	12.299	MM	0.3906	1.28395e4	547.84998	95.6516
2	13.538	MM	0.3431	583.69165	28.35273	4.3484

Totals : 1.34232e4 576.20271

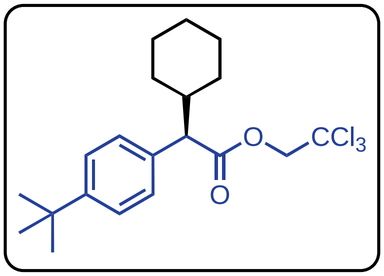

Compound **30**

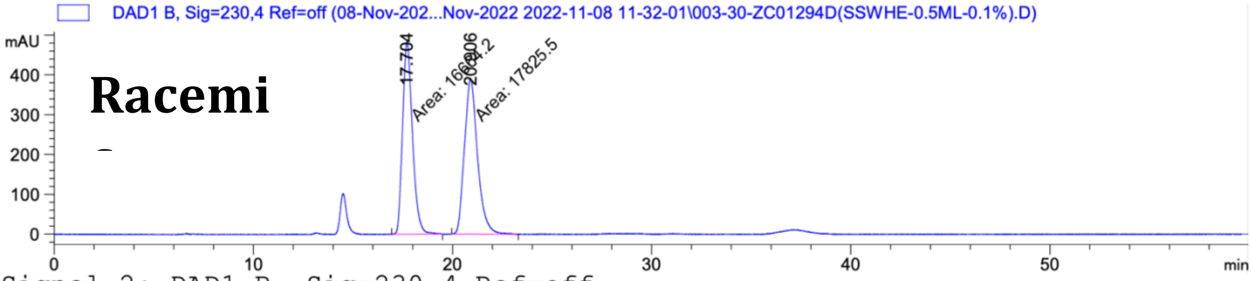

Signal 2: DAD1 B, Sig=230,4 Ref=off

Peak #	RetTime [min]	Type	Width [min]	Area [mAU*s]	Height [mAU]	Area %
1	17.704	MM	0.5737	1.66642e4	484.12158	48.3165
2	20.906	MM	0.7584	1.78255e4	391.71411	51.6835

Totals : 3.44897e4 875.83569

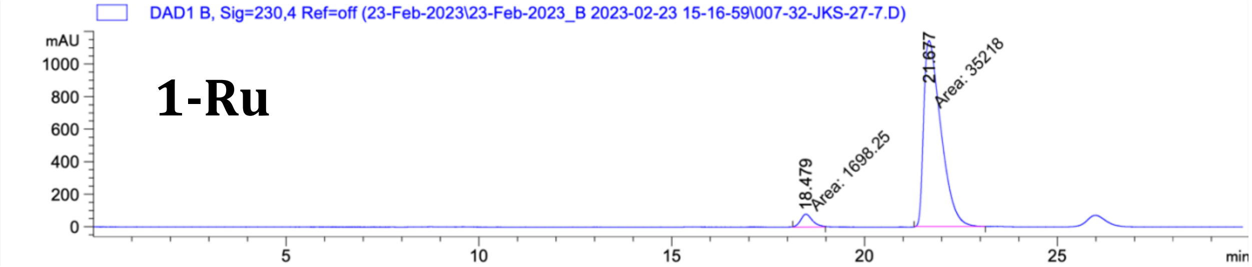

Signal 2: DAD1 B, Sig=230,4 Ref=off

Peak #	RetTime [min]	Type	Width [min]	Area [mAU*s]	Height [mAU]	Area %
1	18.479	MM	0.3558	1698.25134	79.55709	4.6003
2	21.677	MM	0.5137	3.52180e4	1142.72937	95.3997

Totals : 3.69163e4 1222.28646

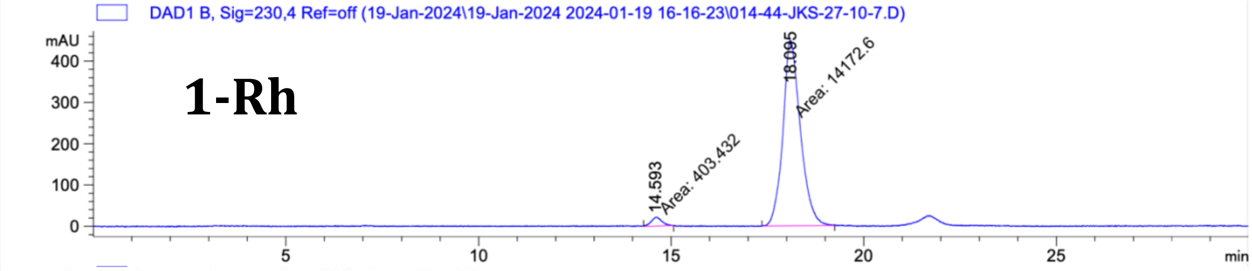

Signal 2: DAD1 B, Sig=230,4 Ref=off

Peak #	RetTime [min]	Type	Width [min]	Area [mAU*s]	Height [mAU]	Area %
1	14.593	MM	0.3174	403.43231	21.18424	2.7678
2	18.095	MM	0.5265	1.41726e4	448.67700	97.2322

Totals : 1.45760e4 469.86124

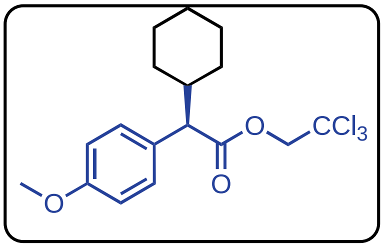

Compound **31**

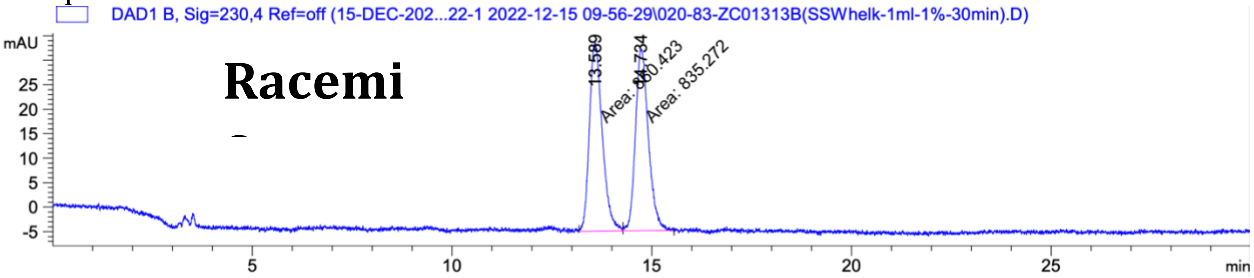

Signal 2: DAD1 B, Sig=230,4 Ref=off

Peak #	RetTime [min]	Type	Width [min]	Area [mAU*s]	Height [mAU]	Area %
1	13.589	MF	0.3730	860.42310	38.44910	50.7416
2	14.734	FM	0.3748	835.27246	37.14481	49.2584
Totals :				1695.69556	75.59391	

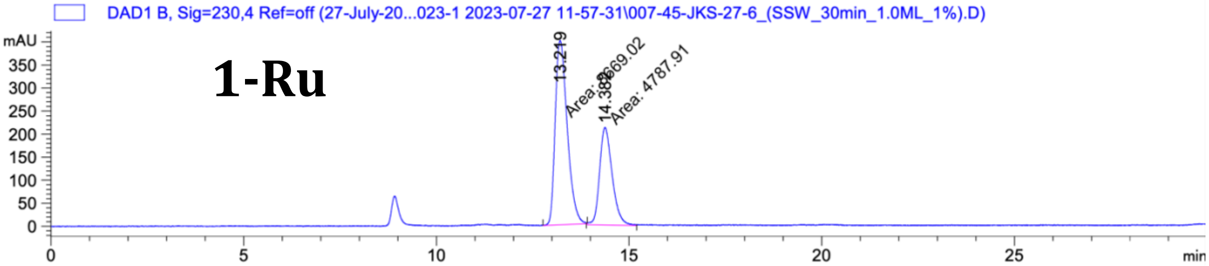

Signal 2: DAD1 B, Sig=230,4 Ref=off

Peak #	RetTime [min]	Type	Width [min]	Area [mAU*s]	Height [mAU]	Area %
1	13.219	MM	0.3608	8669.02051	400.43625	64.4205
2	14.382	MM	0.3765	4787.91064	211.92868	35.5795
Totals :				1.34569e4	612.36493	

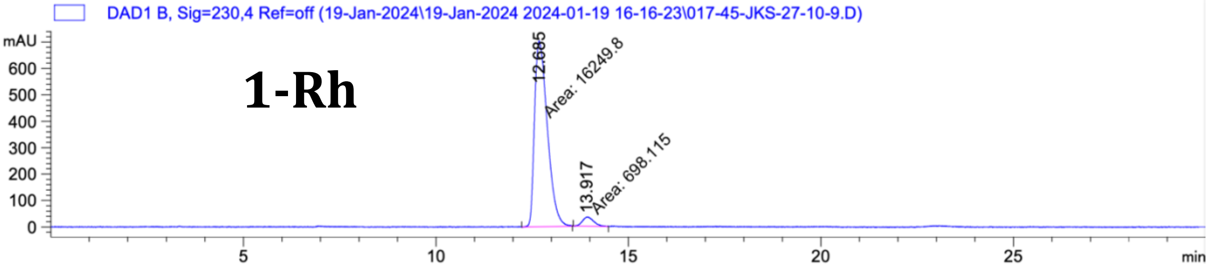

Signal 2: DAD1 B, Sig=230,4 Ref=off

Peak #	RetTime [min]	Type	Width [min]	Area [mAU*s]	Height [mAU]	Area %
1	12.685	MM	0.3842	1.62498e4	704.88867	95.8808
2	13.917	MM	0.3353	698.11475	34.69977	4.1192

Totals : 1.69479e4 739.58844

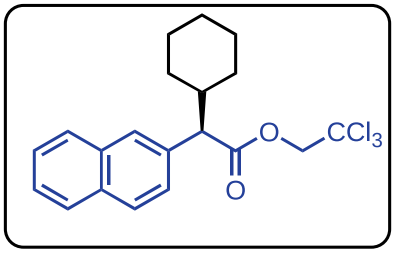

Compound **32**

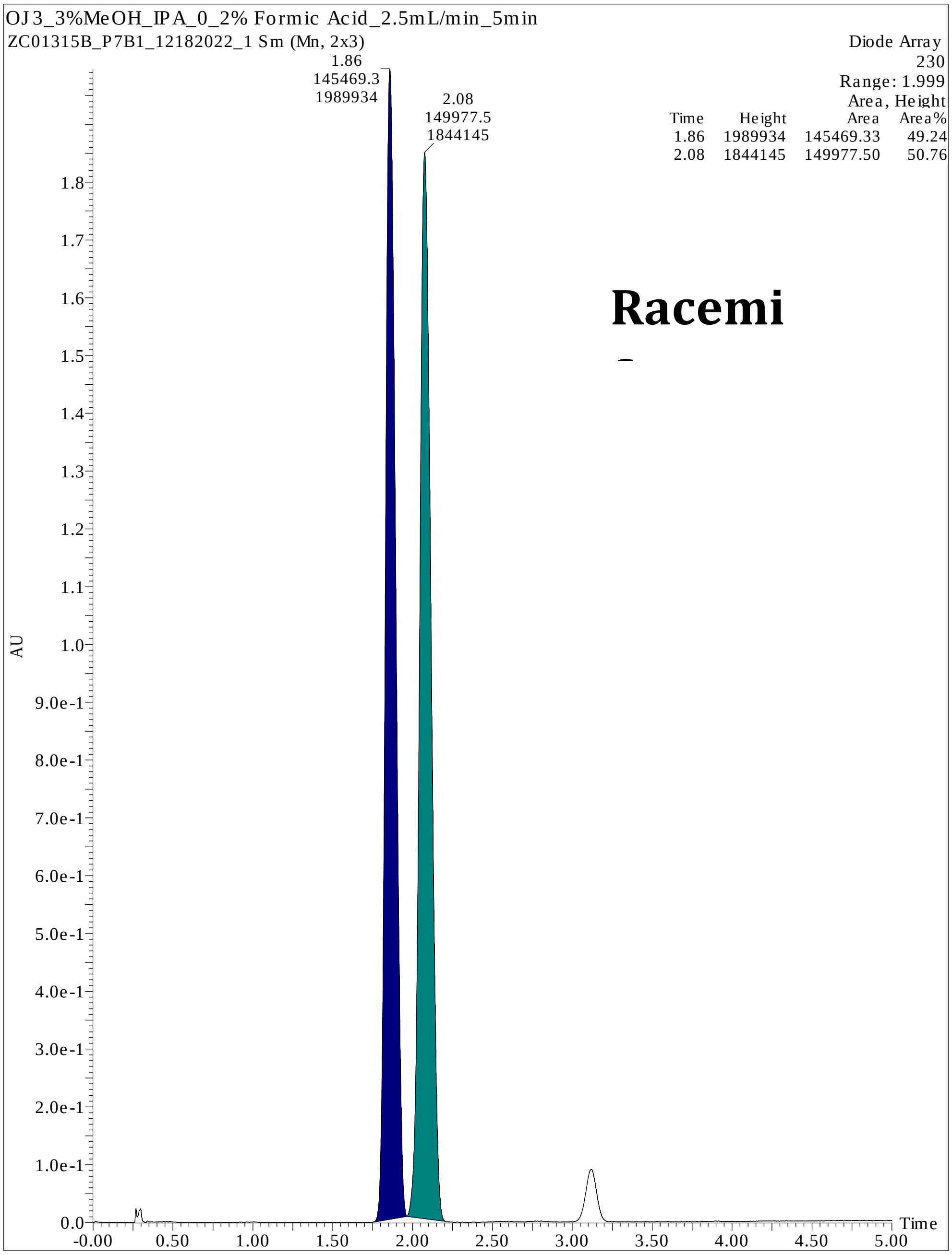
OJ3_3%MeOH_IPA_0_2% Formic Acid_2.5mL/min_5min
ZC01315B_P7B1_12182022_1 Sm (Mn, 2x3)
Diode Array
230
Range: 1.999
Area, Height
1.86
145469.3
1989934
2.08
149977.5
1844145
Time Height Area Area%
1.86 1989934 145469.33 49.24
2.08 1844145 149977.50 50.76
Racemi
AU
Time

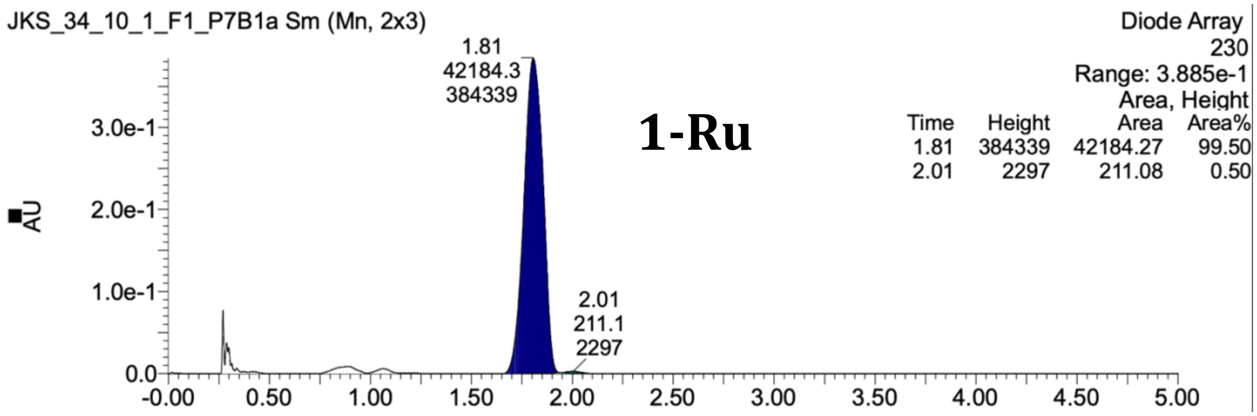

1-Ru

Time	Height	Area	Area%
1.81	384339	42184.27	99.50
2.01	2297	211.08	0.50

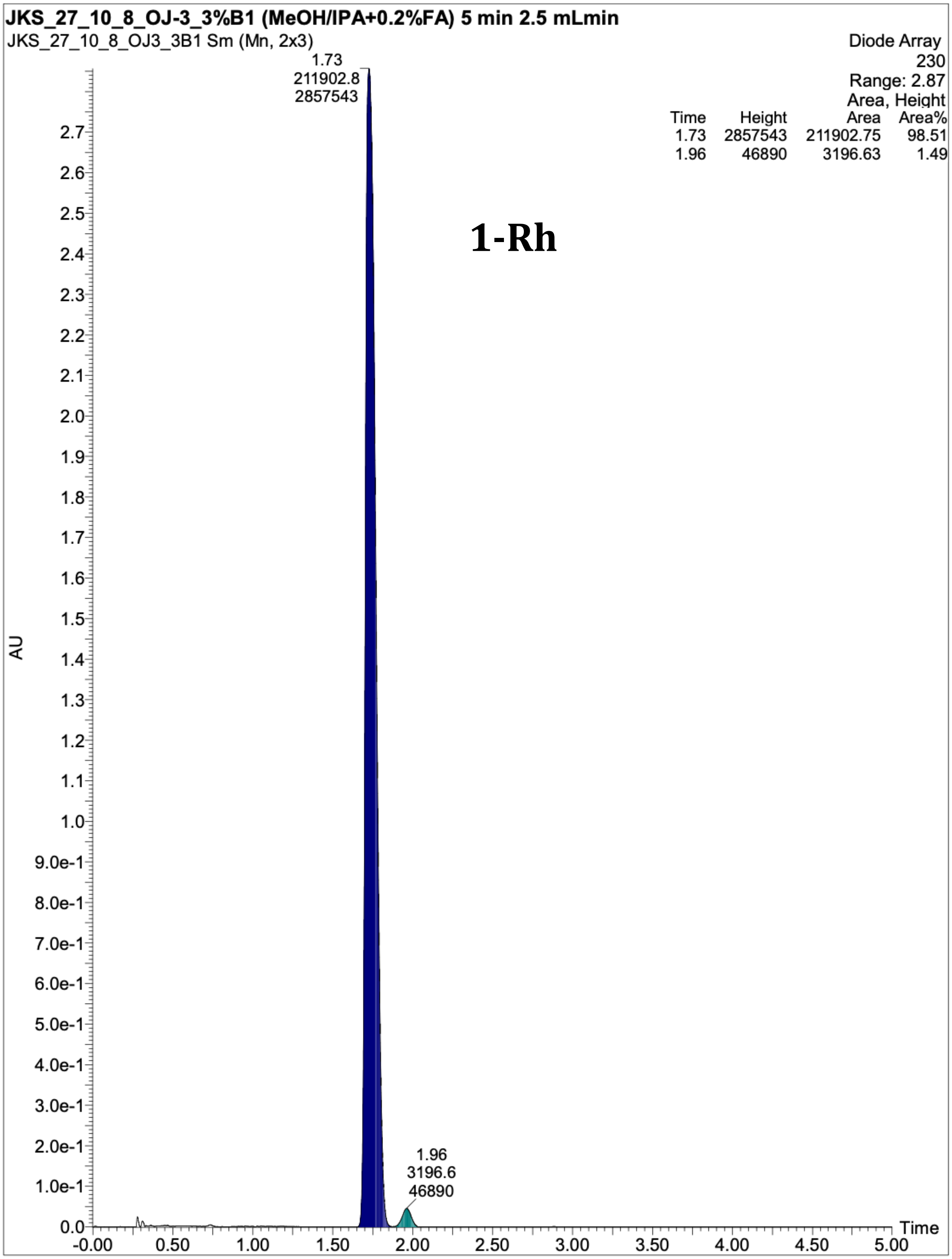
JKS_27_10_8_OJ-3_3%B1 (MeOH/IPA+0.2%FA) 5 min 2.5 mLmin
JKS_27_10_8_OJ3_3B1 Sm (Mn, 2x3)
Diode Array
230
Range: 2.87
Area, Height
Time Height Area Area%
1.73 2857543 211902.75 98.51
1.96 46890 3196.63 1.49
1-Rh
1.73
211902.8
2857543
1.96
3196.6
46890
AU
Time

Crystallographic Data

Ru2BTPCP4

EMORY UNIVERSITY | X-ray Crystallography Center

Submitted by: **Joshua Sailer, Davies Group**

Solved by: **John Bacsa**

***R₁*=5.13%**

Crystal Data and Experimental

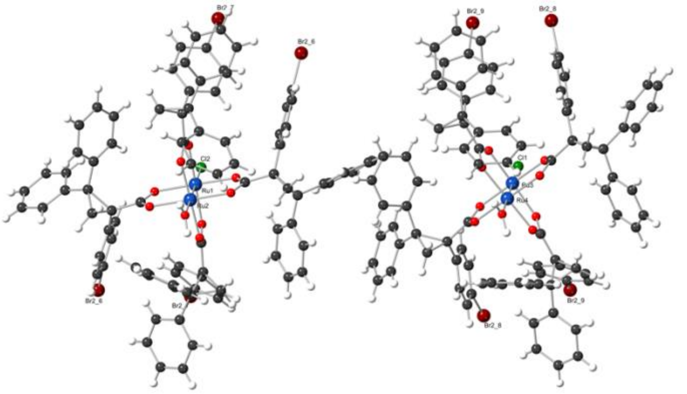

Experimental. Single orange plate-shaped crystals of Ru2BTPCP4 were chosen from the sample as supplied. A suitable crystal with dimensions 0.10 × 0.09 × 0.04 mm^3 was selected and mounted on a loop with paratone on a XtaLAB AFC11 (RCD3): quarter-chi single diffractometer. The crystal was kept at a steady T = 112(18) K during data collection. The structure was solved with the ShelXT (Sheldrick, 2015) solution program and by using Olex2 1.5-alpha (Dolomanov et al., 2009) as the graphical interface. The model was refined with ShelXL 2018/3 (Sheldrick, 2015) using full matrix least squares minimisation on $\boldsymbol{F}^2$.

Crystal Data. $C_{206}H_{194}Br_8Cl_{20}O_{18}Ru_4$, M_r = 4710.16, monoclinic, $P2$ (No. 3), a = 18.3811(3) Å, b = 10.26093(13) Å, c = 26.0244(4) Å, β = 90.1337(15)°, α = γ = 90°, V = 4908.36(14) Å^3, T = 112(18) K, Z = 1, Z' = 0.5, μ(Cu K_α) = 7.361, 89655 reflections measured, 15668 unique (R_{int} = 0.0835) which were used in all calculations. The final wR_2 was 0.1369 (all data) and R_1 was 0.0513 (I≥2 σ(I)).

**Compound**	Ru2BTPCP4
Formula	$C_{206}H_{194}Br_8Cl_{20}O_{18}Ru_4$
$D_{calc.}$/ g cm^{-3}	1.593
μ/mm^{-1}	7.361
Formula Weight	4710.16
Colour	orange
Shape	plate-shaped
Size/mm^3	0.10×0.09×0.04
T/K	112(18)
Crystal System	monoclinic
Flack Parameter	-0.006(6)
Hooft Parameter	-0.009(5)
Space Group	P2
a/Å	18.3811(3)
b/Å	10.26093(13)
c/Å	26.0244(4)
α/°	90
β/°	90.1337(15)
γ/°	90
V/Å^3	4908.36(14)
Z	1
Z'	0.5
Wavelength/Å	1.54184
Radiation type	Cu K_α
Θ_{min}/°	2.940
Θ_{max}/°	65.085
Measured Refl's.	89655
Indep't Refl's	15668
Refl's I≥2 σ(I)	12696
R_{int}	0.0835
Parameters	1109
Restraints	1407
Largest Peak	1.080
Deepest Hole	-0.725
GooF	1.077
wR_2 (all data)	0.1369
wR_2	0.1294
R_1 (all data)	0.0653
R_1	0.0513

Structure Quality Indicators

**Reflections:**	d min (CuKα) 2Θ=130.2° 0.85	I/σ(I) 16.6	Rint m=5.72 8.35%	Full 130.2° 98.1	
**Refinement:**	Shift -0.001	Max Peak 1.1	Min Peak -0.7	GooF 1.077	Hooft -.009(5)

An orange plate-shaped-shaped crystal with dimensions 0.10 × 0.09 × 0.04 mm^3 was mounted on a loop with paratone. Data were collected using a XtaLAB AFC11 (RCD3): quarter-chi single diffractometer equipped with an Oxford Cryosystems low-temperature device operating at T = 112(18) K.

Data were measured using ω scans with Cu K_α radiation. The diffraction pattern was indexed and the total number of runs and images was based on the strategy calculation from the program CrysAlisPro system (CCD 42.72a (release 07-10-2022)). The maximum resolution that was achieved was Θ = 65.085° (0.85 Å).

The unit cell was refined using CrysAlisPro 1.171.43.121a (Rigaku OD, 2024) on 27321 reflections, 30% of the observed reflections. Data reduction, scaling and absorption corrections were performed using CrysAlisPro 1.171.43.121a (Rigaku OD, 2024). The final completeness is 98.30 % out to 65.085° in Θ. A numerical absorption correction based on gaussian integration over a multifaceted crystal model was performed using CrysAlisPro 1.171.42.74a (Rigaku Oxford Diffraction, 2022). An empirical absorption correction using spherical harmonics, implemented in SCALE3 ABSPACK scaling algorithm was also applied. The absorption coefficient μ of this material is 7.361 mm^{-1} at this wavelength (λ = 1.54184Å) and the minimum and maximum transmissions are 0.641 and 0.844.

The structure was solved and the space group P2 (# 3) determined by the ShelXT (Sheldrick, 2015) structure solution program and refined by full matrix least squares minimisation on $\boldsymbol{F^2}$ using version 2018/3 of ShelXL 2018/3 (Sheldrick, 2015). All non-hydrogen atoms were refined anisotropically. Hydrogen atom positions were calculated geometrically and refined using the riding model.

The value of Z' is 0.5. This means that only half of the formula unit is present in the asymmetric unit, with the other half consisting of symmetry equivalent atoms. The moiety formula is 2(C88 H66 Br4 Cl O9 Ru2), 6(C H Cl3), C6 H14, 3[C6], 3[H14].

The Flack parameter was refined to -0.006(6). Determination of absolute structure using Bayesian statistics on Bijvoet differences using the Olex2 results in -0.009(5). The chiral atoms in this structure are: C2(S), C2(S), C2(S), C2(S). Note: The Flack parameter is used to determine chirality of the crystal studied, the value should be near 0, a value of 1 means that the stereochemistry is wrong and the model should be inverted. A value of 0.5 means that the crystal consists of a racemic mixture of the two enantiomers.

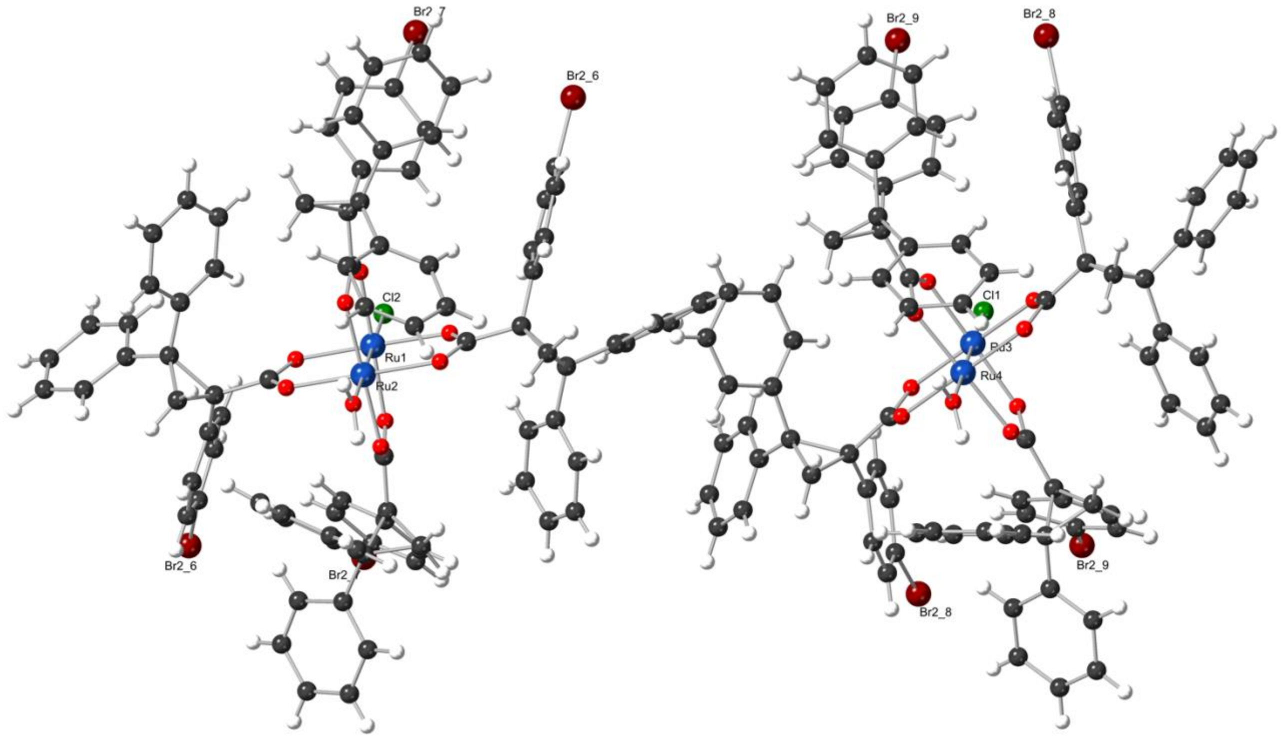

Figure 1 The asymmetric unit contains two Ru2-complexes positioned on the two-fold rotation axes, and the second halves of the molecules are generated by the 2-fold rotations. The complexes have perfect C2 symmetry. Solvent molecules (chloroform, ordered and disorder hexane (treated using SQUEEZE)) are not shown here.

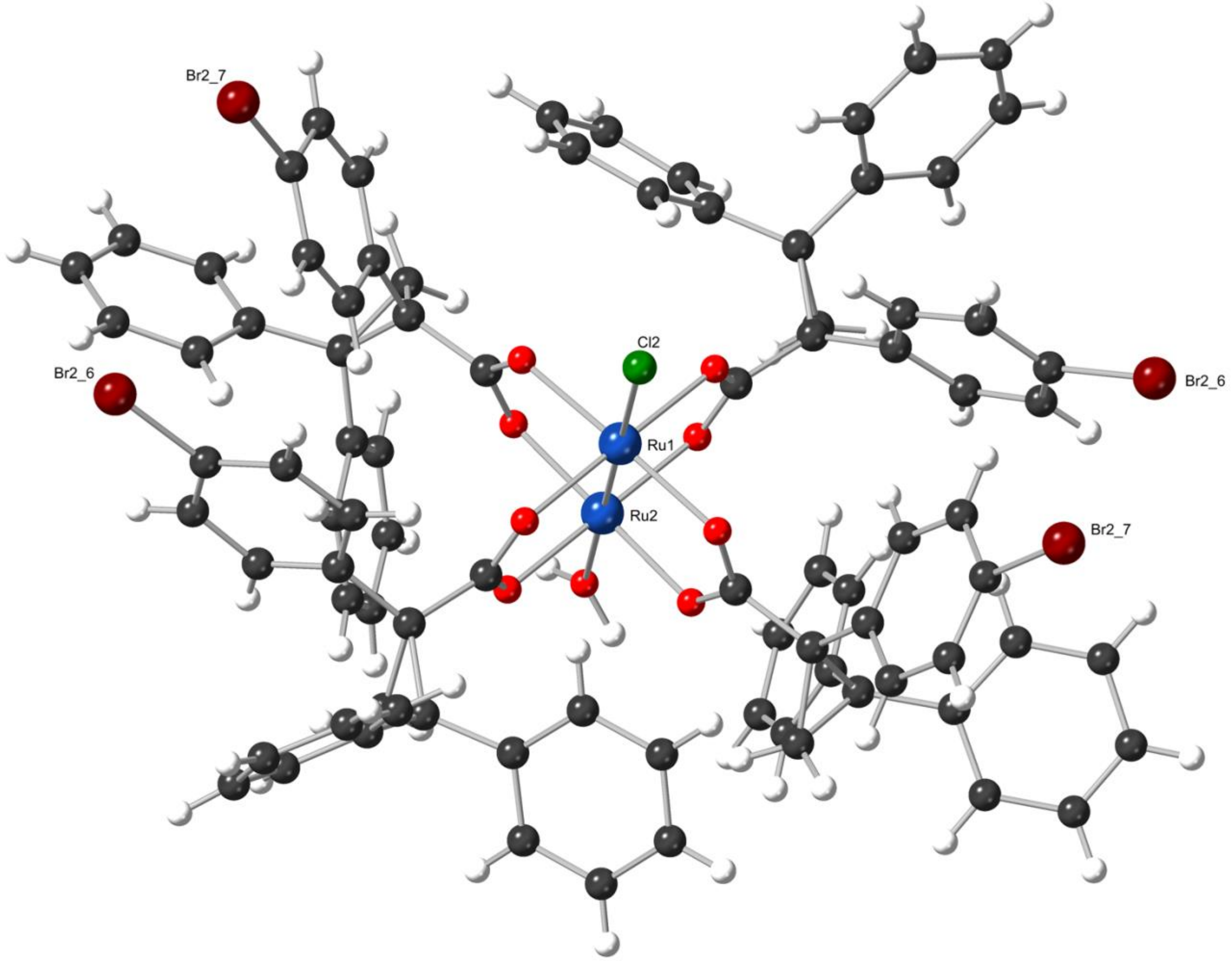

Figure 2 One of the two Ru2-complexes. Half of the atoms are unique, but the other half are generated by the two-fold rotation operation. The crystallographic symmetry enforces perfect C2 symmetry.

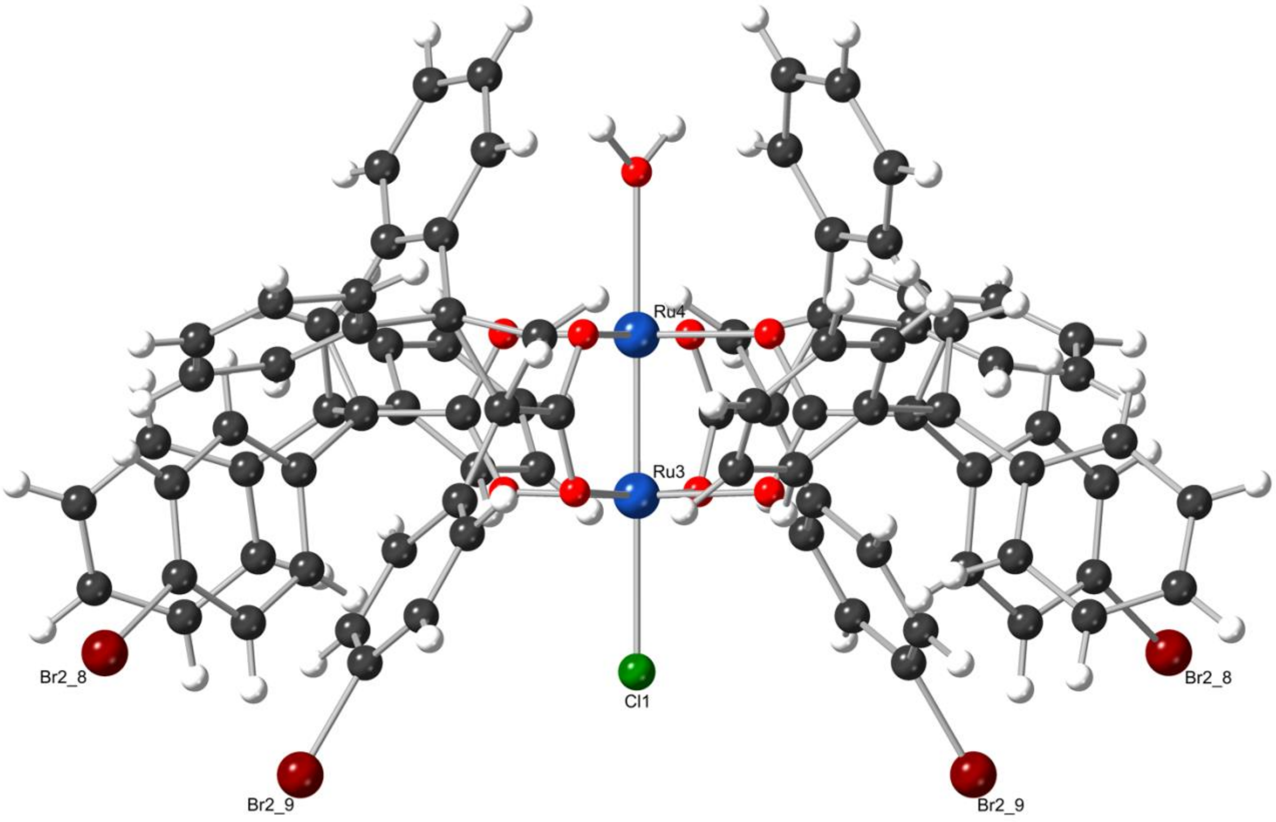

Figure 3 Perspective of one of the two Ru2-complexes showing the perfect C2 symmetry

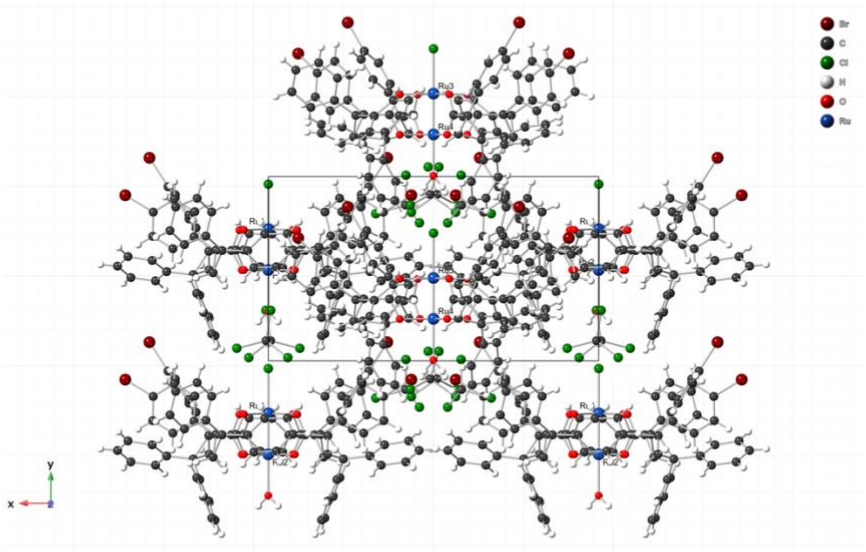

Figure 4 Molecular packing

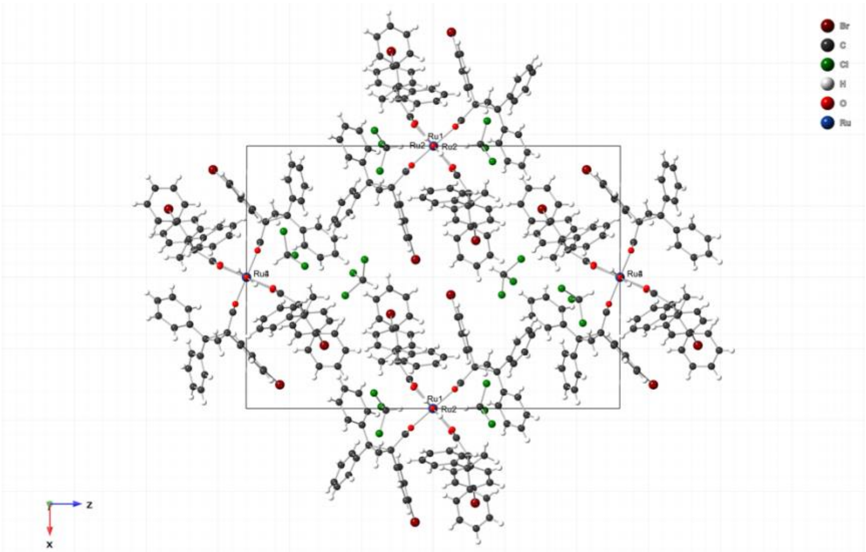

Figure 5

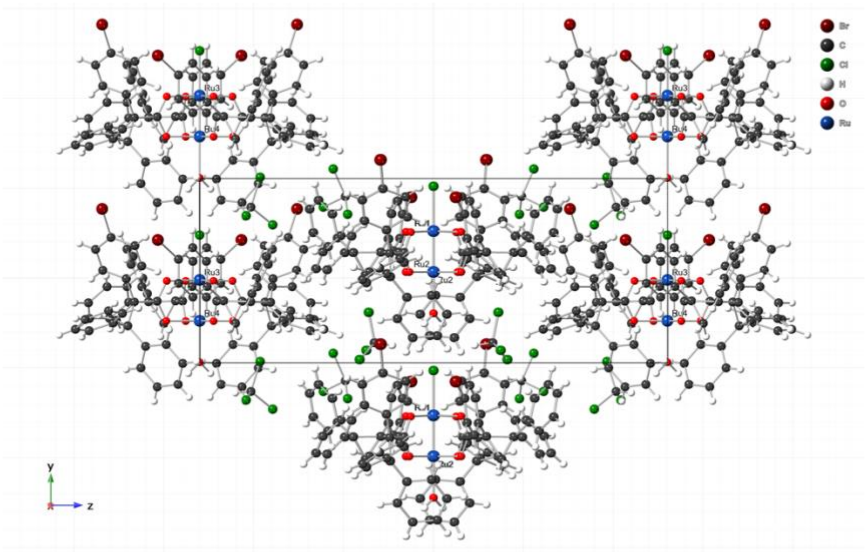

Figure 6

Data Plots: Diffraction Data

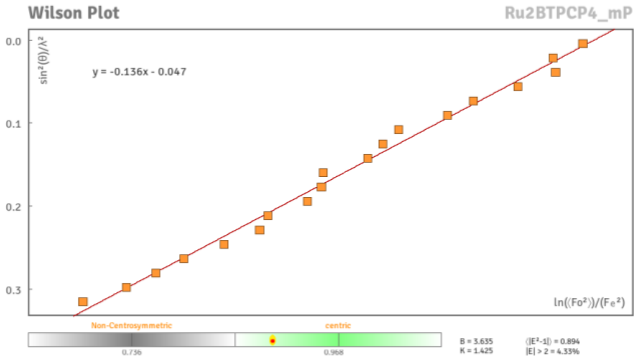

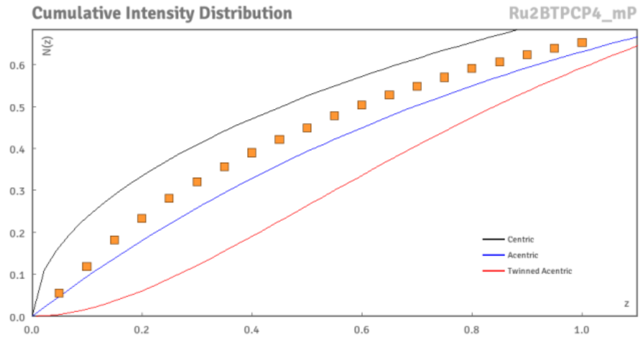

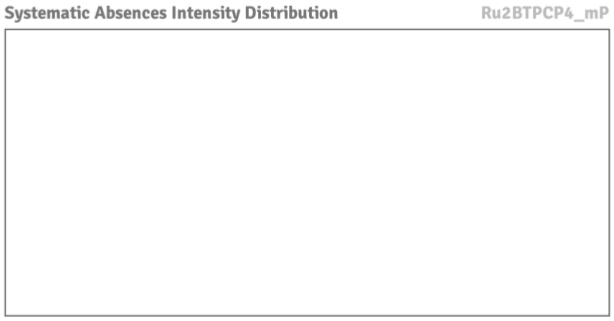

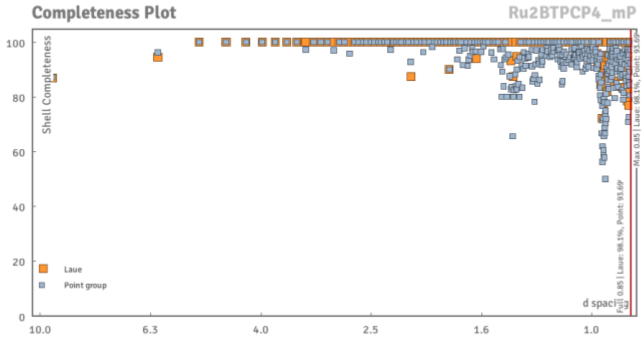

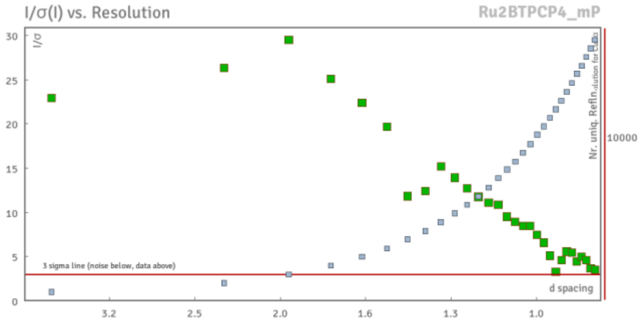

Data Plots: Refinement and Data

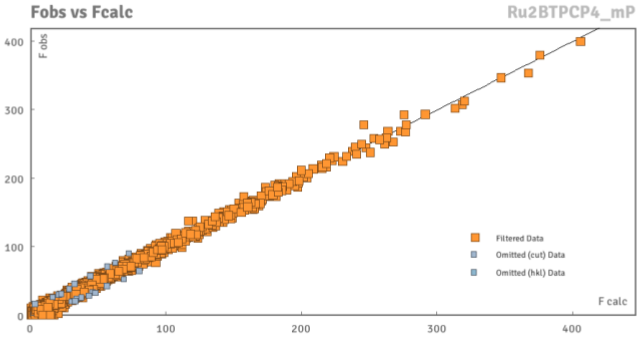

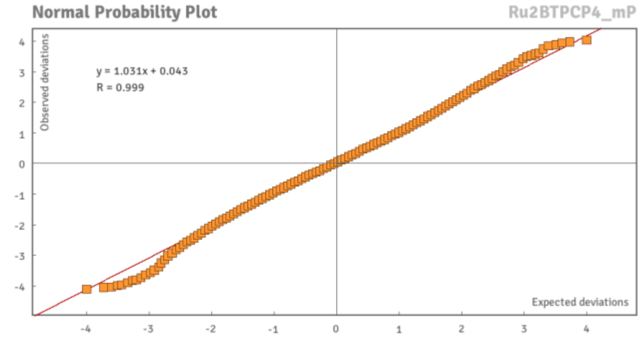

Reflection Statistics

Total reflections (after filtering)	89655	Unique reflections	15668
Completeness	0.937	Mean I/σ	11.61
hkl_{max} collected	(21, 12, 29)	hkl_{min} collected	(-21, -12, -30)
hkl_{max} used	(21, 12, 30)	hkl_{min} used	(-21, -12, 0)
Lim d_{max} collected	100.0	Lim d_{min} collected	0.77
d_{max} used	15.03	d_{min} used	0.85
Friedel pairs	9628	Friedel pairs merged	0
Inconsistent equivalents	3	R_{int}	0.0835
R_{sigma}	0.0601	Intensity transformed	0
Omitted reflections	167	Omitted by user (OMIT hkl)	0
Multiplicity	(5329, 5388, 4426, 2904, 1738, 1346, 1128, 854, 686, 413, 287, 174, 69, 31, 20, 5, 3, 1)	Maximum multiplicity	22
Removed systematic absences	0	Filtered off (Shel/OMIT)	0

Table 1: Fractional Atomic Coordinates (×10^4) and Equivalent Isotropic Displacement Parameters (Å^2×10^3) for Ru2BTPCP4_mP. U_{eq} is defined as 1/3 of the trace of the orthogonalised U_{ij}.

Atom	x	y	z	U_{eq}
Ru1	10000	7150.8	5000	32.2(2)
Ru2	10000	4931.5	5000	35.3(3)
Ru3	5000	4499.1(10)	10000	39.0(3)
Ru4	5000	2280.4(10)	10000	42.0(3)
Cl2	5000	6930(3)	10000	47.7(9)
Cl1	10000	9583(3)	5000	45.4(8)
O1W	10000	2685(10)	5000	78(4)
O2W	5000	33(12)	10000	81(4)
Cl1S_1	9047(3)	10683(5)	6434.2(17)	114.0(15)
Cl2S_1	10072(2)	12741(3)	6385(2)	105.5(15)
Cl3S_1	10591(3)	10179(4)	6580.2(17)	104.9(14)
C1S_1	9932(2)	11092(4)	6260(4)	79(4)
Cl1S_2	5427(3)	7488(4)	8437.6(16)	105.4(14)
Cl2S_2	6748(3)	7983(5)	9008.0(16)	129(2)
Cl3S_2	5835(3)	10071(4)	8707(2)	116.2(16)
C1S_2	5848(4)	8448(4)	8904(3)	96(5)
Cl1S_3	4538(8)	9727(18)	7381(5)	153(4)
Cl2S_3	5920(7)	9314(17)	6898(5)	169(4)
Cl3S_3	4744(9)	7569(14)	6722(6)	185(6)
C1S_3	4980(6)	9191(16)	6829(7)	104(6)
Cl1S_4	5671(7)	8059(17)	6837(6)	175(5)
Cl2S_4	4304(8)	8450(30)	7354(5)	216(8)
Cl3S_4	5162(7)	10519(15)	7144(5)	162(4)
C1S_4	4896(8)	9029(12)	6884(7)	104(6)
C1_5	6152(16)	2500(50)	5854(9)	122(5)
C2_5	5955(13)	2490(50)	5280(9)	122(5)
C3_5	5137(13)	2660(50)	5185(7)	122(5)
C4_5	4887(13)	2510(50)	4627(8)	122(5)
C5_5	4082(13)	2790(40)	4524(9)	122(5)
C6_5	3875(16)	2640(50)	3955(9)	122(5)
Br2_6	5671.0(9)	8983(2)	5472.3(9)	114.3(7)
O1_6	9261(3)	7104(5)	5585(2)	37.6(14)
O2_6	9262(4)	4929(6)	5585(3)	45.1(15)
C1_6	9025(4)	6014(9)	5740(3)	39.6(17)
C2_6	8419(4)	6008(9)	6124(3)	45.9(19)
C3_6	8602(5)	5987(10)	6713(3)	51.8(19)
C4_6	8326(6)	4779(9)	6449(4)	56(3)
C5_6	7753(5)	6728(9)	5959(4)	53(2)
C6_6	7112(5)	6083(11)	5863(4)	57(2)
C7_6	6501(6)	6756(11)	5709(5)	70(3)
C8_6	6516(6)	8070(12)	5656(5)	72(3)
C9_6	7154(5)	8779(10)	5766(5)	67(3)
C10_6	7774(5)	8092(8)	5894(4)	48(2)
C11_6	9376(5)	5977(10)	6896(3)	52(2)
C12_6	9566(6)	5051(12)	7272(5)	75(3)
C13_6	10244(7)	5108(13)	7511(5)	81(4)
C14_6	10747(6)	6021(12)	7366(4)	70(3)
C15_6	10579(6)	6899(11)	6984(4)	58(3)
C16_6	9890(5)	6880(10)	6751(3)	51(2)
C17_6	8109(6)	6776(9)	7047(4)	60(3)
C18_6	7461(6)	6310(12)	7251(4)	73(3)
C19_6	7022(7)	7065(13)	7564(5)	87(4)
C20_6	7196(7)	8345(14)	7642(5)	91(4)
C21_6	7844(8)	8821(12)	7453(5)	84(4)
C22_6	8285(7)	8078(10)	7159(5)	70(3)

Atom	x	y	z	U_{eq}
Br2_7	6401.9(7)	11013.2(14)	3862.9(5)	66.0(3)
O1_7	9124(3)	7089(5)	4508(2)	36.9(14)
O2_7	9197(3)	4927(5)	4477(2)	40.0(15)
C1_7	8883(4)	5991(8)	4371(3)	35.1(16)
C2_7	8199(4)	5945(8)	4056(3)	38.0(16)
C3_7	7728(4)	4676(7)	4071(3)	40.4(18)
C4_7	8208(5)	4935(8)	3623(3)	43(2)
C5_7	7812(4)	7216(7)	3992(3)	37.5(18)
C6_7	7597(5)	7705(8)	3518(3)	46(2)
C7_7	7182(6)	8838(9)	3473(4)	55(3)
C8_7	6963(6)	9446(9)	3913(4)	54(2)
C9_7	7184(5)	9013(8)	4398(4)	46(2)
C10_7	7599(5)	7914(8)	4436(3)	43(2)
C11_7	7923(5)	3558(8)	4429(3)	45(2)
C12_7	8195(6)	2397(8)	4225(4)	51(2)
C13_7	8386(5)	1365(8)	4553(4)	52(2)
C14_7	8289(6)	1475(9)	5077(4)	57(2)
C15_7	8039(6)	2638(9)	5283(4)	55(2)
C16_7	7833(5)	3666(8)	4956(3)	47(2)
C17_7	6923(4)	4840(8)	3999(4)	44.4(19)
C18_7	6585(5)	4341(11)	3557(4)	55(2)
C19_7	5839(6)	4520(17)	3478(4)	89(5)
C20_7	5435(6)	5140(20)	3834(6)	115(6)
C21_7	5753(6)	5657(19)	4276(5)	103(6)
C22_7	6483(5)	5446(10)	4364(4)	58(3)
Br2_8	9094.3(15)	6668(3)	10900.4(15)	197.3(17)
O1_8	6019(4)	4447(6)	9705(2)	49.5(17)
O2_8	6021(4)	2266(6)	9704(2)	50.3(16)
C1_8	6333(5)	3352(9)	9637(3)	47.1(18)
C2_8	7114(5)	3362(9)	9488(3)	53(2)
C3_8	7350(5)	3318(10)	8911(3)	60(2)
C4_8	7416(6)	2144(9)	9237(4)	64(3)
C5_8	7597(5)	4143(9)	9843(4)	56(2)
C6_8	8125(6)	3568(11)	10150(4)	73(3)
C7_8	8576(8)	4314(13)	10455(6)	105(5)
C8_8	8518(8)	5618(13)	10458(7)	111(6)
C9_8	7988(7)	6250(11)	10153(6)	91(4)
C10_8	7537(6)	5509(9)	9860(4)	58(3)
C11_8	6795(6)	3285(11)	8486(4)	64(3)
C12_8	6924(7)	2421(14)	8074(5)	83(4)
C13_8	6470(8)	2408(16)	7647(5)	101(5)
C14_8	5894(8)	3258(15)	7624(5)	90(4)
C15_8	5758(7)	4130(12)	8021(4)	70(3)
C16_8	6215(5)	4138(10)	8448(4)	56(2)
C17_8	7998(5)	4131(10)	8769(4)	68(3)
C18_8	8712(6)	3754(13)	8888(6)	95(5)
C19_8	9286(7)	4596(14)	8790(7)	97(5)
C20_8	9167(7)	5774(14)	8583(6)	91(4)
C21_8	8475(6)	6171(13)	8452(5)	79(3)
C22_8	7906(6)	5342(11)	8539(5)	74(3)
Br2_9	7595.2(9)	8350.3(14)	12088.3(5)	79.1(4)
O1_9	5478(4)	4445(6)	10703(2)	43.0(15)
O2_9	5414(4)	2274(6)	10714(3)	51.1(17)
C1_9	5615(5)	3345(8)	10910(3)	47(2)
C2_9	6040(5)	3301(8)	11400(3)	50(2)
C3_9	6452(5)	2005(8)	11539(3)	49(2)
C4_9	5752(5)	2307(9)	11784(3)	52(2)
C5_9	6365(6)	4568(8)	11571(3)	55(3)

Atom	x	y	z	U_{eq}
C6_9	6311(7)	5021(10)	12073(4)	63(3)
C7_9	6665(7)	6157(10)	12227(4)	70(3)
C8_9	7097(7)	6804(9)	11898(4)	63(3)
C9_9	7189(7)	6358(9)	11388(4)	69(3)
C10_9	6830(7)	5253(9)	11241(4)	62(3)
C11_9	6509(5)	897(8)	11157(3)	50(2)
C12_9	6144(6)	-272(8)	11258(4)	55(3)
C13_9	6172(6)	-1303(9)	10907(4)	58(3)
C14_9	6587(6)	-1169(9)	10463(4)	61(3)
C15_9	6959(6)	-29(9)	10364(4)	58(3)
C16_9	6912(6)	995(10)	10718(4)	56(2)
C17_9	7138(5)	2163(9)	11854(4)	56(2)
C18_9	7146(6)	1742(9)	12367(4)	60(3)
C19_9	7786(6)	1821(11)	12660(4)	68(3)
C20_9	8390(7)	2352(14)	12452(5)	88(4)
C21_9	8392(8)	2821(18)	11954(5)	114(7)
C22_9	7768(6)	2684(12)	11656(4)	78(4)

Table 2: Anisotropic Displacement Parameters (×10[4]) for Ru2BTPCP4_mP. The anisotropic displacement factor exponent takes the form: $-2\pi^2[h^2a^{*2} \times U_{11} + ... + 2hka^* \times b^* \times U_{12}]$

Atom	U_{11}	U_{22}	U_{33}	U_{23}	U_{13}	U_{12}
Ru1	39.7(6)	20.1(5)	36.6(6)	0	-7.8(4)	0
Ru2	41.6(6)	20.6(5)	43.6(6)	0	-11.1(5)	0
Ru3	59.3(7)	24.3(5)	33.5(6)	0	-18.0(5)	0
Ru4	65.8(7)	24.5(5)	35.7(6)	0	-20.4(5)	0
Cl2	71(2)	25.8(17)	46.7(19)	0	-16.0(17)	0
Cl1	57(2)	23.3(15)	56(2)	0	-16.9(17)	0
O1W	75(9)	23(6)	135(13)	0	-19(8)	0
O2W	104(11)	31(6)	107(11)	0	-58(9)	0
Cl1S_1	140(4)	99(3)	103(3)	23(2)	-30(3)	-56(3)
Cl2S_1	83(2)	47.5(18)	186(5)	32(2)	-1(3)	1.4(16)
Cl3S_1	155(4)	49.3(18)	110(3)	22.2(18)	-49(3)	-2(2)
C1S_1	121(10)	56(7)	60(7)	16(6)	-12(7)	-3(7)
Cl1S_2	147(4)	69(2)	101(3)	-22(2)	39(3)	1(2)
Cl2S_2	168(4)	147(5)	73(2)	29(2)	9(3)	73(4)
Cl3S_2	117(3)	61(2)	171(5)	-29(2)	-42(3)	14(2)
C1S_2	117(12)	108(11)	63(8)	9(9)	23(8)	28(9)
Cl1S_3	144(8)	213(11)	102(6)	-54(7)	-25(5)	47(7)
Cl2S_3	129(7)	218(13)	161(8)	-79(9)	-29(6)	18(7)
Cl3S_3	215(14)	153(11)	189(13)	-43(9)	87(11)	-38(10)
C1S_3	110(12)	123(14)	80(11)	-28(10)	-5(9)	24(10)
Cl1S_4	137(7)	221(13)	167(8)	-76(9)	-25(6)	25(7)
Cl2S_4	160(11)	370(30)	118(9)	17(15)	7(8)	-41(15)
Cl3S_4	155(8)	213(11)	118(6)	-53(7)	-31(6)	35(7)
C1S_4	110(12)	123(14)	80(11)	-28(10)	-5(9)	24(10)
C1_5	122(5)	123(5)	123(5)	-0.2(13)	0.5(13)	0.2(13)
C2_5	122(5)	123(5)	123(5)	-0.2(13)	0.5(13)	0.2(13)
C3_5	122(5)	123(5)	123(5)	-0.2(13)	0.5(13)	0.2(13)
C4_5	122(5)	123(5)	123(5)	-0.2(13)	0.5(13)	0.2(13)
C5_5	122(5)	123(5)	123(5)	-0.2(13)	0.5(13)	0.2(13)
C6_5	122(5)	123(5)	123(5)	-0.2(13)	0.5(13)	0.2(13)
Br2_6	64.0(9)	141.2(17)	137.7(16)	48.1(13)	-10.3(10)	23.6(10)
O1_6	50(4)	26(3)	36(3)	3(2)	-5(3)	-4(2)
O2_6	49(4)	31(3)	55(4)	2(3)	-13(3)	-1(3)
C1_6	47(4)	28(3)	43(4)	6(3)	-8(3)	-3(3)

Atom	U_{11}	U_{22}	U_{33}	U_{23}	U_{13}	U_{12}
C2_6	53(4)	38(4)	47(4)	9(4)	1(3)	-2(4)
C3_6	69(4)	44(4)	43(4)	13(4)	6(3)	2(4)
C4_6	69(7)	38(5)	62(6)	12(4)	10(5)	-5(4)
C5_6	53(4)	42(4)	64(6)	5(4)	-3(4)	1(3)
C6_6	54(5)	66(6)	51(6)	-4(5)	3(4)	-10(4)
C7_6	53(6)	88(6)	69(7)	0(6)	-7(5)	-10(5)
C8_6	55(6)	90(6)	72(8)	12(6)	2(6)	1(5)
C9_6	60(5)	58(6)	81(8)	20(6)	-2(6)	8(4)
C10_6	52(5)	40(4)	51(6)	4(4)	3(5)	0(4)
C11_6	68(4)	45(5)	43(5)	10(4)	2(4)	9(4)
C12_6	88(7)	57(7)	81(8)	33(6)	-4(6)	17(6)
C13_6	99(7)	91(9)	53(7)	25(7)	-5(6)	20(6)
C14_6	92(7)	64(7)	54(6)	0(5)	-15(5)	21(6)
C15_6	70(6)	63(7)	39(5)	-3(4)	-6(4)	9(5)
C16_6	66(5)	48(5)	38(5)	1(4)	-3(4)	3(4)
C17_6	73(6)	50(5)	58(6)	17(4)	13(5)	12(4)
C18_6	71(6)	88(8)	59(7)	19(6)	14(5)	8(5)
C19_6	87(9)	108(8)	68(8)	29(7)	21(7)	29(7)
C20_6	108(9)	107(8)	58(7)	19(8)	28(7)	35(8)
C21_6	115(9)	74(8)	63(8)	-3(6)	23(7)	21(6)
C22_6	88(8)	56(5)	65(7)	3(5)	22(6)	8(5)
Br2_7	75.5(8)	36.0(5)	86.4(8)	-0.2(6)	-28.5(6)	15.9(6)
O1_7	47(4)	24(3)	39(3)	-3(2)	-8(3)	-3(2)
O2_7	48(4)	24(3)	48(4)	0(3)	-8(3)	0(2)
C1_7	47(4)	23(3)	35(4)	-2(3)	-5(3)	0(3)
C2_7	49(4)	30(3)	35(4)	-1(3)	-10(3)	-1(3)
C3_7	49(4)	28(4)	45(5)	-9(3)	-18(3)	-3(3)
C4_7	57(6)	26(4)	45(4)	-6(3)	-14(4)	2(4)
C5_7	39(5)	30(4)	44(4)	-5(3)	-21(4)	-3(3)
C6_7	61(6)	31(4)	46(4)	-1(4)	-23(4)	0(4)
C7_7	75(7)	36(5)	53(5)	3(4)	-22(5)	6(4)
C8_7	60(6)	44(6)	58(5)	0(4)	-19(4)	7(5)
C9_7	52(6)	33(4)	54(5)	1(4)	-10(4)	0(4)
C10_7	55(6)	34(4)	39(4)	1(3)	-9(4)	2(3)
C11_7	47(5)	30(4)	57(4)	0(3)	-17(4)	0(4)
C12_7	62(6)	31(4)	58(6)	0(4)	-24(5)	5(4)
C13_7	55(6)	24(5)	77(5)	7(4)	-28(5)	-3(4)
C14_7	59(6)	37(4)	74(5)	21(4)	-20(5)	-5(4)
C15_7	65(7)	46(5)	54(6)	14(4)	-18(5)	-7(4)
C16_7	52(6)	34(5)	57(4)	8(4)	-9(4)	-1(4)
C17_7	49(4)	28(4)	57(5)	7(4)	-21(3)	-5(3)
C18_7	54(5)	58(6)	54(5)	3(5)	-28(4)	1(5)
C19_7	51(5)	167(15)	49(6)	8(7)	-29(5)	7(7)
C20_7	44(7)	214(19)	85(8)	-9(10)	-18(6)	22(9)
C21_7	49(5)	181(18)	77(8)	-19(9)	2(5)	24(7)
C22_7	51(5)	55(6)	69(6)	3(5)	-12(5)	2(4)
Br2_8	155(2)	129.3(19)	307(4)	-118(2)	-151(3)	39.9(16)
O1_8	78(5)	32(3)	38(4)	-4(3)	-20(3)	1(3)
O2_8	78(5)	33(3)	40(4)	-7(3)	-23(3)	-1(3)
C1_8	70(5)	31(3)	40(5)	-3(4)	-22(4)	1(3)
C2_8	70(5)	38(4)	50(4)	-3(4)	-8(4)	11(4)
C3_8	77(6)	51(5)	53(4)	-9(4)	-5(4)	6(5)
C4_8	82(8)	41(5)	69(7)	-11(4)	-3(6)	16(5)
C5_8	72(6)	43(4)	53(6)	-5(4)	-10(4)	8(4)
C6_8	91(8)	60(7)	68(7)	-4(5)	-26(6)	16(6)
C7_8	106(11)	86(7)	124(13)	-33(8)	-52(9)	25(7)
C8_8	86(10)	88(7)	159(14)	-40(8)	-60(9)	19(7)
C9_8	78(8)	60(8)	135(11)	-42(7)	-40(7)	17(6)

Atom	U_{11}	U_{22}	U_{33}	U_{23}	U_{13}	U_{12}
C10_8	76(7)	42(4)	57(7)	-10(4)	-18(5)	10(4)
C11_8	78(6)	57(6)	58(5)	-12(5)	-10(4)	11(5)
C12_8	96(9)	77(8)	77(7)	-36(7)	-9(6)	4(7)
C13_8	111(10)	112(12)	80(8)	-29(8)	-17(7)	12(8)
C14_8	100(9)	97(9)	72(7)	-18(7)	-25(7)	-3(7)
C15_8	86(8)	63(7)	59(6)	3(5)	-18(5)	-8(6)
C16_8	65(6)	51(6)	52(5)	-1(5)	-5(4)	1(4)
C17_8	74(5)	62(6)	68(7)	-4(5)	-1(5)	8(4)
C18_8	75(6)	73(9)	136(14)	19(8)	-10(8)	7(5)
C19_8	74(8)	95(9)	122(13)	27(9)	-6(9)	3(6)
C20_8	78(6)	87(9)	110(11)	18(8)	6(7)	0(7)
C21_8	79(6)	80(8)	78(8)	12(7)	12(6)	5(5)
C22_8	77(7)	69(7)	76(8)	10(6)	1(7)	6(5)
Br2_9	144.0(13)	33.5(5)	59.7(7)	-3.9(5)	-51.1(7)	-9.8(7)
O1_9	64(4)	27(3)	38(3)	1(2)	-20(3)	3(3)
O2_9	81(5)	26(3)	47(4)	0(3)	-16(3)	-2(3)
C1_9	72(6)	29(3)	39(4)	1(3)	-21(4)	7(4)
C2_9	85(6)	25(4)	40(4)	2(3)	-24(4)	2(4)
C3_9	77(5)	27(4)	42(5)	1(3)	-30(4)	-4(4)
C4_9	88(6)	35(5)	33(5)	4(4)	-23(4)	3(4)
C5_9	101(8)	26(4)	38(4)	-5(3)	-33(4)	3(4)
C6_9	104(9)	46(5)	40(5)	-10(4)	-30(5)	-9(5)
C7_9	118(9)	44(5)	49(5)	-3(4)	-47(5)	-11(6)
C8_9	118(9)	32(5)	39(4)	-1(3)	-48(5)	1(5)
C9_9	129(10)	34(5)	42(5)	5(4)	-31(6)	-12(5)
C10_9	120(9)	31(5)	35(5)	-8(4)	-28(5)	-5(5)
C11_9	70(6)	31(4)	50(5)	-2(4)	-28(4)	0(4)
C12_9	81(7)	33(4)	50(5)	-4(4)	-27(5)	-5(4)
C13_9	79(7)	34(5)	62(6)	-8(4)	-29(5)	1(5)
C14_9	81(7)	34(5)	69(6)	-14(4)	-25(5)	10(4)
C15_9	74(7)	37(4)	63(6)	0(4)	-25(5)	18(4)
C16_9	70(6)	39(5)	60(5)	0(4)	-19(4)	-3(5)
C17_9	89(6)	29(5)	51(5)	1(4)	-44(4)	0(4)
C18_9	98(7)	25(5)	57(5)	7(4)	-44(5)	-1(5)
C19_9	105(7)	38(6)	62(7)	10(5)	-47(5)	-5(5)
C20_9	110(8)	69(8)	86(8)	28(7)	-58(7)	-25(7)
C21_9	117(10)	136(15)	90(8)	52(9)	-77(8)	-69(11)
C22_9	93(7)	73(8)	68(7)	9(6)	-50(5)	-20(6)

Table 3: Bond Lengths in Å for Ru2BTPCP4_mP.

Atom	Atom	Length/Å
Ru1	Ru2	2.27721(4)
Ru1	Cl1	2.496(3)
Ru1	O1_6[1]	2.042(6)
Ru1	O1_6	2.042(6)
Ru1	O1_7[1]	2.056(6)
Ru1	O1_7	2.056(6)
Ru2	O1W	2.305(11)
Ru2	O2_6[1]	2.042(7)
Ru2	O2_6	2.042(7)
Ru2	O2_7	2.005(6)
Ru2	O2_7[1]	2.006(6)
Ru3	Ru4	2.2766(13)
Ru3	Cl2	2.495(3)
Ru3	O1_8	2.026(7)

Atom	Atom	Length/Å
Ru3	O1_8[2]	2.026(7)
Ru3	O1_9[2]	2.028(6)
Ru3	O1_9	2.028(6)
Ru4	O2W	2.306(12)
Ru4	O2_8	2.030(7)
Ru4	O2_8[2]	2.030(7)
Ru4	O2_9[2]	2.006(7)
Ru4	O2_9	2.006(7)
O1W	H1WA	0.9379
O1W	H1WB	0.9381
O2W	H2WA	1.0053
O2W	H2WB	1.0057
Cl1S_1	C1S_1	1.742(4)
Cl2S_1	C1S_1	1.742(4)

Atom	Atom	Length/Å
Cl3S_1	C1S_1	1.743(4)
C1S_1	H1S_1	1.0000
Cl1S_2	C1S_2	1.743(4)
Cl2S_2	C1S_2	1.742(4)
Cl3S_2	C1S_2	1.743(4)
C1S_2	H1S_2	1.0000
Cl1S_3	C1S_3	1.742(4)
Cl2S_3	C1S_3	1.742(4)
Cl3S_3	C1S_3	1.742(4)
C1S_3	H1S_3	1.0000
Cl1S_4	C1S_4	1.742(4)
Cl2S_4	C1S_4	1.742(4)
Cl3S_4	C1S_4	1.742(4)
C1S_4	H1S_4	1.0000
C1_5	H1A_5	0.9800
C1_5	H1B_5	0.9800
C1_5	H1C_5	0.9800
C1_5	C2_5	1.535(2)
C2_5	H2A_5	0.9900
C2_5	H2B_5	0.9900
C2_5	C3_5	1.532(2)
C3_5	H3A_5	0.9900
C3_5	H3B_5	0.9900
C3_5	C4_5	1.5306(17)
C4_5	H4A_5	0.9900
C4_5	H4B_5	0.9900
C4_5	C5_5	1.5308(17)
C5_5	H5A_5	0.9900
C5_5	H5B_5	0.9900
C5_5	C6_5	1.535(2)
C6_5	H6A_5	0.9800
C6_5	H6B_5	0.9800
C6_5	H6C_5	0.9800
Br2_6	C8_6	1.875(10)
O1_6	C1_6	1.266(9)
O2_6	C1_6	1.263(10)
C1_6	C2_6	1.499(10)
C2_6	C3_6	1.568(10)
C2_6	C4_6	1.527(11)
C2_6	C5_6	1.493(11)
C3_6	C4_6	1.505(12)
C3_6	C11_6	1.500(11)
C3_6	C17_6	1.494(11)
C4_6	H4A_6	0.9900
C4_6	H4B_6	0.9900
C5_6	C6_6	1.372(12)
C5_6	C10_6	1.411(11)
C6_6	H6_6	0.9500
C6_6	C7_6	1.377(13)
C7_6	H7_6	0.9500
C7_6	C8_6	1.356(14)
C8_6	C9_6	1.409(13)
C9_6	H9_6	0.9500
C9_6	C10_6	1.380(12)
C10_6	H10_6	0.9500
C11_6	C12_6	1.408(12)
C11_6	C16_6	1.377(12)
C12_6	H12_6	0.9500

Atom	Atom	Length/Å
C12_6	C13_6	1.393(14)
C13_6	H13_6	0.9500
C13_6	C14_6	1.369(15)
C14_6	H14_6	0.9500
C14_6	C15_6	1.376(13)
C15_6	H15_6	0.9500
C15_6	C16_6	1.403(12)
C16_6	H16_6	0.9500
C17_6	C18_6	1.391(12)
C17_6	C22_6	1.405(13)
C18_6	H18_6	0.9500
C18_6	C19_6	1.384(14)
C19_6	H19_6	0.9500
C19_6	C20_6	1.367(16)
C20_6	H20_6	0.9500
C20_6	C21_6	1.379(15)
C21_6	H21_6	0.9500
C21_6	C22_6	1.351(13)
C22_6	H22_6	0.9500
Br2_7	C8_7	1.915(9)
O1_7	C1_7	1.261(9)
O2_7	C1_7	1.265(9)
C1_7	C2_7	1.500(10)
C2_7	C3_7	1.564(10)
C2_7	C4_7	1.532(10)
C2_7	C5_7	1.494(10)
C3_7	C4_7	1.487(11)
C3_7	C11_7	1.520(10)
C3_7	C17_7	1.500(11)
C4_7	H4A_7	0.9900
C4_7	H4B_7	0.9900
C5_7	C6_7	1.390(11)
C5_7	C10_7	1.415(11)
C6_7	H6_7	0.9500
C6_7	C7_7	1.396(11)
C7_7	H7_7	0.9500
C7_7	C8_7	1.365(12)
C8_7	C9_7	1.396(12)
C9_7	H9_7	0.9500
C9_7	C10_7	1.365(11)
C10_7	H10_7	0.9500
C11_7	C12_7	1.397(11)
C11_7	C16_7	1.388(11)
C12_7	H12_7	0.9500
C12_7	C13_7	1.405(11)
C13_7	H13_7	0.9500
C13_7	C14_7	1.379(13)
C14_7	H14_7	0.9500
C14_7	C15_7	1.386(13)
C15_7	H15_7	0.9500
C15_7	C16_7	1.406(11)
C16_7	H16_7	0.9500
C17_7	C18_7	1.403(12)
C17_7	C22_7	1.394(12)
C18_7	H18_7	0.9500
C18_7	C19_7	1.398(13)
C19_7	H19_7	0.9500
C19_7	C20_7	1.349(15)

Atom	Atom	Length/Å
C20_7	H20_7	0.9500
C20_7	C21_7	1.394(16)
C21_7	H21_7	0.9500
C21_7	C22_7	1.379(13)
C22_7	H22_7	0.9500
Br2_8	C8_8	1.899(11)
O1_8	C1_8	1.276(10)
O2_8	C1_8	1.265(10)
C1_8	C2_8	1.488(11)
C2_8	C3_8	1.563(11)
C2_8	C4_8	1.516(11)
C2_8	C5_8	1.511(11)
C3_8	C4_8	1.478(13)
C3_8	C11_8	1.503(11)
C3_8	C17_8	1.502(12)
C4_8	H4A_8	0.9900
C4_8	H4B_8	0.9900
C5_8	C6_8	1.387(12)
C5_8	C10_8	1.407(12)
C6_8	H6_8	0.9500
C6_8	C7_8	1.379(14)
C7_8	H7_8	0.9500
C7_8	C8_8	1.342(15)
C8_8	C9_8	1.413(15)
C9_8	H9_8	0.9500
C9_8	C10_8	1.357(12)
C10_8	H10_8	0.9500
C11_8	C12_8	1.412(13)
C11_8	C16_8	1.382(12)
C12_8	H12_8	0.9500
C12_8	C13_8	1.388(15)
C13_8	H13_8	0.9500
C13_8	C14_8	1.374(16)
C14_8	H14_8	0.9500
C14_8	C15_8	1.390(14)
C15_8	H15_8	0.9500
C15_8	C16_8	1.392(13)
C16_8	H16_8	0.9500
C17_8	C18_8	1.402(13)
C17_8	C22_8	1.390(13)
C18_8	H18_8	0.9500
C18_8	C19_8	1.388(15)
C19_8	H19_8	0.9500
C19_8	C20_8	1.340(15)
C20_8	H20_8	0.9500
C20_8	C21_8	1.378(15)
C21_8	H21_8	0.9500
C21_8	C22_8	1.368(14)

Atom	Atom	Length/Å
C22_8	H22_8	0.9500
Br2_9	C8_9	1.897(9)
O1_9	C1_9	1.276(10)
O2_9	C1_9	1.267(10)
C1_9	C2_9	1.495(10)
C2_9	C3_9	1.572(10)
C2_9	C4_9	1.524(11)
C2_9	C5_9	1.498(11)
C3_9	C4_9	1.471(12)
C3_9	C11_9	1.515(10)
C3_9	C17_9	1.511(11)
C4_9	H4A_9	0.9900
C4_9	H4B_9	0.9900
C5_9	C6_9	1.391(12)
C5_9	C10_9	1.402(13)
C6_9	H6_9	0.9500
C6_9	C7_9	1.393(12)
C7_9	H7_9	0.9500
C7_9	C8_9	1.345(14)
C8_9	C9_9	1.414(12)
C9_9	H9_9	0.9500
C9_9	C10_9	1.366(12)
C10_9	H10_9	0.9500
C11_9	C12_9	1.399(12)
C11_9	C16_9	1.366(12)
C12_9	H12_9	0.9500
C12_9	C13_9	1.398(12)
C13_9	H13_9	0.9500
C13_9	C14_9	1.394(13)
C14_9	H14_9	0.9500
C14_9	C15_9	1.379(13)
C15_9	H15_9	0.9500
C15_9	C16_9	1.400(12)
C16_9	H16_9	0.9500
C17_9	C18_9	1.402(12)
C17_9	C22_9	1.377(13)
C18_9	H18_9	0.9500
C18_9	C19_9	1.402(13)
C19_9	H19_9	0.9500
C19_9	C20_9	1.352(15)
C20_9	H20_9	0.9500
C20_9	C21_9	1.380(15)
C21_9	H21_9	0.9500
C21_9	C22_9	1.391(13)
C22_9	H22_9	0.9500

----

^{1}2-x,+y,1-z; ^{2}1-x,+y,2-z

Table 4: Bond Angles in ° for Ru2BTPCP4_mP.

Atom	Atom	Atom	Angle/°
Ru2	Ru1	Cl1	180.0
O1_6	Ru1	Ru2	88.64(15)
O1_6^{1}	Ru1	Ru2	88.64(15)
O1_6	Ru1	Cl1	91.36(15)
O1_6^{1}	Ru1	Cl1	91.36(15)
O1_6^{1}	Ru1	O1_6	177.3(3)
O1_6	Ru1	O1_7^{1}	93.2(2)
O1_6^{1}	Ru1	O1_7	93.2(2)
O1_6	Ru1	O1_7	86.7(2)
O1_6^{1}	Ru1	O1_7^{1}	86.7(2)

Atom	Atom	Atom	Angle/°
O1_7	Ru1	Ru2	88.23(15)
O1_7[1]	Ru1	Ru2	88.23(15)
O1_7[1]	Ru1	Cl1	91.77(15)
O1_7	Ru1	Cl1	91.77(15)
O1_7[1]	Ru1	O1_7	176.5(3)
Ru1	Ru2	O1W	180.0
O2_6[1]	Ru2	Ru1	90.08(17)
O2_6	Ru2	Ru1	90.08(17)
O2_6	Ru2	O1W	89.92(17)
O2_6[1]	Ru2	O1W	89.92(17)
O2_6	Ru2	O2_6[1]	179.8(3)
O2_7	Ru2	Ru1	90.15(16)
O2_7[1]	Ru2	Ru1	90.14(16)
O2_7	Ru2	O1W	89.85(16)
O2_7[1]	Ru2	O1W	89.86(16)
O2_7[1]	Ru2	O2_6	89.0(3)
O2_7[1]	Ru2	O2_6[1]	91.0(3)
O2_7	Ru2	O2_6[1]	89.0(3)
O2_7	Ru2	O2_6	91.0(3)
O2_7	Ru2	O2_7[1]	179.7(3)
Ru4	Ru3	Cl2	180.0
O1_8[2]	Ru3	Ru4	88.49(18)
O1_8	Ru3	Ru4	88.49(18)
O1_8[2]	Ru3	Cl2	91.51(18)
O1_8	Ru3	Cl2	91.51(18)
O1_8	Ru3	O1_8[2]	177.0(4)
O1_8	Ru3	O1_9	86.7(3)
O1_8	Ru3	O1_9[2]	93.2(3)
O1_8[2]	Ru3	O1_9[2]	86.7(3)
O1_8[2]	Ru3	O1_9	93.2(3)
O1_9	Ru3	Ru4	88.43(16)
O1_9[2]	Ru3	Ru4	88.43(16)
O1_9	Ru3	Cl2	91.57(16)
O1_9[2]	Ru3	Cl2	91.57(16)
O1_9	Ru3	O1_9[2]	176.9(3)
Ru3	Ru4	O2W	180.0
O2_8	Ru4	Ru3	90.41(18)
O2_8[2]	Ru4	Ru3	90.41(18)
O2_8[2]	Ru4	O2W	89.59(18)
O2_8	Ru4	O2W	89.59(18)
O2_8[2]	Ru4	O2_8	179.2(4)
O2_9[2]	Ru4	Ru3	90.19(18)
O2_9	Ru4	Ru3	90.19(18)
O2_9	Ru4	O2W	89.81(18)
O2_9[2]	Ru4	O2W	89.81(18)
O2_9[2]	Ru4	O2_8	89.8(3)
O2_9	Ru4	O2_8[2]	89.8(3)
O2_9[2]	Ru4	O2_8[2]	90.2(3)
O2_9	Ru4	O2_8	90.2(3)
O2_9	Ru4	O2_9[2]	179.6(4)
Ru2	O1W	H1WA	127.9
Ru2	O1W	H1WB	127.9
H1WA	O1W	H1WB	104.3
Ru4	O2W	H2WA	128.1
Ru4	O2W	H2WB	128.1
H2WA	O2W	H2WB	103.8
Cl1S_1	C1S_1	Cl2S_1	108.9(4)
Cl1S_1	C1S_1	Cl3S_1	113.2(4)

Atom	Atom	Atom	Angle/°
Cl1S_1	C1S_1	H1S_1	108.5
Cl2S_1	C1S_1	Cl3S_1	109.3(4)
Cl2S_1	C1S_1	H1S_1	108.5
Cl3S_1	C1S_1	H1S_1	108.5
Cl1S_2	C1S_2	H1S_2	109.0
Cl2S_2	C1S_2	Cl1S_2	112.0(5)
Cl2S_2	C1S_2	Cl3S_2	108.7(4)
Cl2S_2	C1S_2	H1S_2	109.0
Cl3S_2	C1S_2	Cl1S_2	109.2(4)
Cl3S_2	C1S_2	H1S_2	109.0
Cl1S_3	C1S_3	Cl2S_3	110.9(11)
Cl1S_3	C1S_3	Cl3S_3	108.5(11)
Cl1S_3	C1S_3	H1S_3	109.3
Cl2S_3	C1S_3	Cl3S_3	109.4(12)
Cl2S_3	C1S_3	H1S_3	109.3
Cl3S_3	C1S_3	H1S_3	109.3
Cl1S_4	C1S_4	Cl3S_4	107.5(11)
Cl1S_4	C1S_4	H1S_4	111.9
Cl2S_4	C1S_4	Cl1S_4	111.6(12)
Cl2S_4	C1S_4	Cl3S_4	101.5(13)
Cl2S_4	C1S_4	H1S_4	111.9
Cl3S_4	C1S_4	H1S_4	111.9
H1A_5	C1_5	H1B_5	109.5
H1A_5	C1_5	H1C_5	109.5
H1B_5	C1_5	H1C_5	109.5
C2_5	C1_5	H1A_5	109.5
C2_5	C1_5	H1B_5	109.5
C2_5	C1_5	H1C_5	109.5
C1_5	C2_5	H2A_5	109.1
C1_5	C2_5	H2B_5	109.1
H2A_5	C2_5	H2B_5	107.8
C3_5	C2_5	C1_5	112.7(12)
C3_5	C2_5	H2A_5	109.1
C3_5	C2_5	H2B_5	109.1
C2_5	C3_5	H3A_5	108.3
C2_5	C3_5	H3B_5	108.3
H3A_5	C3_5	H3B_5	107.4
C4_5	C3_5	C2_5	115.7(14)
C4_5	C3_5	H3A_5	108.3
C4_5	C3_5	H3B_5	108.3
C3_5	C4_5	H4A_5	108.3
C3_5	C4_5	H4B_5	108.3
C3_5	C4_5	C5_5	115.8(14)
H4A_5	C4_5	H4B_5	107.4
C5_5	C4_5	H4A_5	108.3
C5_5	C4_5	H4B_5	108.3
C4_5	C5_5	H5A_5	109.0
C4_5	C5_5	H5B_5	109.0
C4_5	C5_5	C6_5	112.8(12)
H5A_5	C5_5	H5B_5	107.8
C6_5	C5_5	H5A_5	109.0
C6_5	C5_5	H5B_5	109.0
C5_5	C6_5	H6A_5	109.5
C5_5	C6_5	H6B_5	109.5
C5_5	C6_5	H6C_5	109.5
H6A_5	C6_5	H6B_5	109.5
H6A_5	C6_5	H6C_5	109.5
H6B_5	C6_5	H6C_5	109.5

Atom	Atom	Atom	Angle/˚
C1_6	O1_6	Ru1	119.2(5)
C1_6	O2_6	Ru2	117.9(5)
O1_6	C1_6	C2_6	118.2(8)
O2_6	C1_6	O1_6	123.9(7)
O2_6	C1_6	C2_6	117.9(7)
C1_6	C2_6	C3_6	119.6(7)
C1_6	C2_6	C4_6	117.1(7)
C4_6	C2_6	C3_6	58.2(6)
C5_6	C2_6	C1_6	114.6(7)
C5_6	C2_6	C3_6	117.6(7)
C5_6	C2_6	C4_6	118.4(7)
C4_6	C3_6	C2_6	59.6(5)
C11_6	C3_6	C2_6	120.8(7)
C11_6	C3_6	C4_6	117.3(8)
C17_6	C3_6	C2_6	115.6(8)
C17_6	C3_6	C4_6	120.5(8)
C17_6	C3_6	C11_6	113.2(8)
C2_6	C4_6	H4A_6	117.5
C2_6	C4_6	H4B_6	117.5
C3_6	C4_6	C2_6	62.2(5)
C3_6	C4_6	H4A_6	117.5
C3_6	C4_6	H4B_6	117.5
H4A_6	C4_6	H4B_6	114.6
C6_6	C5_6	C2_6	121.1(8)
C6_6	C5_6	C10_6	118.7(8)
C10_6	C5_6	C2_6	120.1(8)
C5_6	C6_6	H6_6	119.7
C5_6	C6_6	C7_6	120.7(10)
C7_6	C6_6	H6_6	119.7
C6_6	C7_6	H7_6	119.6
C8_6	C7_6	C6_6	120.7(10)
C8_6	C7_6	H7_6	119.6
C7_6	C8_6	Br2_6	120.4(8)
C7_6	C8_6	C9_6	120.6(10)
C9_6	C8_6	Br2_6	118.9(8)
C8_6	C9_6	H9_6	120.9
C10_6	C9_6	C8_6	118.2(9)
C10_6	C9_6	H9_6	120.9
C5_6	C10_6	H10_6	119.6
C9_6	C10_6	C5_6	120.8(9)
C9_6	C10_6	H10_6	119.6
C12_6	C11_6	C3_6	117.3(8)
C16_6	C11_6	C3_6	124.0(8)
C16_6	C11_6	C12_6	118.4(8)
C11_6	C12_6	H12_6	119.9
C13_6	C12_6	C11_6	120.2(10)
C13_6	C12_6	H12_6	119.9
C12_6	C13_6	H13_6	119.7
C14_6	C13_6	C12_6	120.6(10)
C14_6	C13_6	H13_6	119.7
C13_6	C14_6	H14_6	120.1
C13_6	C14_6	C15_6	119.8(10)
C15_6	C14_6	H14_6	120.1
C14_6	C15_6	H15_6	119.9
C14_6	C15_6	C16_6	120.3(10)
C16_6	C15_6	H15_6	119.9
C11_6	C16_6	C15_6	120.6(8)
C11_6	C16_6	H16_6	119.7

Atom	Atom	Atom	Angle/˚
C15_6	C16_6	H16_6	119.7
C18_6	C17_6	C3_6	123.8(9)
C18_6	C17_6	C22_6	116.4(9)
C22_6	C17_6	C3_6	119.8(9)
C17_6	C18_6	H18_6	118.9
C19_6	C18_6	C17_6	122.2(11)
C19_6	C18_6	H18_6	118.9
C18_6	C19_6	H19_6	120.3
C20_6	C19_6	C18_6	119.3(11)
C20_6	C19_6	H19_6	120.3
C19_6	C20_6	H20_6	120.4
C19_6	C20_6	C21_6	119.3(11)
C21_6	C20_6	H20_6	120.4
C20_6	C21_6	H21_6	119.3
C22_6	C21_6	C20_6	121.5(11)
C22_6	C21_6	H21_6	119.3
C17_6	C22_6	H22_6	119.5
C21_6	C22_6	C17_6	121.1(10)
C21_6	C22_6	H22_6	119.5
C1_7	O1_7	Ru1	118.5(5)
C1_7	O2_7	Ru2	118.7(5)
O1_7	C1_7	O2_7	123.3(7)
O1_7	C1_7	C2_7	118.4(7)
O2_7	C1_7	C2_7	118.2(7)
C1_7	C2_7	C3_7	118.4(6)
C1_7	C2_7	C4_7	114.3(7)
C4_7	C2_7	C3_7	57.4(5)
C5_7	C2_7	C1_7	115.5(6)
C5_7	C2_7	C3_7	117.8(6)
C5_7	C2_7	C4_7	121.0(7)
C4_7	C3_7	C2_7	60.2(5)
C4_7	C3_7	C11_7	118.4(7)
C4_7	C3_7	C17_7	118.0(7)
C11_7	C3_7	C2_7	120.9(7)
C17_7	C3_7	C2_7	116.6(7)
C17_7	C3_7	C11_7	113.1(7)
C2_7	C4_7	H4A_7	117.5
C2_7	C4_7	H4B_7	117.5
C3_7	C4_7	C2_7	62.4(5)
C3_7	C4_7	H4A_7	117.5
C3_7	C4_7	H4B_7	117.5
H4A_7	C4_7	H4B_7	114.6
C6_7	C5_7	C2_7	123.2(7)
C6_7	C5_7	C10_7	117.6(7)
C10_7	C5_7	C2_7	119.0(7)
C5_7	C6_7	H6_7	119.0
C5_7	C6_7	C7_7	121.9(8)
C7_7	C6_7	H6_7	119.0
C6_7	C7_7	H7_7	120.9
C8_7	C7_7	C6_7	118.3(9)
C8_7	C7_7	H7_7	120.9
C7_7	C8_7	Br2_7	119.1(7)
C7_7	C8_7	C9_7	121.7(8)
C9_7	C8_7	Br2_7	119.0(7)
C8_7	C9_7	H9_7	120.3
C10_7	C9_7	C8_7	119.4(9)
C10_7	C9_7	H9_7	120.3
C5_7	C10_7	H10_7	119.5

Atom	Atom	Atom	Angle/°
C9_7	C10_7	C5_7	121.0(8)
C9_7	C10_7	H10_7	119.5
C12_7	C11_7	C3_7	119.6(8)
C16_7	C11_7	C3_7	121.2(7)
C16_7	C11_7	C12_7	119.1(7)
C11_7	C12_7	H12_7	119.9
C11_7	C12_7	C13_7	120.1(9)
C13_7	C12_7	H12_7	119.9
C12_7	C13_7	H13_7	119.8
C14_7	C13_7	C12_7	120.4(8)
C14_7	C13_7	H13_7	119.8
C13_7	C14_7	H14_7	120.1
C13_7	C14_7	C15_7	119.7(8)
C15_7	C14_7	H14_7	120.1
C14_7	C15_7	H15_7	119.9
C14_7	C15_7	C16_7	120.1(9)
C16_7	C15_7	H15_7	119.9
C11_7	C16_7	C15_7	120.3(8)
C11_7	C16_7	H16_7	119.8
C15_7	C16_7	H16_7	119.8
C18_7	C17_7	C3_7	119.7(8)
C22_7	C17_7	C3_7	122.6(8)
C22_7	C17_7	C18_7	117.6(8)
C17_7	C18_7	H18_7	119.8
C19_7	C18_7	C17_7	120.4(9)
C19_7	C18_7	H18_7	119.8
C18_7	C19_7	H19_7	119.9
C20_7	C19_7	C18_7	120.1(10)
C20_7	C19_7	H19_7	119.9
C19_7	C20_7	H20_7	119.5
C19_7	C20_7	C21_7	121.1(10)
C21_7	C20_7	H20_7	119.5
C20_7	C21_7	H21_7	120.6
C22_7	C21_7	C20_7	118.9(11)
C22_7	C21_7	H21_7	120.6
C17_7	C22_7	H22_7	119.2
C21_7	C22_7	C17_7	121.6(10)
C21_7	C22_7	H22_7	119.2
C1_8	O1_8	Ru3	119.6(6)
C1_8	O2_8	Ru4	117.8(6)
O1_8	C1_8	C2_8	117.8(8)
O2_8	C1_8	O1_8	123.4(8)
O2_8	C1_8	C2_8	118.7(8)
C1_8	C2_8	C3_8	121.3(7)
C1_8	C2_8	C4_8	117.4(8)
C1_8	C2_8	C5_8	114.3(7)
C4_8	C2_8	C3_8	57.3(6)
C5_8	C2_8	C3_8	116.0(8)
C5_8	C2_8	C4_8	119.0(8)
C4_8	C3_8	C2_8	59.7(6)
C4_8	C3_8	C11_8	117.3(9)
C4_8	C3_8	C17_8	122.0(9)
C11_8	C3_8	C2_8	121.2(8)
C17_8	C3_8	C2_8	116.3(8)
C17_8	C3_8	C11_8	111.6(8)
C2_8	C4_8	H4A_8	117.5
C2_8	C4_8	H4B_8	117.5
C3_8	C4_8	C2_8	62.9(6)

Atom	Atom	Atom	Angle/°
C3_8	C4_8	H4A_8	117.5
C3_8	C4_8	H4B_8	117.5
H4A_8	C4_8	H4B_8	114.5
C6_8	C5_8	C2_8	122.5(9)
C6_8	C5_8	C10_8	117.4(9)
C10_8	C5_8	C2_8	120.1(8)
C5_8	C6_8	H6_8	119.5
C7_8	C6_8	C5_8	121.0(10)
C7_8	C6_8	H6_8	119.5
C6_8	C7_8	H7_8	119.7
C8_8	C7_8	C6_8	120.5(11)
C8_8	C7_8	H7_8	119.7
C7_8	C8_8	Br2_8	121.6(10)
C7_8	C8_8	C9_8	120.7(11)
C9_8	C8_8	Br2_8	117.6(9)
C8_8	C9_8	H9_8	120.7
C10_8	C9_8	C8_8	118.5(10)
C10_8	C9_8	H9_8	120.7
C5_8	C10_8	H10_8	119.1
C9_8	C10_8	C5_8	121.9(10)
C9_8	C10_8	H10_8	119.1
C12_8	C11_8	C3_8	117.3(9)
C16_8	C11_8	C3_8	124.1(9)
C16_8	C11_8	C12_8	118.3(9)
C11_8	C12_8	H12_8	119.6
C13_8	C12_8	C11_8	120.8(11)
C13_8	C12_8	H12_8	119.6
C12_8	C13_8	H13_8	120.3
C14_8	C13_8	C12_8	119.4(11)
C14_8	C13_8	H13_8	120.3
C13_8	C14_8	H14_8	119.5
C13_8	C14_8	C15_8	121.0(11)
C15_8	C14_8	H14_8	119.5
C14_8	C15_8	H15_8	120.4
C14_8	C15_8	C16_8	119.2(10)
C16_8	C15_8	H15_8	120.4
C11_8	C16_8	C15_8	121.2(9)
C11_8	C16_8	H16_8	119.4
C15_8	C16_8	H16_8	119.4
C18_8	C17_8	C3_8	122.3(9)
C22_8	C17_8	C3_8	120.5(9)
C22_8	C17_8	C18_8	117.0(9)
C17_8	C18_8	H18_8	120.0
C19_8	C18_8	C17_8	119.9(11)
C19_8	C18_8	H18_8	120.0
C18_8	C19_8	H19_8	119.6
C20_8	C19_8	C18_8	120.8(12)
C20_8	C19_8	H19_8	119.6
C19_8	C20_8	H20_8	119.5
C19_8	C20_8	C21_8	121.0(12)
C21_8	C20_8	H20_8	119.5
C20_8	C21_8	H21_8	120.6
C22_8	C21_8	C20_8	118.8(11)
C22_8	C21_8	H21_8	120.6
C17_8	C22_8	H22_8	118.8
C21_8	C22_8	C17_8	122.4(11)
C21_8	C22_8	H22_8	118.8
C1_9	O1_9	Ru3	119.4(5)

Atom	Atom	Atom	Angle/°
C1_9	O2_9	Ru4	118.7(5)
O1_9	C1_9	C2_9	119.3(7)
O2_9	C1_9	O1_9	122.7(7)
O2_9	C1_9	C2_9	118.0(7)
C1_9	C2_9	C3_9	118.2(7)
C1_9	C2_9	C4_9	113.5(8)
C1_9	C2_9	C5_9	115.7(7)
C4_9	C2_9	C3_9	56.7(5)
C5_9	C2_9	C3_9	118.3(7)
C5_9	C2_9	C4_9	121.7(7)
C4_9	C3_9	C2_9	60.0(6)
C4_9	C3_9	C11_9	120.3(8)
C4_9	C3_9	C17_9	118.1(8)
C11_9	C3_9	C2_9	121.2(7)
C17_9	C3_9	C2_9	115.8(7)
C17_9	C3_9	C11_9	112.2(7)
C2_9	C4_9	H4A_9	117.4
C2_9	C4_9	H4B_9	117.4
C3_9	C4_9	C2_9	63.3(6)
C3_9	C4_9	H4A_9	117.4
C3_9	C4_9	H4B_9	117.4
H4A_9	C4_9	H4B_9	114.5
C6_9	C5_9	C2_9	122.7(9)
C6_9	C5_9	C10_9	116.9(8)
C10_9	C5_9	C2_9	119.8(8)
C5_9	C6_9	H6_9	119.5
C5_9	C6_9	C7_9	121.1(10)
C7_9	C6_9	H6_9	119.5
C6_9	C7_9	H7_9	119.8
C8_9	C7_9	C6_9	120.4(9)
C8_9	C7_9	H7_9	119.8
C7_9	C8_9	Br2_9	122.1(7)
C7_9	C8_9	C9_9	120.7(9)
C9_9	C8_9	Br2_9	117.2(8)
C8_9	C9_9	H9_9	120.9
C10_9	C9_9	C8_9	118.2(9)
C10_9	C9_9	H9_9	120.9
C5_9	C10_9	H10_9	118.7
C9_9	C10_9	C5_9	122.6(9)

Atom	Atom	Atom	Angle/°
C9_9	C10_9	H10_9	118.7
C12_9	C11_9	C3_9	119.1(8)
C16_9	C11_9	C3_9	122.1(8)
C16_9	C11_9	C12_9	118.8(8)
C11_9	C12_9	H12_9	119.8
C13_9	C12_9	C11_9	120.4(9)
C13_9	C12_9	H12_9	119.8
C12_9	C13_9	H13_9	120.4
C14_9	C13_9	C12_9	119.3(9)
C14_9	C13_9	H13_9	120.4
C13_9	C14_9	H14_9	119.7
C15_9	C14_9	C13_9	120.7(9)
C15_9	C14_9	H14_9	119.7
C14_9	C15_9	H15_9	120.5
C14_9	C15_9	C16_9	118.9(10)
C16_9	C15_9	H15_9	120.5
C11_9	C16_9	C15_9	121.9(9)
C11_9	C16_9	H16_9	119.1
C15_9	C16_9	H16_9	119.1
C18_9	C17_9	C3_9	119.4(9)
C22_9	C17_9	C3_9	122.6(8)
C22_9	C17_9	C18_9	117.9(8)
C17_9	C18_9	H18_9	119.7
C17_9	C18_9	C19_9	120.5(10)
C19_9	C18_9	H18_9	119.7
C18_9	C19_9	H19_9	120.2
C20_9	C19_9	C18_9	119.6(10)
C20_9	C19_9	H19_9	120.2
C19_9	C20_9	H20_9	119.3
C19_9	C20_9	C21_9	121.4(10)
C21_9	C20_9	H20_9	119.3
C20_9	C21_9	H21_9	120.5
C20_9	C21_9	C22_9	119.0(11)
C22_9	C21_9	H21_9	120.5
C17_9	C22_9	C21_9	121.5(10)
C17_9	C22_9	H22_9	119.2
C21_9	C22_9	H22_9	119.2

––––
[1]2-x,+y,1-z; [2]1-x,+y,2-z

Table 5: Torsion Angles in ° for Ru2BTPCP4_mP.

Atom	Atom	Atom	Atom	Angle/°
Ru1	O1_6	C1_6	O2_6	6.1(11)
Ru1	O1_6	C1_6	C2_6	-172.7(5)
Ru1	O1_7	C1_7	O2_7	-7.6(10)
Ru1	O1_7	C1_7	C2_7	173.8(5)
Ru2	O2_6	C1_6	O1_6	-6.1(11)
Ru2	O2_6	C1_6	C2_6	172.7(5)
Ru2	O2_7	C1_7	O1_7	12.5(10)
Ru2	O2_7	C1_7	C2_7	-168.9(5)
Ru3	O1_8	C1_8	O2_8	6.1(11)
Ru3	O1_8	C1_8	C2_8	-171.7(5)
Ru3	O1_9	C1_9	O2_9	-5.7(13)
Ru3	O1_9	C1_9	C2_9	173.3(7)
Ru4	O2_8	C1_8	O1_8	-6.0(11)
Ru4	O2_8	C1_8	C2_8	171.8(6)

Atom	Atom	Atom	Atom	Angle/˚
Ru4	O2_9	C1_9	O1_9	9.6(13)
Ru4	O2_9	C1_9	C2_9	-169.4(7)
C1_5	C2_5	C3_5	C4_5	-173(5)
C2_5	C3_5	C4_5	C5_5	-175(3)
C3_5	C4_5	C5_5	C6_5	179(4)
Br2_6	C8_6	C9_6	C10_6	-179.2(9)
O1_6	C1_6	C2_6	C3_6	-91.4(10)
O1_6	C1_6	C2_6	C4_6	-158.4(8)
O1_6	C1_6	C2_6	C5_6	56.3(11)
O2_6	C1_6	C2_6	C3_6	89.7(10)
O2_6	C1_6	C2_6	C4_6	22.6(12)
O2_6	C1_6	C2_6	C5_6	-122.6(9)
C1_6	C2_6	C3_6	C4_6	-105.4(9)
C1_6	C2_6	C3_6	C11_6	0.2(13)
C1_6	C2_6	C3_6	C17_6	142.8(8)
C1_6	C2_6	C4_6	C3_6	109.6(8)
C1_6	C2_6	C5_6	C6_6	113.1(10)
C1_6	C2_6	C5_6	C10_6	-66.5(12)
C2_6	C3_6	C11_6	C12_6	-131.5(11)
C2_6	C3_6	C11_6	C16_6	54.0(14)
C2_6	C3_6	C17_6	C18_6	86.7(13)
C2_6	C3_6	C17_6	C22_6	-91.8(12)
C2_6	C5_6	C6_6	C7_6	-179.9(10)
C2_6	C5_6	C10_6	C9_6	-176.7(10)
C3_6	C2_6	C5_6	C6_6	-98.5(12)
C3_6	C2_6	C5_6	C10_6	81.9(11)
C3_6	C11_6	C12_6	C13_6	-170.9(11)
C3_6	C11_6	C16_6	C15_6	172.5(9)
C3_6	C17_6	C18_6	C19_6	179.2(11)
C3_6	C17_6	C22_6	C21_6	178.9(12)
C4_6	C2_6	C3_6	C11_6	105.6(10)
C4_6	C2_6	C3_6	C17_6	-111.8(9)
C4_6	C2_6	C5_6	C6_6	-31.7(14)
C4_6	C2_6	C5_6	C10_6	148.7(9)
C4_6	C3_6	C11_6	C12_6	-62.4(12)
C4_6	C3_6	C11_6	C16_6	123.1(10)
C4_6	C3_6	C17_6	C18_6	18.4(15)
C4_6	C3_6	C17_6	C22_6	-160.1(10)
C5_6	C2_6	C3_6	C4_6	107.9(9)
C5_6	C2_6	C3_6	C11_6	-146.5(9)
C5_6	C2_6	C3_6	C17_6	-4.0(12)
C5_6	C2_6	C4_6	C3_6	-106.5(9)
C5_6	C6_6	C7_6	C8_6	-0.9(18)
C6_6	C5_6	C10_6	C9_6	3.6(16)
C6_6	C7_6	C8_6	Br2_6	-177.5(9)
C6_6	C7_6	C8_6	C9_6	-1(2)
C7_6	C8_6	C9_6	C10_6	4.5(19)
C8_6	C9_6	C10_6	C5_6	-5.7(17)
C10_6	C5_6	C6_6	C7_6	-0.3(16)
C11_6	C3_6	C4_6	C2_6	-111.4(8)
C11_6	C3_6	C17_6	C18_6	-128.0(11)
C11_6	C3_6	C17_6	C22_6	53.6(13)
C11_6	C12_6	C13_6	C14_6	-3(2)
C12_6	C11_6	C16_6	C15_6	-2.0(16)
C12_6	C13_6	C14_6	C15_6	1(2)
C13_6	C14_6	C15_6	C16_6	1.4(18)
C14_6	C15_6	C16_6	C11_6	-0.7(16)
C16_6	C11_6	C12_6	C13_6	3.9(18)

Atom	Atom	Atom	Atom	Angle/°
C17_6	C3_6	C4_6	C2_6	103.6(9)
C17_6	C3_6	C11_6	C12_6	85.1(12)
C17_6	C3_6	C11_6	C16_6	-89.4(11)
C17_6	C18_6	C19_6	C20_6	6(2)
C18_6	C17_6	C22_6	C21_6	0.4(19)
C18_6	C19_6	C20_6	C21_6	-7(2)
C19_6	C20_6	C21_6	C22_6	5(2)
C20_6	C21_6	C22_6	C17_6	-2(2)
C22_6	C17_6	C18_6	C19_6	-2.3(18)
Br2_7	C8_7	C9_7	C10_7	-178.5(7)
O1_7	C1_7	C2_7	C3_7	-155.6(7)
O1_7	C1_7	C2_7	C4_7	139.7(7)
O1_7	C1_7	C2_7	C5_7	-7.7(11)
O2_7	C1_7	C2_7	C3_7	25.7(11)
O2_7	C1_7	C2_7	C4_7	-39.0(10)
O2_7	C1_7	C2_7	C5_7	173.6(7)
C1_7	C2_7	C3_7	C4_7	-102.1(8)
C1_7	C2_7	C3_7	C11_7	5.1(11)
C1_7	C2_7	C3_7	C17_7	149.3(7)
C1_7	C2_7	C4_7	C3_7	109.3(7)
C1_7	C2_7	C5_7	C6_7	128.9(9)
C1_7	C2_7	C5_7	C10_7	-56.4(10)
C2_7	C3_7	C11_7	C12_7	-109.5(10)
C2_7	C3_7	C11_7	C16_7	70.8(11)
C2_7	C3_7	C17_7	C18_7	113.2(9)
C2_7	C3_7	C17_7	C22_7	-68.5(11)
C2_7	C5_7	C6_7	C7_7	174.3(9)
C2_7	C5_7	C10_7	C9_7	-173.6(8)
C3_7	C2_7	C5_7	C6_7	-83.0(11)
C3_7	C2_7	C5_7	C10_7	91.7(9)
C3_7	C11_7	C12_7	C13_7	179.1(8)
C3_7	C11_7	C16_7	C15_7	-177.9(9)
C3_7	C17_7	C18_7	C19_7	-178.1(11)
C3_7	C17_7	C22_7	C21_7	176.0(12)
C4_7	C2_7	C3_7	C11_7	107.3(9)
C4_7	C2_7	C3_7	C17_7	-108.6(8)
C4_7	C2_7	C5_7	C6_7	-16.2(12)
C4_7	C2_7	C5_7	C10_7	158.5(8)
C4_7	C3_7	C11_7	C12_7	-39.1(12)
C4_7	C3_7	C11_7	C16_7	141.3(9)
C4_7	C3_7	C17_7	C18_7	44.5(11)
C4_7	C3_7	C17_7	C22_7	-137.3(9)
C5_7	C2_7	C3_7	C4_7	110.7(8)
C5_7	C2_7	C3_7	C11_7	-142.0(8)
C5_7	C2_7	C3_7	C17_7	2.2(10)
C5_7	C2_7	C4_7	C3_7	-105.2(8)
C5_7	C6_7	C7_7	C8_7	-2.3(16)
C6_7	C5_7	C10_7	C9_7	1.4(13)
C6_7	C7_7	C8_7	Br2_7	179.3(8)
C6_7	C7_7	C8_7	C9_7	4.3(17)
C7_7	C8_7	C9_7	C10_7	-3.4(16)
C8_7	C9_7	C10_7	C5_7	0.5(14)
C10_7	C5_7	C6_7	C7_7	-0.5(14)
C11_7	C3_7	C4_7	C2_7	-111.3(8)
C11_7	C3_7	C17_7	C18_7	-99.9(10)
C11_7	C3_7	C17_7	C22_7	78.4(10)
C11_7	C12_7	C13_7	C14_7	1.8(16)
C12_7	C11_7	C16_7	C15_7	2.5(15)

Atom	Atom	Atom	Atom	Angle/°
C12_7	C13_7	C14_7	C15_7	-3.6(16)
C13_7	C14_7	C15_7	C16_7	4.8(17)
C14_7	C15_7	C16_7	C11_7	-4.3(16)
C16_7	C11_7	C12_7	C13_7	-1.3(15)
C17_7	C3_7	C4_7	C2_7	106.3(8)
C17_7	C3_7	C11_7	C12_7	105.1(10)
C17_7	C3_7	C11_7	C16_7	-74.5(10)
C17_7	C18_7	C19_7	C20_7	-2(2)
C18_7	C17_7	C22_7	C21_7	-5.8(17)
C18_7	C19_7	C20_7	C21_7	3(3)
C19_7	C20_7	C21_7	C22_7	-5(3)
C20_7	C21_7	C22_7	C17_7	6(2)
C22_7	C17_7	C18_7	C19_7	3.6(16)
Br2_8	C8_8	C9_8	C10_8	-175.6(12)
O1_8	C1_8	C2_8	C3_8	-93.3(10)
O1_8	C1_8	C2_8	C4_8	-160.0(8)
O1_8	C1_8	C2_8	C5_8	53.6(11)
O2_8	C1_8	C2_8	C3_8	88.8(11)
O2_8	C1_8	C2_8	C4_8	22.1(12)
O2_8	C1_8	C2_8	C5_8	-124.3(9)
C1_8	C2_8	C3_8	C4_8	-104.5(10)
C1_8	C2_8	C3_8	C11_8	1.0(14)
C1_8	C2_8	C3_8	C17_8	142.1(9)
C1_8	C2_8	C4_8	C3_8	111.3(9)
C1_8	C2_8	C5_8	C6_8	113.0(11)
C1_8	C2_8	C5_8	C10_8	-69.0(12)
C2_8	C3_8	C11_8	C12_8	-137.2(12)
C2_8	C3_8	C11_8	C16_8	50.1(16)
C2_8	C3_8	C17_8	C18_8	76.3(14)
C2_8	C3_8	C17_8	C22_8	-99.6(12)
C2_8	C5_8	C6_8	C7_8	177.4(13)
C2_8	C5_8	C10_8	C9_8	-176.3(12)
C3_8	C2_8	C5_8	C6_8	-98.3(12)
C3_8	C2_8	C5_8	C10_8	79.7(12)
C3_8	C11_8	C12_8	C13_8	-174.7(14)
C3_8	C11_8	C16_8	C15_8	174.2(11)
C3_8	C17_8	C18_8	C19_8	-174.1(13)
C3_8	C17_8	C22_8	C21_8	173.0(12)
C4_8	C2_8	C3_8	C11_8	105.5(11)
C4_8	C2_8	C3_8	C17_8	-113.4(10)
C4_8	C2_8	C5_8	C6_8	-32.8(14)
C4_8	C2_8	C5_8	C10_8	145.1(10)
C4_8	C3_8	C11_8	C12_8	-67.7(14)
C4_8	C3_8	C11_8	C16_8	119.5(12)
C4_8	C3_8	C17_8	C18_8	7.1(16)
C4_8	C3_8	C17_8	C22_8	-168.9(11)
C5_8	C2_8	C3_8	C4_8	109.2(9)
C5_8	C2_8	C3_8	C11_8	-145.3(9)
C5_8	C2_8	C3_8	C17_8	-4.2(12)
C5_8	C2_8	C4_8	C3_8	-103.9(9)
C5_8	C6_8	C7_8	C8_8	-1(3)
C6_8	C5_8	C10_8	C9_8	1.7(18)
C6_8	C7_8	C8_8	Br2_8	176.6(13)
C6_8	C7_8	C8_8	C9_8	1(3)
C7_8	C8_8	C9_8	C10_8	0(3)
C8_8	C9_8	C10_8	C5_8	-2(2)
C10_8	C5_8	C6_8	C7_8	-0.6(19)
C11_8	C3_8	C4_8	C2_8	-111.9(9)

Atom	Atom	Atom	Atom	Angle/°
C11_8	C3_8	C17_8	C18_8	-139.0(12)
C11_8	C3_8	C17_8	C22_8	45.1(14)
C11_8	C12_8	C13_8	C14_8	1(3)
C12_8	C11_8	C16_8	C15_8	1.5(19)
C12_8	C13_8	C14_8	C15_8	0(3)
C13_8	C14_8	C15_8	C16_8	0(2)
C14_8	C15_8	C16_8	C11_8	-0.8(19)
C16_8	C11_8	C12_8	C13_8	-2(2)
C17_8	C3_8	C4_8	C2_8	103.9(10)
C17_8	C3_8	C11_8	C12_8	80.1(14)
C17_8	C3_8	C11_8	C16_8	-92.7(13)
C17_8	C18_8	C19_8	C20_8	0(3)
C18_8	C17_8	C22_8	C21_8	-3(2)
C18_8	C19_8	C20_8	C21_8	-2(3)
C19_8	C20_8	C21_8	C22_8	0(2)
C20_8	C21_8	C22_8	C17_8	2(2)
C22_8	C17_8	C18_8	C19_8	2(2)
Br2_9	C8_9	C9_9	C10_9	-179.5(9)
O1_9	C1_9	C2_9	C3_9	-157.2(9)
O1_9	C1_9	C2_9	C4_9	139.3(9)
O1_9	C1_9	C2_9	C5_9	-8.4(14)
O2_9	C1_9	C2_9	C3_9	21.9(14)
O2_9	C1_9	C2_9	C4_9	-41.7(12)
O2_9	C1_9	C2_9	C5_9	170.6(9)
C1_9	C2_9	C3_9	C4_9	-100.9(9)
C1_9	C2_9	C3_9	C11_9	8.4(13)
C1_9	C2_9	C3_9	C17_9	150.1(9)
C1_9	C2_9	C4_9	C3_9	109.3(8)
C1_9	C2_9	C5_9	C6_9	133.3(11)
C1_9	C2_9	C5_9	C10_9	-56.1(13)
C2_9	C3_9	C11_9	C12_9	-112.4(10)
C2_9	C3_9	C11_9	C16_9	68.4(13)
C2_9	C3_9	C17_9	C18_9	110.1(10)
C2_9	C3_9	C17_9	C22_9	-71.2(13)
C2_9	C5_9	C6_9	C7_9	175.2(10)
C2_9	C5_9	C10_9	C9_9	-174.7(10)
C3_9	C2_9	C5_9	C6_9	-78.0(13)
C3_9	C2_9	C5_9	C10_9	92.6(11)
C3_9	C11_9	C12_9	C13_9	178.6(9)
C3_9	C11_9	C16_9	C15_9	-179.6(9)
C3_9	C17_9	C18_9	C19_9	177.0(9)
C3_9	C17_9	C22_9	C21_9	179.9(13)
C4_9	C2_9	C3_9	C11_9	109.4(9)
C4_9	C2_9	C3_9	C17_9	-109.0(9)
C4_9	C2_9	C5_9	C6_9	-11.6(15)
C4_9	C2_9	C5_9	C10_9	159.0(10)
C4_9	C3_9	C11_9	C12_9	-41.3(12)
C4_9	C3_9	C11_9	C16_9	139.5(10)
C4_9	C3_9	C17_9	C18_9	41.9(12)
C4_9	C3_9	C17_9	C22_9	-139.4(11)
C5_9	C2_9	C3_9	C4_9	111.1(9)
C5_9	C2_9	C3_9	C11_9	-139.5(9)
C5_9	C2_9	C3_9	C17_9	2.1(12)
C5_9	C2_9	C4_9	C3_9	-105.2(9)
C5_9	C6_9	C7_9	C8_9	-3.3(19)
C6_9	C5_9	C10_9	C9_9	-3.5(17)
C6_9	C7_9	C8_9	Br2_9	-179.7(9)
C6_9	C7_9	C8_9	C9_9	1.1(19)

Atom	Atom	Atom	Atom	Angle/°
C7_9	C8_9	C9_9	C10_9	-0.2(18)
C8_9	C9_9	C10_9	C5_9	1.5(18)
C10_9	C5_9	C6_9	C7_9	4.4(17)
C11_9	C3_9	C4_9	C2_9	-110.8(8)
C11_9	C3_9	C17_9	C18_9	-104.9(10)
C11_9	C3_9	C17_9	C22_9	73.8(12)
C11_9	C12_9	C13_9	C14_9	2.0(16)
C12_9	C11_9	C16_9	C15_9	1.2(16)
C12_9	C13_9	C14_9	C15_9	-0.8(17)
C13_9	C14_9	C15_9	C16_9	-0.2(17)
C14_9	C15_9	C16_9	C11_9	0.0(16)
C16_9	C11_9	C12_9	C13_9	-2.2(16)
C17_9	C3_9	C4_9	C2_9	105.1(8)
C17_9	C3_9	C11_9	C12_9	104.7(10)
C17_9	C3_9	C11_9	C16_9	-74.5(11)
C17_9	C18_9	C19_9	C20_9	2.5(18)
C18_9	C17_9	C22_9	C21_9	-1.4(19)
C18_9	C19_9	C20_9	C21_9	0(2)
C19_9	C20_9	C21_9	C22_9	-3(3)
C20_9	C21_9	C22_9	C17_9	4(3)
C22_9	C17_9	C18_9	C19_9	-1.8(16)

Table 6: Hydrogen Fractional Atomic Coordinates (×10^4) and Equivalent Isotropic Displacement Parameters (Å^2×10^3) for Ru2BTPCP4_mP. U_{eq} is defined as 1/3 of the trace of the orthogonalised U_{ij}.

Atom	x	y	z	U_{eq}
H1WA	10295.15	2123.92	5194.01	117
H1WB	9704.65	2123.92	4805.99	117
H2WA	4729.33	-572.09	9763.31	121
H2WB	5270.76	-572.09	10236.89	121
H1S_1	9987.12	10944.28	5881.96	95
H1S_2	5573.42	8366.59	9233.16	115
H1S_3	4819.79	9730.18	6529.87	125
H1S_4	4647.59	9136.95	6544.24	125
H1A_5	6679.11	2387.36	5893.4	184
H1B_5	6002.73	3325.07	6007.3	184
H1C_5	5900.78	1776.93	6026.3	184
H2A_5	6219.32	3207.38	5106.48	147
H2B_5	6117.38	1659.56	5125.47	147
H3A_5	4874.86	2008.79	5396.92	147
H3B_5	4991.63	3532.97	5306.77	147
H4A_5	4990.17	1601.96	4515.83	147
H4B_5	5182.73	3094.6	4410.16	147
H5A_5	3972.2	3693.18	4635.56	147
H5B_5	3781.13	2191.75	4731.49	147
H6A_5	3356.46	2836.48	3910.4	184
H6B_5	4163.38	3249.21	3747.87	184
H6C_5	3972.25	1747.33	3843.82	184
H4A_6	7823.22	4494.87	6526.68	67
H4B_6	8675.55	4059.14	6393.51	67
H6_6	7090.23	5164.09	5903.71	69
H7_6	6063.48	6293.95	5640.19	84
H9_6	7157.95	9703.81	5752.35	80
H10_6	8221.11	8543.55	5939.24	57
H12_6	9230.96	4385.68	7363.22	91
H13_6	10359.27	4507.51	7777.44	97

Atom	x	y	z	U_{eq}
H14_6	11209.73	6048.37	7528.7	84
H15_6	10931.32	7519.82	6877.52	69
H16_6	9777.59	7496.13	6489.88	61
H18_6	7313.79	5443.96	7173.38	87
H19_6	6604.82	6697.75	7722.16	105
H20_6	6874.49	8900.37	7825.11	109
H21_6	7982.54	9690.97	7530.11	101
H22_6	8720.86	8442.16	7026.39	84
H4A_7	7972.04	5174.97	3293.84	51
H4B_7	8650.18	4391	3586.53	51
H6_7	7737.49	7255.19	3214.58	55
H7_7	7054.42	9176.95	3145.65	66
H9_7	7047.13	9479.15	4697.57	56
H10_7	7746.67	7614.01	4765.32	52
H12_7	8249.78	2305.22	3864.15	61
H13_7	8584.01	586.52	4413.93	62
H14_7	8393.3	757.38	5295.73	68
H15_7	8007.09	2739.69	5644.79	66
H16_7	7630.39	4438.47	5097.67	57
H18_7	6864.25	3878.88	3310.46	66
H19_7	5616.9	4204.72	3171.98	107
H20_7	4925.28	5224.58	3782.69	137
H21_7	5470.68	6147.3	4512.55	123
H22_7	6692.06	5720.04	4680.15	70
H4A_8	7911.8	1865.98	9338.28	77
H4B_8	7072.96	1418.42	9171.14	77
H6_8	8176.85	2647.27	10150.09	88
H7_8	8931.57	3902.28	10665.6	126
H9_8	7947.71	7173.54	10152.75	109
H10_8	7170.71	5925.07	9661.13	70
H12_8	7326.55	1841.83	8089.53	100
H13_8	6557.53	1815.72	7373.67	121
H14_8	5583.17	3250.19	7331.45	108
H15_8	5357.53	4713.07	8001.09	83
H16_8	6127.17	4740.32	8717.96	67
H18_8	8802.34	2923.2	9035.79	114
H19_8	9768.42	4334.33	8869.46	117
H20_8	9566.22	6344.26	8526.58	110
H21_8	8394.9	7005.71	8304.01	95
H22_8	7430.77	5603.65	8437.73	89
H4A_9	5760.86	2550.86	12152.41	63
H4B_9	5324.52	1781.94	11679.96	63
H6_9	6028.56	4548.7	12314.79	76
H7_9	6601.61	6475.04	12566.73	84
H9_9	7491.74	6814.61	11153.78	82
H10_9	6898.1	4936.36	10901.39	74
H12_9	5874.54	-365.01	11566.97	65
H13_9	5912.15	-2085.48	10971.55	70
H14_9	6614.21	-1870.36	10225.31	73
H15_9	7242.65	59.18	10061.66	69
H16_9	7168.19	1781.43	10651.22	68
H18_9	6715.4	1401.99	12516.45	72
H19_9	7793.98	1502.92	13002.77	82
H20_9	8821.83	2403.39	12651.59	106
H21_9	8812.87	3230.62	11818.44	137
H22_9	7776.71	2955.93	11307	94

Table 7: Atomic Occupancies for all atoms that are not fully occupied in Ru2BTPCP4_mP.

Atom	Occupancy	Atom	Occupancy	Atom	Occupancy
H1WA	0.5	C1S_4	0.5	C4_5	0.5
H1WB	0.5	H1S_4	0.5	H4A_5	0.5
H2WA	0.5	C1_5	0.5	H4B_5	0.5
H2WB	0.5	H1A_5	0.5	C5_5	0.5
Cl1S_3	0.5	H1B_5	0.5	H5A_5	0.5
Cl2S_3	0.5	H1C_5	0.5	H5B_5	0.5
Cl3S_3	0.5	C2_5	0.5	C6_5	0.5
C1S_3	0.5	H2A_5	0.5	H6A_5	0.5
H1S_3	0.5	H2B_5	0.5	H6B_5	0.5
Cl1S_4	0.5	C3_5	0.5	H6C_5	0.5
Cl2S_4	0.5	H3A_5	0.5		
Cl3S_4	0.5	H3B_5	0.5		

Table 8: Solvent masking (PLATON/SQUEEZE) information for Ru2BTPCP4_mP.

No	x	y	z	V	e	Content
1	0.000	0.015	0.000	657.4	164.1	3C6,3H14
2	0.389	0.520	0.324	8.1	0.0	?
3	0.611	0.520	0.676	8.1	0.0	?

Citations

CrysAlisPro (ROD), Rigaku Oxford Diffraction, Poland (?).

CrysAlisPro Software System, Rigaku Oxford Diffraction, (2024).

O.V. Dolomanov and L.J. Bourhis and R.J. Gildea and J.A.K. Howard and H. Puschmann, Olex2: A complete structure solution, refinement and analysis program, *J. Appl. Cryst.*, (2009), **42**, 339-341.

Sheldrick, G.M., Crystal structure refinement with ShelXL, *Acta Cryst.*, (2015), **C71**, 3-8.

NMR Spectra for Novel Compounds

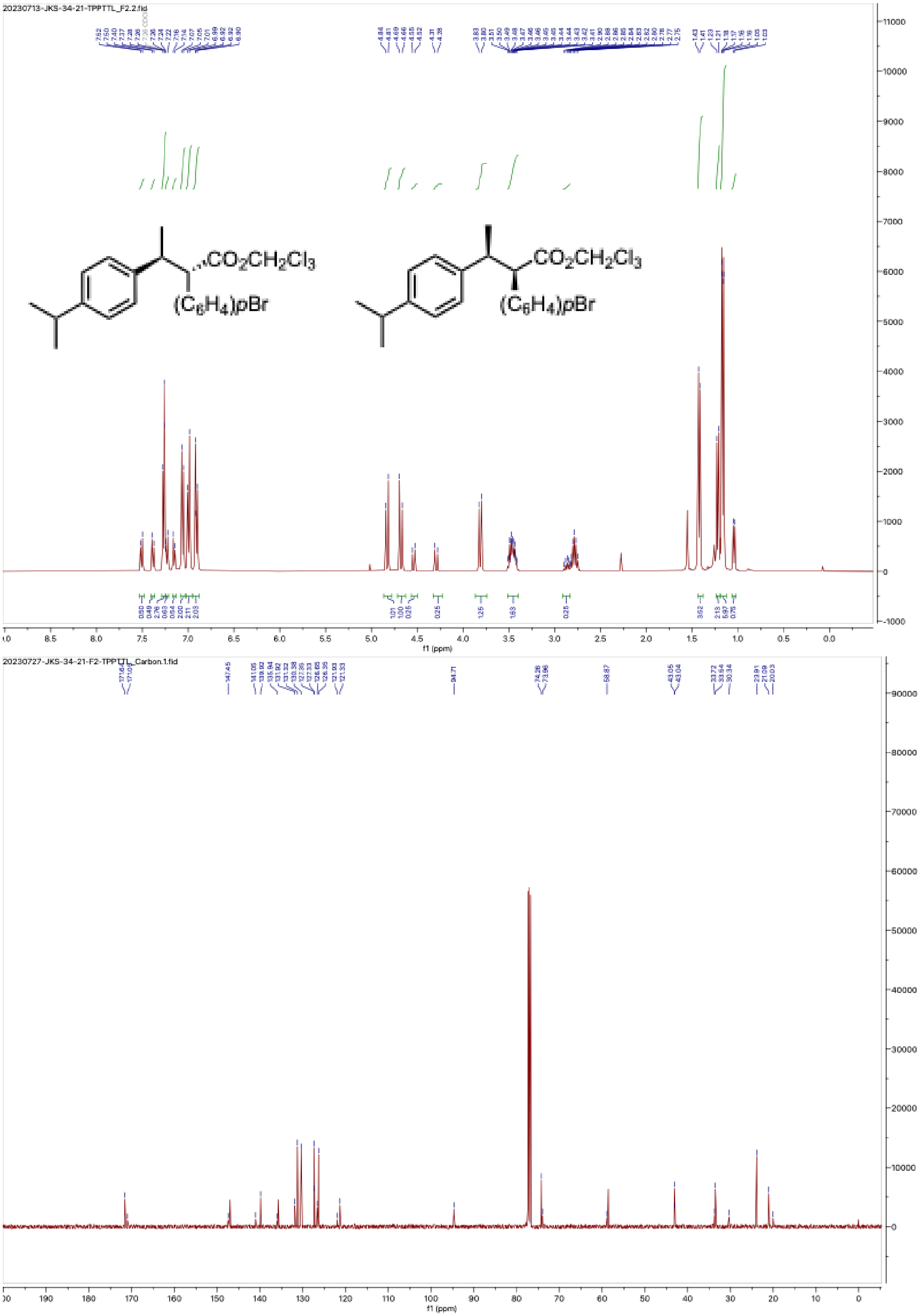
20230713-JKS-34-21-TPPTTL_F2.2.fid
f1 (ppm)
20230727-JKS-34-21-F2-TPPTTL_Carbon.1.fid
f1 (ppm)

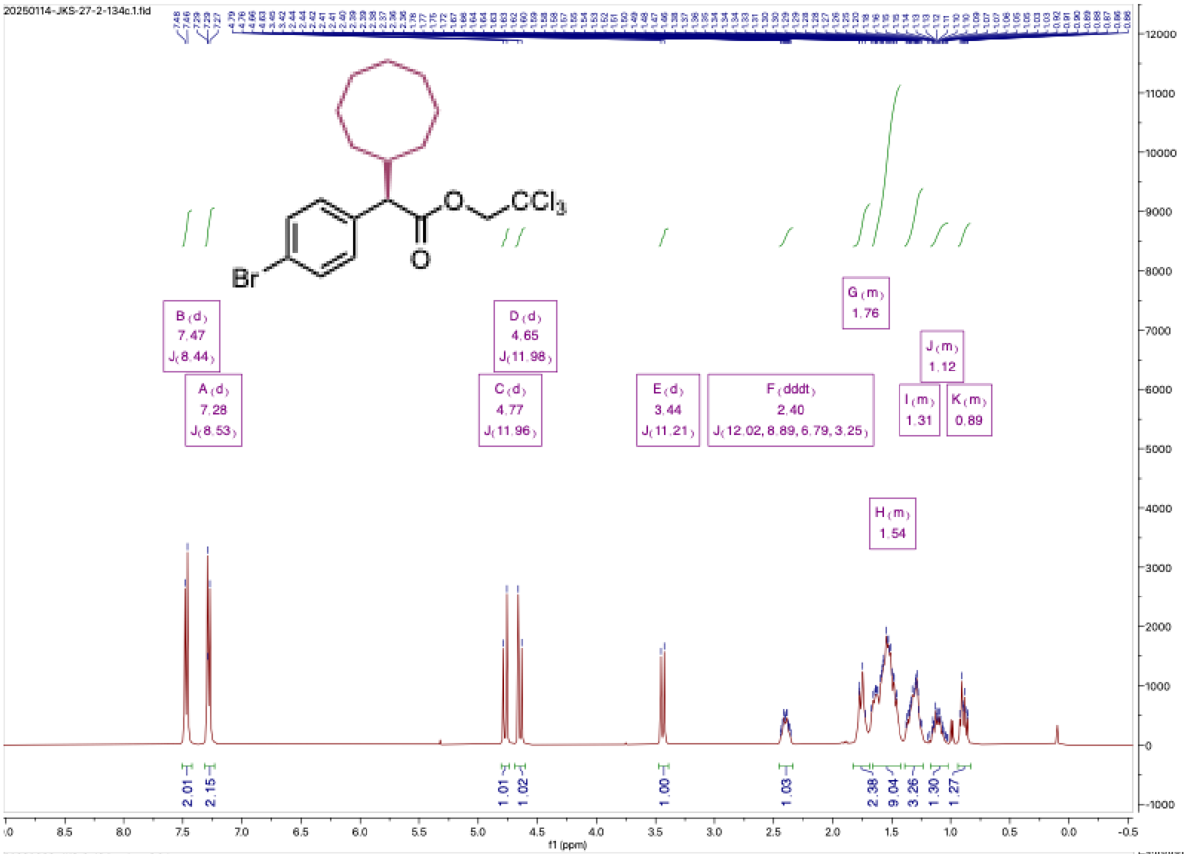

20250114-JKS-27-2-134c.1.fid
Br
CCl3
O
A (d) 7.28 J(8.53)
B (d) 7.47 J(8.44)
C (d) 4.77 J(11.96)
D (d) 4.65 J(11.98)
E (d) 3.44 J(11.21)
F (dddt) 2.40 J(12.02, 8.89, 6.79, 3.25)
G (m) 1.76
H (m) 1.54
I (m) 1.31
J (m) 1.12
K (m) 0.89
2.01
2.15
1.01
1.02
1.00
1.03
2.38
9.04
3.26
1.30
1.27
f1 (ppm)

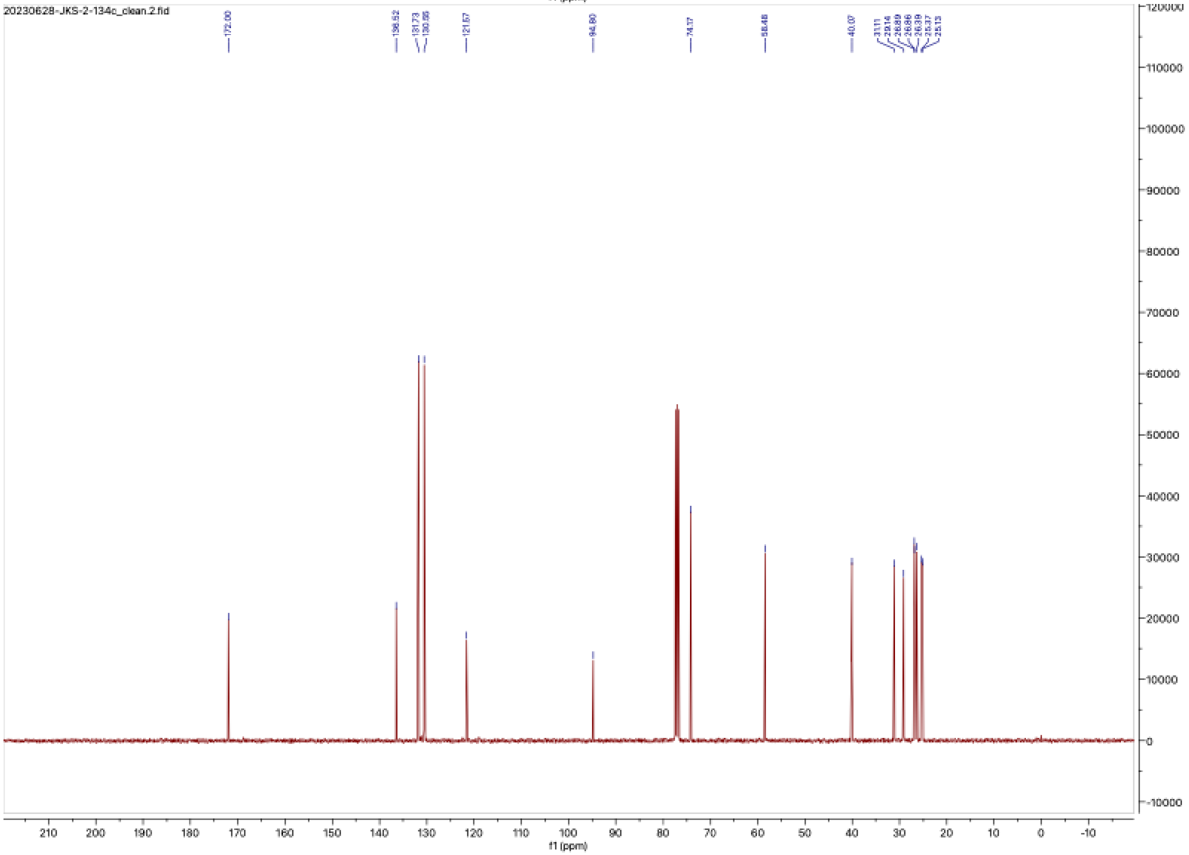

20230628-JKS-2-134c_clean.2.fid
172.00
136.52
131.73
130.55
121.57
94.80
74.17
58.48
40.07
31.11
29.14
26.89
26.86
26.39
25.37
25.13
f1 (ppm)

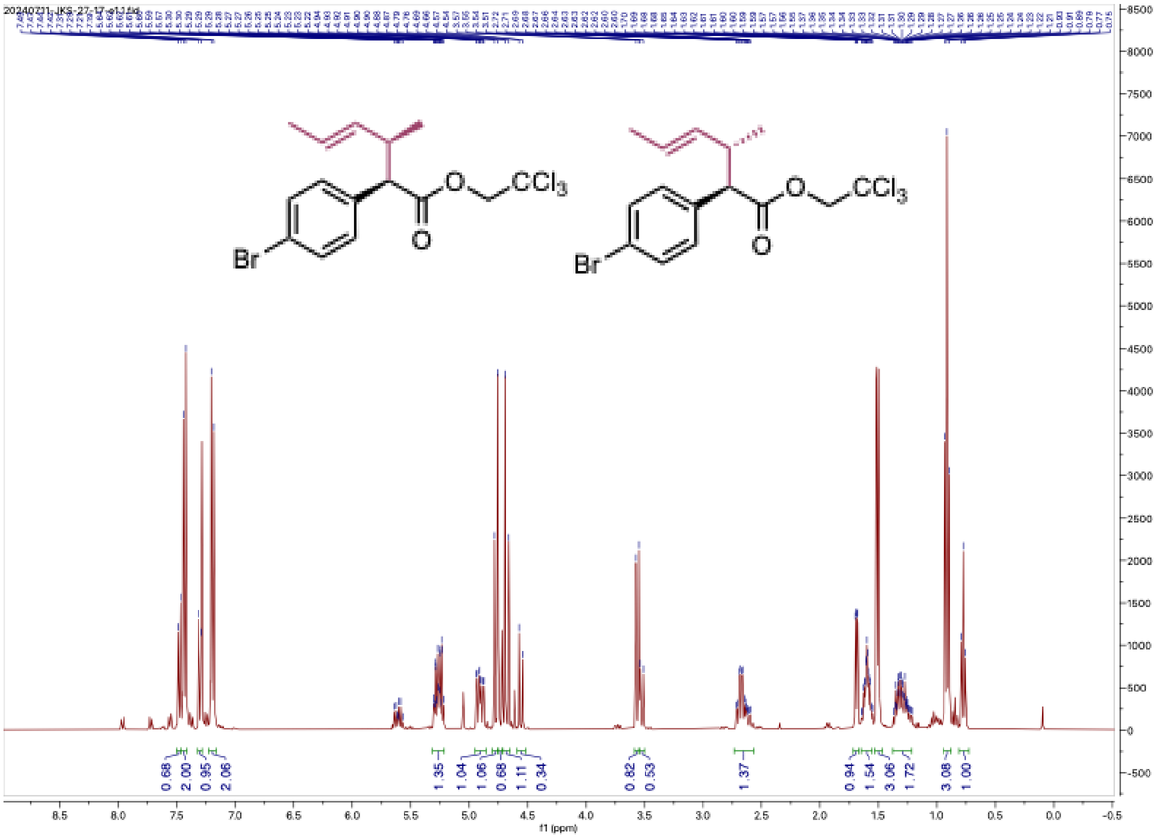

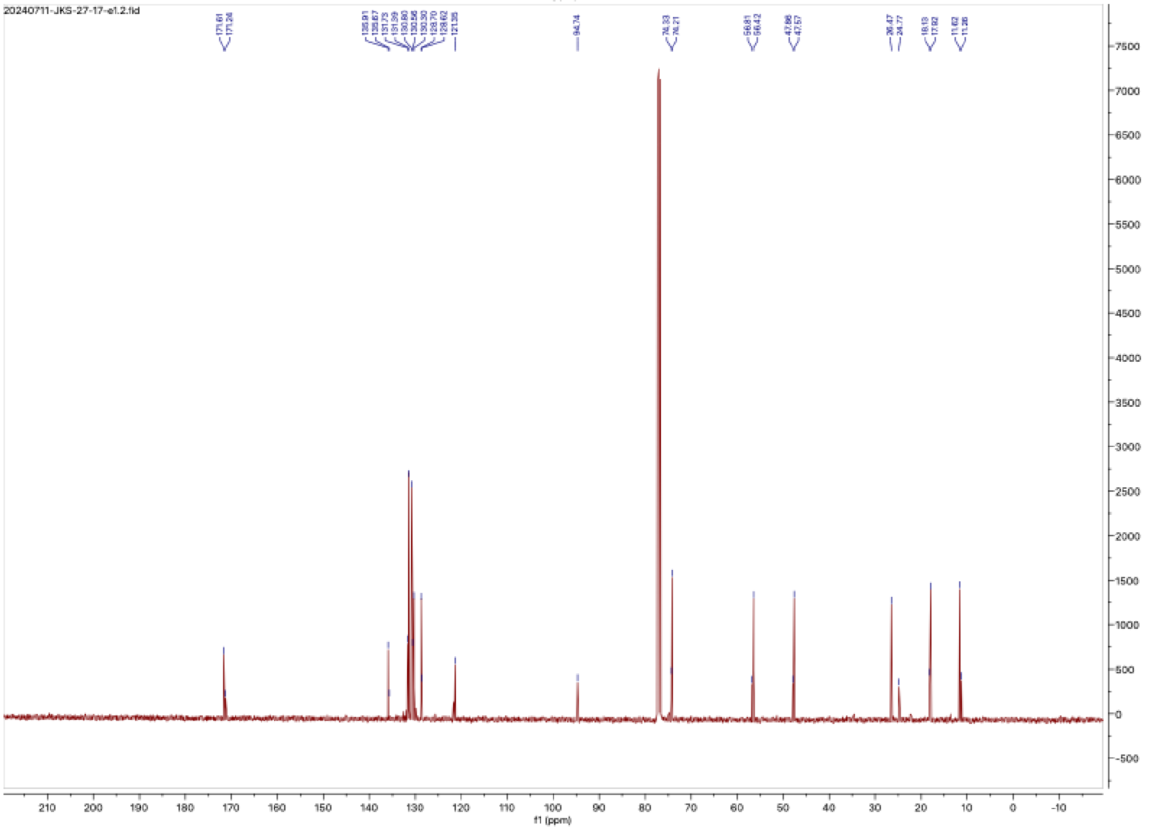

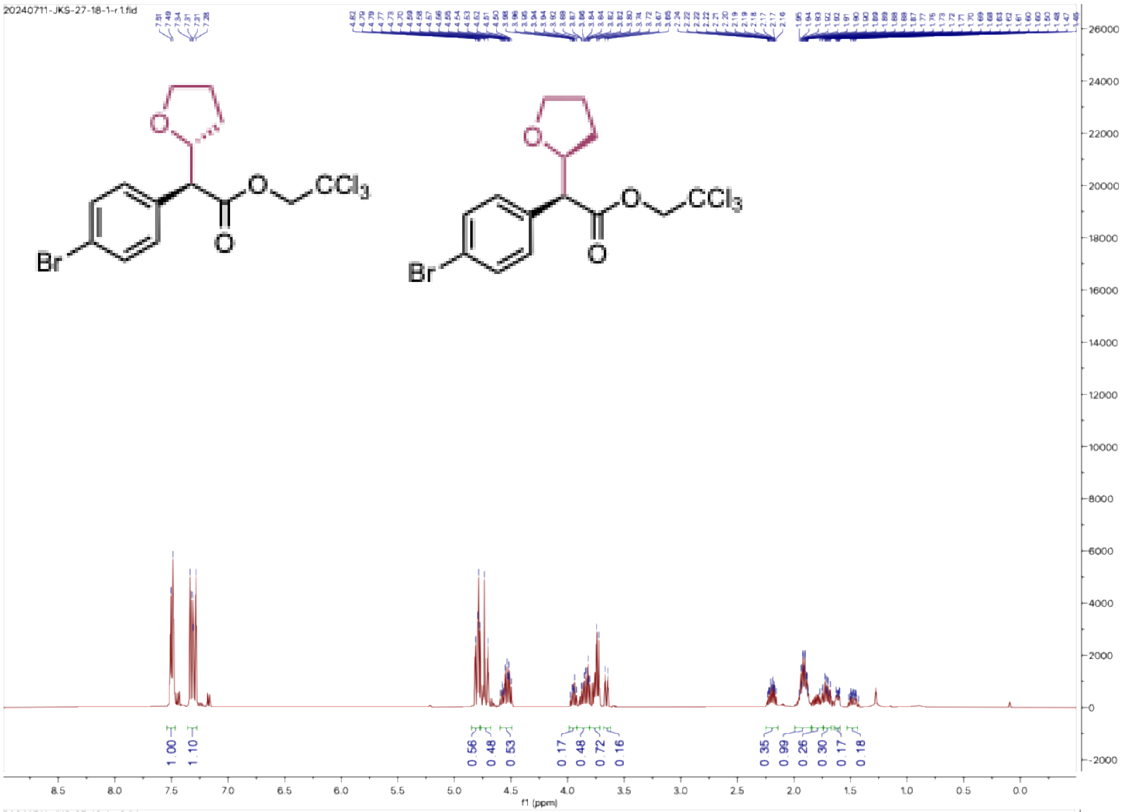
20240711-JKS-27-18-1-r.1.fid
Br
CCl3
O
f1 (ppm)

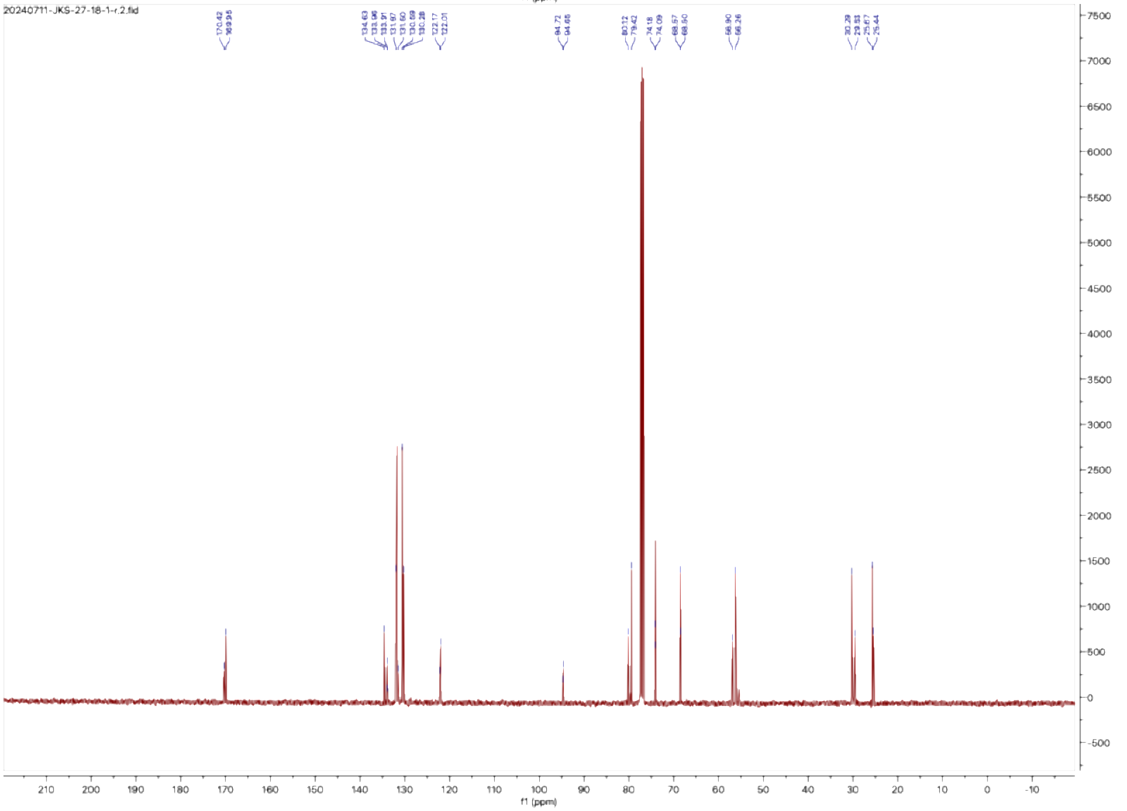
20240711-JKS-27-18-1-r.2.fid
f1 (ppm)

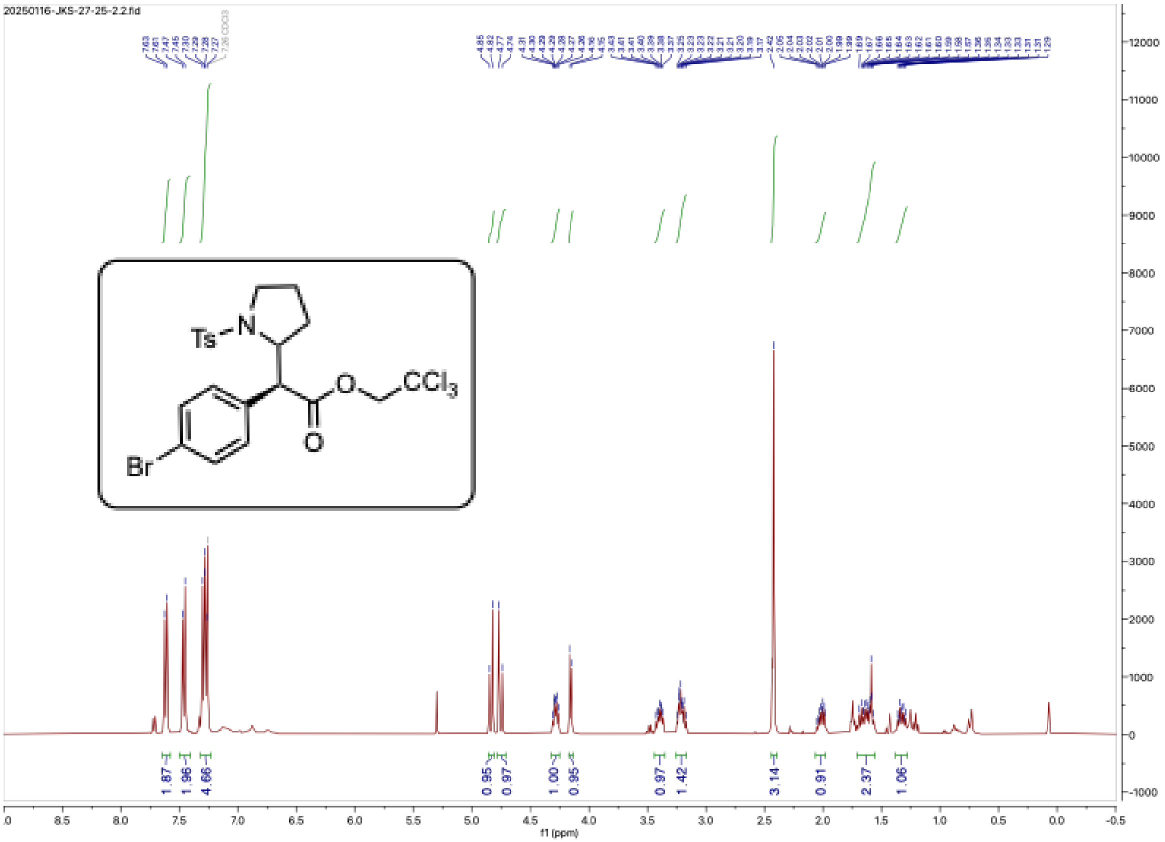
20250116-JKS-27-25-2.2.fid
Ts
N
O
CCl3
Br
f1 (ppm)

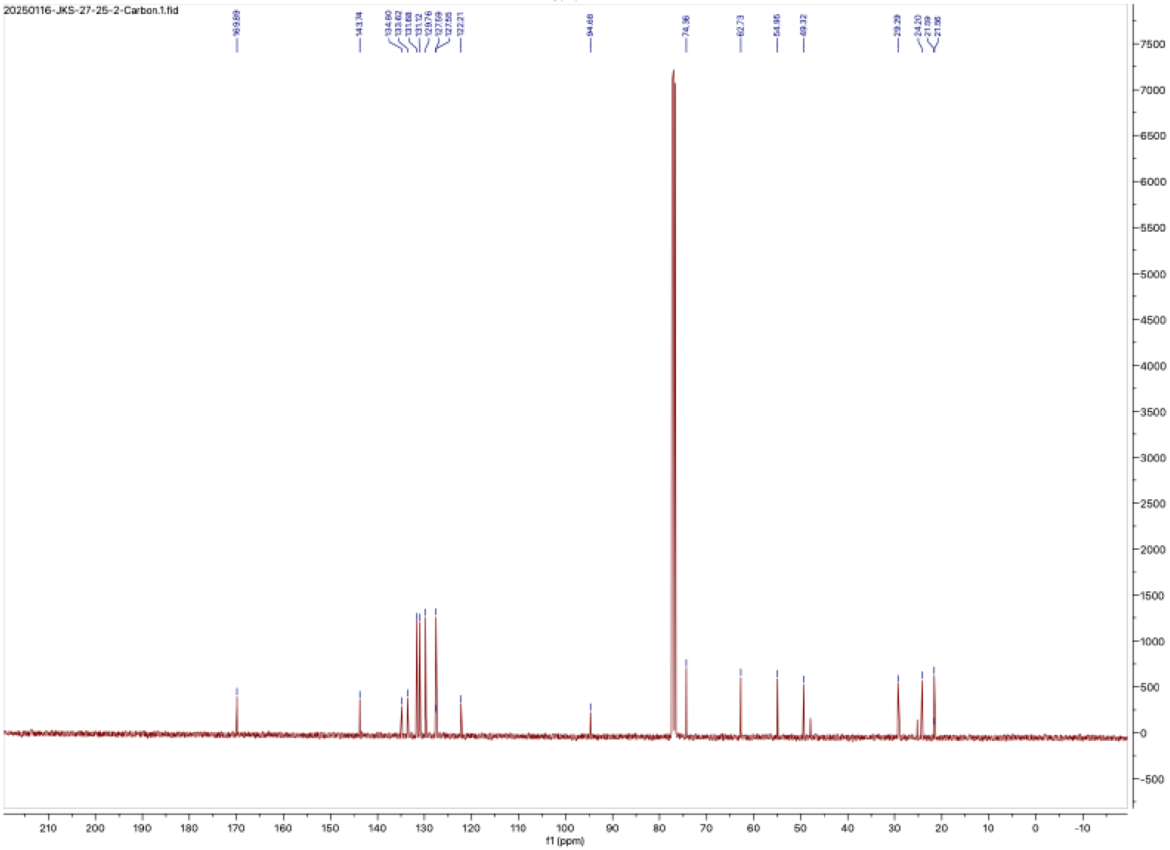
20250116-JKS-27-25-2-Carbon.1.fid
f1 (ppm)

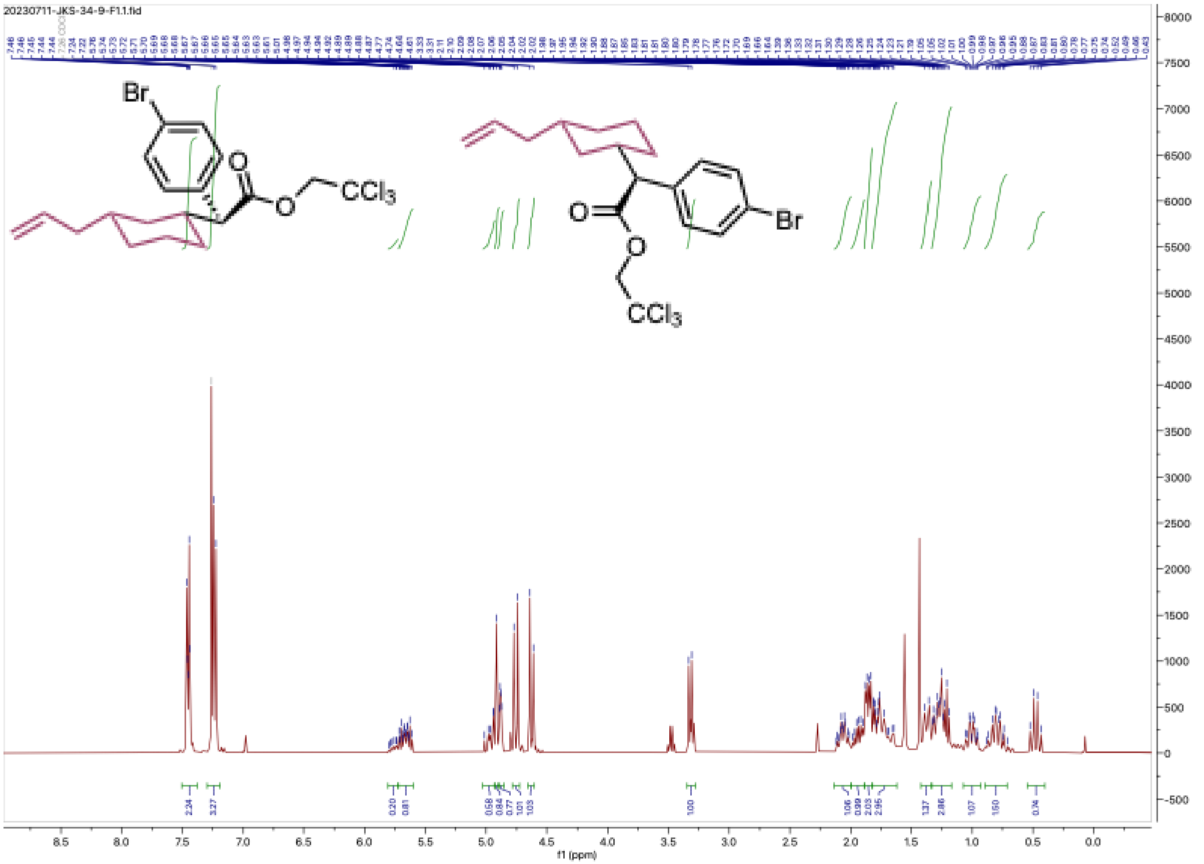
20230711-JKS-34-9-F1.1.fid
Br
O
O
CCl3
f1 (ppm)

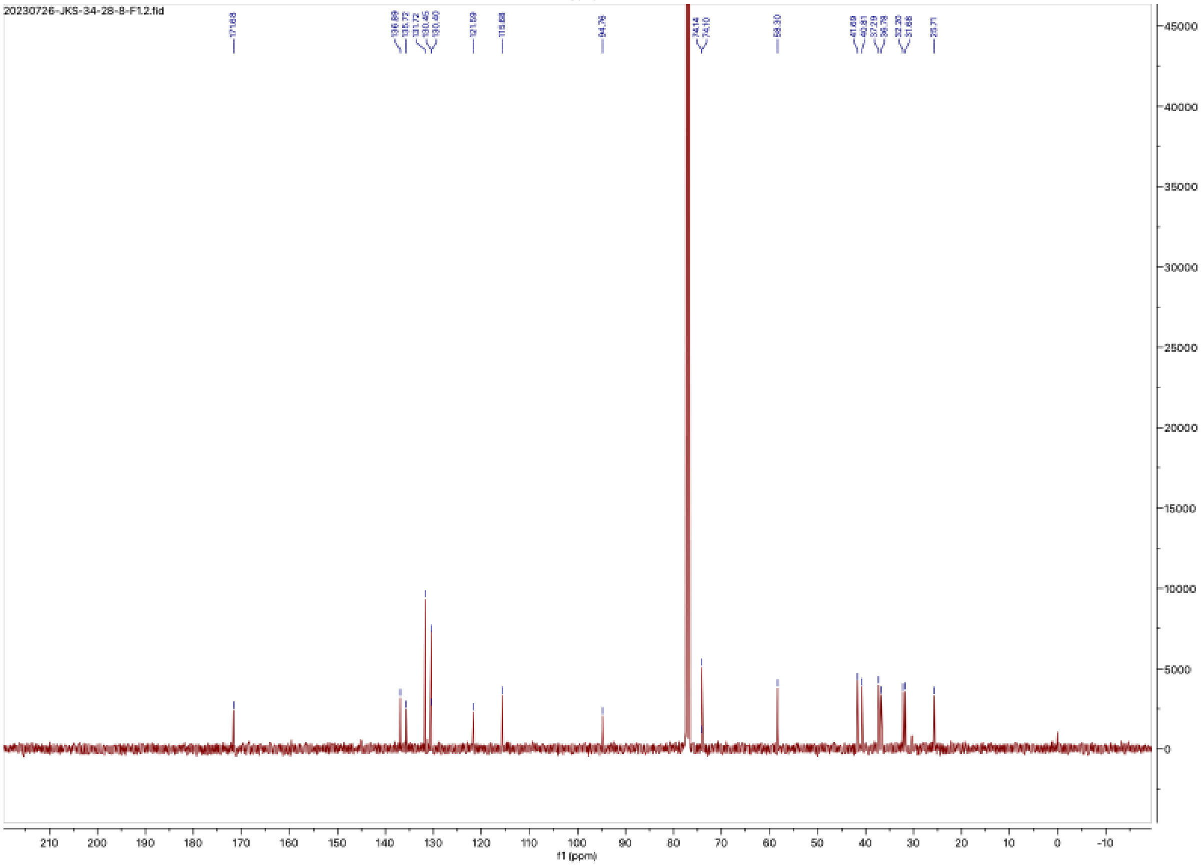
20230726-JKS-34-28-8-F1.2.fid
171.68
136.89
135.72
131.72
130.45
130.40
121.59
115.68
94.76
74.14
74.10
58.30
41.69
40.81
37.29
36.78
32.20
31.68
25.71
f1 (ppm)

References

1. Green, S. P.; Wheelhouse, K. M.; Payne, A. D.; Hallett, J. P.; Miller, P. W.; Bull, J. A., Thermal Stability and Explosive Hazard Assessment of Diazo Compounds and Diazo Transfer Reagents. *Org. Process Res. Dev.* **2020,** *24* (1), 67-84.
2. Chen, Z.; Shimabukuro, K.; Bacsa, J.; Musaev, D. G.; Davies, H. M. L., D4-Symmetric Dirhodium Tetrakis(binaphthylphosphate) Catalysts for Enantioselective Functionalization of Unactivated C–H Bonds. *J. Am. Chem. Soc.* **2024,** *146* (28), 19460-19473.
3. Sailer, J. K.; Sharland, J. C.; Bacsa, J.; Harris, C. F.; Berry, J. F.; Musaev, D. G.; Davies, H. M. L., Diruthenium Tetracarboxylate-Catalyzed Enantioselective Cyclopropanation with Aryldiazoacetates. *Organometallics* **2023,** *42* (15), 2122-2133.
4. Fu, J.; Ren, Z.; Bacsa, J.; Musaev, D. G.; Davies, H. M. L., Desymmetrization of cyclohexanes by site- and stereoselective C–H functionalization. *Nature* **2018,** *564* (7736), 395-399.
5. Liao, K.; Negretti, S.; Musaev, D. G.; Bacsa, J.; Davies, H. M. L., Site-selective and stereoselective functionalization of unactivated C–H bonds. *Nature* **2016,** *533* (7602), 230-234.
6. Wertz, B.; Ren, Z.; Bacsa, J.; Musaev, D. G.; Davies, H. M. L., Comparison of 1,2-Diarylcyclopropanecarboxylates with 1,2,2-Triarylcyclopropanecarboxylates as Chiral Ligands for Dirhodium-Catalyzed Cyclopropanation and C–H Functionalization. *J. Org. Chem.* **2020,** *85* (19), 12199-12211.
7. Tortoreto, C.; Rackl, D.; Davies, H. M. L., Metal-Free C–H Functionalization of Alkanes by Aryldiazoacetates. *Org. Lett.* **2017,** *19* (4), 770-773.
